# Fabrication of Li_4_Ti_5_O_12_ (LTO) as Anode Material for Li-Ion Batteries

**DOI:** 10.3390/mi15030310

**Published:** 2024-02-23

**Authors:** Christian M. Julien, Alain Mauger

**Affiliations:** Institut de Minéralogie, de Physique des Matériaux et de Cosmochimie (IMPMC), Sorbonne Université, CNRS-UMR 7590, 4 place Jussieu, 75252 Paris, France; alain.mauger@sorbonne-universite.fr

**Keywords:** spinel structure, lithium titanate, anode material, Li-ion batteries, synthesis processes

## Abstract

The most popular anode material in commercial Li-ion batteries is still graphite. However, its low intercalation potential is close to that of lithium, which results in the dendritic growth of lithium at its surface, and the formation of a passivation film that limits the rate capability and may result in safety hazards. High-performance anodes are thus needed. In this context, lithium titanite oxide (LTO) has attracted attention as this anode material has important advantages. Due to its higher lithium intercalation potential (1.55 V vs. Li^+^/Li), the dendritic deposition of lithium is avoided, and the safety is increased. In addition, LTO is a zero-strain material, as the volume change upon lithiation-delithiation is negligible, which increases the cycle life of the battery. Finally, the diffusion coefficient of Li^+^ in LTO (2 × 10^−8^ cm^2^ s^−1^) is larger than in graphite, which, added to the fact that the dendritic effect is avoided, increases importantly the rate capability. The LTO anode has two drawbacks. The energy density of the cells equipped with LTO anode is lower compared with the same cells with graphite anode, because the capacity of LTO is limited to 175 mAh g^−1^, and because of the higher redox potential. The main drawback, however, is the low electrical conductivity (10^−13^ S cm^−1^) and ionic conductivity (10^−13^–10^−9^ cm^2^ s^−1^). Different strategies have been used to address this drawback: nano-structuration of LTO to reduce the path of Li^+^ ions and electrons inside LTO, ion doping, and incorporation of conductive nanomaterials. The synthesis of LTO with the appropriate structure and the optimized doping and the synthesis of composites incorporating conductive materials is thus the key to achieving high-rate capability. That is why a variety of synthesis recipes have been published on the LTO-based anodes. The progress in the synthesis of LTO-based anodes in recent years is such that LTO is now considered a substitute for graphite in lithium-ion batteries for many applications, including electric cars and energy storage to solve intermittence problems of wind mills and photovoltaic plants. In this review, we examine the different techniques performed to fabricate LTO nanostructures. Details of the synthesis recipes and their relation to electrochemical performance are reported, allowing the extraction of the most powerful synthesis processes in relation to the recent experimental results.

## 1. Introduction

**Figure 1:**
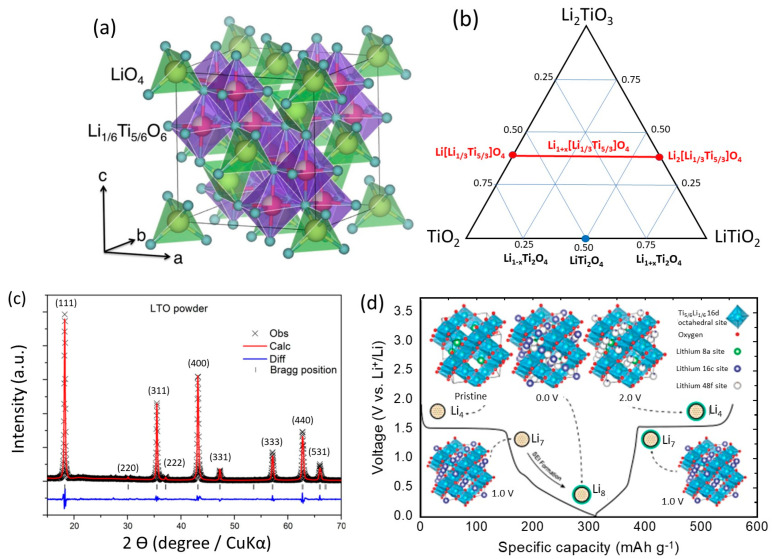
(**a**) The crystal structure of spinel Li_4_Ti_5_O_12_. The tetrahedral A site is occupied by Li^+^, while the octahedral B site is occupied by 1/6 Li^+^ and 5/6 Ti^4+^. Reproduced from Ref. [13]. Copyright 2017 under the terms of the Creative Commons Attribution 4.0 Int. License. (**b**) Ternary phase diagram for the Li–Ti–O system in the region bounded by Li_2_TiO_3_, TiO_2_, and LiTiO_2_. The line corresponding to the lithiation process between Li[Li_1/3_Ti_5/3_]O_4_ and Li_2_[Li_1/3_Ti_5/3_]O_4_ occurs at 1.55 V vs. Li^+^/Li. (**c**) Rietveld refinement of XRD pattern of spinel LTO powders with space group *Fd*3*m*. Reproduced with permission from [14]. (**d**) Schematic of the energy storage mechanism of the spinel LTO host lattice Reproduced with permission from [12]. Copyright 2019 under the ACS Free to read License.

Lithium-ion batteries (LIBs) are rechargeable power sources built on the idea of the flow of Li^+^ ions back and forth between two intercalation electrodes with different redox potentials [1]. In most commercial LIBs, the negative electrode (anode) is a carbon-based material (e.g., graphite) and the positive electrode (cathode) is a lithiated transition-metal oxide. Graphite has the advantages of exceptional kinetics, low cost, and high abundance, but its low Li-intercalation potential (approaching 0 V vs. Li^+^/Li towards the end of charging) poses serious safety issues related to Li dendrite growth on the anode surface during the overcharge process. Therefore, extensive research has been conducted on the alternative anode [2]. Titanium-based materials (i.e., TiO_2_, LiTi_2_O_4_, Li_2_TiO_3_, Li_4_Ti_5_O_12_) belong to the class of alternative materials to graphite as anode materials of LIBs that operate at a potential above 0.8 V vs. Li^+^/Li, where a stable solid-electrolyte interphase (SEI) layer is not required. Since the 1950s, Jonker reported the first study of spinel Li_4_Ti_5_O_12_, which is a stable phase of the Li_2_O–TiO_2_ system also denoted as Li_1.33_Ti_1.67_O_4_, Li_4/3_Ti_5/3_O_4_, or [Li]_8a_[Li_1/3_Ti_5/3_]_16d_[O_4_]_32e_. Li_4_Ti_5_O_12_ (with the popular acronym LTO) appeared in the form of a well-crystallized black powder, in which the titanium is in the oxidation state +4 [3]. Subsequently, Deschanvres et al. [4] first reported a comprehensive structural study of single-crystal LTO. Materials with a spinel structure have a general chemical formula of *AB*_2_O_4_, in which the oxygen ions (the Wyckoff 32*e* sites in the space group *Fd*3*m* (No. 227)) form a cubic-close-packed (ccp) array with tetrahedrally (8*a*, 8*b*, 48*c*) and octahedrally (16*c*, 16*d*) coordinated interstices partially occupied by the *A* and *B* cations (Figure 1a). In 1973, Li_3+y_Ti_6−y_O_12_ spinels (0 ≤ y ≤ 1) were under investigation because of their superconductivity at a relatively high transition temperature [5]. Interestingly, by tuning the oxygen concentration in the sample preparation, the spinel phase changes from LiTi_2_O_4−δ_ to Li_4_Ti_5_O_12_ along with the superconductor-insulator phase transition. In 1983, Murphy et al. first reported the capability of Li_4_Ti_5_O_12_ (*a* = 8.357 Å) to react with n-butyllithium (n-BuLi) giving Li_7_Ti_5_O_12_ (*a* = 8.368 Å), in which all the lithium ions are likely on octahedral sites [6]. Figure 1b shows the ternary phase diagram between Li_2_TiO_3_, TiO_2_, and LiTiO_2_, on which the composition Li[Li_1/3_Ti_5/3_]O_4_ is converted to Li_2_[Li_1/3_Ti_5/3_]O_4_ upon lithiation corresponding to the reaction:

[Li]_8a_[Li_1/3_Ti_5/3_]_16d_[O_4_]_32e_ + Li^+^ + e^−^ ⇆ [□]_8a_[Li_2_]_16c_[Li_1/3_Ti_5/3_]_16d_[O_4_]_32e_. (1)

Since the 1980s, LTO has been widely investigated as a Li insertion compound. Colbow et al. first measured the galvanostatic charge–discharge properties of a Li//Li_4_Ti_5_O_12_ half-cell and found that LTO reacted with one Li atom [7]. In 1995, Ferg et al. [8] evaluated the LTO anode associated with spinel-type cathodes (Li_1.03_Mn_1 97_O_4_ and LiZn_0.025_Mn_1 95_O_4_) or LiCoO_2_ in room-temperature lithium cells. In 2001, Amatucci et al. [9] introduced the aqueous asymmetric Li_4_Ti_5_O_12_//AC cell as a safer hybrid pseudocapacitor showing 10–15% capacity loss after 5000 cycles at 10C charge/discharge rates. A typical plot of the Rietveld refinement of the X-ray diffraction (XRD) pattern of pristine LTO powders is displayed in Figure 1c.

The Li_4_Ti_5_O_12_ spinel-framework structure (white in color, *Fd*3*m*, *a* = 8.36 Å) can be electrochemically reduced to Li_2_[Li_1/3_Ti_5/3_]O_4_ (dark blue, *Fd*3*m*, *a* = 8.37 Å) at a voltage of 1.55 V and the reaction is highly reversible. Ohzuku and co-workers [10] reported that the lattice dimension, determined by X-ray diffraction measurements, did not change during the reaction in Equation (1). Lithium insertion causes a first-order displacement of the tetrahedrally-coordinated Li ions in the Li[Li_1/3_Ti_5/3_]O_4_ framework into octahedral sites to generate the ordered rock-salt phase Li_2_[Li_1/3_Ti_5/3_]O_4_ (Li_7_Ti_5_O_12_). During lithium insertion (discharging), three Li atoms move from 8*a* sites to 16*c* sites, and the inserted three Li ions move to the 16*c* sites via 8*a* sites with the simultaneous redox reaction of Ti^4+^/Ti^3+^ [11]. From experiment investigations of the structural properties of the lithiated Li_4_Ti_5_O_12_ electrode using neutron diffraction, Liu et al. [12] demonstrated that Li_4_Ti_5_O_12_ transforms to [Li_0.16_]_8a_[Li_1_Ti_5_]_16d_[Li_5.84_]_16c_[O_12_]_32e_ (*Fd*3*m* S.G.) after the 1.55 V plateau in the discharge. Continued discharging of LTO to 0.01 V not only takes one extra Li into the LTO bulk but also promotes electrolyte reduction at the LTO surface to form a thick SEI layer. The fully discharged LTO is noted as [Li_0.62_]_8a_[Li_1_Ti_5_]_16d_[Li_6_]_16c_[Li_0.38_]_48f_[O_12_]_32e_ (*Fd*3*m* S.G.), with 8*a* and 48*f* sites being partially occupied (Figure 1d) [12,13,14]. The reaction (Equation (1) is called a zero-strain insertion reaction. Thus, negative electrodes made of Li_4_Ti_5_O_12_ material can undergo many hundreds of cycles without structural disintegration. Lithium dendrite growth is completely avoided at this moderate voltage, enhancing the safety of the battery [15]. Furthermore, the voltage of a Li//Li_4+x_Ti_5_O_12_ cell changes abruptly at the end of discharge and charge. Thus, a Li_4+x_Ti_5_O_12_ spinel electrode provides very sharp end-of-charge and end-of-discharge indicators, which are useful for controlling cell operation and preventing overcharge abuse and over-discharge.

Due to its outstanding rate capability, the LTO battery is most promising to equip devices that require rapid charge and discharge. LTO has the ability for a recharge efficiency of up to 98%, which is considerably more than conventional energy storage mechanisms, and can be extensively used in electric vehicle (EV) charging stations, renewable energy storage power, etc. Li-ion batteries based on the Li_4_Ti_5_O_12_//LiFePO_4_ full cell, with nanostructured carbon-coated electrodes operating at a flat voltage of 1.85 V are a promising option for potential stationary storage and electric vehicle applications [16,17,18]. A Li_4_Ti_5_O_12_//LiFePO_4_ 18650-type battery, which delivered a capacity of 800 mAh, showed outstanding cycling stability, with a definitively stable capacity after 20,000 cycles at a 10C (6 min) charge rate and a 5C (12 min) discharge rate, and even 95% capacity retention was observed after 30,000 cycles at a 15 C (4 min) charge rate and 5C discharge-rate. This battery was used in a real car with the charge time reduced to 5 min using a three-level charger in parallel (500 V, 125 A) [18]. Another aspect to be considered in the fast charge/discharge process is the natural increase of temperature of the batteries, which must be preserved to a precise limit to avoid thermal runaway. In practice, it is critical to keep the Li-ion cells at an operating temperature of 25–40 °C and a temperature uniformity of less than 5 °C for batteries used in EVs. Fast recharge usually requires a liquid cooling system used in different EVs, for instance in Tesla, BYD, and BMW i8 cars. In this context, the LTO battery, which consists of a Li(Ni_1−y−z_Co_y_Mn_z_)O_2_ (named NCM) cathode and LTO anode, is used as a model to study the efficiency of different cooling systems [19,20]. Recently, Behi et al. studied experimentally a hybrid thermal management system (TMS) using phase change material (PCM) heat buffer plate and liquid cooling on such a LTO battery consisting of 30 23 Ah prismatic cells connected in series with no space between the cells [21]. The tests, conducted at a constant initial temperature of 25 °C and 4C charging/discharging rate between 10% and 90% of SOC, show that the temperature of each cell never exceeds 32 °C for the charging and discharging process. Moreover, this hybrid TMS was effective in providing temperature uniformity inside the module. It thus allows commercial use of the LTO battery at a 4C rate both for the charge and the discharge. LTO is also a competitive choice for grid-scale energy storage systems (ESS). Yang et al. developed a 10 Ah lithium–titanate battery with lithium cobalt oxide–lithium nickel cobalt manganese oxide dual-phase cathode and investigated its application in 100 kWh-level ESS. This battery demonstrated a specific capacity of 79 Wh kg^−1^ with a high-capacity retention rate of 91.8% after 1000 cycles at 55 °C and >80% capacity retention at 15 C_cell_. The 125 kWh ESS shows an energy efficiency of 97.82% and 89.97% at 0.2 and 1.5 C_System_, respectively. Self-discharge and constant power tests confirmed that this battery is suitable for storing intermittent power from renewable sources such as solar and wind, and its ability to smooth the fluctuating power is confirmed by coordinating with a wind power system [22]. The use of NCM as the cathode is the first solution to increase the energy density of the LTO-based lithium-ion batteries since NCM belongs to the family of high-voltage cathode elements, compensating for the operating voltage of 1.55 V of LTO. Another high-voltage cathode element that has been considered is the spinel LiNi_0.5_Mn_1.5_O_4_ (LNMO) cathode having a high voltage of 4.7 V vs. Li^+^/Li. The use of LNMO has been postponed due to the oxidation of electrolytes present on the surface of LNMO under a high voltage and Mn cation dissolution. However, Piao et al. solved this problem by designing a nonflammable all-fluorinated electrolyte denoted as AFE with a composition of 1 mol L^−1^ lithium hexafluorophosphate (LiPF_6_) in a mixture of fluoroethylene carbonate (FEC), 3,3,3-fluoroethylmethyl carbonate (FEMC), 1,1,2,2-tetrafluoroethyl-2,2,3,3-tetrafluoropropylether (TTE) and 0.02 mol L^−1^ lithium difluoro(oxalato)borate (LiDFOB) [23]. At 5C, The LNMO//LTO cell is a high-energy cell with an operating voltage of 3.3 V. With this electrolyte, it delivered a capacity of 114 mAh g^−1^ with a capacity retention of 90.3% and an average Coulombic efficiency of 99.9% after 1500 cycles.

The performance of the LTO anode was achieved by overcoming the drawback of Li_4_Ti_5_O_12_ material, namely its low intrinsic electronic conductivity (ranging from 1.0 × 10^−8^ to 1.0 × 10^−7^ S cm^−1^, arising from the empty Ti 3*d* state), which prevents its electrochemical rate performances [24,25,26,27]. One solution is the nano-structuration of LTO to reduce the path of Li^+^ ions and electrons inside LTO. The reduction of the crystallite size *L* to nanometer scale can decrease the duration *τ* of the insertion (discharge) and deinsertion (charge) reaction (Equation (2)):

(2) $$\tau =\frac{L^{2}}{4\pi \tilde{D}},$$

where $\tilde{D}$ is the diffusion coefficient of ions. It is believed that the kinetically induced effective two-phase reaction in Li_4+x_Ti_5_O_12_, resulting from the mixed 8*a*/16*c* occupation, is most likely responsible for the high-rate capabilities [28]. Nanosizing can also increase the interfacial surface contact between the electrode and electrolyte, leading to a high charge-discharge rate [29]. Kavan et al. [30] analyzed systematically the dependence of the particle size on the electrochemical properties of the LTO using particles ranging from 1 µm to 9 nm in thin electrode films. They found an optimum battery performance for particles with a size around 20 nm (∼100 m^2^ g^−1^) in a potential interval of 2.5–0.9 V. Improved rate performance of LTO anode can be also achieved by other strategies such as: (i) modification of the material morphology (i.e., particle shape, porosity, aggregates), (ii) introduction of an electronically conductive phase such as carbon, metal or metal oxide, and (iii) doping with foreign cations or anions, which can change the energy level distribution. Several promising routes have been recently suggested in a series of works aiming to rational design of LTO anodes [31], in relation to the electrochemical properties [32,33,34,35,36,37].

**Scheme 1:**
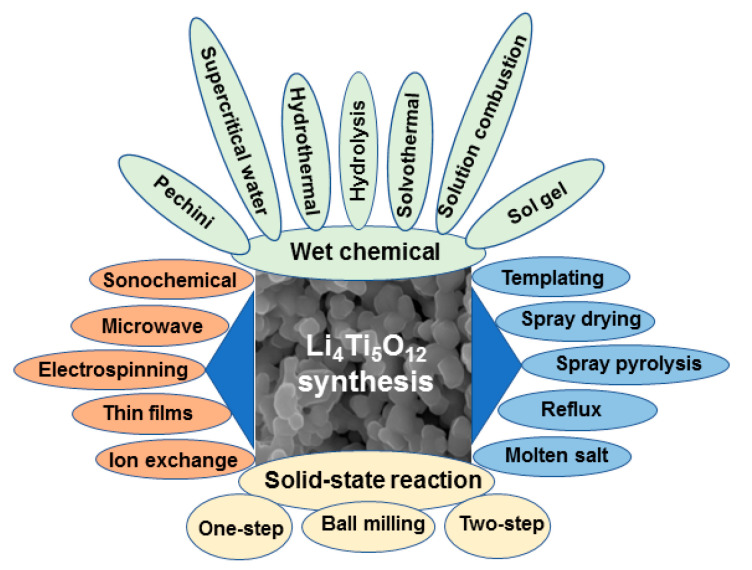
Graphical abstract giving a brief introduction about the focus of the review paper.

In this review, we examine the different techniques performed to fabricate LTO nanostructures. Details of the synthesis recipes and their relation to electrochemical performance are reported, allowing the extraction of the most powerful synthesis processes in relation to the recent experimental results (See Scheme 1). The electrochemical properties of LTO are dependent on synthesizing methods to a great extent. The synthesis of LTO with the appropriate structure and the optimized doping and the synthesis of composites incorporating conductive materials is thus the key to achieving high-rate capability. That is why a variety of synthesis recipes have been published on the LTO-based anodes. For each synthesis route, various parameters should be considered: starting reagents (e.g., titanium precursor or lithium salt), morphology of the precursor, presence of additive, reaction temperatures and reaction time, duration, temperature, and atmosphere of calcination, etc. The present article aims to review the techniques of fabrication of LTO anode materials. The influence of reaction conditions on the products is investigated in detail. It is shown that uniform, monodisperse, and stable mesoporous nanoparticles can be prepared in mild synthesis conditions and the particle size can be controlled from 25 to 200 nm by varying synthesis parameters and adding suitable additive agents including alcohols, amine, inorganic bases, and inorganic salts. Novel synthesis methods are also used to control the particle size (see Refs. [38,39,40,41,42]). In this work, we examine the different techniques performed to fabricate LTO nanostructures. Details of the synthesis recipes and their relation to the electrochemical performance are reported, allowing the extraction of the most powerful synthesis processes in relation to the recent experimental results.

## 2. Synthesis Methods

Various strategies have been reported for the synthesis of LTO with the aim of finding facile and simple processes for large-scale production. The main objective is the development of a method, which would provide a product with good homogeneity, regular morphology, and narrow particle size distribution. For instance, Wang et al. [34] reported that the synthesis of monodisperse LTO nanospheres is the key to reducing the irreversible capacity of LTO materials, which can be solved by the pH regulation of the growth process. This process provides LTO anode materials with high electrochemical performance (109 mAh g^−1^ at 60C rate; 92% retention after 500 cycles). Additionally, the high-temperature post-treatment is not desirable in terms of fabrication expenses. Conventional synthesis techniques include solid-state reaction (SSR) [25,43,44,45,46,47,48,49], hydrothermal [50,51,52,53,54,55,56,57,58], solvothermal method [59,60], sol–gel method [61,62,63,64,65,66,67,68,69,70], biphasic interfacial reaction [71], spray pyrolysis [72,73,74,75,76,77,78], molten-salt method [79,80,81,82], microwave heating method [83,84], template method [85,86,87,88,89], emulsion-gel process [90], reflux method [91], flux method [92], mechanochemical synthesis [93,94,95], electrospinning method [95,96], solution combustion synthesis [97,98,99,100,101,102,103], sonochemical method [104,105,106,107,108], single step metal organic precursor (SSMO) method [109], template-free hydrothermal process (TFHP) [110], spray drying process [111,112], rheological phase method [113], supercritical fluids [114], and electrospray pyrolysis method [115].

Various types of nanosized LTO materials have been synthesized. They can be classified according to their dimensionality from 0D to 3D architectures (see Table 1): 0D-LTO nanomaterials include nanoparticles [97,103,116,117,118,119], nanocrystals [120,121], nanospheres [39], and hollow spheres [85]. 1D LTO nanomaterials are composed of nanorods [122,123,124,125,126,127], nanowires [128,129,130,131,132], nanofibers [95,133,134,135,136,137,138,139,140], nanotubes [54,141], nanoflakes [142], and nanobelts [143,144,145,146]. 2D LTO nanomaterials encompass nanosheets [136,147,148,149,150,151], nanoplates [152], and wave-like structures [153]. 3D LTO nanomaterials include nanoflowers [153], nanoporous structures [55,154,155,156,157], mesoporous structures [67], hierarchical structures [158], and nanoarrays [159,160,161,162,163,164,165,166,167,168,169]. 

Currently, the growth of LTO nano-powders is achieved by using successive techniques, which can include the synthesis of a precursor at the start and at least one calcination step at the end. For example, Lu et al. [121] prepared nanosized LTO using a three-step process. First, α-Li_2_TiO_3_ was hydrothermally prepared (at 160 °C for 48 h) from 0.5 g anatase TiO_2_ (4.5 nm particles) and added to a 2 mol L^−1^, 40 mL LiOH aqueous solution. Second, cubic NaCl-type (Li_0.4_H_0.6_)_2_TiO_3_ was formed by mixing the white-colored α-Li_2_TiO_3_ with a 1 mol L^−1^ HCl aqueous solution. Finally, the product was calcined at 400 °C for 2 h to obtain nanosized LTO (40 nm in size). Another approach consists of the growth of a precursor with the requested final morphology. For instance, a novel Li_4_Ti_5_O_12_@carbon nanotubes (CNT) composite, composed of Li_4_Ti_5_O_12_ nanowires intertwined with CNT (4 wt%), was synthesized by a simple method. The Li_4_Ti_5_O_12_@CNT shows impressive electrochemical performances, such as high initial coulombic efficiency (ICE, ~94%), acceptable capacity (147.6 mAh g^−1^) and ultra-long life (3000 cycles) [128]. Similarly, Kim et al. [132] prepared LTO nanowires via a two-step ionic exchange process using Na_2_Ti_3_O_7_ nanowires formed by hydrothermal treatment of TiO_2_ powders dispersed in a 10 mol L^−1^ NaOH aqueous solution and subjected to ion exchange in an HCl aqueous solution to produce H_2_Ti_3_O_7_ nanowires. Next, the H_2_Ti_3_O_7_ powders were again hydrothermally treated in a LiOH aqueous solution at 100 °C for 24 h to form lithiated titanates via hydrothermal ionic exchange.

Sorensen et al. [167] fabricated 3D ordered macroporous Li_4_Ti_5_O_12_ samples (3DOM-LTO) synthesized using poly(methyl methacrylate) colloidal crystal templates and metal-organic aqueous precursors via the mixture (5:4 *v*/*v*) of titanyl oxalate solution and lithium acetate. The 3DOM structure, with an interconnected network of nanometer-thick walls, is an attractive architecture for battery materials. The 3DOM architecture has two types of porosity: large macropores, on the order of 100 nm, formed by the spheres, and smaller mesopores, on the order of 15 nm, formed by contact points between the spheres. The surrounding walls of the 3DOM structure are not solid monoliths but consist of crystallites that form an irregular wall surface. Surface roughness can work to the advantage of a battery electrode because it creates more defects for lithium to enter the electrode material.

**Figure 2:**
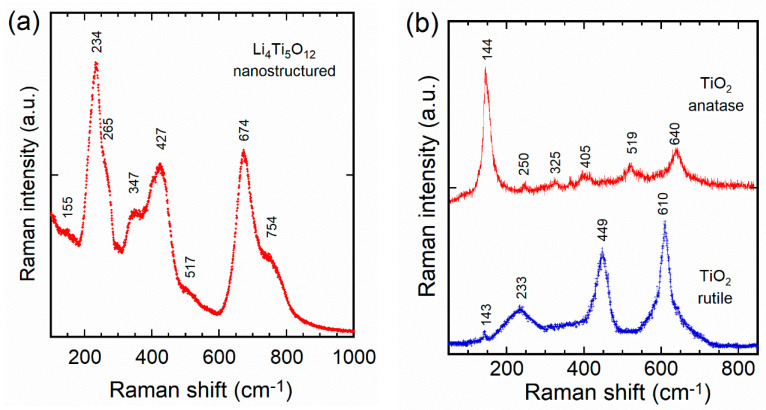
Raman spectra of spinel Li_4_Ti_5_O_12_ nanoparticles (**a**) compared with patterns of rutile and anatase TiO_2_ (**b**).

Raman spectroscopy is a powerful tool for the study of the local bonding configurations in Li_4_Ti_5_O_12_ [170]. Moreover, a low amount (<1%) of other titanates (anatase and rutile TiO_2_) can be easily detected in Raman spectra, which have also been identified as a unique tool to probe structural defects (Figure 2). 

Following the theoretical calculation of A[B_2_]O_4_ spinel-type compound, spinel phase Li_4_Ti_5_O_12_ consists of a symmetric group *O*_h_^7^ with the five expected 3*F*_2g_ + *E*_g_ + *A*_1g_ Raman active vibrations [171,172]. The Raman spectrum of nanocrystalline LTO (Figure 2) displays characteristic bands at 234, 265, 347, 427, 517, 674, and 754 cm^−1^. For a complete understanding, see the analysis of the structural heterogeneity of compacted lithium titanate in Ref. [173]. The Raman band attribution is given in Table 2, together with frequency values reported in the literature [174,175,176,177].

### 2.1. Starting Materials

LTO synthesis techniques have been developed using various titanium and lithium raw materials with the main objective of a low-cost process and good control of the final product morphology. Thus, the choice of precursor for the LTO synthesis is crucial for its application as anode materials for lithium-ion batteries because the particle dispersibility and particle size have peculiar features yielding high Li^+^ diffusion coefficient and better kinetics of Li^+^ ions during charge transfer reactions [178,179]. In the pseudo-binary phase diagram of the Li_2_O-TiO_2_ system, the Li_4_Ti_5_O_12_ region is extremely narrow, making the growth of phase-pure LTO difficult. LTO is usually produced via the formation (or use) of an intermediate Ti-oxide phase converted to the final product through thermal treatment.

#### 2.1.1. Titanium Alkoxides

An alkoxide is the conjugate base of an alcohol and consists of an organic group bonded to a negatively charged oxygen atom (noted RO^−^). Alkoxides of titanium (IV), which are widely employed in organic synthesis of LTO, include titanium ethoxide Ti_4_(OC_2_H_5_)_16_ (noted Ti(OEt)_4_ or TET), titanium butoxide Ti(OC_4_H_9_)_4_ (noted Ti(OBu)_4_ or TBT), titanium isopropoxide Ti(OC_3_H_7_)_4_ (noted Ti(O-i-Pr)_4_ or TIP) and tetraethyl ortho-titanate (Ti(OC_2_H_5_)_4_ (noted TOT). All titanium alkoxides react with water to deposit TiO_2_ in the form of powders or thin films, which can be expressed with the balanced equation:

Ti(OC_3_H_7_)_4_ + 2H_2_O → TiO_2_ + 4OC_3_H_8_. (3)

Generally, the crystallinity and morphology of the TiO_2_ product can be modified by the addition of a weak acid such as acetic acid and by the change in hydrolysis ratio. To provide considerable amounts of unreacted TiO_2_ phase (or other products such as Li_1+x_Ti_2−x_O_4_, Li_2_TiO_3_), inventors developed methods comprising an organo-titanium compound selected from titanic acid esters in an organic solvent. Particularly preferred alkoxides as starting reagents are titanium(IV) isopropoxide and titanium(IV) butoxide (or tetrabutyl titanate). For example, ultrafine LTO nanoparticles can be easily synthesized by hydrothermal and solvothermal methods by reacting TBT or TIP with lithium hydroxide or lithium acetate. However, these processes involve high energy consumption and cost, which prevent them from being used in large-scale productions and practical applications [180,181]. For the fabrication of the LTO/CNT composite, the LTO precursor was prepared by the mixture of 47.5 mmol TBT with 66.5 mmol lithium nitrate dissolved in a mixture of 54 mL ethanol and 4.2 mL (36% in H_2_O) hydrochloric acid, which produces a slightly yellow clear solution [182]. In a typical approach to fabricating LTO-coated TiO_2_ nanotube arrays, the LTO precursor was prepared by mixing three solutions. Stoichiometric amounts of tetrabutyl titanate (Ti(OC_4_H_9_)_4_) (solution A) and citric acid (solution B) were dissolved in alcohol, respectively. In solution B, the citric acid to total metal ions of solution A ratio was 0.8. An alcoholic solution of CH_3_COOLi·2H_2_O was added to a mixed solution of A and B, the Ti/Li = 0.8, and a transparent sol was obtained after stirring for 4 h [183]. Recently, Wang et al. [184] experimented successfully with an energy-saving solid-phase synthesis route for LTO powders using metatitanic acid (H_2_TiO_3_) as a titanium source. Then, 0.7832 g H_2_TiO_3_ powder was mixed with 0.2560 g Li_2_CO_3_ (excessive 4%) in an agate mortar directly. Then, the precursor powder was heated at 500 °C for 2 h and heated to 700 °C with a heating rate of 5 °C min^−1^ for 6 h in Ar atmosphere.

#### 2.1.2. TiO_2_ as Ti Source

With most titanium salts, the starting precursors are easily hydrolyzed to form TiO_2_. Various processes have adopted the use of a TiO_2_ precursor to develop low-cost LTO [39,93,102,115,179,184,185,186,187,188,189,190,191,192,193,194]. Commercial or homemade TiO_2_ (anatase or rutile phase) and tetrabutyl titanate (Ti(C_4_H_9_O)_4_) are the most popular titanium sources used for the preparation of LTO. The use of micro-sized TiO_2_ as a starting material can efficiently reduce the synthesis cost of LTO. Nanosized TiO_2_ (size 20 nm) commercialized as P25 is composed of 85% rutile and 15% anatase phase (price of €212 per kg). Amorphous TiO_2_ has been also employed [193]. The precursor TiO_2_ can be prepared from H_2_TiO_3_ synthesized through the following procedures: (1) industrial titanyl sulfate solution was diluted with de-ionized water to obtain 20 g L^−1^ (TiO_2_) solution; (2) the diluted solution was boiled in a 1000 mL round-bottomed flask attaching to a refluxing condenser with a constant temperature of 105 °C; (3) the solution was then hydrolyzed for 2 h under vigorous stirring; (4) a white precipitate formed gradually and the H_2_TiO_3_ precipitate was washed 2 times with a sulfuric acid aqueous solution of pH 1–2, then washed with de-ionized water several times until no sulfate ion was present (determined by 0.5 mol L^−1^ barium chloride solution), and finally dried in an oven at 80 °C. H_2_TiO_3_ was calcined at 850 °C for 5 h in a tubular furnace to synthesize the precursor TiO_2_. To reduce the synthesis cost and increase the efficiency of LTO, Hong and co-workers [193] prepared TiO_2_ microspheres (1–2 µm in size) assembled with nanoparticles through the urea-based hydrolysis of titanium (IV) oxysulfate (TiOSO_4_), which is generally produced during the sulfate process in titanium mining of ilmenite (FeTiO_3_). Table 3 provides some examples of Ti and Li raw materials used in LTO synthesis techniques.

The conversion of nanocrystalline TiO_2_ (anatase) into LTO was explored by a reaction of colloidal TiO_2_ with LiOH. However, this strategy was not successful, nor its variants employing Li_2_CO_3_, CH_3_COOLi, and LiNO_3_ in combination with the stoichiometric amount of colloidal anatase in acidic or alkaline media at temperatures up to 250 °C (in autoclave). In all cases, the product contained Li_1+x_Ti_2−x_O_4_ with considerable amounts of unreacted anatase [195]. Li et al. [179] used an axiolitic TiO_2_ to prepare LTO nanoparticles. The axiolitic TiO_2_ phase, which was assembled by 10–20 µm particles, was synthesized by solvothermal reaction using the mixture of acetic acid (50 mL) and tetrabutyl titanate (TBT, 2 mL) forming a white suspension. After stirring for 15 min, the suspension was hydrothermally treated at 180 °C for 24 h in a 100 mL Teflon-lined stainless-steel autoclave and collected by centrifugation, washed with ethanol several times, and dried at 60 °C for 24 h. Finally, the precursor of anatase TiO_2_ was obtained after calcination at 500 °C for 4 h. It is rarely reported that nano-sized materials can be synthesized when ordinary TiO_2_ is used as the source of titanium. For example, Han et al. prepared spinel Li_4_Ti_5_O_12_ with the size of ~1 μm using ordinary TiO_2_ and Li_2_CO_3_ through solid-state reaction, which showed poor high-rate capacity [196]. On the contrary, the use of the precursor with special morphology or special treatment makes it possible to obtain as-prepared LTO particles of the same morphology and size as those of the precursors, displaying excellent high-rate capacity [197]. Industrial titanyl sulfate solution is an intermediate product in the commercial preparation of TiO_2_ by sulfate route, which is obtained by acidulating ilmenite (FeTiO_3_) with sulfuric acid that can solubilize titanium to form its sulfate [198].

Numerous groups of researchers have controlled the morphology of the precursor using as-prepared TiO_2_ instead of commercial titanium dioxide [57,107,181,199,200]. Tsai and co-workers [199] fabricated monodispersed sphere-like TiO_2_ precursors (~15 nm) as Ti source for the growth of LTO via a sol–gel method using titanium isopropoxide (TIP) with carboxylic acid addition. In a typical synthesis procedure, acetic acid (3.75 mL, 65 mmol) was injected into the 25 mL anhydride ethanol, followed by the addition of 0.3 mL (1 mmol) TIP. The solution was then heated at 85 °C for 6 h under reflux in air. After that, hydrolysis and condensation were initiated by adding a 7 mL solution consisting of 2 mL DI water and 5 mL ethanol causing the particles to gradually precipitate over 4 h, turning the solution turbid. The residual ethanol and DI water were then pumped out in a vacuum system. TiO_2_ submicron spheres were also prepared with different volumes of butyric (0.3~1.25 mL), valeric (0.5~2 mL), and octanoic (0.5~2 mL) acids, which resulted in larger particle sizes. Jin et al. [107] prepared small and uniformly sized LTO particles (50 to 100 nm) from TiO_2_ powders prepared through the urea-forced hydrolysis/precipitation route below 100 °C, which have extraordinarily large surface areas of more than 250 m^2^ g^−1^. Wang and co-workers [57] used a microemulsion method to prepare ultrafine TiO_2_ precursor for the synthesis of very fine LTO nanoparticles through moderate calcination. TiO_2_ particles were fabricated from the mixture of two solutions: (i) a milky solution obtained by ultrasonic mixing of a certain amount of cetyltrimethyl ammonium bromide (CTAB) in 150 mL deionized water, and (ii) titanium isopropoxide (TIP) and oleic acid with a mole ratio of 1:0.06 dissolved into 60 mL *n*-hexane. The concentration of Ti^4+^ is 0.13 mol L^−1^ and the mole ratio of TIP and CTAB is 1:1.17. The yellowish precipitate was collected by filtrating, washing several times, and drying at 80 °C to produce ultrafine TiO_2_ monodispersed nanoparticles with a particle size of 3~5 nm.

TiO_2_ nanospheres were prepared via the mixture of Ti(OC_4_H_9_)_4_ with (CH_2_OH)_2_ poured into a solution of ethanol and DI water (63:1 in volume) and stirred for 2 h. The obtained white precipitate harvested by centrifugation was washed with ethanol and refluxed for 30 min to form the final product [200].

The microemulsion method was also used successfully by Zhang et al. [201] who synthesized LTO/graphene (G) composites by interfacial electrostatic self-assembly in a water-in-oil (W/O) microemulsion system. In the W/O microemulsion system, the aqueous phase was uniformly dispersed into discontinuous nanoscale “water pools”. The growth of Ti(OH)_4_ was effectively suppressed by the space confinement of the water pools, and the uniform distribution of the water pools made the titanium source hydrolyze uniformly. So, Ti(OH)_4_ colloid was electrostatically self-assembled with the graphene uniformly at the water-oil interface. after adding to this precursor an appropriate amount of LiOH solution, and followed by drying and calcining, LTO particles with a size of less than 50 nm were uniformly anchored on the graphene sheets. This anode demonstrated a capacity of 174 mAh g^−1^ (304.5 mAh cm^−3^) and 152 mAh g^−1^ (266 mAh cm^−3^) at 1C and 10C rate, respectively, and a 97% capacity retention after 600 cycles at 10C rate.

Liao et al. [202] synthesized highly mesoporous anatase phase hierarchical TiO_2_ spheres via a solvothermal method. In a typical synthesis, 0.65 mL TBT was added to 40 mL acetic acid (HAc) with stirring. After a few minutes, the mixture of TBT and HAc was transferred into a 50 mL Teflon-lined stainless-steel autoclave placed in an electronic oven and maintained at 160 °C for 12 h

In many cases, researchers used a few weight-percent excess Li sources to compensate for lithia volatilization during high-temperature heating. Few research works have addressed the issue of nanoporous or nanocrystalline LTO synthesized by soft chemical methods by dipping producing in situ lithium ethoxide that reacts with titanium alkoxide to form a double Li-Ti alkoxide without particle precipitation [67]. Lithium ethoxide (CH_3_CH_2_OLi) was obtained by the gradual exothermic reaction between metallic Li beads in a bath of ethanol cooled at −16 °C with the production of hydrogen according to the relation:

CH_3_CH_2_OH_exc_ +Li → CH_3_CH_2_OLi + ½H_2_. (4)

A similar procedure was carried out by Yu et al. [203], who dissolved 50 mg (7.2 mmol) of metallic lithium in 20 mL benzyl alcohol at 50 °C forming a yellowish solution. Several studies have shown the influence of the synthesis parameters on the structural and electrochemical properties of LTO anode materials [204,205,206,207]. Mahmoud et al. [207] investigated the effect of the gel-drying temperature, annealing time at 900 °C, and calcination temperature on the purity phase, particle size, agglomeration of the particles, and porosity of LTO grown by the sol–gel method. Xu et al. [206] explored the properties of LTO prepared by solid-state reaction with subsequent heat treatment in air and N_2_ atmosphere at 800 °C for 3 h. The calcination carried out in N_2_ ambient results in a larger lattice parameter, which can be attributed to the reduction of some Ti^4+^ ions into Ti^3+^ when the process occurred under low-oxygen partial pressure (darker color of LTO powders). Thus, the presence of Ti^3+^ ions is ascribed to the increase in electron concentration, which results from non-stoichiometry Li_4_Ti_5_O_12−δ_ (Equation (5)):

(5) $$O_{o}^{x}\to \;\frac{1}{2}O_{2}\left(g\right)+V_{o}^{{}^{\circ}{}^{\circ}}+2e^{\prime }.$$

For example, defective mesoporous Li_4_Ti_5_O_12−y_ anode material with improved high-rate performance was prepared by solvothermal method with oxygen vacancies and Ti^3+^–O^2−^–Ti^4+^ pairs formed by annealing the product in N_2_ at 500 °C for 2 h [208]. Defects of the samples were identified by EPR which is sensitive to the electrons and holes trapped at defect sites. The EPR spectrum for N_2_-treated LTO displays one intense signal at *g* = 2.00, which confirms the presence of oxygen vacancies and Ti^3+^. The high-rate performance is represented by a discharge capacity of 139 mAh g^−1^ at a high rate of 20C with a capacity retention of 91.4% over 300 cycles. Similar results have been reported for LTO heat treated under H_2_/Ar atmosphere [16]. The nanostructure of LTO material was found to depend on the preceding intermediate phase formation conditions and the applied annealing protocol.

#### 2.1.3. Intermediate Phase

**Figure 3:**
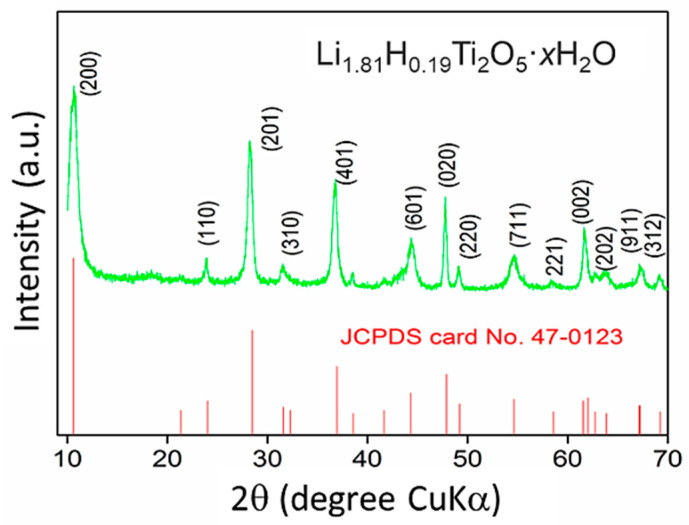
XRD pattern of the intermediate Li_1.81_H_0.19_Ti_2_O_5_·*x*H_2_O (white powder) hydrothermal product. Reproduced with permission from [215]. Copyright 2015 Elsevier.

The process for producing LTO includes the steps of synthesizing a lithium titanate hydrate intermediate via aqueous chemical processing and thermally treating it to produce LTO. Several workers reported the synthesis of LTO nanoparticles via the thermal transformation (annealing) of the intermediate orthorhombic phase, lithium titanate hydrate Li_1.81_H_0.19_Ti_2_O_5_·*x*H_2_O (LTH), obtained by hydrothermal process [209,210,211,212,213]. One advantage of converting the LTH intermediate phase into LTO is its theoretical Li/Ti ratio, 0.905, higher than 0.8 LTO. The over-stoichiometric ratio with uniform distribution in atomic scale can compensate for Li loss during sintering, and provide high homogeneity and purity in the final LTO product. In the patent filed by Demopoulos et al. [211], the LTH intermediate was synthesized as follows: a volume of 20 mL of a 2 mol L^−1^ TiCl_4_ aqueous solution was added dropwise to 180 mL of an ice-cold 1.33 mol L^−1^ LiOH stirred aqueous solution (Li/Ti molar ratio equals 6). The temperature was maintained below 10 °C during addition. The terminal pH of the reaction was within the range of 11.5–12, and the mixture was stirred for an additional 2 h. The as-neutralized precipitate was collected by centrifugation and washed 3 times with deionized (DI) water. The recovered product was transferred to a closed vessel for aging at 80 °C without stirring for 36 h. The LTH intermediate was then further dried in an oven at 80 °C. Also, Chiu and Demopoulos [210] reported the production of nanoflower-structured LTO with “petals” 17–50 nm thick by annealing the LTH (*x* = 2) intermediate phase at 400 °C. Liu et al. [214] reported a hydrothermal treatment of a solution containing titanium isopropoxide as a precursor, LiOH, plus H_2_O_2_, carried out at 130–170 °C to prepare an intermediate that is subsequently transformed to LTO by calcination at 550 °C. Xu et al. [215] analyzed the intermediate LTH phase (white powder) obtained by hydrothermal reaction at 180 °C for 36 h of 1.7 mL (5 mmol) of tetrabutyl titanate, and 0.189 g of LiOH·H_2_O thoroughly mixed in 20 mL of ethanol and 25 mL DI water at room temperature (XRD pattern shown in Figure 3). Calcined at 700 °C for 6 h in air, the white hydrothermal LTH product is transformed into cubic LTO nanosheets (9 nm thick).

Another strategy is to disperse TiO_2_ powders in a concentrated NaOH solution to obtain sodium titanate as an intermediate phase via a hydrothermal reaction. Subsequent treatment in HCl solution for 5 h yields the ion exchange H^+^ for Na^+^ and the formation of H_2_Ti_3_O_7_ (HTO), which is like hydrate titanate possessing a monoclinic phase (*C*2/*m* space group). Further, this intermediate phase is ultrasonically mixed with LiOH and hydrothermally treated at 150 °C for 12 h providing LTO nanowires with a diameter of 50–100 nm and a length of about 10–20 µm [130].

#### 2.1.4. Lithium Salts

For solid-state synthesis of LTO, researchers have employed varying lithium salts, i.e., Li_2_CO_3_, LiOH·H_2_O. Lithium acetate dihydrate (CH_3_COOLi·2H_2_O) noted as LiOAc is used as a precursor solution in many synthesis methods. Lithium nitrate, LiNO_3_, associated with titanyl nitrate (TiO(NO_3_)_2_) as the oxidant precursors and glycine as the fuel are generally used in the solution-combustion method, which provides porous morphology, or nanometer-sized particles [97]. Lithium ethoxide (LiC_2_H_5_O) is used in the sol–gel process with Ti(IV) alkoxides to achieve nanoparticles with high surface areas (>50 m^2^ g^−1^) and small particle sizes (<20 nm). Lithium *tert*-butoxide (LiOC(CH_3_)_3_) is employed as a lithium source in the solvo-thermal method. Lithium acetate starting solution in 2-propanol is associated with titanium isopropoxide (Ti(OC_3_H_7_)_4_) for the growth of LTO through electrospray pyrolysis [115]. Lithium acetylacetonate (LiC_5_H_7_O_2_) is a Li source for the fabrication of electrospun LTO that is soluble in organic solvents as an organometallic compound, in which acetylacetonate anion complexes by bonding each oxygen atom to the metallic cation to form a chelate ring.

LTO prepared by solid-state reaction with two different Li-containing precursors, i.e., Li_2_CO_3_ and LiOAc or different heat treatments, exhibit the same crystal structure as characterized by X-ray diffraction, but show different morphologies, as revealed by scanning electron microscopy (SEM) [216]. SEM images showed that primary LTO/LiOAc particles aggregate to loosely packed small clusters, while LTO/Li_2_CO_3_ particles of submicron size are connected to form a percolated network. The effects of bridged grain boundaries on maintaining the occupancy balance between 8a and 16c sites in LTO structures have an impact on the rate performance and over-potentials of electrodes.

### 2.2. Solid State Reaction

**Figure 4:**
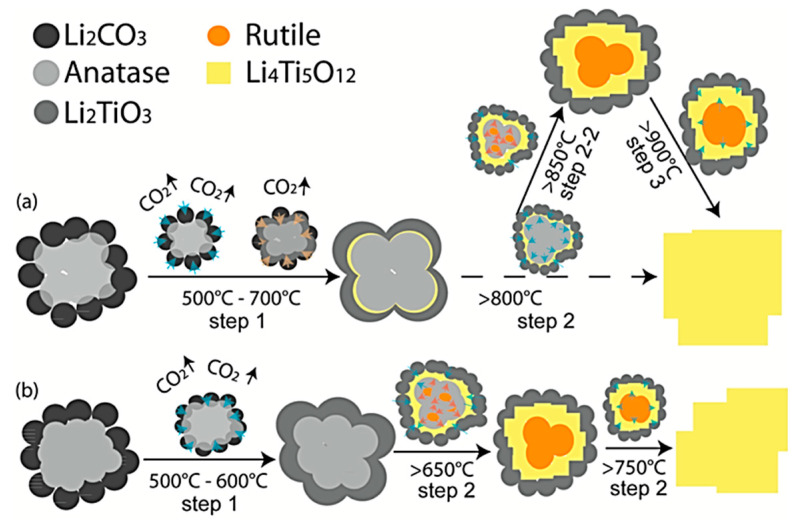
Schematic representation of the formation process of Li_4_Ti_5_O_12_ with different anatase crystallite sizes. When the temperature increases above 500 °C, Li_2_COO_3_ starts to react with the anatase at the particle interface forming a Li-rich Li_2_TiO_3_ phase. This process occurs at a steady rate until all the Li_2_COO_3_ articles have completely reacted at about 650 °C. The reaction conditions are based on results from anatase with crystallite sizes of ~50 nm (**a**) and ~20 nm (**b**). Reproduced from [220] with permission. Copyright 2014 American Chemical Society.

In large-scale industrial synthesis of active material for lithium-ion batteries, solid-state reaction (SSR) is considered one of the most simple and practicable techniques. However, this method has a distinct drawback in that the final particle size of the prepared electrode material increases greatly after high-temperature calcination. It is very difficult to obtain a product with a narrow size distribution. Two types of preparation are utilized: (i) one-step SSR [44,179,217,218] and (ii) two-step synthesis with SSR as the final process [219]. In this process, various methods were used to assist the SSR synthesis, not only for reducing the particle size of reactants but also the increasing the homogeneity of the starting mixture. The formation mechanism of LTO was investigated by [220]. Combined in situ high-temperature powder X-ray diffraction (HT-PXRD) and thermal gravimetry-differential thermal analysis (TG-DTA) showed the origin of the impurity phases and revealed that the formation of LTO from anatase TiO_2_ and Li_2_CO_3_ is a two-stage process. Initially, TiO_2_ and Li_2_CO_3_ react to form monoclinic Li_2_TiO_3_, followed at higher temperatures by a reaction with the remaining TiO_2_ to yield Li_4_Ti_5_O_12_. This is different from the mechanism suggested by Shen et al. [220], arguing that crystalline Li_2_CO_3_ changes to an amorphous Li-precursor upon heating and then Li diffuses into TiO_2_ to form Li_2_TiO_3_. It is also demonstrated that Li_4_Ti_5_O_12_ decomposes to some Ti-rich phases and probably Li_2_O when heated above 1000 °C. A schematic representation of the formation process of Li_4_Ti_5_O_12_ with different anatase crystallite sizes is presented in Figure 4.

#### 2.2.1. One-Step SSR

From a commercial viewpoint, the synthesis of LTO powders via SSR exhibits a potential application due to the simple synthesis route and low synthesis cost. The LTO crystalline phase can be prepared by the one-step SSR method using stoichiometric quantities of starting materials ground together to a fine uniform powder and pressed into pellets fired at high temperatures (>800 °C). Various starting materials are employed [4,7,221]. In method A, the mixture of lithium carbonate and titanium metal is fired at 900 °C, in air, for 20 h but the rutile form of TiO_2_ is present as an impurity of a few percent:

2Li_2_CO_3_ + 5Ti +4O_2_ → Li_4_Ti_5_O_12_ + 2CO↑. (6)

In method B, lithium hydroxide and titanium dioxide powders are the starting materials. The reaction is carried out at 800 °C in an inert atmosphere:

4LiOH + 5TiO_2_ → Li_4_Ti_5_O_12_ + 2H_2_O. (7)

In method C, LTO is synthesized from a mixture of TiO_2_ and Li_2_TiO_3_ intimately ground in an agate mortar and then heated in a platinum crucible in air at temperatures between 800 and 1000 °C:

2Li_2_TiO_3_ + 3TiO_2_ → Li_4_Ti_5_O_12_. (8)

**Figure 5:**
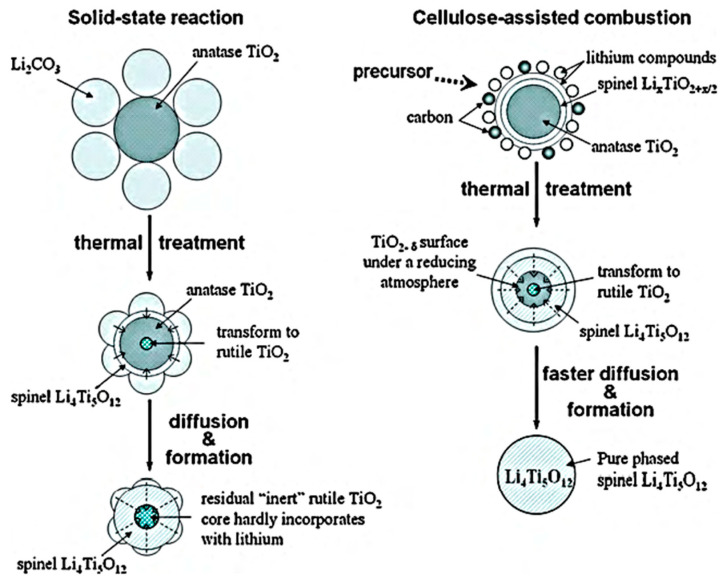
Schematic comparison of the synthesis mechanism for the solid-state reaction (**left**) and cellulose-assisted glycine-nitrate combustion (**right**). The solid-phase reaction between TiO_2_ and lithium compounds to form Li–Ti–O spinel and the phase transition of TiO_2_ from anatase to “inert” rutile phase occur competitively during both synthesis processes. Reproduced with permission from [217]. Copyright 2010 Elsevier.

The solid-phase reaction involves cation and anion diffusion. Commonly, the precursors are anatase TiO_2_ and Li_2_CO_3_ (method D). TiO_2_ and Li_2_CO_3_ have melting points of 1640 and 723 °C, respectively. The overall reaction, providing impurity-free LTO, can be expressed by Equation (9):

2Li_2_CO_3_ + 5TiO_2_ → Li_4_Ti_5_O_12_ + 2CO_2_, (9)

where 16.1% of the weight loss is expected due to CO_2_ evolution. The diffusivity of Li^+^ ions being much higher than that of Ti^4+^ suggests that Li_2_CO_3_ is much more active than TiO_2_ at intermediate temperature ranges. As a result, the formation of LTO is attributed to Li_2_CO_3_ reacting with stable TiO_2_ (see Figure 5) [217]. Duh et al. [187,222] prepared LTO powders by a one-step solid-state reaction using LiCl, H_2_C_2_O_4_, and TiCl_4_, as the raw materials. First, an appropriate amount of LiCl and 70 wt.% of oxalic acid was mixed together, and then, TiCl_4_ was added. Mixing of TiCl_4_ and oxalic acid resulted in the metathesis reaction and an acid mist of HCl was emitted during the process. The precursor was heated at 150 °C for 0.5 h. Next, the precursor was sintered at 400 °C for 3 h in air providing micron-sized particles.

Sorensen et al. [167] prepared titanyl oxalate H_2_TiO(C_2_O_4_)_2_ in solution as Ti precursor, combining Ti(OH)_4_ precipitate (formed by 49.6 mL of 2.017 mol L^−1^ TiOCl_2_ with 27 mL of aqueous NH_4_OH) with 0.2 mol of oxalic acid. A mixture (5:4 *v*/*v*) of titanyl oxalate solution and lithium acetate was used as a precursor solution for the fabrication of a three-dimensionally ordered microporous LTO material. Wang et al. [223] synthesized a graphitic carbon-coated lithium titanium (LTO/GC) sample by a simple one-step solid-state reaction process with the assistance of sucrose without elevating the sintering temperature. The average grain size of the as-prepared LTO/GC was about 100–200 nm, one order smaller than that of pure LTO prepared similarly. After 300 cycles, the capacity retention was more than 90% at a high rate of 15C.

Several workers stressed the importance of the process of mixing raw materials. Generally, the starting Ti and Li source materials are ground with enough liquid (methanol, ethyl alcohol, acetone) to form a slurry, which is further dried and heat treated to obtain a pure LTO ceramic [43,198]. Growth of LTO powders via SSR using Li_2_CO_3_ and anatase TiO_2_ as starting reagents was reported by Shenouda and Murali [42], who first mixed and grounded the precursor materials in a mortar with some drops of acetone. The powder mixture of samples was dried in an oven at 150 °C for 1/2 h and finally fired at 900 °C for 10 h in air. The resulting particles were crystallized with a spherical shape but SEM images showed a wide particle size distribution (average grain size of ~1.5 µm). Yi et al. [224] used the same procedure but the calcination at 850 °C for 24 h in a flowing air atmosphere yields particle size in the range 400–600 nm. Yang and Gao [225] synthesized LTO powders via a simple solid-state reaction using TiO_2_-anatase and Li_2_CO_3_ (mole ratio of Li:Ti = 4.2:5) as reaction precursors, mixed and heated at 850 °C for 16 h to obtain well-crystallized LTO grains with average size of 480 nm. Kim and Cho [226] fabricated a large quantity of spinel Li_4_Ti_5_O_12_ nanowires (diameter of 150 nm) by firing a mixture of TiO_2_·1.25H_2_O nanowires and Li acetates at 800 °C for 3 h. The Brunauer–Emmett–Teller (BET) specific surface area (SSA) of the spinel wire was 38 m^2^ g^−1^, which was seven times higher than that of fine particles, which had a SSA of only 4.6 m^2^ g^−1^. Wu and co-workers [198] synthesized two kinds of LTO samples via SSR using H_2_TiO_3_ and TiO_2_ as the Ti precursors and Li_2_CO_3_. The molar ratio of Li and Ti was 0.84. The process steps were: (1) being initially mixed by grinding in the ethanol for 2 h at room temperature, (2) being dried in the oven at 80 °C for 6 h, and (3) thermal treatment at 850 °C for 16 h in a tubular furnace. Spherical particles with average sizes of about 1.0 and 0.5 µm were grown using the precursor H_2_TiO_3_ and TiO_2_, respectively. The size of TiO_2_ particles is smaller than that of H_2_TiO_3_ particles, which is attributed to the dehydration in the calcination of H_2_TiO_3_. Han et al. [227] fabricated 15 kg of LTO to test a mass production line. Here, 4.88 kg coarse Li_2_CO_3_ (*D*_m_ = 4.50 µm, purity > 99.5%) and anatase-phase TiO_2_ (*D*_m_ = 230 nm, purity > 99.0%) were used as the starting materials, corresponding to a Li/Ti ratio of 4.05/5.00. The mixture was added to 29.91 kg of de-ionized water containing 2 wt.% of the ammonium salt of polycarboxylic acid dispersant. The slurries underwent 6 h of high-energy milling at 1500 rpm using 0.30 mm ZrO_2_ beads. Then, heat-treated at 800 °C for 3 h in air at a heating rate of 3 °C min^−1^. The mean particle size was 242 nm. Yao and co-workers [228] synthesized LTO whiskers (~300 nm in diameter) via a solid-state process using TiO_2_-B and lithium acetate dihydrate as Ti and Li precursors, respectively. TiO_2_-B was obtained by calcination in a muffle oven at 500 °C in the air for 2 h of an intermediate product, which is the result of the ion-exchange process of hydrated K_2_Ti_2_O_5_ in 0.1 mol L^−1^ HCl solution. Several researchers investigated the influence of the TiO_2_ particle size on the properties of Li_4_Ti_5_O_12_ anode [229,230]. Chen et al. [229] proposed a simple approach to synthesize nanostructured LTO with different morphologies (nanorods, hollow spheres, and nanoparticles), in which the TiO_2_ precursor was first coated with a conductive carbon layer by the chemical vapor deposition (CVD) method, followed by a solid-state reaction with lithium salt. The results indicate that, by employing the carbon pre-coating process, the carbon-coated nanostructured LTO can maintain the initial morphologies of the TiO_2_ precursors. Table 4 lists the different synthesis conditions for the preparation of LTO anode materials via solid-state reaction.

#### 2.2.2. Two-Step SSR

**Figure 6:**
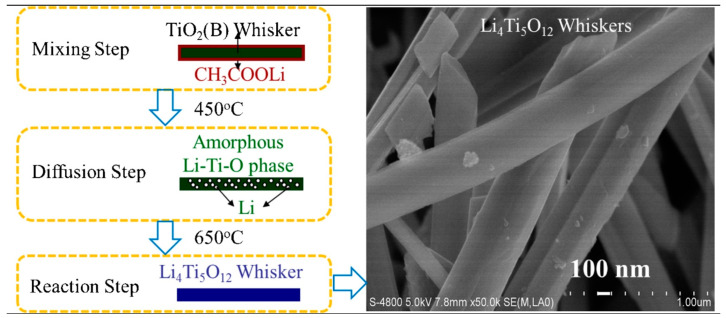
Typical solid-state reaction for the synthesis of LTO whiskers using TiO_2_-B and CH_3_COOLi·2H_2_O (ratio fixed at 1:1.05). The two steps (diffusion and reaction) lead to a low solid-state reaction temperature of 650 °C and maintain the morphologies of whiskers. Reproduced from [228]. Copyright 2016 Elsevier.

The mechano-chemical activation process, i.e., ball milling, has been shown to assist SSR synthesis. It allows the intimate mixing and induces the phase reaction of the reactants at room temperature, improving the structural characteristics of LTO particles [46,48,92,238,239,240,241,242,243,244]. Thus, a high compositional homogeneity can be obtained at lower calcination temperatures. Huang et al. [242] showed that, despite first planet ball milling commercial LiCO_3_ and TiO_2_ starting materials for 4 h with alcohol as a solvent, the LTO grown via solid-state reaction has a particle size of 0.9 µm. LTO samples were prepared via an SSR process using TiO_2_ and Li_2_CO_3_, successively, together mixed by ball milling using ethanol as a dispersant agent for 5 h, dried at 80 °C, calcined at 800 °C for 12 h, and then ground into fine particles by hand. The displayed particle size was less than 1 µm. Most LTO particles have a spindle shape, like two particles interconnecting together [243]. Becker and co-workers [244] homogenized the mixture of Li_2_CO_3_ and anatase TiO_2_ using a wet ball-milling route with *n*-pentane at 400 rpm for 1 h. Then, the final LTO white powders were obtained by heat-treatment of the dried mixture in a platinum crucible at 900 °C for 8 h in air. In another experiment, 200 µL of 2-propanol was added as a dispersing agent. Yao et al. [228] synthesized LTO whiskers (300 nm in diameter) from TiO_2_-B whiskers via a solid-state reaction at 650 °C. The crystal structure of the TiO_2_-B is easy for lithium diffusion and the process is performed in two separate steps (i.e., diffusion and reaction), which makes it possible to decrease the solid-state reaction temperature down to 650 °C and then maintain the morphologies of whiskers (Figure 6).

A comparison of the electrochemical performance of nanostructured LTO materials synthesized by solid-state reaction with different morphologies is given in Table 5. Lai et al. employed the initial ball milling step to obtain titanate. In a typical experiment, 4.02 g TiOSO_4_·*x*H_2_O and 2.765 g LiOH·H_2_O were added to the agate tank and then well mixed by ball milling in a planetary ball mill under air atmosphere at a speed of 300 rpm for 4 h [46]. The effects of Li_2_CO_3_ particle size (*d*_50_ in the range 0.4–4.5 µm) on the properties of LTO were investigated using a ball-milling-assisted solid-state reaction [240]. Then, 31.08 g of Li_2_CO_3_ and 80 g of TiO_2_ (Li:Ti = 0.84) were mechanically dispersed in 200 mL DI water and ground by a conventional ball milling for 1 h at a rotor speed of 500 rpm. The powders were sintered at 800 °C in air for 12 h. Electrochemical tests showed that LTO samples synthesized by fine Li_2_CO_3_ particles exhibit better rate capacity and cycle performance. Huang et al. [245] fabricated LTO using a ball-milling-modified solid-state reaction. The precursor of TiO_2_·*x*H_2_O (the hydrolysis product of TBT) coated Li_2_CoO_3_ was formed from a TBT, Li_2_CoO_3_, HNO_3_ solution dispersed in an agate jar and well mixed by ball milling performed in air at 400 rpm rotational speed for 10 h. Finally, porous LTO was obtained after calcination at 800 °C for 7 h in air. Natalia et al. [246] prepared an LTO precursor by mixing anatase TiO_2_ and Li_2_CO_3_ at a molar ratio of 5:2 with zirconia balls as crushers. The mixing process was performed in a bill mill at 300 rpm for 2 h and then continued at 480 rpm for 2 h. LTO@CN composite was synthesized via a solid-state process, and the detailed process was described as follows. First, 2.2281 g of Li_2_CO_3_, 5.9985 g of TiO_2_, and 1 g of folic acid were mixed by ball milling in ethyl alcohol for 6 h. Second, the solvent was evaporated by drying the readily prepared mixture at 80 °C for 2 h. Third, the synthesized precursor was heated under an argon atmosphere at 750 °C for 8 h [247]. An alternative way to improve the crystallinity of LTO is the addition of a small amount of salt during synthesis, which is referred to as a salt-assisted solid-state reaction [233]. In this work, TiO_2_, LiOH, NaCl (2–6 wt.%), and methanol (acting as a dispersant) were mixed in a ball mill for 4 h forming a slurry sintered at 800 °C for 12 h in an O_2_ atmosphere.

Li et al. [179] studied the effect of precursor size on the morphology of nanosized LTO synthesized by ball-milling/SSR process using axiolitic TiO_2_ (a-TiO_2_) as Ti source instead of commercial TiO_2_. The detailed process using urea as a complexing agent is as follows: a-TiO_2_, Li_2_CO_3_, CO(NH_2_)_2_ (molar ratio Ti:Li:urea = 5:4:1), and 10 mL titanium isopropoxide were added into a grinding bowl for 5 h forming a white slurry. After drying at 80 °C in air for 24 h, the obtained precursor was ground fully and calcined at 850 °C in N_2_ for 12 h. These LTO powders (average particle size of 220 nm) showed excellent capacity of 120 mAh g^−1^ at 10C even after 20 cycles. Using small-size TiO_2_ as the precursor significantly decreases the particle size of LTO, which is beneficial for the fast kinetics of Li ions. The solid-state calcination route was also widely used to prepare LTO/C composite. For instance, Hu and co-workers [219] prepared LTO/C composites through a one-step solid-state reaction method using four commonly used organic compounds or organic polymers as carbon sources, i.e., polyacrylate acid (PAA), citric acid (CA), maleic acid (MA), and polyvinyl alcohol (PVA). LiOH·H_2_O was dissolved into an aqueous solution of PAA with different molar ratios, and powdered TiO_2_ was added into the PAA-Li solution and stirred for 6 h to yield homogeneous PAA-Li+TiO_2_ slurries. LTO/C composite with a small particle size of ~300 nm displayed an initial discharge capacity of 161 mAh g^−1^ and a capacity retention of 95.9% after 50 cycles. Krajewsli et al. [248] performed a three-step solid-state synthesis to produce LTO powders. In the first stage, stoichiometric amounts of Li_2_CO_3_ and TiO_2_ starting reagents were together mixed, ground in an agate mortar, and heated in an alumina crucible to 950 °C and for 10 h in air. In the second step, the obtained powder was ground and heated at 500 °C for 6 h and then at 800 °C for the additional 20 h in air. Impurities such as Li_2_TiO_3_ and rutile TiO_2_ were detected. In the third stage, the sample was mixed with ethanol medium and mechanically ground for 12 h in a planetary ball mill at a rotation speed of 200 rpm. The mixed reactant was evaporated and subsequently dried at 150 °C for a few hours in air. Finally, the powder was ground and heated at 500 °C for 6 h and then at 800 °C for an additional 20 h under air. After the first and second steps, the powders consisted of large crystallites with well-developed surface faceting ~2−3 µm in size, while particles were much smaller (~200−500 nm) after the third ball-milling process.

#### 2.2.3. SSR-Assisted Method

The rapid microwave-assisted solid-state process is a fast and economical synthetic method that yields uniform Li_4_Ti_5_O_12_ nanocrystallites prepared in large quantities. The microwave system can significantly shorten the synthesis time to minutes [251,252]. Compared to the existing solid-state synthetic methods using conventional heating, microwave irradiation prompts a much more rapid solid-state reaction due to the interaction of the microwave field with reactant molecules. In particular, the reaction time and temperature of consecutive steps can be finely programmed in modern microwave systems, facilitating easy and efficient optimization of reaction conditions and scale-up material preparation. In a typical experiment, TiO_2_ as raw material has an appropriate dielectric loss tangent (tan *δ* ≈ 1 × 10^−4^) in the microwave field, which is preferable for energy absorption during microwave-induced synthesis. The starting materials of TiO_2_ and Li_2_CO_3_ were mixed and ball milled in acetone for 10 h and, then, treated in a microwave system (frequency of 2.45 GHz and power between 200 and 1400 W) at 600−900 °C for 30 min. LTO produced at 800 °C has a distinctively narrower particle size distribution without agglomeration, and the particle size ranges from 100 to 350 nm with an average size of 180 nm.

#### 2.2.4. Sintering Process

**Figure 7:**
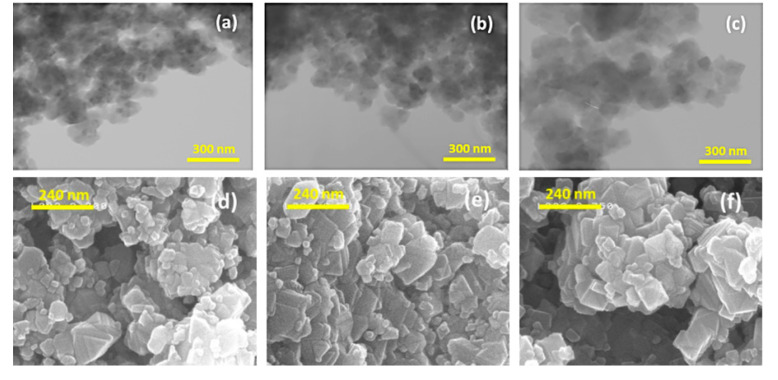

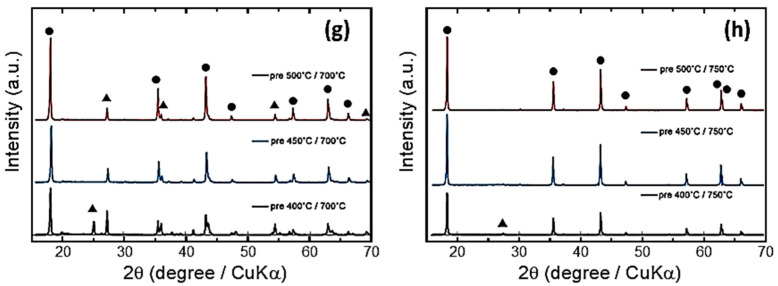
LTO nanoparticles prepared by a two-step sintering. (**a**–**c**) TEM images of the samples for the pre-sintering temperature of 400, 450, and 500 °C, respectively. (**d**–**f**) SEM images of the samples sintered at 750 °C with the pre-sintered temperature of 400, 450, and 500 °C, respectively. XRD patterns of the samples sintered at 700 °C (**g**) and at 750 °C (**h**) with the pre-sintered temperature of 400 °C (black line), 450 °C (blue line), and 500 °C (red line), which show ● Li_4_Ti_5_O_12_ and ▲ Li_2_TiO_3_. Reproduced from [249]. Copyright the terms of the Creative Commons Attribution 4.0 International License.

Several researchers have investigated the effect of sintering, which is a mandatory step to obtain a well-crystallized final product [205,253]. Ohtake investigated the crystallinity of LTO nanoparticles prepared by a two-step sintering through solid-phase synthesis from lithium acetate and anatase TiO_2_ starting chemicals Mixing of reagents via ball milling at a rotating speed of 320 rpm for 1 h was followed by a pre-sintering at 400, 450, and 500 °C for 1 h in air, and a precursor was formed [253]. Furthermore, the precursor was mixed by the ball milling for 1 h similarly and was sintered at 700 and 750 °C at 10 °C min^−1^ rate for 1 h in air. The results showed that the pure phase LTO (fine particles of 110 nm) was obtained under sintering at 750 °C subsequently to a pre-sintering temperature of 500 °C via Li_2_TiO_3_ (Figure 7). Yuan et al. [204] evaluated the optimal calcination atmospheres for the synthesis of LTO (air, N_2_, H_2_/Ar). The oxygen vacancy created due to the reduction of TiO_2_ in the reducing atmosphere of hydrogen helps to enhance the oxygen ion diffusivity during calcination, whereas carbon coated over TiO_2_ in the starting material acts as a reducing agent during calcination in the nitrogen atmosphere. Thus, the LTO samples prepared by heating under diluted hydrogen (H_2_/Ar) and nitrogen atmosphere showed a remarkably higher rate capability and better cycle stability compared to samples prepared by heating in air.

### 2.3. Sol–Gel Process

Among the wet-chemical techniques, the sol–gel route has gradually attracted the attention of the electrochemistry community as a versatile way to prepare complex intercalation electrodes. LTO has been synthesized through a variety of sol–gel techniques, including various experimental conditions producing a homogeneous distribution of uniform, submicron-size particles with good stoichiometric control [62,63,188,254,255,256,257,258,259,260,261,262,263,264]. This process yields small particle size, homogeneous and narrow size distribution, low temperature, and short periods of calcination. It is based on the hydrolysis and condensation of molecular precursors [265]. In the sol–gel process, a solid phase is formed through the gelation of a colloidal (gel) suspension. Drying of the gel can then give a “dry gel” (xerogel) state and subsequent heat treatment can be used to remove unreacted organic residues, stabilize the gel, densify it, or introduce crystallinity [266]. This method involves the mixing of metal acetates with a complexing agent (chelate) in an aqueous medium. The complexing agent acts as a fuel during the formation process of transition-metal oxide powders, decomposes the homogeneous precipitate of metal complexes at low temperatures, and yields an impurity-free compound. Thus, the control of the growth of LTO crystal is ensured by the carboxylic acid groups present in the complexing agent, which could form chemical bonds with the metal ions to produce a viscous resin (precursor) upon evaporation of the solvent. Chelating agents such as acetic acid (AA) [62,267], citric acid (CA) [263,268,269,270,271,272,273], oxalic acid (OA) [63,187,274], ethylene diamine tetra-acetic acid (EDTA) [263,275], and triethanolamine (TEA) [64,65,276] are often used to reduce the excessive reactivity of metal alkoxides towards water. Zhou et al. [277] demonstrated that EDTA–CA–metal complexing reaction is a non-ion selective process.

**Figure 8:**
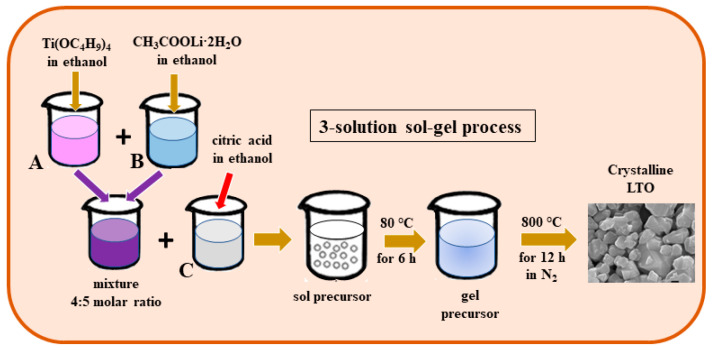
Typical 3-solution sol–gel method (A,B,C baths) for the preparation of LTO nanoparticles using tetrabutyl titanate and lithium acetate as Ti and Li raw materials and citric acid as complexing agent.

Several workers choose the homopolymer polyvinylpyrrolidone (PVP, (C_6_H_9_NO)_n_) as a chelating agent [278,279,280]. When PVP is added to the metal colloidal dispersion, its non-ionic surfactant property makes PVP readily dissolve in water and the imide group (N and O atoms) establishes a strong affinity to a single unit of metal colloid. This imide and metal bonding stabilizes the particle growth and increases the probability of nucleus formation [281]. Further, the sintering of this complex melts PVP and decomposes. Figure 8 shows the typical 3-solution sol–gel preparation of LTO using tetrabutyl titanate and lithium acetate as Ti and Li raw materials and citric acid as a complexing agent.

In 1998, Bach et al. [254] synthesized spinel LTO via a sol–gel process in non-aqueous media (ethanol). They developed the hydrolysis and condensation of metal alkoxides, Ti(OR)_4_, which are the result of a direct combination of a metal *M* with an alcohol ROH. As an example, titanium isopropoxide, Ti(OCH(CH_3_)_2_)_4_ is hydrolyzed according to Equation (10):

Ti(OCH(CH_3_)_2_)_4_ + *x*H_2_O → Ti(OH)_x_[OCH(CH_3_)_2_]_4−x_ + (CH_3_)_2_CHOH, (10)

and a polymerization-condensation reaction occurs by dehydration. The mixture of 0.082 mol titanium isopropoxide with lithium acetate in 75 mL of ethanol solution results in a yellow solution, which becomes more and more viscous, yielding the formation of a white monolithic gel after 1 h. Mani and co-workers [69] explained the nanostructure formation of LTO by a dissolution–crystallization–self-assembly mechanism by controlling the annealing temperature of the gel obtained from titanium isopropoxide sol. Supersaturated anatase (Ti, Li)O_2_ is formed at 400 °C, whereas anatase and/or rutile interspersed with Li_2_TiO_3_ and Li_4_Ti_5_O_12_ are observed between 450 °C and 800 °C. Most sol–gel syntheses are conducted using lithium acetate (CH_3_COOLi) or lithium hydroxide (LiOH∙H_2_O) and tetrabutyl titanate (Ti(OC_4_H_9_)_4_) as starting materials, using the molar ratio Li:Ti = 4:5 to form the sol. After the sol is dried at 120 °C for 10 h, a gel is formed and then calcined in N_2_ atmosphere at 800 °C for 12 h to obtain the final LTO powders [232,261]. Instead of a pure aqueous solution, Luo et al. [261] employed a solution of 17 mL ethanol and 1 mL DI water, in which 3.46 g of glacial acetic acid was added to slow down the hydrolysis reaction of tetrabutyl titanate. Ma and Cheng [282] investigated by single factor method the effects of chelating agent (triethanolamine), raw material ratio, calcination temperature and calcination time on LTO products with pure phase, uniform grain size, 100–200 nm particle size and no obvious agglomeration. Xia and co-workers [283] used a solution of alcohol and propionic acid (alcohol: propionic acid = 2:1), the propionic acid is as a hydrolysis inhibitor in the solution to suppress the tetrabutyl titanate decomposition. Qiu and co-workers [284] synthesized pure LTO particles using a modified sol–gel method with ethyl acetoacetate as a chelating agent and extended this technique for the preparation of La-doped LTO, which can restrain the agglomeration of particles during heat treatment. Mahmoud et al. [191] prepared LTO nanoparticles (360 nm in size) by using the sol–gel procedure with the stoichiometric amounts of TiCl_4_ diluted in ethanol, LiOH·H_2_O as reagents, and TEA as a complexing agent with a Ti/TEA molar ratio of 0.8. The best electrochemical performances were obtained for the sample calcined at 900 °C for 1 h using the heating/cooling rate of 2 °C min^−1^. Impurity-free nanosized LTO powders were synthesized by the sol–gel process using TIP and LiOAc (LI/Ti = 4.5) mixed in 75 mL of ethanol forming a yellow-colored solution, which turned to white color after stirring for 10 min [255]. The LTO nanoparticles obtained by grounding to fine powder and calcination at 800 °C for 12 h exhibited a specific discharge capacity of 242 mAh g^−1^ at 0.1C rate in the potential range of 0.01–1.75 V vs. Li^+^/Li. Examples of starting materials used in wet-chemical methods for the synthesis of LTO anodes are given in Table 6.

To mitigate the formation of agglomerates, surfactants may be used during sol–gel synthesis. A surfactant acts as a template for nanoparticle formation. The adsorption of surfactant-like molecules to nucleated nano-crystals lowers the free energy of the surface and, therefore, the reactivity of the particles. The ratio of surfactant to metal precursor can control the size distribution of the nanoparticles [289,290]. Several researchers employed ionic surfactants to control the crystal growth of LTO such as CTAB, sodium dodecyl benzene sulfonate (SDBS), or triblock copolymer PEO)_20_(PPO)_70_(PEO)_20_ (Pluronic, P123) [288,291,292,293,294]. In a typical process, CTAB is dissolved in 100 mL ethanol under magnetic stirring. Four grams of lithium acetate dihydrate was dissolved in the above solution with continued magnetic stirring. Titanium (IV) isopropoxide is added to the above solution dropwise by keeping the mole ratio Li:Ti = 1:1.25. The temperature of the solution is raised to 90 °C and stirred continuously to form the gel. The gel is aged at 100 °C for 24 h in air and the precursor is decomposed at 400 °C for 4 h followed by calcination at 800 °C for 12 h in air. The average discharge capacity of the prepared LTO taken over 20 cycles is ~60 mAh g^−1^ at a constant current density of 21.37 mA g^−1^ [292]. Jiang et al. [70] have prepared highly dispersed LTO nanoparticles by the triblock copolymer P123 surfactant-assisted sol–gel process, exhibiting very good rate capability. By using the P123 surfactant, the LTO sample is very loose, with the individual grains being well dispersed and almost without any aggregation. Zhang et al. [288] synthesized mesoporous LTO nanoparticles (~100 nm) via the sol–gel process by employing a nonionic P123 surfactant as the structure-directing agent. In a typical procedure, the Ti-based sol contains 3.0 g P123 and 6 mL HNO_3_ (65 wt.%) dissolved in 30 mL anhydrous ethanol, subsequently added by 8.8 mL titanate isopropoxide. Wang and co-workers [287] synthesized LTO nanocrystals with an ultra-fine particle size distribution between 120 and 250 nm through the sol–gel method using lauric acid as a surfactant. A white sticky gel was obtained by mixing three alcoholic solutions containing CH_3_COOLi∙2H_2_O, Ti(OC_4_H_9_)_4_, and lauric acid, respectively. Li et al. [293] synthesized hierarchical Li_4_Ti_5_O_12_-TiO_2_ microspheres using an SDBS/CTAB mixed surfactant. When the critical micelle concentration is reached, the self-assembly of anionic and cationic surfactants is observed and intermediates [Ti–O–Ti]^−^_n_ transforms gradually into spherical mixed micelles.

**Figure 9:**
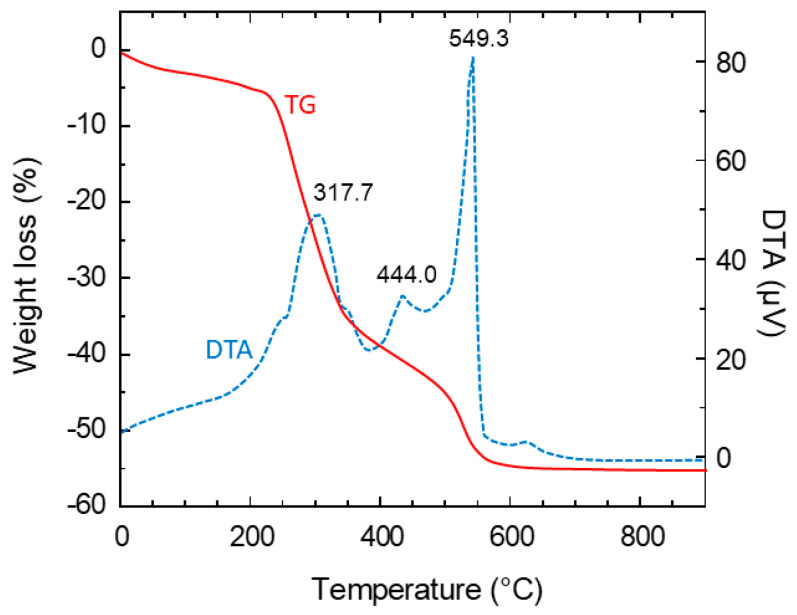
TG-DTA curves of the LTO precursors prepared by triethanolamine as complexing agent. Three distinct steps of weight loss are observed. The exothermic peak at 317.7 °C is assigned to the combustion of the xerogel precursor and release of CO_2_ and H_2_O gases. The second (444.0 °C) and third (549.3 °C) exothermic peaks are due to the continued combustion of the residue carbonate components and the decomposition of the acetate. LTO crystallization is initiated at ~600 °C (particle size of ~80 nm). Reproduced with permission from [65]. Copyright 2007 Elsevier.

In 2002, Kavan and Grätzel [285] reported the preparation of nanocrystalline LTO particles in a slurry using lithium ethoxide and Ti(IV) alkoxides. The incorporation of polyethylene glycol (PEG) produced a viscous liquid that was deposited using a doctor-blading technique. The thickness is about 2–4 μm and the electrochemical performance is excellent, at least at the beginning of the charging/discharging cycling. The synthesis of double metal alkoxides containing lithium can be produced through a condensation reaction with the elimination of a volatile side product. The simplest case is the reaction of metallic lithium with an alkoxide in a solution of either pure alcohol or alcohol and some organic solvent. This reaction results in the formation of a double metal alkoxide and the evolution of hydrogen gas [295]. Shen et al. [62] reported the first synthesis of Li_4_Ti_5_O_12_ nanocrystalline via the sol–gel method to obtain nano-scale particles (100 nm) using tetrabutyl titanate (Ti(OC_4_H_9_)_4_) and isopropyl alcohol mixed with a mole ratio of 1:60, and lithium acetate dissolved into the mixture solutions of isopropyl alcohol/deionize water/acetic acid. Kuo and Lin [270] fabricated LTO/C composites via a modified one-pot sol–gel synthesis using citric acid as a chelating agent and carbon source. The gel precursor was obtained by mixing two solutions A and B prepared by dissolving CH_3_COOLi·H_2_O and Ti(OC_4_H_9_)_4_ in ethanol solution (99 wt.%) with Li:Ti molar ratio of 4:5 with a third ethanol solution containing citric acid. The resultant mixture (ratio of 4:5:1.25) was heated at 80 °C for 6 h and then calcinated by a two-step treatment at 350 °C for 4 h followed by heating at 800 °C for 12 h in an N_2_ atmosphere. The final product shows uniform particle shape and size distribution (size of ca. 100–300 nm). Rho and Kanamura used two kinds of sols for the synthesis of LTO films prepared by spin coating: (i) 7.65 mL of titanium isopropoxide into 200 mL ethanol/2-propanol with poly(vinylpyrrolidone) (PVP) as solvent and (ii) 0.6 g LiOH dissolved in DI water. The sols mixed for 1 h convert to gel during the spin coating process at 3000 rpm [278]. Hao et al. [65] studied the influence of various complex agents on the electrochemical properties of Li_4_Ti_5_O_12_ anode material. This method involves the mixing of two solutions, which form a transparent gel after slow hydrolysis for several hours: (i) lithium acetate dissolved in the mixture solutions of ethanol and DI water, and (ii) tetrabutyl titanate dissolved in alcohol and mixed with the complexing agent. The compound synthesized by TEA (see Figure 9 showing the thermogravimetry TG-DTA analysis) had the smallest average particle size of 80 nm with homogeneous distribution and, also yielded the best cycling behavior. At a constant current density of 0.5 mA cm^−2^, it delivered a discharge capacity of 170 mAh g^−1^ in the first cycle and 150 mAh g^−1^ after 30 cycles.

Taking advantage of the sol–gel process accompanied by spinodal decomposition, which yields interconnected porous structure with uniform through-pores, Hasegawa et al. [296] prepared a hierarchically porous LTO anode material via the alkoxy-derived titania sol–gel technique. Porous TiO_2_ gels were treated in LiOH aqueous under relatively mild conditions, giving rise to platy-layered hydrous lithium titanate crystallites on the macroporous surface. The dissolution-reprecipitation leads to the flower-like structures composed of platy crystallites with a thickness of ≈30 nm when calcinated up to 700 °C. By combining the versatile sol–gel process and a hydrothermal reaction, Wang et al. [297] synthesized hierarchical hollow Li_4_Ti_5_O_12_ urchin-like microspheres with an ultra-high specific surface area of over 140 m^2^·g^−1^ and a diameter of more than 500 nm. LTO microspheres delivered a high specific capacity of 120 mAh·g^−1^ at 20C and long cycling stability of <2% decay after 100 cycles.

Erdas et al. [292] produced LTO powders via a sol–gel method, using citric acid as a chelating agent. Then, 5 mmol of titanium isopropoxide was dissolved in 150 mL of 2-propanol to obtain a saturated solution. Then, 4 mmol of lithium nitrate was added with mild stirring. A saturated aqueous citric acid was then added at a molar ratio of 9 mmol. pH of the final solution was kept at 7.0 by adding ammonium hydroxide. Then, excess ammonia and water were removed by heating the solution to 90 °C with vigorous stirring, until a transparent gel was obtained. After the drying process (12 h in an air oven at 120 °C forming a metal citrate precipitation), the precursors were decomposed at 450 °C for 4 h and 850 °C for 7 h in air to eliminate organic contents. The scanning electron microscopy (SEM) image indicates that the sample has a polyhedron morphology composed of subgrains (particle size ranging from 60 to 90 nm), and a uniform particle size distribution.

Erdas et al. [298] synthesized a Cu–LTO electrode by combining sol–gel process and electroless copper deposition technique. A one-pot co-precipitation method was carried out by Liu et al. [299] for the fabrication of Li_4_Ti_5_O_12_/V_2_O_5_ nanocomposites without surfactant. TBT and LiOH∙H_2_O were dissolved in ethanol and deionized water (at the molar ratio of Li:Ti = 4.1:5), respectively. V_2_O_5_ (2 wt.%) was added to LiOH∙H_2_O solution and stirred for 4 h, then the mixture was poured drop-wise into the TBT ethanol solution and was slowly stirred for 24 h. It is assumed that VO_3_^−^ ion formation with the dissolution of the added V_2_O_5_ restrained aggregation and dramatically reduced the particle size from 500 nm for pristine to 100 nm for V_2_O_5_-coated Li_4_Ti_5_O_12_. LTO was also synthesized using a modified and facile sol–gel method with a combined EDTA-citrate as a chelating agent [263,300,301]. The bi-components EDTA and CA were dropped in a mixed metal ion solution (i.e., stoichiometric amounts of TBT and Li_2_CO_3_ dissolved in an ethanol-HNO_3_ preblended solution in the volume ratio of TBT: ethanol:HNO_3_ of 1:12:0.5) with the mole ratio of total metal ions to EDTA and to CA is 1:1:2. EDTA usually forms four or six five-membered rings with most metal ions while the chelating reaction is fast, complete, and in one single step. As a result, the product is stable and dispersed and the presence of hydroxyl groups in the citrate structure makes the polymerization reaction between CA and EDTA feasible. This method provided particles of size 200–500 nm after a post-treatment at 750 °C for 5 h in air. The LTO nanoparticles showed a network morphology with high dispersion, giving a capacity of 164 and 108 mAh g^−1^ at 1C and 10C discharge rates, respectively. The result of the cycling performance showed a high-capacity retention of 97% at 1C and 25 °C after 1000 cycles.

The emulsion-gel process is a derivative of the sol–gel method. The gelation proceeds in a water–oil emulsion, so that both hydrolysis and condensation of metal alkoxides take place in a droplet, and spherical particles of metal oxides can be prepared at relatively low temperatures. The preparation of spherical LTO particles (with very narrow particle size (450 nm) distribution) was conducted as follows. The solution of TIP mixed with 180 mL of *n*-octanol and 0.3 g of hydroxypropyl cellulose (HPC, *M*_w_ = 62,500) was stirred with an emulsifier at 40 °C for 15 min. Subsequent dissolution of TIP (0.02–0.12 mol dm^−3^) in acetonitrile droplets generates emulsion. Then, 7.2 mL of LIOH aqueous solution was poured into the mixed solution to form a gel after stirring for 1 h [89].

Researchers from Sichuan University reported several works related to the sol–gel synthesis of LTO using various chelating agents. In the first report, various initial conditions were studied in order to find the optimal parameters for the synthesis of LTO using an oxalic acid-assisted sol–gel method with Li_2_CO_3_ and Ti(OC_4_H_9_)_4_ as starting materials [63]. LTO powders synthesized with an oxalic acid to titanium ratio of *R* = 1.0 heat-treated at 800 °C for 20 h have a uniform cubic morphology with an average particle size of 200 nm. In a second work [64], they reported a novel sol–gel method with triethanolamine (TEA) as a chelating agent, which exhibited an initial discharge capacity of 168 mAh g^−1^ and a subsequent charge capacity of 151 mAh g^−1^. TEA was used as a chelating agent in the sol– gel process. It could complex with Ti^4+^ to form a stable complex for preventing the excessively rapid hydrolysis of Ti^4+^. TEA can be used as a surfactant in many reactions to prepare powders with good dispersivity. The combustion heat generated due to the decomposition of the carbonaceous residue produced from TEA makes the nucleation process complete at the early stage of the sol–gel process, thus leading to a narrow size distribution of the LTO powders. They also reported [267] the synthesis of LTO (average particle size of 500 nm) by a sol–gel method assisted with citric acid in an alcoholic solution as a chelating agent and Li_2_CO_3_ and tetrabutyl titanate as starting materials. When the molar ratio *R* of citric acid to total metal ions was 0.5, LTO exhibited an initial discharge capacity of 167 mAh g^−1^ at 23.5 mA g^−1^ and a subsequent charge capacity of 151 mAh g^−1^. Besides these, they further investigated [63] the preparation of LTO via the oxalic acid-assisted sol–gel method, which exhibits 171 mAh g^−1^ in the first cycle and 150 mAh g^−1^ after 35 cycles under an optimal synthesis condition at 800 °C for 20 h, while oxalic acid to titanium ratio *R* = 1.0. Alias et al. [302] used lithium *tert*-butoxide (0.05 mol) and titanium isopropoxide (0.03 mol) as starting materials, which were together mixed in ethanol and DI water and kept for one week. The residue was harvested and finally sintered at different temperatures (700–1000 °C) and times (1–5 h). Recently, Pershina et al. [286] developed an optimized sol–gel method for the synthesis of single-phase LTO using tetraethoxytitanium (Ti(C_2_H_5_O)_4_, TET) as the Ti precursor and citric acid as a complexing agent. First, the hydrolysis of TET was carried out by stirring for 3 h followed by the dissolution of a white precipitate of metatitanic acid (TiH_4_O_4_) with the addition of diluted HNO_3_ (1:1), which resulted in a transparent titanyl solution. Then, the titanyl solution was added to a solution of Li_2_CO_3_ with citric acid and heated at 80 °C for 12 h to form a gel. Then the gel was heated in air to a temperature of ~200 °C and held for 5 h. Upon subsequent heating to 500 °C and, holding for 1 h, all organic compounds are completely decomposed and volatilized. It was found that the phase formation of lithium titanate is accompanied by the formation of an intermediate phase Li_2_TiO_3_ enriched in lithium. The final LTO product, consisting of particles 300–650 nm in size, was sintered at 750 °C for 1 h and 800 °C for 5 h in air. Recently, Llaín-Jiménez et al. [303] synthesized LTO powders (~150 nm average crystal size) via a modified sol–gel method using TBT, LiOAc, and CA as a complexing agent. Different samples were obtained, which differed in the addition rate of three sols: sol-1 formed by TBT added to ethyl alcohol (EtOH), sol-2 consisted of LiAs dissolved in EtOH added to sol-1, and sol-3 consisted of citric acid in EtOH. When sol-2 was gradually added to sol-3 within 45 min, pure LTO was obtained, while a quick mixture within 3 min produced an LTO/rutile (1.7%) hybrid. Recently, Kang et al. [178] investigated the impact of titanium precursors on the formation and electrochemical properties of LTO synthesized via sol–gel method. Lithium acetate dihydrate, anatase TiO_2_ or tetrabutyl titanate (TBT), and citric acid (CA) were used as Li sources, Ti sources, and chelating agents, respectively. In the synthesis, the proper amount of reagents with the Li:Ti:CA molar ratio of 4:5:4 was separately dissolved in 50 mL of 95% ethanol. The gel precursor was formed after heating the precursor solution at 120 °C under continuous stirring for 8 h and dried at 60 °C for 2 days to remove the residual solvent. Finally, well-crystallized LTO powders were obtained by a thermal pre-treatment at 350 °C for 4 h under air atmosphere and calcination at 800 °C for 12 h under air atmosphere. The sample synthetized with a TBT source revealed better particle dispersibility, and its particles were slightly smaller in size. These specific features resulted in better kinetic of Li^+^ ions during charge transfer reactions. The specific capacity values for both electrodes equal 150 and 63 mAh g^−1^ for TBT-LTO and 120 and 58 mAh g^−1^ for TiO_2_-LTO at C-rates of 1C and 10C, respectively. Table 7 lists the electrochemical performance of nanostructured LTO materials synthesized by the sol–gel route with different morphologies.

### 2.4. Pechini Process

The Pechini method is based on a gelation reaction between alcohol and acid used as solvents, unlike the sol–gel process in which the metal alkoxide participates in the gel formation reactions. The precursor is a polymeric resin, in which the cations are well distributed, yielding a nanostructured oxide during calcination. Currently, highly cross-linked resins containing a more uniform distribution of the reacting cations are obtained using a polyacrylic acid with higher functionality. Stenina et al. [275] prepared LTO samples with good ionic conductivity using the Pechini process. In this process, titanium tetrabutylate, citric acid, and Li_2_CO_3_ were sequentially added to an ethylene glycol + nitric acid mixture (5:2 volume ratio) with constant stirring. The titanium to citric acid molar ratio was 1:4. The resultant mixture was calcined at 70 and 130 °C for 24 h at each temperature. As a result, the ethylene glycol and citric acid molecules underwent polycondensation to give polymer gel, which was then pyrolyzed at 325 °C for 5 h. In the EDTA–citrate process, titanium tetrabutylate and lithium carbonate were dissolved in an ethanol + nitric acid mixture (5:1 volume ratio). An ammoniacal solution of EDTA and citric acid was added to the resultant solution. The molar ratio of metal:EDTA:citric acid was 1:1:2. The resultant mixture was calcined at 70 °C until a gelatinous substance was formed, which was then heat-treated at 225 °C for 5 h. Mo^4+^ partially substituted spinel Li_4_Ti_5−x_Mo_x_O_12_ was synthesized by the Pechini method using LiOOCCH_3_·2H_2_O, Ti[OCH(CH3)_2_]_4_, (NH_4_)_6_Mo_7_O_24_·4H_2_O, citric acid and ethylene glycol [309].

### 2.5. Hydrolysis (Wet-Chemical) Method

Hydrolysis is a chemical reaction of the interaction of chemicals with water, leading to the decomposition of both the substance and water. Generally, to obtain the final LTO phase, an intermediate titanium dioxide phase (either rutile or anatase) is prepared by reactions of hydrolysis using titanium tetrachloride (TiCl_4_), titanium(IV) butoxide (TBOT, Ti(OBu)_4_), as Ti source. In a typical process, LTO submicron-spheres are obtained using a three-step synthesis: (i) hydrolysis of TiCl_4_ to form rutile TiO_2_, (ii) hydrothermal treatment of rutile TiO_2_ with LiOH to prepare an intermediate phase of LiTi_2_O_4+δ_, and (iii) the calcination of LiTi_2_O_4+δ_ to obtain spinel Li_4_Ti_5_O_12_ [310]. Three major factors, including LiOH concentration, LiOH dosage, and hydrolysis temperature are optimized to obtain a phase-pure LTO [311]. Li et al. [312] have grown a perfect LTO crystal structure by the reaction of LiOH with Ti(OH)_4_, which is the hydrolysate of Ti(OC_4_H_9_)_4_ (Equation (11)) in the presence of ammonia to form the precipitation of LiTi_5_O_6_(OH)_9_∙6H_2_O (Equation (12)):

(11) $$\mathrm{Ti}({\mathrm{OC}}_{4}H_{9}{)}_{4}+4H_{2}O\;\overset{{NH}_{3}\cdot H_{2}O}{\to }\mathrm{Ti}(\mathrm{OH}{)}_{4}+4{\mathrm{CH}}_{3}({\mathrm{CH}}_{2}{)}_{3}\mathrm{OH},$$

LiOH + 5Ti(OH)_4_ ⮀ LiTi_5_O_6_(OH)_9_∙6H_2_O. (12)

Equation (12) approximates a reversible reaction, which indicates an ordered and even process. However, the amount of ammonia must be controlled to completely consume Ti(OH)_4_, which could be converted into TiO_2_ with excessive NH_3_. Recently, Wang et al. [313] prepared submicron-sized LTO particles through a simple hydrolysis method by using lithium acetate dihydrate (CH_3_COOLi·2H_2_O) and titanium (IV) butoxide (C_16_H_36_O_4_Ti) as raw materials and showed that sintering conditions significantly affect purity and dispersion of particle sizes. The optimized LTO product calcined at 650 °C for 20 h demonstrates small particle sizes (570 nm) and excellent electrochemical performances. In the voltage range of 1.0 to 3.0 V, it delivers an initial discharge capacity of 242 mAh g^−1^ and remains at 117 mAh g^−1^ over 500 cycles at the current density of 60 mA g^−1^; its discharge capacity remains at 64 mAh g^−1^ after 2500 cycles at 1.2 A g^−1^. Liu et al. [152] synthesized novel wave-like spinel LTO nanosheets using a facile “co-hydrolysis” method, which exhibits a capacity of ~150 mAh g^−1^ at high charge/discharge rates of 8.5 A g^−1^ (50C). LTO nanosheets with a scale of about 10 nm in thickness and 400–1000 nm in width/length were obtained by dissolving Li metal in 2-dimethylaminoethanol (DMEA) and 1-butanol reagents. Wang et al. [39] prepared monodisperse LTO nanospheres (diameter of 120 nm) via hydrolysis of peroxo-titanium prepared from TiN powders immersed in DI water, hydrogen peroxide and ammonia solution. After stirring for 30 min, a transparent yellow solution of peroxo-titanium complex was obtained. The peroxo-titanium complex was gradually hydrolyzed as described (Equation (13)):

2[Ti(OH)_3_O_2_]^−^ → 2TiO_2_ + 2H_2_O + O_2_↑ + 2OH^−^, (13)

that is, the addition of OH^−^ anions into the precursor solution can retard the hydrolysis of the peroxo-titanium complex, and thus reduce the aggregation of TiO_2_ and lead to the formation of monodisperse TiO_2_ nanospheres. The different preparations of the LTO spinel phases by hydrolysis route are summarized in Table 8.

### 2.6. Mechanochemical Synthesis

**Figure 10:**
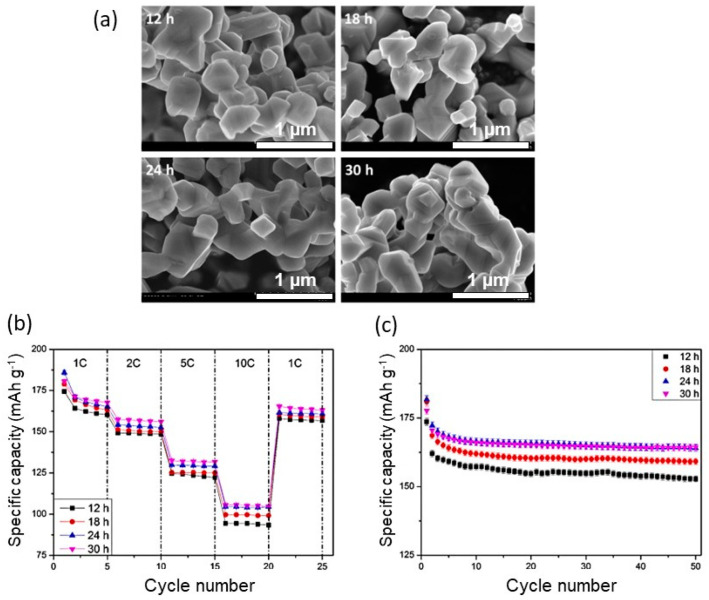
(**a**) SEM images of LTO obtained after 12, 18, 24, and 30 h of high-energy ball-milling process. (**b**) Rate capability as a function of synthesis time. (**c**) Cycleability tests at 1C rate. Reproduced with permission from [325]. Copyright 2014 Elsevier.

Mechanochemical synthesis, i.e., ball milling, has been widely used to synthesize LTO [92,93,170,205,238,316,317,318,319,320,321]. The ball milling technique meets the requirements of simplicity, ease of scale-up, low manufacturing and material cost, and lack of sophisticated apparatus. It is believed that mechanochemical actions such as ball milling generate local high-pressure and high-temperature environments. Therefore, the ball-mill process is always employed to help prevent aggregation and obtain reduced particle size. Different grinding systems are currently used such as attrition, planetary, or vibratory mills. According to the milling speed, we distinguish conventional ball milling (speeds <1000 rpm) and high-energy ball milling (HEBM), which is an efficient method due to its high rotor turning at speeds up to several thousand rpm using small grinding media. The effects of HEBM on the synthesis of LTO were reported by Zaghib et al. [238] and by Wang et al. [92], who studied the influence of the milling time on particle size. TiO_2_ anatase (~20 nm), LiOH∙H_2_O, activated carbon (~1993 m^2^ g^−1^), and glycerol were mixed at the weight ratio of 1.2:1.0:6.8:28.5 by ball milling and then calcined in air at 800 °C for 12 h. The product with a particle size of ~150 nm delivered a discharge capacity of 110 mAh g^−1^ at a 10 C rate. Han and co-workers [93] synthesized 100 g of LTO powder using 32.5 g of Li_2_CO_3_ (*D_m_* = 4.5 µm) and 86.8 g of anatase TiO_2_ (*D_m_* = 230 nm), which corresponds to a Li/Ti = 4.05/5.00, mixed with 200 g of DI water after adding 2 wt.% of the ammonium salt of polycarboxylic acid as dispersant (yellowish-brown liquid, viscosity of 85 cps). They controlled the particle sizes under four different milling conditions (i.e., ZrO_2_ bead sizes: 0.05, 0.10, 0.45, and 5 mm) at 3000 rpm for 3 h. The mean particle size was 146, 175, 215, and 285 nm, respectively. Pure LTO powders (rutile TiO_2_ and Li_2_TiO_3_ free) were obtained with ball sizes of 0.05 and 0.10 mm after calcination at 800 °C for 3 h. Hong et al. [317] investigated the influence of starting materials using either anatase or rutile TiO_2_, annealing temperatures (e.g., 700, 800, 900 °C), and mechanochemical activations (e.g., ball milling or high-energy milling). The hydrolysis process was performed using 200 mL DI water added to 2 wt.% of the ammonium salt of polycarboxylic acid, as a dispersant. These studies showed that mechanochemical activation by HEBM of the starting materials was more effective in decreasing the reaction temperature and particle size as well as increasing the LTO content of the final powder than those prepared by conventional ball milling. Thus, rutile TiO_2_ is more desirable in acquiring pure LTO than anatase due to the anatase-to-rutile phase transition. The changes in structure and morphology of starting materials and the effects, on the properties of the final products, have been reported [322]. In a typical experiment, TiO_2_ precursors are prepared with a HEBM machine at the speed of 2500 rpm using 0.4 mm ZrO_2_ beads as grinding media. After 1 h of grinding, the TiO_2_ particle size (*D*_50_) decreased from 1.0 to 0.5 µm. Li and co-workers [312] proposed a new LiCO_3_-ammonia-ball milling system to achieve the hydrolysis and condensation of Ti and Li sources step by step using ammonia as the adjustment and effectively controlling the number of formed crystal grains. The formed hydrate is in a metastable state, which is transformed into a perfect crystal of LTO with a high stability, e.g., spinel phase. The capacity of obtained LTO reaches 175 mAh g^−1^ at a current rate of 0.5C and keeps nearly 100% capacities after 300 cycles. Jia and co-workers [323] used a high-temperature (700–800 °C) ball milling process to synthesize LTO with different Li and Ti sources. The best product in terms of electrochemical performance is the sample prepared at 700 °C for 3.5 h using Li_2_CO_3_ and amorphous TiO_2_ as raw materials with the molar ratio Li:Ti = 0.82. In a second word, Jia et al. [324] reported that LTO prepared via a high-temperature ball-milling route without pretreatment contains Li_2_TiO_3_ impurities even at *T* = 800 °C. They suggested optimizing the experimental conditions with the assistance of ultrasonic dispersion, mechanical agitation, and glucose monohydrate (C_6_H_12_O_6_·H_2_O) as a solid phase dispersant was added to raw materials to refine grain, disperse evenly, and avoid agglomerating. The influence of the high-energy ball-milling time on the structure, morphology, and electrochemical performance of synthesized powders was investigated by Michalska et al. [325]. Remarkably, the electrochemical tests performed at high rates showed significantly improved specific capacity of the powders obtained at a constant speed of 200 rpm and extended processing time of 30 h (Figure 10).

Ball milling was also adopted to assist in the preparation of pure phase LTO. The production cost can be reduced by lowering the firing period [46,205,286,319,320,326,327,328,329,330,331,332]. The ball-milling assistance is capable to increase the reactivity of powders by reducing particle size and increasing particle surface areas, which highly facilitates the completeness of the synthesis. Mesoporous LTO was prepared in a planetary ball-milling-assisted solid-state reaction with titanyl sulfate (TiOSO_4_∙xH_2_O) and LiOH as precursors [46]. The sample annealed at 500 °C for 2.5 h shows agglomerates of nanoparticles with a size of ~8 nm, predominant mesopores centered at 1.9 and 3.8 nm, and a large specific surface area of 154 m^2^ g^−1^. Guerfi et al. [320] demonstrated that a pre-treatment by dry mechanochemical activation of precursors (anatase TiO_2_ and Li_2_CO_3_ ground during 30 and 300 min) is a successful process that reduces the firing time to 1/6 of that with standard mixed precursors (calcination at 800 °C for 1 h). The homogeneous coloration of powders compared to conventional milling indicates an intimate mixing of powders.

In the synthesis of LTO via a ball-milling-assisted sol–gel route, Yan and co-workers [326] evidenced that ball-milling and calcination temperature had a significant effect on the formation of the phase-pure Li_4_Ti_5_O_12_. The sol prepared from mixing 8.2 g of lithium acetate dissolved in 100 mL ethanol with a TiCl_4_ solution (Li:Ti ratio of 4:5) and citric acid as a chelating agent dissolved in solution B with stirring to form a transparent gelation was calcined in a muffle furnace at 800 °C for 10 h in air to obtain LTO precursors. Further, these products were ball milled and then calcined in the range 600–900 °C for 10 h in air. The precursor without ball milling exhibits two XRD peaks at 2*θ* = 27.4° and 54.3°, which were attributed to a secondary phase of rutile TiO_2_, while, the ball-milled sample is a pure LTO phase (lattice parameter *a* = 8.367 Å). Investigation of the influence of calcination temperature on the formation of LTO showed that the samples obtained by calcination up to 800 °C led to the single-phase spinel type, while impurities of rutile and anatase TiO_2_ existed in samples treated at temperatures lower than 700 °C. When the calcination temperature is beyond 900 °C, the impurity phase of TiO_2_ appears again, which is due to the loss of lithium at high temperatures.

Yuan and co-workers [205] synthesized LTO nanoparticles (grain size of 300–400 nm) from stoichiometric TiO_2_ anatase (~2 μm secondary particles composed of ~20 nm primary particles) and Li_2_CO_3_ mixed in liquid ethanol by HEBM. The milling process was conducted in the air using a planetary mill with grinding bowl sizes of 80 mL at a rotational speed of 400 rpm for 1 h. The wet-ball-milling-assisted solid-state reaction was associated with an ultra-high-speed nano-pulverization pretreatment process to grow LTO nanoparticles [245]. In this process, the precursor of TiO_2_∙xH_2_O (the hydrolysis product of tetrabutyl titanate) coated Li_2_CO_3_ was obtained by ball milling in air at 400 rpm rotational speed for 10 h. Kim et al. [327] reported the synthesis of LTO by a novel sol–gel method with high-energy ball milling of precursor. LTO anode material exhibited the first discharge capacity of 173 mAh g^−1^ and excellent cycleability. Suzuki et al. [213] demonstrated the mechanochemical-hydrothermal synthesis of layered lithium titanate hydrate, Li_1.81_H_0.19_Ti_2_O_5_∙xH_2_O (LHTO) nanotubes with lengths over 300 nm, using planetary ball milling of LiOH and TiO_2_ with water at room temperature. The LHTO precursor transformed into LTO nanotubes via a thermal treatment at 500 °C for 2 h. Pristine and La-doped LTO powders were fabricated via a simple ball-milling-assisted modified solid-state method [328]. The three-step procedure was conducted as follows: (i) mixture of a solution obtained by dissolving Li_2_CO_3_ in alcohol/deionized water (ratio of 4:1) with an alcoholic solution containing tetrabutyl titanate, (ii) drying the slurry mixture and calcination at 800 °C for 7 h, and (iii) dispersion of powders in a solution of alcohol and DI water and ball milling in air at 400 rpm for 10 h using agate-ball-to-powders weight ratio of 3:1. The white slurry was dried to obtain the final powders.

Recently, Pershina et al. [286] optimized the synthesis of LTO powders using a ball-milling-assisted solid-state reaction with Li_2_CO_3_ and rutile TiO_2_ starting materials. Before sintering, the blend was homogenized in an agate mortar or mechanically activated in a wet ball mill with agate balls 10 mm in diameter at a weight ratio of balls:charge of 3:1 at a speed of 750 rpm for 30 min with the addition of ethyl alcohol. Chauque et al. [331] mentioned that high-energy milling increases the specific capacity if crystalline degree remains high. Zhang et al. [332] mixed and active the LTO precursors by mechanical ball milling using ethanol as dispersant agent for 12 h (rate 330 rpm, ball feed ratio 16:1).

Jang et al. obtained LTO/CNT nanocomposites by complexing bulk LTO particles and carbon nanotubes (CNTs) via mechanofusion. In addition, the surface of the LTO/CNT material was easily fluorinated by physically mixing LTO/CNT with NH_4_F followed by heating at 300 °C for 2 h in a nitrogen (N_2_) atmosphere. The optimized surface fluorinated MF-LTO/CNT was obtained by mixing MF-LTO/CNT and NH_4_F in a mass ratio of 95:5, in which case the capacity reached ~170 mAh g^−1^ at 0.2 C, and ~140 mAh g^−1^ at a high rate of 20 C. At 10C, this composite showed a capacity of 128.5 mAh g^−1^ after 500 cycles. [329]. For comparison of different syntheses of LTO/CNT, we can mention the work of Ye et al. who synthesized composite anode materials with LTO nanoparticles uniformly chained by CNTs by a facile sol–gel process followed by high-temperature calcination step [330]. The best rate capability was obtained for 11% CNTs/LTO. At 0.2C, this composite delivered a capacity of 147.2 mAh g^−1^, 89.7% of which was retained at 30C. It was coupled with a superactive carbon (AC) cathode to assemble a Li-ion capacitor (LIC), with the mass ratio of AC cathode to 11% CNTs/LTO set at about 2.5. An energy density of 58.6 Wh kg^−1^ was achieved at a power density of 214.2 W kg^−1^, and an energy density of 35 Wh kg^−1^ was maintained even at a high-power density of 7434 W kg^−1^. This outperforms the properties of many other LTO-anode-based LICs.

Table 9 lists the electrochemical performances of nanostructured LTO materials synthesized by mechano-chemical method with different morphologies. Recently, Akintola and co-workers [288] synthesized LTO nanoparticles in a planetary ball mill with agate balls at a speed of 250 rpm using TBT, LiOAc as reagents, and a triblock copolymer P123 as the surfactant. This method provided well-defined-edge nanoparticles (average size of 350 nm) with a specific surface area of 55.7 m^2^ g^−1^ and good dispersion and uniformity.

### 2.7. Molten-Salt Synthesis

Molten-salt synthesis is a flux growth method, which has been reported to be one of the simplest means to prepare multi-component oxides [80,333,334,335,336,337]. The molten salt method was preferred since it does not need any complicated apparatus for material synthesis. It is also cost effective and no organic additives are needed. The method is characterized by an accelerated reaction rate and controllable particle morphology. The most important advantage is the prevention of particle agglomeration due to the diffusion of molten salts. Molten salts can provide a liquid reaction environment when serving as reaction media, thus accelerating the crystal formation reaction. Cheng et al. [333] synthesized nanosized LTO using LiCl only as a high-temperature flux and adopted this material for a coin-type cell and hybrid capacitor. However, the as-synthesized spinel LTO can deliver a capacity over 150 mAh g^−1^ (at a current rate of 40 mA g^−1^ between 1.0 V and 3.0 V) provided that the molar ratio LICl:TiO_2_ = 16. Zr- and Si-doped LTO microcrystals were synthesized via the molten-salt method using LiOH∙H_2_O and TiO_2_ as the starting precursor and LiCl and KCl, which act as fluxes. The stoichiometric quantities of LiOH·H_2_O and TiO_2_ were ground well for ½ h. Then the flux LiCl and KCl in the molar ratio of 50:50 was added to the above mixture and mixed for another half an hour to obtain a homogeneous powder. The resultant powder was then heated at 800 °C for 10 h with a heating rate of 10 °C min^−1^ to obtain the desired product. The particle size histogram infers that the maximum number of particles is lying in the size range of 1.75 μm [334]. Bai and co-workers [80] adopted a low content of LiCl–KCl (the molar ratio of LiCl–KCl to TiO_2_ is fixed to just 2 and the molar ratio of LiCl:KCl varies from 0.25 to 4.0) to prepare LTO particles. For LiCl:KCl = 1.5, the LTO particles are uniformly distributed (*D*_50_ = 3.4 µm) and have a well-developed octahedral shape. This material achieves high initial discharge capacity (169 mAh g^−1^), charge–discharge efficiency (94%) at 0.2*C* rate, and good rate performances from 0.2 to 5C.

Pure LTO particles with an average size of 1.3 µm were prepared by using the LiCl-KCl molten-salt method using anatase TiO_2_ (2 g, 100 nm in average particle diameter), LiOH·H_2_O (0.85 g), LiCl (10.2 g) and KCl (12.46 g) [336]. Rahman et al. [337] synthesized LTO–TiO_2_ composites via the molten salt method with minimization of the water content in the starting material mixture for the molten salt (LiNO_3_ -LiOH∙H_2_O). The products yield a high capacity (166 mAh g^−1^ at 0.5C) and good rate capability (110 mAh g^−1^ at 10C). The excellent performance was partly related to the presence of high grain boundary density among the particles. Nithya et al. [334,335] prepared several LTO anode materials via a single-step molten-salt synthesis using LiCl-KCl as a flux. Specific discharge capacities of 240, 265, 305, and 200 mAh g^−1^ were delivered by pristine LTO, Li_4_Ti_4.95_Mn_0.05_O_12_, Li_4_Ti_4.9_Mn_0.1_O_4_, and 5%Si-95%LTO composite at a current density of 0.1 mA cm^−2^. Sharmila et al. [338] reported the manufacturing of LTO via a single-step molten salt using LiOH∙H_2_O and TiO_2_ as the starting compounds and LiCl–KCl as a flux. The mixture heated at 800 °C for 10 h in air yields a uniform morphology displaying 3D polyhedral shapes with smooth surfaces and particle size of ~1.5–2 µm. A nanocrystalline Li_4_Ti_5_O_12_–TiO_2_ duplex phase was synthesized by a simple basic molten salt process (BMSP) using a eutectic mixture of LiNO_3_–LiOH–Li_2_O_2_ at 400–500 °C. The sample prepared by heat-treating at 300 °C for 3 h revealed dense agglomerates of ultra-fine nanocrystalline LTO; with heat treatment at 400 °C for 3 h, there was a duplex crystallite size (fine < 10 nm, and coarse > 20 nm) of Li_4_Ti_5_O_12_–TiO_2_; at 500 °C for 3 h, a much coarser and less-dense distribution of lithium titanate (crystallite size ∼15–30 nm) [339].

### 2.8. Solution-Combustion Method

The solution-combustion method is a facile and economical technique for the preparation of nanomaterials for lithium-ion batteries [340]. Combustion synthesis implies heating a precursor to a relatively low process temperature, after which the system generates the necessary energy for complete conversion and crystallization to the desired oxide. It has the advantage of relatively simple equipment, formation of high-purity products with size and shape control, and stabilization of metastable phases. The initial reaction medium is an aqueous or nonaqueous solution, the reactants are oxidizers (nitrates) and organic compounds as fuels (glycine, urea, α-amino-acid L-alanine, lactic acid, etc.), which are used as the source of C and H to liberate heat through combustion. Complexes formed with the metal ions could facilitate the homogeneous mixing of cations in solution. The combustion energy that is released upon heating the sample depends on the amounts of fuel and oxidizer in the precursor. Prakash et al. [97] reported a solution-combustion synthesis of LTO nanopowders using titanyl nitrate [TiO(NO_3_)_2_] and LiNO_3_ as precursors. In a typical reaction, which is completed in a few seconds, an aqueous redox mixture containing a stoichiometric amount of titanyl nitrate (0.0362 mol), 2 g of LiNO_3_ (0.0289 mol), and 4.22 g of glycine (0.0562 mol) were taken in a 120 mL alumina crucible and placed into a muffle furnace preheated to 800 °C. Raja et al. [103] reported an aqueous combustion process using titanium(IV) methoxide (Ti(OCH_3_)_4_) and Li_2_CO_3_ as reagents, and a common α-amino-acid L-alanine as fuel. Ti(OCH_3_)_4_ was dissolved in nitric acid and an aqueous solution of LiNO_3_, which yielded LTO nanoparticles of uniform morphology with an average particle size in the range of 40–80 nm. Yuan et al. [101,217] studied the mechanism of LTO synthesis using a cellulose-assisted glycine-nitrate combustion process. High-purity and well-crystallized LTO spinels were obtained at a calcination temperature of 750 °C via the sequence, for which the cellulose was first adsorbed by the mixed solution of LiNO_3_ and glycine followed by the impregnation of TiO_2_ suspension. Temperature-programmed oxidation experiments demonstrate that cellulose thermal pyrolysis creates a reducing atmosphere, which may facilitate oxygen-ion diffusion. In the combustion process performed by De Sloovere and co-workers [100], the Ti precursor was prepared using lactic acid added to the hydrated titanium hydroxide in a 3:1 molar ratio in aqueous solution. Then, the mixture was refluxed at 80 °C for complete dissolution and adjusted at a pH of ~6.8 by the addition of ammonia (35%). Yuan et al. [99] reported a glycine-nitrate combustion process. LTO calcined at 700 °C showed the best electrochemical performance, which reached a specific capacity of 125 mAh g^−1^ at a 10C rate with fairly stable cycling performance even at 40 °C. A similar method was used to prepare the La-incorporated Li_4_Ti_5_O_12_ with different La to Ti molar ratios via a one-pot cellulose-assisted combustion method [341]. Intermediate steps include the fabrication of a titanyl nitrate solution (transparent bright yellow) from the mixture of the precipitate TiO(OH)_2_ with nitric acid and the absorption of the mixed solution into dried activated cotton fibers. LTO nanopowders were prepared by solution−combustion synthesis using a titanyl nitrate [TiO(NO_3_)_2_] aqueous solution and LiNO_3_ as the oxidant precursors and glycine as the fuel, with stoichiometric amounts of titanyl nitrate (0.0362 mol), 2.1 g of LiNO_3_ (0.0303 mol), and 3.00 g of glycine (0.400 mol) [342]. Electrochemical performances of nanostructured LTO materials synthesized by the combustion method are provided in Table 10. 

The samples prepared via the glycine-assisted combustion method show micrometer-sized secondary particles composed of nanometer-sized primary particles, with uniform sizes in the range of 50–100 nm. Similarly, N-doped LTO nanopowders were prepared using urea (2.64–5.35 g) as fuel. HRTEM image showed that a thin shell with a thickness of approximately 5 nm was formed on the surface of the LTO nanocrystals from the thermal decomposition of urea. The nitrogen content of the shell was 8.5 wt.%, which is considerably greater than the average mass ratio (1.1 wt.%) of nitrogen in the LTO material. Chang et al. [280] synthesized LTO anode materials by a gel-combustion method with PVP as the polymer chelating agent and fuel followed by a post-annealing at 800 °C. Excellent reversible capacities of 167, and 144 mAh g^−1^ were achieved at the current densities of 0.5C and 5C. Yuan et al. [101] used a cellulose-assisted glycine-nitrate combustion process for a production of high-purity and well-crystallized LTO oxides. Particles of 0.2–0.8 µm were obtained after calcination at 750 °C via the sequence, for which the cellulose was first adsorbed by the mixed solution of LiNO_3_ and glycine followed by the impregnation of TiO_2_ suspension. 

### 2.9. Hydrothermal Method

Hydrothermal reaction (HTR) is a wet-chemical process using water as a solvent [342,343]. It is an efficient and facile strategy for controlling the chemical composition, and morphology of nanostructured materials (particle shape, and crystallite size) because it can decrease the activation energy of precursors in a simple and inexpensive way, so LTO can be obtained at 450–550 °C approximately. Nevertheless, the conventional hydrothermal process involves a very complicated reaction route and a long hydrothermal process (5–24 h) to synthesize LTO [344,345,346,347,348,349,350,351,352,353,354,355,356]. For example, LTO nanotubes were obtained as white precipitates from a mixture of titanium n-butoxide (TNBT), cetyltrimethyl ammonium chloride 25 wt.% aqueous solution (CTAC), and lithium hydroxide hydrothermally treated at 120 °C for 24 h [54]. However, the choice of precursors is crucial. It is difficult to avoid the formation of impurity phases like TiO_2_ and Li_2_TiO_3_ during the synthesis of LTO. The main reason may be the gradual deviation of the Ti:Li ratio from the desired proportion under a prolonged heating time at high temperatures, even when using different types of nanosized TiO_2_. This is due to the loss of the lithium salts during the heat treatment process and the less stability of most titanium salts in air. An accurate Ti:Li ratio can be controlled with the assistance of a solvent, which has the excellent ability to form hydrogen bonds with other compounds [342]. A soft synthetic route to obtain nano-LTO is realized by the introduction of organic additives such as surfactants (cetyltrimethylammonium bromide (CTAB), polyethylene glycol (PEG)), biomolecule (urea, vitamin C), ionic liquids, organic acids (citric, oxalic, EDTA), and alcoholic solvents (glycol, ethanol, glycerol). During the synthesis process, organic additives mainly act as the shape controller, reducing agent, and hydrolyzation inhibitor. Li et al. [344] reported that, in the hydrothermal process, the introduction of CTAB as a surfactant significantly improves the rate performance of LTO anode. The LTO/C composite (carbon layer with a thickness of ~1 nm) prepared in the presence of CTAB shows a larger diffusion coefficient of lithium ions (6.82 × 10^−12^ cm^2^ s^−1^) and smaller charge-transfer resistance (*R*_ct_) (19.2 Ω) than those of the composite (1.22 × 10^−13^ cm^2^ s^−1^ and 50.2 Ω) free from CTAB in the preparation. LTO nanowires were obtained from H_2_Ti_3_O_7_ prepared powders (0.5 g) through hydrothermal treatment in a LiOH aqueous solution (50 mL) at 100 °C for 24 h, in which lithiated titanate was formed via hydrothermal ionic exchange [132]. After washing the precipitates with ethanol until a pH of 7 was reached, the precipitates were dried at 60 °C and then calcined and heated at 800 °C for 6 h in air to obtain a pure LTO phase. Wu and co-workers studied the effects of the reactant concentration and heat treatment temperature on the phase structure of LTO nanosheets synthesized via hydrothermal method using Ti(OC_4_H_9_)_4_ and LiOH as the raw materials [345]. The best LTO nanostructure was obtained using 2 mol L^−1^ LiOH and heat treatment at 550 °C for 6 h. This material can deliver a specific capacity of 150 mAh g^−1^ at a 20C rate when the cut-off voltage is from 0.8 to 2.5 V. The best LTO nanostructure was obtained using 2 mol L^−1^ LiOH and heat treatment at 550 °C for 6 h. This material can deliver a specific capacity of 150 mAh g^−1^ at a 20C rate when the cut-off voltage is from 0.8 to 2.5 V.

Wang et al. embedded LTO nanoparticles in a reduced graphene oxide (rGO) conductive network by an in situ electrostatic self-assembly effect using a simple hydrothermal reduction method. The LTO nanoparticles were combined with rGO nanosheets by a Ti–O–C covalent bond, and rGO not only increased the conductivity but also prevented the agglomeration of LTO. In a half cell, this anode showed an outstanding rate capability, with a capacity maintained at ~272 mAh g^−1^ after the 1000th cycle at 10C when tested in a half battery [357]. For comparison, LTO nano-particles having a size in the range of 20–100 nm, wrapped and distributed uniformly inside the rGO sheets delivered a capacity of 141 mAh g^−1^ at 10C with a capacity retention rate as high as 97.2% after 1000 cycles [358].

**Figure 11:**
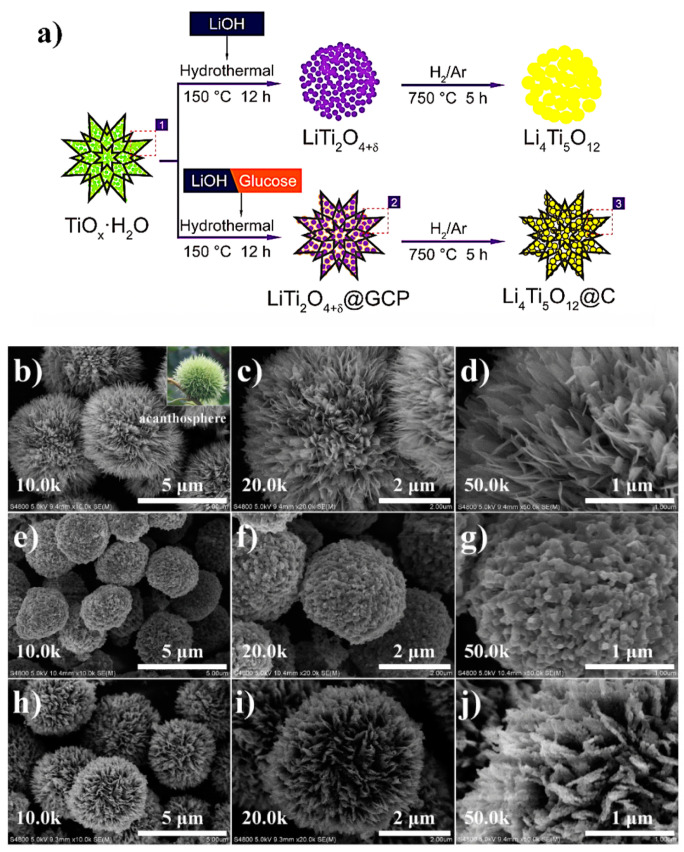
(**a**) Schematic illustration of the feasible morphology-tailoring process of the bare LTO and LTO@C microspheres via hydrothermal treatment. SEM images of the TiO_x_·H_2_O precursor (**b**–**d**), the bare LTO (**e**–**g**), and the LTO@C (**h**–**j**) samples with different magnifications. Inset in (**b**) shows a photograph of an acanthosphere. Reproduced with permission from [359]. Copyright 2016 Elsevier.

Hierarchical LTO nanoparticles were also prepared using a multi-step hydrothermal synthesis [150,352,359]. Ding et al. [352] fabricated a hierarchical yolk-shell Li_4_Ti_5_O_12_-SnO_2_ structure by controlling the reaction temperature of the hydrothermal process at 130 °C for 12 h. This composite maintained a specific capacity of 253 mAh g^−1^ after 200 cycles at a 1C rate. Wang et al. synthesized a nanoflower-like composite by coating a 3D conductive rGO and SnO_2_ nanoflower on LTO via an in situ electrostatic self-assembly and hydrothermal reduction process. [360]. Here, both the 3D rGO network and the doping of Sn ions on the LTO lattice improved the electron transport capability of LTO. In addition, SnO_2_ enhanced the mobility of Li in the interlayer of LTO/rGO/SnO_2_. Consequently, the LTO/rGO/SnO_2_ electrode showed remarkable rate capability, delivering a capacity of 445 mAh g^−1^ at a rate of 20C after 1000 cycles. DFT calculations showed excellent structural stability through the formation of Ti-O-C bond, Sn-O-C, and Sn-O-Ti bonds. The full cell with this anode and LiFePO_4_ anode has a high energy density of 130.56 Wh kg^−1^ with a high-power density of 2868.5 W kg^−1^. Zhu et al. used hydrothermal, freeze-drying, and calcination methods to embed SnO_2_ in carbon-coated LTO nanoparticles. At 0.2 Ag^−1^, this porous layered SnO_2_-Li_4_Ti_5_O_12_@C composite showed a capacity of 958 mAhg^−1^ after 300 cycles, and 766 mAhg^−1^ after 1000 cycles [361]. Sha et al. [151] investigated the phase development of LTO nanoplates prepared through a two-step hydrothermal method using either benzyl alcohol-NH_3_·H_2_O (BN) or ethanol-NH_3_·H_2_O (EN) as the Ti-source hydrolysis solvent. Ammonia in the solution increased the pH value of the solvent to facilitate Li^+^ diffusion and dissolution into TiO_2_·*n*H_2_O. In the benzyl alcohol–ammonia solution Li_1.81_H_0.19_Ti_2_O_5_·nH_2_O was successfully formed after a short hydrothermal treatment time of 30 min. The LTO-BN sample hydrothermally treated at 180 °C for 6 h showed superior electrochemical performance. The discharge capacity reached as high as 175 and 157 mAh g^−1^ at 1C and 40C, respectively; after charge–discharge cycling at 40C rate for 1000 times, capacity retention of 92.5% was still maintained with a decay rate of 0.0078% per cycle. Ultrathin Li_4_Ti_5_O_12_ nanosheet (6.6 ± 0.25 nm thick, specific surface area 178 m^2^ g^−1^) based hierarchical microspheres were synthesized through a three-step hydrothermal procedure [353]. First, TiO_2_ powder (2.0 g, P25) was dispersed in NaOH solution (80 mL, 10 mol L^−1^) by ultrasonic treatment. The obtained dispersion, i.e., sodium titanate (NaTO), was transferred into a 100 mL Teflon-lined stainless-steel autoclave and maintained at 120 °C for 24 h, and then the NaTO NWs were separated by centrifugation. Second, NaTO NWs (0.2 g) were dispersed in NaOH solution (38.5 mL, 2 mol L^−1^), and then, H_2_O_2_ (1.5 mL, 30%) was added into the solution. The solution was transferred into a 50 mL Teflon-lined stainless-steel autoclave and maintained at 150 °C for 12 h to prepare Na_2_Ti_3_O_7_. Third, the Na_2_Ti_3_O_7_ was added into HNO_3_ (0.05 mol L^−1^) solution, stirring for 12 h to exchange the hydrogen ions for Na^+^ ions, repeated two times, and the H_2_Ti_3_O_7_ intermediate phase (HTO) was achieved. Finally, the HTO was dispersed in LiOH solution (40 mL, 0.3 mol L^−1^) by ultrasonic treatment and the solution was transferred into a 50 mL Teflon-lined stainless-steel autoclave and maintained at 120 °C for 24 h. The products were separated by centrifugation, washed three times with water, and dried in an oven. After calcination at 400 °C for 4 h, the LiHTO-NS samples were achieved. Hu and co-workers [128] used the same hydrothermal/ion exchange process for the fabrication of surface-fluorinated LTO nanowires/reduced graphene oxide (F-LTO/rGO) composite. The fluorination was performed by an additional hydrothermal treatment of LTO/rGO nanowires in 3 mol L^−1^ NH_4_F solution at 160 °C for 6 h followed by a calcination at 500 °C for 4 h under an Ar/H_2_ atmosphere. The material exhibits nanowire morphology with a diameter of 50–100 nm and a length of ~10–20 µm. Hierarchical LTO porous microspheres with open structures of 4–6 µm diameters were prepared via a two-step hydrothermal process. The products consisted of a bunch of willow leaf-like nanosheets formed of LTO nanoparticles of 20 nm in size [359]. First, TiO_x_·H_2_O microspheres were prepared by HTR (at 150 °C for 24 h) of 1 mL tetrabutyl titanate dropped into 50 mL acetic acid. A second HTR (at 150 °C for 12 h) was used to prepare the final LTO product from 0.27 g TiO_x_·H_2_O microspheres mixed with 1.75 g of LiOH·H_2_O dissolved in 50 mL de-ionized water. Further sintering at 750 °C for 5 h in an H_2_/Ar atmosphere (10:90 in volume) was performed to obtain LTO microspheres; Similarly, LTO@C composites were prepared with the addition of glucose in the second HTR step (see Figure 11).

Nano-sized LTO pore microspheres (primary particles with a size between 30 and 40 nm) were prepared by hydrothermal treatment of Ti-(OC_4_H_9_)_4_ and LiAc·2H_2_O as raw materials and subsequent calcination at a high temperature of 1050 °C for 1 h, which provided big secondary particles (agglomerates) of 1.5–2.0 µm in diameter [362]. The nano-LTO exhibits a specific capacity of 133 mAh g^−1^ at a 10C rate and even 80 mAh g^−1^ at 60C. Porous LTO microspheres were also hydrothermally fabricated by Shen and co-workers [55]. Nano/microspherical superstructures, with sizes of ca. 4 µm in diameter, exhibited rich hierarchical pores and a specific surface area of 57.5 m^2^ g^−1^. At the high rate of 20C, the specific charge capacity was still ~92 mAh g^−1^, and less than 4.8% discharge capacity loss was observed after 200 cycles. Kim et al. [363] reported on Li_4_Ti_5_O_12_ microspheres assembled from nanosheets and synthesized by mixing Ti(IV)isopropoxide, LiOH, and H_2_O_2_ at 130 °C for 20 h under hydrothermal conditions; these nanosheets evidence a stable cycling performance, with high-capacity retention (140 mAh g^−1^) at a current density of 10 A g^−1^ even after 4000 cycles. Wu et al. [364] synthesized petal-like Li_4_Ti_5_O_12_–TiO_2_ nanosheets by boiling a lithium titanium peroxide–ammonium solution in an oil bath followed by a low-temperature, short-duration solid-state calcination. The resulting nanosheets give rise to excellent performance with a remarkably high discharge capacity of ~112 mAh g^−1^ at 20C rate and retain 97.1% of their initial capacity after 100 cycles. “Flower-like” motifs of Li_4_Ti_5_O_12_ consisting of thin petal-like component nanosheets were synthesized by using a facile and large-scale hydrothermal process involving unique Ti foil precursors followed by a short, relatively low-temperature calcination (500 °C for 3 h) in air [192]. The as-prepared LTO anode materials delivered capacities of 141 and 60 mAh g^−1^ at 10C and 100C, respectively, and capacity retention of 87 mAh g^−1^ at 20C rate after 300 cycles. Chen et al. [365] reported that a small amount of rGO (only 1.2 wt.%) greatly improves the whole morphology and electrochemical performance of the composite. LTO was prepared by hydrothermal method using 5 mmol TBT dissolved into 25 mL ethylene glycol mixed with 1 mL acetic acid, 0.5 g CTAB, and 2 mg rGO. After complete dissolution, this mixture was added to 0.336 g LiOH·H_2_O dispersed into 20 mL DI water and hydrothermally treated at 180 °C for 24 h. The nanoparticles uniformly grow on the rGO nanosheets effectively suppressing the agglomeration and enhancing the specific surface area. This anode material delivers high rates discharge capacity of 128 mAh g^−1^ at 80 C (discharge-charge time only 33s) and retains 50% of its initial capacity after 2000 cycles at 80C.

Several workers showed that the particle growth can be controlled through the hydrothermal route with the addition of glycerol, ethanol, or ethyl alcohol because of their excellent ability to form hydrogen bonds with starting materials [366,367,368]. In a typical ethyl-alcohol-assisted hydrothermal synthesis, 0.05 mol L^−1^ tetrabutyl titanate was dispersed in 17 mL ethyl alcohol forming a pale-yellow solution. Then, the 20.5 mL of 2 mol L^−1^ LiOH·H_2_O solution was added dropwise into this solution to form a white suspension with strong stirring for 15 min and then transferred to a 100 mL stainless-steel autoclave, which was maintained at 180 °C for 34 h to form a white precipitate [368]. Zhang et al. [354] added 2 mol L^−1^ LiOH in an aqueous solution to another solution formed by the mixture of tetrabutyl titanate and ethyl alcohol (volume ratio of 1:1) to form a white suspension further hydrothermally treated at 180 °C for 24 h. Glycerol has been used to assist the formation of various inorganic nano-materials in hydrothermal synthesis, because of its unique solvent properties. During the synthesis process, glycerol mainly assists in the dispersion of raw materials and acts as the shape controller, reducing agent, and hydrolyzation inhibitor. In a typical procedure of a glycerol-assisted hydrothermal process, Ti(SO_4_)_2_ (4 mmol) is dissolved in the solution formed by deionized water (15 mL) and glycerol (5 mL) and mixed with a fixed amount of LiOH∙H_2_O dissolved in deionized water (20 mL). Ti(SO_4_)_2_ is selected as the titanium source due to its good solubility and stability in solution [367]. During the hydrothermal process, glycerol is added as a modifier to assist the dispersion of starting materials, which can help to control the morphology of LTO. The glycerol-assisted samples have a cubic shape and the particle size is relatively uniform at 15–20 nm with a specific surface area of 86 m^2^ g^−1^ when calcined at 500 °C for 5 h in air.

**Figure 12:**
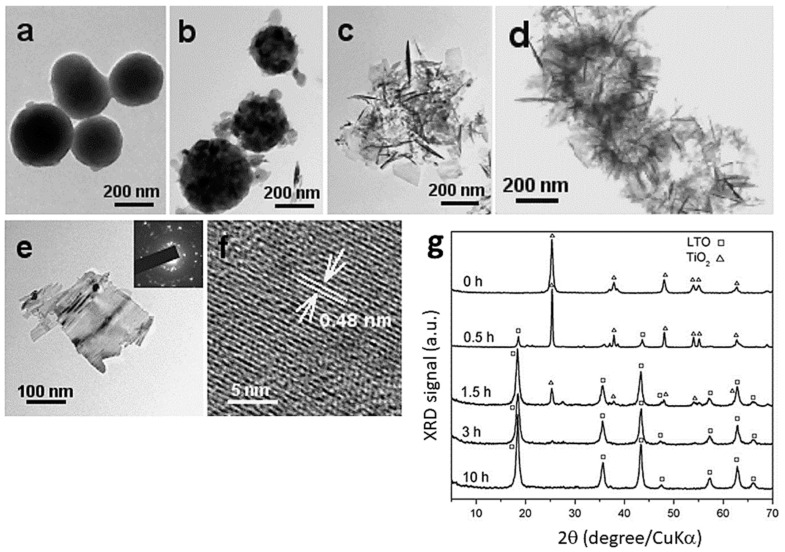
Hydrothermal synthesis of hollow structured LTO with the shell consisting of nanosheets prepared from AHTO. In a typical process of fabricating an LTO hollow structure, 4 mmol spherical AHTO colloids were dispersed in 20 mL, 0.2 mol L^−1^ LiOH solution. After stirring for 10 min, the suspension was transferred into a 30 mL Teflon-lined stainless-steel autoclave and then placed in an oven at 180 °C. TEM images present samples obtained for different hydrothermal reaction times: (**a**) 0 h, (**b**) 0.5 h, (**c**) 1.5 h, and (**d**) 10 h. (**e**) TEM image of a nanosheet from a hollow microsphere (inset is the SAED pattern taken from the nanosheet). (**f**) HRTEM image from the nanosheet. (**g**) XRD patterns of the products from different hydrothermal reaction stages and after calcination. The AHTO precursor is transformed to pure anatase TiO_2_ after calcination at 500 °C and the spinel LTO phase emerged after a 3 h reaction. Reproduced with permission from [52]. Copyright 2009 Elsevier.

The 3D nano/micro hierarchical structure, which combines the merits of nanosized building blocks with the benefits of thermodynamically stable microsized assemblies, represents an ideal host for Li storage. For instance, Tang et al. [146] prepared flower-like LTO nanosheets using 20 mL hot ethylene glycol (90 °C) with 10 mmol titanium tetraisopropoxide and 2 mL ammonia (25–28 wt.%) mixed in 8 mmol LiOH solution (30 mL H_2_O). After hydrothermal reaction at 170 °C for 36 h, the final product was obtained by calcination at 500 °C for 2 h. These structures exhibit a high reversible capacity and an excellent rate capability of ~166 mAh g^−1^ at 8C. Tang et al. [51] prepared hollow structured LTO with a shell consisting of nanosheets prepared from amorphous hydrous titanium oxide (AHTO). In a typical process to fabricate an LTO hollow structure, 4 mmol spherical AHTO colloids were dispersed in 20 mL, 0.2 mol L^−1^ LiOH solution. After stirring for 10 min, the suspension was transferred into a 30 mL Teflon-lined stainless-steel autoclave and then placed in an oven at 180 °C. Figure 12 presents the TEM images of samples obtained for different hydrothermal reaction times (0–10 h). TEM micrographs clearly demonstrate the evolution of structure from solid microspheres to hollow microspheres and finally to separated nanosheets. XRD patterns (Figure 12g) evidence the composition of products from different hydrothermal reaction stages and after calcination. TEM micrographs clearly demonstrate the evolution of structure from solid microspheres to hollow microspheres and finally to separated nanosheets. The AHTO precursor can transform to pure anatase TiO_2_ after calcination at 500 °C. With the reaction proceeding, the diffraction peaks of anatase TiO_2_ become weak, while the diffraction peaks of spinel LTO emerge after a 3 h reaction.

**Figure 13:**
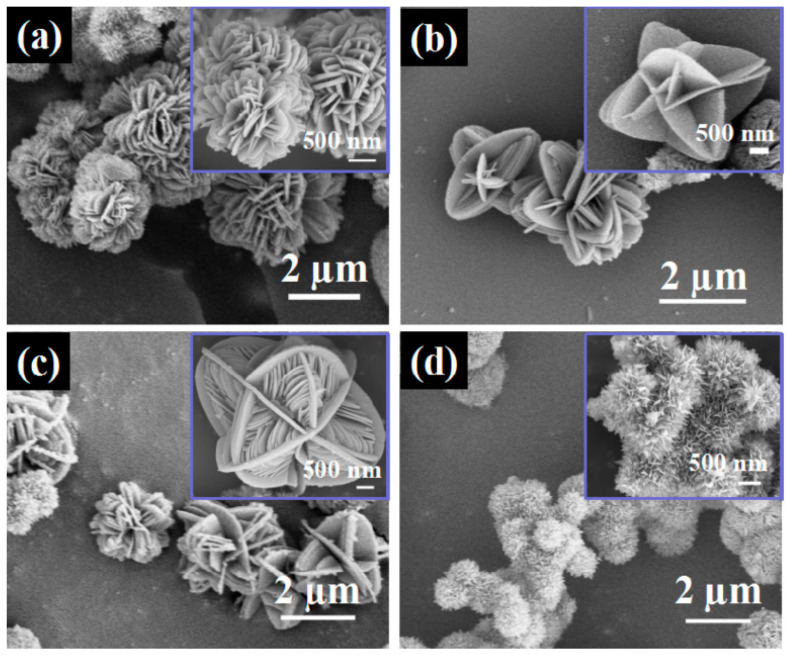
SEM images of the LTO-TiO_2_ samples obtained at different hydrothermal treatment times: (**a**) 12 h: flower-like nanosheets, (**b**) 15h: ultrathin nanosheets, (**c**) 18 h: uniform carambola-like frame, and (**d**) 24 h: urchin-like morphology. Reproduced with permission from [369]. Copyright 2016 Elsevier.

Hierarchical mesoporous LTO, with a nest-like structure (thickness of the wall of 50–80 nm), the tiny crystallite size of 8.2 nm, the very large specific surface area of 219 m^2^ g^−1^ and pore volume of 0.509 cm^3^ g^−1^, were prepared by a hydrothermal reaction, using CTAB as the surfactant, and [NH_4_^+^]_4_[H^+^]_2_[Ti_4_(C_2_H_2_O_3_)_4_(C_2_H_3_O_3_)_2_(O_2_)_4_O_2_]^6−^ water-soluble titanium complex as the titanium precursor [292]. Typically, the water-soluble titanium complex was prepared as follows: 2 mmol titanium metal powder was added to an ice-cooled aqueous solution consisting of 8 mL 30% H_2_O_2_ and 2 mL 28% NH_3_. After 3 h, 3 mmol glycolic acid was added, and this solution was heated at 80 ◦C with stirring for 6 h to remove excess H_2_O_2_ and NH_3_ and form a gel-like orange precursor. The 20 mL aqueous solution containing the Ti precursor, 0.3 mol L^−1^ CTAB, and 0.5 mol L^−1^ LiOH was stirred for 1 h, and hydrothermally treated into a Teflon-lined stainless autoclave maintained at 180 °C for 12 h. The products show remarkable electrochemical performance (i.e., low polarization and high specific capacities of 135 and 113 mAh g^−1^ at 2.5 and 10 A g^−1^, respectively, after 200 cycles). Zhang et al. [369] elaborated a versatile hydrothermal method for the synthesis of hierarchically structured Li_4_Ti_5_O_12_–TiO_2_ (LTO-TiO_2_) composites, by which the LTO and TiO_2_ phases were adjustable in the final products. With control over the time of the hydrothermal reaction at 18 h, an appropriate amount of anatase TiO_2_ (46.8%) can be obtained, and it possesses a uniform carambola-like framework assembled by numerous ultrathin nanosheets, which enables a relatively large specific surface area along with abundant interlayer channels to favor electrolyte penetration. Typically, 10 mL H_2_O_2_ aqueous solution (30%) was added to 150 mL of 0.5 mol L^−1^ LiOH solution. Then, 4.14 g tetrabutyl titanate was added to the solution. The H_2_O_2_ worked as a chelating agent, and could also slow down the hydrolysis of TBT. The hydrothermal reaction was carried out at 130 °C for 18 h (providing a light-yellow precipitate), then washed, dried at 80 °C and; finally, sintered at 500 °C for 3 h in air. Specifically, this anode material delivered a discharge capacity of 91.2 mAh g^−1^ at a very high current rate of 40C. Using the same synthesis process but prolonging the hydrothermal reaction time to 24 h, an urchin-like morphology was obtained (Figure 13).

Many researchers have optimized the synthesis of LTO using hydrothermal processes combined with other techniques such as microwave [351,370,371], sol–gel [372,373], dispersion [367], and ball milling [374]. Ultrathin LTO nanosheets with ordered microstructures were prepared via a polyether-assisted hydrothermal process [212]. The polyether (Pluronic P123) can impede the growth of Li_2_TiO_3_ in the precursor and also act as a structure-directing agent to facilitate the (Li_1.81_H_0.19_)Ti_2_O_5_·2H_2_O precursor to form the LTO nanosheets with the ordered microstructure. The synthesis was carried out as follows. First, a suspension was prepared: (i) a yellow solution was formed by TBT added into 15 mL of dehydrated ethanol, (ii) a solution was obtained by P123 dissolved in another 5 mL of ethanol, (iii) the dropwise mixing of solutions under continuous stirring for 4 h, (iv) finally, another 5 mL of LiOH (3.075 mmol) in a water solution was slowly added, where the stoichiometric ratio of Li/Ti was set as 4.1/5 with 2.5% excess Li. Then, the hydrothermal reaction was conducted at 180 °C for 18 h, and the solid product was calcined at 600 °C for 3 h in air. The ultrathin LTO nanosheets showed a rate capability much higher than that of the LTO sample without P123 in a Li battery with over 130 mAh g^−1^ of capacity remaining at the 64C rate. Hui et al. [371] showed that LTO prepared via microwave-assisted hydrothermal (MWHT) reaction using TiO_2_ and LiOH as raw materials exhibit interconnected nanoparticles (~40 nm in diameter) with mesoporous morphology (8 nm in pore size). MWHT-prepared LTO (at 170 °C for 2 h) delivered a specific capacity of 90 mAh g^−1^ at 60C (~10 A g^−1^), corresponding to a time of 60 s for full charge, which is superior to that of LTO synthesized via HTR at 160 °C for 48 h. Shi et al. proposed an advanced microwave-hydrothermal (MW-HT) method for the preparation of LTO microspheres composed of nanoflakes wrapped in graphene nanosheets [351]. First, 8 mmol LiOH was dissolved in 20 mL deionized water, 1 mL 30% H_2_O_2_ was then added to the solution, and finally, 2 mmol titanium isopropoxide was added to the solution and stirred for 20 min. The as-prepared solution was transferred to a polytetrafluoroethylene (PTFE)-lined autoclave, which was sealed for microwave irradiation. The LTO precursor, obtained after treatment at 150 °C for 15 min, was dissolved in 100 mL DI water with 0.25 mL 70% hydrazine hydrate and 0.1 g GO after 2 h of ultrasonic dispersion time to form the LTO/graphene composite. A sol–gel precursor and hydrothermal methods are used for the synthesis of LTO nanoparticles (100–200 nm) [279]. First, 2.38 g C_2_H_3_O_2_Li∙2H_2_O was dissolved in 10 mL of an anhydrous alcohol (Solution A). Then, 8 mL tetrabutyl titanate was dissolved in 30 mL of isopropyl alcohol (99.7%) (Solution B). Each solution was vigorously stirred at room temperature for 30 min, and solution A was then dumped into solution B under constant stirring. The suspension was placed inside a stainless-steel Teflon-lined reaction vessel (60 mL) heated at 160 °C for 24 h. Finally, the prepared sample was dried and calcined at 800 °C for 10 h under an Ar atmosphere. Recently, a novel self-supported LTO nanobelt array was fabricated on the titanium foil by a two-step hydrothermal process and subsequent ion exchange [145,165].

Ti foil can act as both the Ti precursor for LTO growth and a current collector. First, titanium foils in a mixed NaF/NaOH solution were hydrothermally treated at 180 °C for 24 h resulting in Na_0.98_H_1.02_Ti_4_O_9_·3.3H_2_O, which was transformed to H_2_Ti_5_O_11_·3H_2_O after protonation in 0.1 mol L^−1^ HCl solution, indicating that Na^+^ in sodium titanate can be completely replaced by H^+^. The second hydrothermal process with 0.2 mol L^−1^ LiOH produced the precursor (Li_1.81_,H_0.19_)Ti_2_O_5_·2H_2_O. Finally, the precursor calcined at 450 °C for 1 h in air to form LTO nanobelts. Wu et al. [148] explored the formation of ultrathin dual-phase nanosheets consisting of alternating spinel LTO and rutile-TiO_2_ lamellas through a facile and scalable hydrothermal method. The white suspension appeared immediately once the LiOH·H_2_O aqueous solution was slowly dropped into the Ti(OC_4_H_9_)_4_ ethanol solution and further hydrothermally treated at 180 °C for 24 h and calcined at 600 °C for 6 h in a horizontal tube furnace in air. The thickness of constituent lamellas can be controlled exactly by adjusting the mole ratio of Li:Ti in the original reactants. The best electrochemical performance, i.e., the stable discharge capacity of 123 mAh g^−1^ at a current density of 50C after 500 cycles, was obtained with Li:Ti = 4.1:5. LTO nanorods with sizes of 100–200 nm in diameter and 1–2 µm in length were prepared via a hydrothermal process of TiO_2_-B nanorods with LiOH in aqueous solution [124]. The final product heat treated at 500 °C for 4 h exhibits a high specific discharge capacity of 101 mAh g^−1^ after 1000 cycles at a 20C rate.

A double-hydrothermal method was performed to prepare LTO nanobelt arrays. First, stamped titanium foils were hydrothermally treated in NaOH and NaF solution at 180 °C for 24 h giving the product Na_0·98_H_1·02_Ti_4_O_9_·3.3H_2_O, which was subsequently transformed into H_2_Ti_5_O_11_·3H_2_O by protonation in acid. Next, lithiation was obtained by a second hydrothermal reaction resulting in (Li_1.81_,H_0.19_)Ti_2_O_5_·2H_2_O, which was finally transformed to pure spinel Li_4_Ti_5_O_12_ after calcining [159]. Nanoparticle-stacked Li_4_Ti_5_O_12_-TiO_2_ nanowire arrays were constructed via a hydrothermal/ion exchange process [375]. Films obtained by hydrothermal treatment of Ti foils in NaOH+NaCl solution were immersed in a diluted HCl solution for several hours to thoroughly exchange the Na^+^ with H^+^. The resultant H_2_Ti_2_O_5_·H_2_O arrays were placed in a 2 mol L^−1^ LiOH·H_2_O solution at 60 °C for 10 h and calcined in N_2_ at 600 °C for 3 h. This electrode material achieved a specific capacity of ~200 mAh g^−1^ at a current rate of 0.75C (potential range of 1.0–3.0 V). Huang et al. [14] fabricated pure LTO and LTO-rutile TiO_2_ (LTO-RTO) composite via HTS by adjusting the amount of TBT as a Ti source. LTO-RTO was obtained using 16.25 g of TBT dissolved in 34 mL alcohol and mixed with 1.68 g of LiOH·H2O dissolved in 20 mL of distilled water. The gained white suspension was transferred into a 100 mL stainless-steel autoclave and then reacted at 180 °C for 36 h. After filtrating and drying at 80 °C for 7 h, the precipitate was calcined at 600 °C for 10h and transferred into final product. By changing the amount of TBT to 15.10 g, the pure LTO was obtained using a similar procedure. The Li_4_Ti_5_O_12_/C composite with lump morphology and excellent rate performance was synthesized using a facile hydrothermal process, in which CTAB was introduced as a surfactant significantly improving the rate performance. The specific capacities of the obtained composite at charge and discharge rates of 0.1, 1, 5, 10, and 20C are 176, 163, 156, 151, and 136 mAh g^−1^, respectively [344]. Electrochemical performances of nanostructured LTO materials synthesized by hydrothermal reaction are provided in Table 11.

### 2.10. Supercritical Synthesis

Supercritical water (SCW) has low viscosity, high diffusivity, and zero surface tension. The enhancement of the reaction rate and high supersaturation based on the nucleation theory due to lowering the solubility is another motivation for the use of SCW. These synergetic effects have been used to prepare LTO powders with high—rate capability using supercritical hydrothermal synthesis in batch process. In practice, the reaction media to prepare LTO free of TiO_2_ and Li_2_TiO_3_ impurities is SCW, *T*_c_ = 374 °C, *P*_c_ = 221 bar [377,378,379]. The effects of various synthesis conditions—feed concentration, reaction time, and calcination—on the particle properties, including particle size, surface area, particle morphology, phase purity, and crystallinity, were carefully analyzed [379]. In this work, each of the Ti-sol and LiOH solutions was fed forward to a reactor by high-pressure pumps X-500 at a flow rate of 5 g min^−1^, and these two streams were mixed. On the other hand, distilled water was fed by another high-pressure pump at a flow rate of 40 g min^−1^ and heated to an appropriate temperature by an electrical furnace. Then, the reactant mixture of Ti sol and LiOH was mixed with the SCW. Phase-pure LTO particles are obtained with a long reaction time of 6 h in supercritical water at 400 °C and 300 bar without subsequent calcination, while the anatase TiO_2_ impurity phase is detected at shorter reaction times of 5 min to 2 h. Nugroho et al. [377] synthesized LTO by reacting lithium hydroxide monohydrate and titanium(IV) isopropoxide (TIP) in supercritical water using a tube reactor of 11 mL inner volume. Free-impurity quasi-spherical LTO nanoparticles were obtained with an SSA of 38 m^2^ g^−1^ and a size of 50–100 nm. The SCW LTO products show a high initial discharge capacity of 212 mAh g^−1^ at a 1C rate in the potential range of 0.1–3.0 V vs. Li^+^/Li and a capacity retention of 91% after 50 cycles. Further, the same group of researchers fabricated, in a very short time, hierarchical mesoporous LTO microspheres in supercritical methanol [380]. The suspension of lithium hydroxide and TIP in methanol was transferred in a tube reactor maintained at 400 °C, in which the pressure increased to 30 MPa in 1 min. Hayashi et al. [379] reported the synthesis of LTO nanoparticles via supercritical water flow hydrothermal reaction method using TiO_2_-sols and Ti(SO_4_)_2_ as the Ti source. The syntheses were carried out at temperatures from 350 to 410 °C, at pressure of 30 MPa, and for residence times from 10 to 26 s. Particles of BET-specific surface area of 82 m^2^ g^−1^ are in the range of several 10 nm to 50 nm and particle size did not change during the annealing process up to 400 °C. Li_4_Ti_5_O_12_ was crystallized directly via a continuous flow supercritical synthesis using lithium ethoxide and TIP as reactants. Crystalline nanoparticles are obtained in a single step and in less than one minute, by mixing the reactants with superheated water in a continuous flow reactor at near- and supercritical conditions. The LTO nanoparticles have an average crystallite size of 4.5 nm with a specific surface area of ≥230 m^2^ g^−1^ [381].

### 2.11. Solvothermal Method

Unlike the hydrothermal technique, non-aqueous solutions are used in the solvothermal synthesis, which allows for easy control of the morphology, particle size, and homogeneity of the products. Since a variety of organic solvents, e.g., ethanol, benzyl alcohol, polyol, etc., with high boiling points can be selected, the temperature can be elevated much higher than that in the hydrothermal method [59,60,382,383,384,385,386,387]. For example, LTO nanosheets [209], nanoflakes [384], sawtooth-like nanosheets [385], and nanoflowers [146] fabricated by solvothermal method exhibit good electrochemical performance. The solvothermal reaction can be performed by 0.1 mol tetrabutyl titanate dissolved in 100 mL ethanol and then followed by the addition of 0.1 or 0.08 mol LiOH. The undissolved LiOH particles were suspended in the orange solution. The alcoholic suspension was solvothermally treated at 140 °C for 24 h in a 150 mL autoclave under pressure of ~747 kPa. The direct solvothermal synthesis of the LTO nanocrystals was achieved through the self-control of the basicity using a two-phase system of water–ethanol and toluene–oleic acid. The Li–Ti–O precursor was produced under relatively high pH conditions (pH > 11) in the initial stage. The spinel phase was then formed through delithiation and dehydration from the precursor by lowering the pH of the system (pH < 10) [386]. Feckl and co-workers [387] created ultrasmall building blocks for a porous spinel framework in the absence of water, using a solvothermal reaction in *tert*-butanol, which is an excellent reaction medium for the synthesis of ultrasmall crystals at a low temperature of 170 °C. Yu et al. [203] introduced a one-pot template-free solvothermal synthesis of crystalline Li_4_Ti_5_O_12_ nanostructures based on the “benzyl alcohol route”. Spherical particles (1–2 µm size) are constituted of nanocrystals in the size range of a few nm. Microwave wet chemistry not only reduces the chemical reaction times by several orders of magnitude but also offers high reaction yields and reproducibility of synthesis protocols due to the suppression of uncontrolled side reactions [189,388]. Nowack et al. [189] presented a novel, rapid, and low-temperature synthesis protocol to achieve battery-grade LTO microspheres using a solvothermal microwave-assisted method. The solution of metallic lithium dissolved in benzyl alcohol and titanium isopropoxide (Li:Ti = 0.8) was treated in a microwave reactor at 260 °C for a period of less than 2 min. Products are spherical micron-sized particles with an average size of 4.4 µm, which were electrochemically tested at 60 °C, delivering a specific capacity of 136 mAh g^−1^ at 10C rate. Several researchers combined the solvothermal method with other techniques such as sol–gel and ball milling [372]. Recently, Gangaja et al. [389] synthesized Li_4_Ti_5_O_12_ nanoplates by an off-stoichiometric solvothermal process, which leads to the coexistence of phase-separated crystalline nanoparticles of Li_4_Ti_5_O_12_ and TiO_2_ exhibiting reasonable high-rate performances. An ultrahigh-charging-rate capability of up to 1200C (60 mAh g^−1^; discharge limited to 100C) was demonstrated.

**Figure 14:**
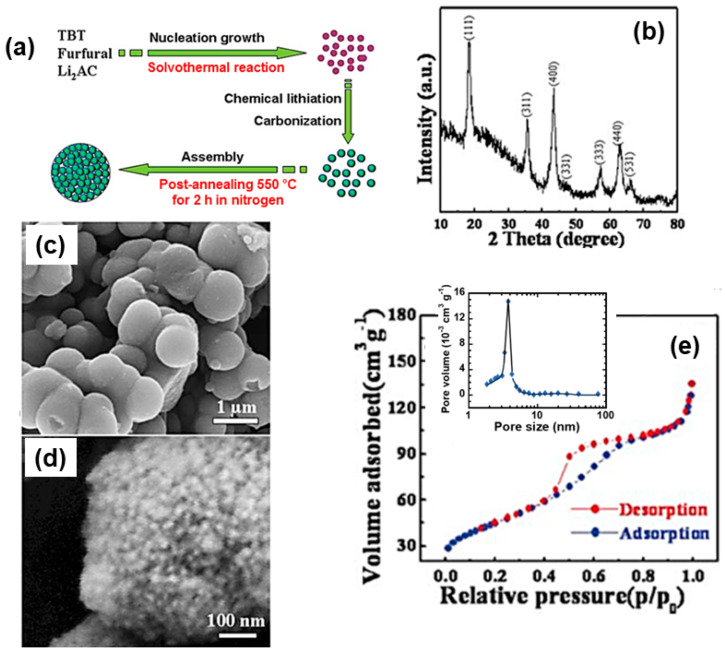
(**a**) Schematic representation of the solvothermal synthesis of mesoporous LTO-C microspheres. (**b**–**e**) Characterization of mesoporous LTO microspheres. (**b**) XRD pattern, (**c**,**d**) FE-SEM images, and (**e**) N_2_ adsorption/desorption isotherm (inset: BJH distribution). Reproduced with permission from [60]. Copyright 2011 The Royal Society of Chemistry.

Santhoshkumar et al. [390] report a homogeneous composite of integrated LTO-TiO_2_ (LTO-TO) nano-hybrid synthesis by a solvothermal technique followed by a calcination process. CTAB was used not only as a precursor but also as a surfactant to optimize the composition. The optimized hybrid showed a capacity of 468 and 83 mAh g^−1^ at current densities100 to 8 A g^−1^, respectively. After 300 cycles, at a current density of 5 A g^−1^, the capacity was still 198 mAh g^−1^. In this work, the calcination temperature was 600 °C. A lower temperature is recommended to avoid the transition from the phase TiO_2_(B) to the anatase phase [391]. The best results with LTO-TO were obtained by Lu et al. who fabricated TiO_2_(B) nanosheets on the surface of the LTO microsphere by using a solvothermal method to obtain LTO-TiO_2_(B) (LTO-TOB) composite after calcination at 300 °C for 2 h. This anode demonstrated superior rate capability (145, 139, and 124 mAh g^−1^ at 20C, 30C, and 50C, respectively) and superior cycling stability (98.9% capability retention after 500 cycles at 5C) [392]. Meso-structured, micron-sized LTO/C dyad spheres with high surface area were successfully synthesized via solvothermal treatment of tetrabutyl titanate and lithium acetate in the presence of furfural (heterocyclic aldehyde, CHO) allowing for the formation and co-assembly of carbon and LTO crystallites into the interpenetrating nanoarchitecture [60]. In the first step, TiO_2_ crystallites are formed by a controlled slow nucleation process via a solvothermal reaction of 1.2 mL TBT, 1.0 mL furfural, and 2.5 g LiOAc dissolved in 60 mL ethanol. Afterward, the chemical lithiation of TiO_2_ and carbonization of furfural completed simultaneously under solvothermal conditions result in the formation and co-assembly of micrometer-sized L-T-O-C intermediate phase. Finally, the L-T-O-C network is converted to mesoporous LTO-C microspheres by a short post-annealing at 550 °C for 2 h in the N_2_ atmosphere (Figure 14).

Li_4_Ti_5_O_12_/TiO_2_ (LTO/TO) nanoparticles were synthesized via a one-step solvothermal method followed by further heat treating using LiOH·H_2_O and TBT as raw materials [384]. Typically, 0.2 g LiOH·H_2_O was completely dissolved in 50 mL of ethanol after ultrasonication for 30 min as solution A. Then, 4 mL of TBT was dissolved in 10 mL of ethanol as solution B. Solution B was then slowly dropped into solution A with magnetic stirring. The mixed solution was transferred into a 100 mL Teflon-lined stainless-steel autoclave and reacted at 200 °C for 24 h. After natural cooling to ambient temperature, the precursor product was collected by centrifugation, washed with ethanol and ultrapure water several times, and dried overnight at 80 °C. Finally, the LTO/TO sample was obtained via calcining at 600 °C for 3 h in air. Though the same procedure, the pure LTO phase can be synthesized by dissolving 2 mL of TBT in 10 mL of ethanol to form solution B. Electrochemical performances of nanostructured LTO materials synthesized by the solvothermal method are provided in Table 12.

### 2.12. Reflux Method

The solution-based reflux method is a green synthesis method used to grow nanostructured inorganic materials. Reflux involves heating the chemical reaction for a specific amount of time, while continually cooling the vapor produced back into liquid form, using a condenser. The vapors produced above the reaction continually undergo condensation, returning to the flask as a condensate [90,393,394]. In 2004, Singhai and Skandan [393] patented a new method to prepare nanostructured LTO powders using a reflux-type synthesis. The process utilized: (i) nanoparticles of TiO_2_ (~20–25 nm), (ii) a lithium salt, and (iii) an organic solvent such as hexanol (CH_3_(CH_2_)_5_OH) with a boiling point of 158 °C. The solution was refluxed for 15 h (heated to a temperature and at a pressure to facilitate the diffusion of Li ions into the TiO_2_ nanoparticles). Annealing at 500–800 °C in an O_2_ atmosphere provides ultrafine powders of LTO with particles of average size of ~500 nm, composed of nanocrystallites with an average size of approximately 30 nm. The effect of the solvent on the phase purity was investigated. Unreacted TiO_2_ material and other unidentified impurities were identified when LiOH·H_2_O and nano-TiO_2_ (or Li_2_CO_3_ and nano-TiO_2_) were refluxed in water. In another attempt, pure LTO particles were obtained by refluxing the TiO_2_/LiNO_3_/methanol mixture for approximately 15 h. Kim et al. [90] used a reflux method to obtain LTO nanoparticles (6–8 nm in size), which were prepared from ethylene glycol solution of titanium tetra-isopropoxide (Ti(O-*i*Pr)_4_) and excess Li_2_O_2_ by refluxing at 197 °C for 12 h. The obtained particles were filtered and dried at 100 °C for 12 h, as a post-reaction step, and the dried powder samples further heated. Post-treated at 500 °C for 3 h, the LTO anode exhibited the initial capacity of 320 mAh g^−1^ at a current density of 0.05 mA·cm^−2^. Yagi and co-workers [141] reported the growth of LTO nanotubes. First, a colloidal solution of anatase TiO_2_ in 10 mol L^−1^ NaOH hydrothermally treated at 150 °C for 24 h resulted in Na^+^-intercalated hydrogen titanate nanotubes (TNTs). Then, Na+ ions in the interlayer of the synthesized nanotubes were exchanged with Li^+^ ions by a reflux treatment using 100 mL of a 2.5 mol L^−1^ LiOH aqueous solution, leading LTO nanotubes (diameter and length of ~ 5 nm and several submicrons, respectively) when post-annealed at 500 °C for 2 h, while cubic powders 10 − 50 nm were obtained at 700 °C. Lin et al. [180] prepared monodispersed mesoporous LTO submicrospheres by the solvothermal process using a water–ethanol (60 vol%) solvent followed by a calcination process at 600 °C, revealing a large sphere size of 660 ± 30 nm with a small primary particle size of 20–100 nm, a large specific surface area of 15.5 m^2^ g^−1^, an appropriate pore size of 4.5 nm and an ultra-high tap density of 1.62 g cm^−3^. They displayed a charge capacity of 179 mAh g^−1^ at a C/2 rate between 1.0 and 2.5 V vs. Li^+^/Li and an initial Coulombic efficiency of 93.5%.

Yin et al. obtained in situ self-assembly of LTO submicron spheres by simple one-step liquid phase deposition using a low-cost inexpensive titanium source (H_2_TiO_3_). The sample calcined with 4 mL of 25% NH_3_‧H_2_O at 700 °C showed the best performance. Its capacity remained at 176.4 mAh g^−1^ over 100 cycles at 87.5 mA g^−1^, 136.7 mAh g^−1^ over 500 cycles at 875, and 105.6 mAh g^−1^ over 1000 cycles at a current density of 1750 mA g^−1^ with a retention rate of 96.7% [395].

### 2.13. Templating Method

Utilization of a template may help the formation of porous structures in materials. Various templates have been used to synthesize LTO composites that resolve the predicament of agglomerates [110,396,397,398]. The synthesis of LTO nanocrystals from solution-phase precursors using a carbon-templated growth process achieves complete crystal growth (all of the nutrients are consumed), and the surfaces are annealed at high temperature at nearly the same rate at which the template burns off [85,86,87,88,397]. Monodisperse Li_4_Ti_5_O_12_ hollow spheres were prepared by using carbon spheres as templates [85]. Scanning electron microscopy images show hollow spheres that have an average outer diameter of 1.0 μm and an average wall thickness of 60 nm.

Lv and co-workers [154] implemented a method involving the in-situ oxidation and gasification of the phenol-formaldehyde (PF) resin as a “template” to prepare porous LTO with a mesopore size of 2–20 nm. TiO_2_-rutile, Li_2_CO_3_, and water-soluble phenol–formaldehyde (10 wt.%) resin (solid content 60%) were used as starting materials. Firstly, PF resin was dissolved in the solution of de-ionized water and alcohol, and then TiO_2_ and Li_2_CO_3_ (at the molar ratio of Li:Ti = 4.2:5) were mixed, respectively, in the solution under magnetic stirring. After 0.5 h stirring, the dark red solution was treated at 65 °C to remove solvents gradually. The mixed slurry was dried at 120 °C in the vacuum drying oven to solidify the PF resin. Then, after ball milling, the precursors were heat-treated at 800 °C for 8 h under an air atmosphere. Alginic acid aquagel was used as a template and carbon source in the one-pot solid-state synthesis of Li_4_Ti_5_O_12_/C nanocomposites [249]. The final product was obtained by the pyrolysis at 800 °C for 3 h under argon flow (50 mL min^−1^) of the mixture of 470 mg of TiO_2_, 505 mg of LiOAc, and the resulting aquagel formed by 200 mg of alginic acid gelled in water (10 mL) by heating at 90 °C for 2.5 h.

**Figure 15:**
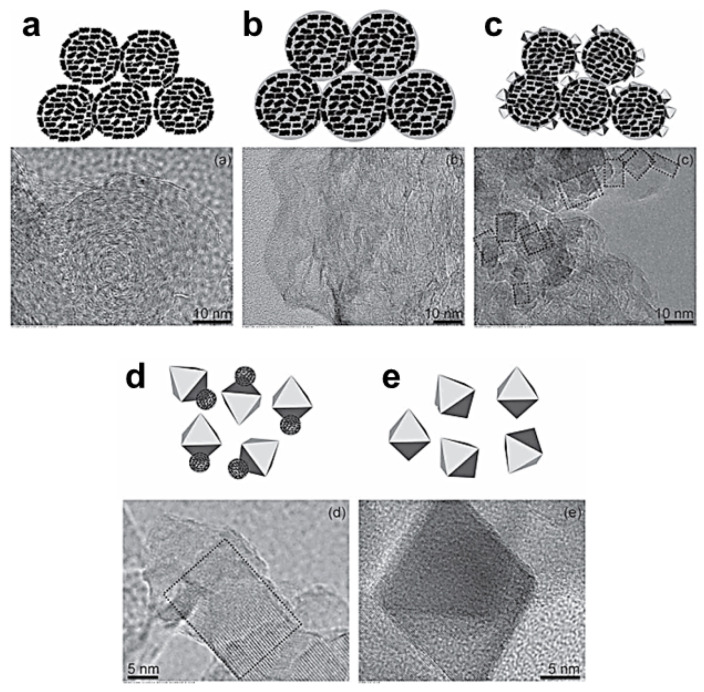
TEM images highlighting the growth mechanism of C-LTO nanocrystals from a carbon black template. (**a**) Carbon template at room temperature composed of 50 nm spheres, (**b**) precursor solution loaded with the carbon template, (**c**) crystalline domains (~10 nm in diameter) growing off of the carbon black template, (**d**). crystalline domains after annealing at 450 °C for 1 h, (**e**) final product as regular octahedra after annealing to 700 °C. Reproduced with permission from [397]. Copyright 2013 Wiley-VCH.

Polystyrene (PS) beads of 400 nm diameter were used to prepare the template for the LTO inverse hemispheric structure, which was fabricated on a Pt/Ti/SiO_2_/Si substrate by the sol–gel and dip coating method. A coating solution prepared using precursor sources was dropped on the template-deposited substrates, which were then calcinated at 400 °C [398]. Mesoporous LTO thin-film electrodes with open pores of ~18 nm in diameter were prepared by a soft-templating method derived from sol–gel [188]. Thin films were produced via dip-coating on polar substrates, using a mixture of 333 mg of Ti(OBut)_4_ in 0.5 mL of dry EtOH and 0.15 mL of glacial acetic acid combined with both 52 mg of Li(CH_3_COO) and 40 mg of a structure-directing agent (poly(ethylene-*co*-butylene)-block-poly(ethylene oxide), KLE)) dissolved in 0.5 mL of dry ethanol and 1.0 mL of 2-methoxyethanol. Hermawan et al. [68] synthesized porous LTO samples by sol–gel method using an eggshell membrane as a soft template. Lithium nitrate and titanium tetrachloride as precursors were dissolved separately in DI water and concentrated HCl (37%), respectively, and then added by ammonia solution to form colloidal particles, which were attached to the as-prepared eggshell membrane immersed into the solution for 12 h. Hao et al. [397] applied a solution-based method starting from alkoxide precursors of lithium and titanium to obtain atomic-level mixing from which to synthesize LTO. Nanocrystals (with a high surface area of 27 m^2^ g^−1^ and concomitant small particle size of 58 nm) were formed from a templated synthesis using this precursor solution and carbon (acetylene) black (composed of ~70 nm particles) (Figure 15). LTO templated electrodes demonstrated reversible cycling storage ~160 mAh g^−1^ at a 10C rate and maintaining a capacity >150 mAh g^−1^ even at 100C.

Polyisoprene-block-poly(ethylene oxide) (PI-b-PEO) with an sp^2^-hybridized carbon-containing hydrophobic block was employed as a structure-directing agent as well as a carbon source for the synthesis of a mesostructured LTO/C composite [155]. The inorganic precursor solution was prepared by mixing 1.287 mL of titanium tetra-isopropoxide and 0.181g of lithium ethoxide (3.48 mL of 1 mol L^−1^ LiOC_2_H_5_ in tetrahydrofuran stock solution) and added to 0.39 g of oxalic acid. The LTO/C composite was formed after heat-treatment of the mixture at 700 °C under N_2_ and held for 2 h. Xia and co-workers [283] used butterfly wings as biotemplates, which resulted in a 3D porous architecture with a periodic network structure formed by the interconnected “ridge” and “strut”. Liu et al. [159] adopted a template-hydrolyzation plus glucose decomposition method to prepare LTO/C nanotube arrays grown directly from TiO_2_ nanotubes on stainless-steel foil. The precursor of TiO_2_ nanotube arrays was fabricated by a one-step deposition and in situ etching solution route. In a typical synthesis, the as-synthesized ZnO nanorod arrays on stainless steel foil were immersed in an aqueous solution consisting of 0.075 mol L^−1^ (NH_4_)_2_TiF_6_ and 0.2 mol L^−1^ H_3_BO_3_ at room temperature for 40 min. In this solution, (NH_4_)_2_TiF_6_ hydrolyzed to TiO_2_ on the surface of individual ZnO nanorods template, while ZnO dissolved simultaneously in the solution with acids produced by (NH_4_)_2_TiF_6_ hydrolysis. The final LTO product obtained by a chemical lithiation route possesses much shorter ion diffusion paths because the electrolyte both inside and outside the nanotubes could simultaneously diffuse leading to accelerated reaction kinetics. The ZnO nanorod array was also used as the sacrificial template for the fabrication of LTO nanotube arrays (TiCl_4_ and H_2_O as the Ti and O precursors) via an ADL-lithiation process using 100 mL of 3 mol L^−1^ LiOH aqueous solution kept at 80 °C for 1 h [399]. The ZnO template was simultaneously etched by the LiOH during the lithiation process. A high specific capacity of 130 mAh g^−1^ was delivered at 40C after 1600 cycles. In a typical synthesis of nanotube arrays, the ZnO nanorods template was fabricated via a chemical bath deposition consisting of a reaction solution Zn(NO_3_)_2_ and ammonia in DI water kept at 95 °C for 5 h. Singh et al. [400] proposed a facile and low-cost templating method using carbonate salts creating 3D interconnected ionic pathways that improve the ionic charge transport without compromising the electrode density significantly. The method was demonstrated for C/Li_4_Ti_5_O_12_ electrode material resulting in excellent capacity retention reaching ~90% at 5C and ~50% at 200C rate combined with high active material electrode densities around 1.45 g cm^3^. Electrochemical performances of nanostructured LTO materials synthesized by the templating method are listed in Table 13.

### 2.14. Spray Drying

**Figure 16:**
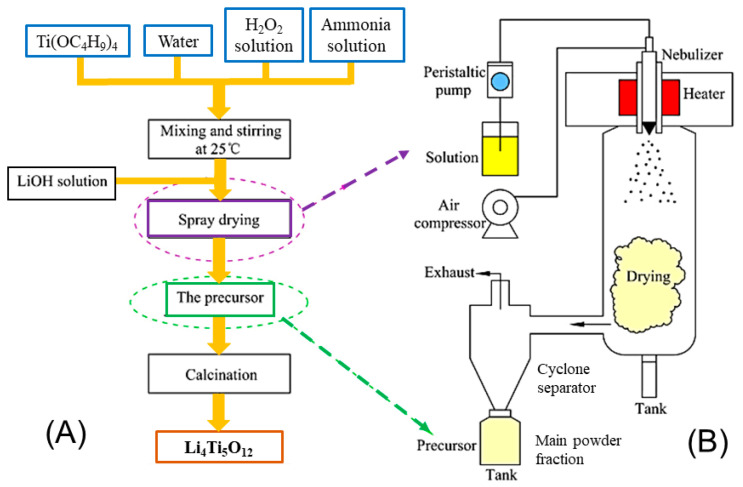
(**A**) Chart of the LTO spray-drying process. (**B**) Spray drying apparatus. The process consists of a sequence of four phases: (1) Atomization of the liquid to be dried (slurry), (2) Contact between hot gas and nebulized liquid, (3) Evaporation of solvent, and (4) Separation of the dried product from the drying medium. Reproduced from [403]. Copyright 2012 Elsevier.

The spray drying (SD) method is particularly attractive because it has good reproducibility and is easily up-scalable to the industrial level [401]. To demonstrate the scalability of the template-based spray drying approach, Nowack et al. [402] fabricated LTO particles in a pilot-scale spray dryer capable of a production rate of 4 kg h^−1^. SDM is used to produce powders from aqueous and/or organic-aqueous solutions or suspensions. The most common solvent for solutions is water. Alcohols are also used, either pure or mixed with water. SD involves the atomization of a liquid in a chamber, in which a hot gas recirculates. As the nebulized droplets are exposed to a very large surface with the drying fluid, the drying reaction is almost instantaneous. Two of the important parameters to control the process are the output temperature and the relative humidity after the cyclone collector (Figure 16) [403]. Numerous works report the SD synthesis of LTO [111,112,404,405,406,407,408,409,410,411,412,413,414,415,416,417,418,419,420,421,422,423,424,425,426,427,428,429]. In an early work, Hsiao et al. [409] synthesized porous and dense LTO powders by spray drying followed by solid-state calcination. The spray drying precursor was slurries of LiOH, anatase TiO_2_ (21 nm particle size), and 5 wt.% (relative to TiO_2_) BYK-190 dispersant (solution of a high molecular weight block copolymer with pigment affinic groups) in DI water. After ball milling at a speed of 300 rpm for 8 h, the homogeneous slurries were atomized at 250 °C using a two-fluid nozzle with an atomizing pressure of 3 kg cm^−2^. The final crystalline product was obtained by solid-state calcination in air at 850 °C for 8 h.

In a typical synthesis, LTO powders are prepared by heating at 850 °C for 2 h under air a precursor obtained by spray-drying an aqueous suspension of TiO_2_ and LiOH∙H_2_O taking a stoichiometric ratio Li:Ti = 4:5. The spray-drying experiments are conducted using a rotating injector with inlet temperature 190 °C, outlet temperature 110 °C, feed rate 25 mL min^−1^ and air pressure of 3 bars [430]. Yuan et al. [431] constructed three-dimensional (3D) crumpled graphene sheets wrapped in nano-Li_4_Ti_5_O_12_ (LTO@GS) composites using a one-pot spray-drying assisted solid-phase reaction method with anatase TiO_2_ as Ti source and LiNO_3_ (3% mole excess) as Li source. The GO suspension (1 mg L^−1^) with a mass ratio of LTO to GO of 80:20 was sonicated for 1 h and spray-dried at 200 °C. The specific capacitance of this LTO@GS composite as an anode in a hybrid battery-capacitor BatCap system still retains 90% of the initial value after 20,000 cycles. The LTO@GS//AC BatCap system showed a specific energy of 29.2 Wh kg^−1^ at a power density of 58.4 W kg^−1^. Wu et al. [432] developed a low-cost procedure for the synthesis of Cr-doped LTO microspheres (1–5 µm in diameter, BET SSA of 9.39 m^2^ g^−1^), which involves a versatile solution-based method, spray drying, which enables ion-level mixing of liquid components. An ammonia solution was used to maintain a pH value of ~10. Zhang et al. [420] synthesized a Li_4_Ti_5_O_12_/rGO composite by spray-drying and annealing processes using aqueous suspensions of LTO and GO vigorously stirred together to form a slurry with LTO:GO = 50:1 by weight. De-ionized water was added to the mixture to adjust the solid content to 2 wt.%. Subsequently, the slurry was stirred, and ultrasonically exposed for 30 min, which was then spray dried at 200 °C to form a solid LTO/GO composite. The composite was heated to 400 °C at a rate of 10 °C·min^−1^ and sintered at that temperature for 5 h under an Ar atmosphere to form the active LTO/rGO anode material. The LTO primary particles aggregated in micro-sized spherical secondary particles, which were wrapped homogeneously and loosely with a rGO network. This anode material showed excellent rate performance and high cyclic stability; after 300 cycles at 20C, the specific capacity was still 100 mAh g^−1^. Jia et al. [433] synthesized nanostructured Li_4_Ti_5_O_12_/carbon nanotube (LTO/CNT) composite particles using an aerosol spray drying process followed by thermal annealing. The electrodes delivered a high reversible capacity of 108 mAh g^−1^ at an extremely high rate of 100C. Moreover, ultralong cycling stability was attained through 8000 rapid charge-discharge cycles with 89% capacity retention. Mesoporous spherical LTO/TiO_2_ nanocomposite was prepared by aerosol spraying hydrolysis using TBT (1.0 mL), LiNO_3_ (4/5 by Li/Ti molar ratio), triblock copolymer P123 (0.25 g), and ice-acetic acid (HAc, 0.5 mL) dissolved in ethanol (20 mL). The solution was transferred into a medical-use ultrasonic humidifier (1.7 MHz, 35 W) for the aerosol-spraying process. The final product was obtained by calcining its precursor at 600 °C for 8 h in air. The mesoporous composite had a specific surface area of 43.18 m^2^ g^−1^, an average pore diameter at ca. 2.4 nm, and delivered a capacity of 111 mAh g^−1^ at a 20C rate [434]. Hole-rich Li_4_Ti_5_O_12_/CNTs composites were synthesized by spray drying of titanium peroxide solution and carbon nanotubes as additives in the precursor solution, subsequently followed by calcination at high temperature in air [423]. The SD solution was formed by Li_2_CO_3_ and titanium(IV) isopropoxide (TIP) in stoichiometric amounts (molar ratio of Ti/Li = 0.86) dissolved in ethanol and further added to a second solution formed by deionized water containing oxalic acid (OA) with mole ratios TIPP:OA of 3:1. After mixing with CNTs and ultrasonic sonication for 5 h, the obtained sol was subsequently spray-dried by hot air at 230 °C, followed by sintering at 800 °C for 12 h in air. The as-synthesized hole-rich LTO hybrid exhibited an initial discharge capacity of 145 mAh g^−1^ at a 2C rate and decreased gradually with continuous cycling, retaining 138 mAh g^−1^ after 200 cycles.

The preparation of the spray-drying aqueous solution proposed by Park et al. [435] involved a mixture of ternary precursor materials: Li_2_CO_3_ and rutile-structured TiO_2_ (~21 nm) with Li:Ti molar ratio 4.1:5 were dissolved into distilled water with glucose (C_6_H_12_O_6_). A polymeric dispersant, i.e., gum Arabic, was mixed into the solution via an ultrasonic bath for 30 min to form a relatively homogenous mixture. The dispersed solution (~15 mL) was sprayed onto a titanium plate, which maintained a uniform surface temperature of 150 °C and evaporated the solvent instantaneously via a controllable hot plate (PC-420D-230, Corning) and a compressor (Monster comp001, China). The Li_4_Ti_5_O_12_/C composite powders were obtained by a spray-drying process, collected, and calcined at 800 °C for 2 h in a furnace purged with Ar gas.

To overcome the drawbacks of the spray drying method, i.e., poor dispersion, insufficient mixing, and too large particle size of the starting precursors, unreacted TiO_2_ or secondary phase of Li_2_TiO_3_, Chang-Jian et al. [42] succeeded in synthesizing pure LTO by controlling the Li and Ti ratios in the range of 0.800–0.900 or changing the parameters of thermal annealing through the spray-drying method. The pilot scale solution was composed of 411.34 g of TiO_2_ powder, 152.11 g of Li_2_CO_3_, and 60 mL of polyvinyl alcohol (PVA) solution (10%) added to 1360 mL of DI water. Lee et al. [436] fabricated Li_4_Ti_5_O_12_/pristine multiwalled carbon nanotube (LTO/P-MWCNT) composites with high-rate capability and compared, two additional LTO composites prepared by using oxidized MWCNTs and surfactant-treated MWCNTs through a similar spray-drying process. The hybrid supercapacitor composed of an LTO/P-MWCNT anode and an activated carbon cathode delivers an energy density of 70.9 Wh kg^−1^ at a power density of 0.03 kW kg^−1^ and a maximum power density of 21.8 kW kg^−1^ is achieved at an energy density of 24.3 Wh kg^−1^. Chien et al. [437] used two Ti sources, i.e., 100% anatase TiO_2_ and P25 TiO_2_ (80% anatase + 20% rutile), and LiOH as Li source to synthesize LTO via spray-drying method with the air inlet and outlet temperature of the spray-dryer were 175 and 113 °C, respectively. The electrochemical performance of the LTO material tested at 0.1C/0.1C prepared from P25 TiO_2_ (specific capacity of 169 mAh g^−1^) was superior to that of the LTO prepared from 100% anatase TiO_2_ (165 mAh g^−1^). Hsieh et al. [438] evaluated the influence of Li addition on the charge/discharge behavior of LTO. Other LTO products were prepared by spray technique using a mixture of TIP, LiNO_3_, and isopropanol [439,440]. 

Nanoscale and highly porous LTO powders (with 200 nm particle size) were synthesized by SD process from Li_2_CO_3_ and nanocrystalline anatase type TiO_2_ (72.75 wt.%, 30−40 nm) as precursors, in aqueous solution containing 3 wt.% of dispersant ammonium polycarboxylate. The homogeneous slurry obtained was spray-dried with the inlet and outlet temperatures of 200 and 100 °C, respectively, and heat treated at 850 °C for 5 h [404]. The influences of Li/Ti atomic ratios (0.784, 0.800, 0.816, and 0.832) on the performance of LTO synthesized through SDM were investigated by He et al. [408]. The results indicate that when the spray-drying precursors at the Li/Ti molar ratio of 0.816 are calcined at 700 °C for 16 h in air, a pure LTO phase (200 nm primary particle size) with a lithium-excess composition is obtained, which shows the best electrochemical properties, i.e., a specific discharge capacity of 135 mAh g^−1^ after 100 cycles at 5C rate. Ren et al. [416] prepared uniform LTO microspheres with high tap density by a newly designed industrial spray-drying approach at 220 °C. LTO sample obtained with a Li/Ti molar ratio of 0.75 and sintered at 850 °C exhibits the highest specific capacity of 173 mAh g^−1^ after 400 cycles at 1C rate. Several workers provided proof that Li addition influences the charge-discharge behavior of spray-dried LTO [338,339,340]. Table 14 summarizes the electrochemical performances of nanostructured LTO materials synthesized by the spray-drying method.

The overall reversible capacity in the voltage range of 2.5–0.01 V vs. Li^+^/Li is limited by the vacant tetrahedral (8a) and octahedral (16c) sites. The decrease in the capacity ratio from the second plateau (1.0–0.01 V) to the first plateau (2.5–1.0 V) with the *R* = *I*_(311)_/*I*_(400)_ value proves that the ratio of site vacancy greatly affects the capacity contribution. At high *C* rates, the tetrahedral (8a) sites exhibit a better accessibility to accommodate Li-ions during the intercalation process, compared with the accessibility of octahedral (16c) sites. 

### 2.15. Spray Pyrolysis Method

In conventional spray pyrolysis, the solution is atomized into a hot wall reactor where the aerosol droplets undergo evaporation and solute concentration within the droplet, drying, and thermolysis of the precipitate particle at higher temperature to form a microporous particle, and eventually a dense one by sintering [439,440]. Ceramic nanoparticles with high purity and relatively narrow size distribution can be prepared (see Zhu et al. [441] for experimental details). Spray pyrolysis reactors are routinely used in industry to make a variety of oxides (SiO_2_, TiO_2_, Al_2_O_3_, etc.). Combustion of liquid precursor droplets is currently used in the synthesis of mixed oxide powders by the so-called flame spray pyrolysis (FSP) process (see Figure 1a). The FSP is capable of producing mixed metal oxide powders in the size range of 1–200 nm from low-cost precursors with production rates up to 250 g h^−1^ [442]. This method has been successfully applied for the synthesis of LTO [115,117,443,444]. In 2005, Doi and co-authors [115] first reported the preparation of uniform nanosized LTO particles using an electrospray deposition method. The spinel-LTO particles have a fairly-uniform particle size of ~12 nm with a distinct crystal structure.

**Figure 17:**
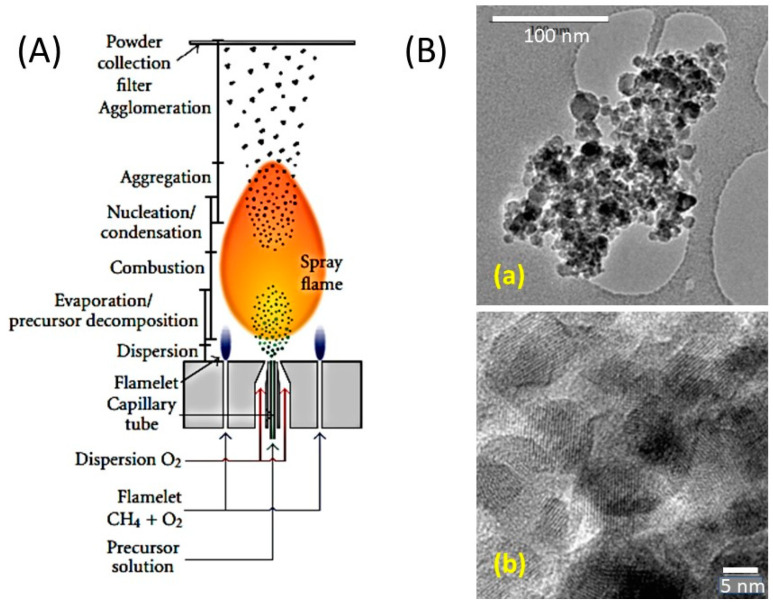
(**A**) Scheme of a flame spray pyrolysis apparatus. The main elements are the atomizer, preheater, non-premixed slot burner, and collection system. Ultrasonic transducers operating at 1.7 MHz provide the driving force for atomization of the precursor inside the vessel, generating a fine aerosol with a number-based mean diameter of 3.5 μm. A flow of carrier gas comprising air enriched with oxygen transported the solution droplets at a rate of approximately 1 g droplets min^−1^. The flame synthesis temperature (~1700 °C) is adjusted by the gas mixture CH_4_/N_2_ used as fuel. (**B**) morphology of LTO products: (**a**) TEM image of single aggregate, (**b**) High-resolution TEM image. Reproduced with permission from [444]. Karhunen et al. under the Creative Commons Attribution License.

Bresser et al. [117] fabricated LTO nanoparticles using the FSP method with an average diameter of approximately 20–30 nm and a specific surface area of 91.7 m^2^ g^−1^. However, phase impurities such as rutile, anatase TiO_2_, and Li_3_Ti_3_O_7_ were detected by means of XRD analysis. The advantage of nanoparticles was electrochemically illustrated at a high rate of 100C providing a discharge capacity of 72 mAh g^−1^. In the FSP process used by Karhunen et al. [444], LiOAc and TIP raw materials were dissolved with a stoichiometric ratio of (4:5) in an equal volume mixture of toluene and 2-ethyl hexanoic acid. The resulting solution with a total metal concentration of 0.5 mol L^−1^ and the premixed methane-oxygen flamelet ignited the aerosolized precursor solution at temperatures in excess of 1700 °C. LTO particles with a mean size of 80 nm were formed (Figure 17). Ernst and co-authors [75] used the FSP method to synthesize spinel-LTO with primary crystallite sizes of 7–30 nm and a high-temperature stability from lithium tert-butoxide dissolved in tetrahydrofuran (THF) and titanium (IV) isopropoxide dissolved in xylene with Li/Ti ratios in the range of 0.5–1.0. It has been found that the FSP process optimization could be used to further remove impurities. Moreover, FSP-prepared LTO nanoparticles showed good sintering stability at elevated temperatures and the scalability of the process. Waser et al. [445] reported the design of a scalable process for size-controlled flame spray synthesis of LTO particles. The role of air entrainment (AE) during aerosol synthesis was quantitively analyzed by computational fluid dynamics and temperatures were verified by FTIR spectroscopy. The LTO precursor solution (1.0 mol L^−1^ total metal concentration) consisted of Li-acetylacetonate and Ti(IV)-isopropoxide in a stoichiometric molar ratio of Li:Ti = 4:5, diluted with a 1:3 (by volume) mixture of 2-ethylhexanoic acid and xylene solvents. For AE > 50 L min^−1^, more than 95% pure LTO was obtained with only minor impurities of anatase and rutile TiO_2_. This process appears superior to the as-prepared LTO obtained by open FSP [75] with 87 wt.% LTO content and *d*_BET_ of 20 nm and FSP-made nano LTO by Bresser et al. [117], but similar to that by Karhunen et al. [444].

Gockeln et al. [77] fabricated ultrafine LTO/C composite using combined double flame spray pyrolysis and pressure-based lamination technique. Within the flame spray, the nanostructured LTO particles are formed at temperatures of ≥2000 °C upon nucleation, surface growth, coagulation, and coalescence. To synthesize carbon from xylene, the N_2_ gas was chosen as the dispersant (1.5 L min^−1^, nozzle pressure drop 1.5 bar) and the support flame was maintained at under-ventilated fuel-to-air ratio conditions (CH_4_ of 2.5 mL min^−1^ and O_2_ of 0.5 mL min^−1^) resulting in a strong sooting flame. Ju et al. [72,73,74] controlled the mean size of the LTO powders by changing the concentrations of the spray solution. The precursor solution was prepared by dissolving LiNO_3_ and Ti[OCH(CH_3_)_2_]_4_ in distilled water using a small amount of nitric acid. The precursor powders had fine size, narrow size distribution, dense inner structure, and homogeneous composition when the flow rate of the carrier gas and the preparation temperature were 10 L min^−1^ and 800 °C, respectively. high discharge capacities were delivered by particles fabricated with high concentrations above 0.5 mol L^−1^. In another work, the same group of researchers studied the effects of drying control chemical additives on the properties of LTO using a solution containing dimethylacetamide (DMA) as a drying control chemical additive and citric acid (CA) and ethylene glycol (EG) as organic additives [446]. It was concluded that the addition of DMA to the polymeric spray solutions containing CA and EG helps in the effective control of the morphology of as-sprayed LTO powders. Meierhofer et al. [76] studied the LTO phase purity anode using FSP via precursors dissolved in five different organic solvents, i.e., ethanol (EtOH), benzyl alcohol (BnOH), tetrahydrofuran (THF), xylene, and ethylhexanoic acid (EHA). The effect of precursor and solvent parameters such as chemical reactivity, boiling point, and combustion enthalpy on the particle formation either via gas-to-particle (evaporation/nucleation/growth) or via droplet-to-particle (precipitation/incomplete evaporation) was discussed. The presence of carboxylic acid in the precursor solution resulted in pure (>95 mass %) and homogeneous LTO nanoparticles of size 4−9 nm, for two reasons: (1) stabilization of water-sensitive metal alkoxides precursor and (2) formation of volatile carboxylates from lithium nitrate. In contrast, the absence of carboxylic acids resulted in larger inhomogeneous crystalline TiO_2_ particles with a significant reduction of LTO content as low as ~34 mass %. LTO thin films (400 nm thick) were prepared on quartz and gold substrates by the spray pyrolysis method by using lithium-acetyl acetonate (Li-acac) and titanil-acetil acetonate (TiO(acac)_4_) with di-methyl formamide (DMF) solvent as starting materials [447]. Yamada et al. [448] prepared spray-pyrolyzed C/Li_4_Ti_5_O_12_ powders using LiNO_3_ and titanium isoproxide dissolved in an aqueous solution with a different organic acid such as lactic, malic, and citric acid in 0.1–0.4 mol L^−1^ concentration. The use of lactic acid as a carbon source was most effective for their improvement. Kim and Kang [449] prepared amorphous LTO particles using inexpensive lithium nitrate (LNT) and TTIP dissolved in a mixture of ethyl alcohol and distilled water (volumetric ratio, 3/7) with a total metal concentration of 0.5 mol L^−1^. Du et al. [450] synthesized crystalline LTO particles (10–30 nm) using LiOAc and TBT dissolved in ethanol to obtain a 0.5 mol L^−1^ solution. The molecular-level uniformity of the precursor allows for the synthesis of LTO with a significantly shorter heat treatment compared to conventional solid-state reaction, which in turn saves energy during large-scale production. Spray-pyrolyzed LTO delivered a discharge capacity of 146 mAh g^−1^ at a 10C rate for up to 500 cycles. Electrochemical performances of nanostructured LTO materials synthesized by the spray-pyrolysis technique are listed in Table 15.

### 2.16. Sonochemical Process

The sonochemical process is an energy-miser route, which yielded nanoscale homogeneous spinel LTO product. Generally, the estimated temperature and pressure in the liquid zone around the collapsing bubble generated in a sonochemical reactor at a driving frequency of 20 kHz with an input power of 179 W are approximately 1000 °C and 500 atm, respectively. Lee et al. [104] synthesized homogeneous LTO nanoparticles using TiO_2_ nanoparticles coated with a LiOH thin coating (2–5 nm) via a sonochemical method, operated at 20 kHz and 220 W for 20 min. The resulting LTO nanoparticles thermally treated at 500 °C for 1 h, have an average grain size of about 30–40 nm with excellent phase purity. Kim et al. [454] reported the growth of LTO ceramic from hydrogen titanate nanowires as precursors implemented by using TiO_2_ having a size of 60 nm and NaOH, and performing synthesis at 70 °C for 6 h with a sonochemical method. Ghosh et al. [105] reported the sonochemical synthesis carried out using commercial precursors LiOH·H_2_O and TiO_2_ (spherical particles of diameter 135 nm) mixed in ethyl alcohol dispersive media. The ultrasonic process was conducted in the following three steps: (1) first, TiO_2_ was dispersed in ethyl alcohol using an ultrasonicator for 20 min, (2) LiOH·H_2_O was dissolved into this mixture with continuous stirring for 10 min, (3) this precursor mixture was kept under the probe sonicator inside an ice bath to perform sonication experiment at room temperature. The sonication operation was carried out by applying sonic wave for 10–40 min at a pulse rate of 2 s with a pause of 1 s using a Ti probe (Φ = 13 mm) operating at 500 W (with amplitude of 35%, 20 kHz). The LTO nanomaterial investigated by Ni et al. [106] was prepared via an ultrasonic-assisted liquid deposition method. Typically, 3.4 g titanium tetrabutoxide (C_16_H_36_O_4_Ti, TBOT) was dispersed in 80 mL ethanol with sonication for 1 h. A mix solution of distilled water (2 mL), ethanol (18 mL) and 0.56 g CH_3_COOLi was added drop-wise into the above suspension with further sonication for 1 h. The precursor was calcined at 400 °C for 4 h, and subsequently sintered at 750 °C for another 10 h under N_2_ to obtain well-dispersed nanoparticles (~100 nm in calculated average size). Jin and co-workers [107] used a sonication process for 2 h to disperse TiO_2_ nanopowders in DI water before mixing them with LiOH, which yields white-colored suspensions. Mao et al. [109] reported a one-step continuous synthesis of spherical LTO/graphene composites through direct aerosolization of a GO suspension mixed with LiOH and titanium(IV) bis(ammonium lactato) dihydroxide as Li and Ti precursors. The mixed solution was nebulized by an ultrasonic nebulizer (2.4 MHz) to form aerosol particles, which were carried by argon gas through a horizontal tube furnace preheated at a desired temperature (750 °C). The TEM image also reveals the LTO nanocrystals are closely packed on the graphene surface, with a diameter of a few nanometers (5–10 nm). The LTO/CG composite, as an anode in LIBs, cycled at 1.25 A g^−1^ shows a capacity of ca. 100 mAh g^−1^ and retaining 88 mAh g^−1^ after 5000 cycles.

### 2.17. Microwave Synthesis

Microwave (MW) synthesis has been known to provide a fast route for reactions in the solid state, and it often occurs at lower temperatures than the conventional synthesis process. The higher reaction and cooling rates during microwave irradiation make the formation of metastable phases possible. The pioneering research on microwave synthesis of titanates was reported by Bhat and coworkers, and they have obtained lithium lanthanum titanate from metal oxides [455]. Due to the high penetration depths of the microwave, it can be uniformly and rapidly absorbed by LTO, and thus the high temperature needed in a solid-state reaction can be obtained within several minutes [216,351,396,456,457]. Yang et al. [458] implemented a hybrid microwave solid-state synthesis to prepare LTO grains with an average size of ~1 µm starting using a mixture of Li_2_CO_3_ and TiO_2_, as reactants, finely grounded and pressed into pellet with a pressure of 9 MPa and placed in a domestic microwave oven working at the frequency of 2.45 GHz and maximum power of 800 W. Li et al. [456] fabricated regular sphere-like LTO nanoparticles (average size of 40–50 nm) using microwave irradiation at 700 W for 15 min. However, the morphology of the product is also not easy to control in solid-state reactions. The present combination of microwave-assisted hydrothermal and microwave post-annealing methods is a great time-saving method to produce nanomaterials with controlled morphologies. Chou et al. [370] implemented a rapid method for the preparation of microspheres composed of nanoflakes within 1 h by a combination of a microwave-assisted hydrothermal method and a microwave post-annealing process. A polytetrafluoroethylene (Teflon)-lined autoclave was put under microwave-assisted hydrothermal treatment at 150 °C for 15 min to yield the as-prepared LTO precursor, followed by washing three times in deionized water and three times in acetone and finally annealed by microwave heating in air for 20 min.

### 2.18. Rheological Phase Reaction

The rheological phase reaction is an efficient soft chemistry method to prepare electrode materials. Contrary to the conventional SSR, this method provides homogeneous [459]. A typical rheological phase reaction involves five steps: dissolution, diffusion, reaction, nucleation, and growth. The solid reactants are fully mixed by adding a proper amount of deionized water or other solvents to produce a solid–liquid rheological mixture, in which the solid particles are uniformly distributed in the liquid substance. Yin et al. [113] have reported the synthesis of individual particle Li_4_Ti_5_O_12_ (~140 nm) with a high-rate performance by a modified rheological phase method, which occurs as follows. Stoichiometric amounts of lithium acetate dihydrate (CH_3_COOLi∙2H_2_O) and tetra-*n*-butyl titanate (Ti(OC_4_H_9_)_4_) were sufficiently ground in the mortar at room temperature for half an hour. The hydrolysis of Ti(OC_4_H_9_)_4_ started readily during the grinding process, accompanied by evaporation of part of acetic acid and butanol. As the reaction proceeded, the mixture became mushy and underwent gradual changes in color from colorless to white. Then a trace amount of deionized water was added to accelerate the hydrolysis of Ti(OC_4_H_9_)_4_ and reduce the conglutination resistance during the grinding. After sufficiently grinding for a few minutes, the white rheological phase body was transferred into a container and the container was sealed in a stainless autoclave at 110 °C for 12 h. Subsequently, the ultrafine white powder lithium titanate was obtained by calcination at 800 °C for 12 h in air. Liu and co-workers [460] synthesized LTO particles (average size of 2.1 μm with a narrow size distribution) by a simple rheological phase method using polyvinylbutyral (PVB) as both template and carbon source. Stoichiometric amounts of Li_2_CO_3_ and TiO_2_ were blended homogeneously and then added into the PVB/ethanol (9.2 g L^−1^) solution to obtain a solid–liquid rheological mixture. After continuous magnetic stirring of the mixture for 4 h, drying at 80 °C for 6 h in a vacuum oven to eliminate ethanol adequately, the sample was sintered at 800 °C for 15 h under argon flow. Wang et al. [461] synthesized an LTO/C composite (particle size of ~500 nm) by starch-sol-assisted rheological phase method using anatase TiO_2_ and LiOH·H_2_O as raw materials. The electrochemical tests show that LTO/C presents a capacity retention of 87% (500 cycles at 1C) and 73.0% (2000 cycles at 20C).

### 2.19. Electrospinning Method

**Figure 18:**
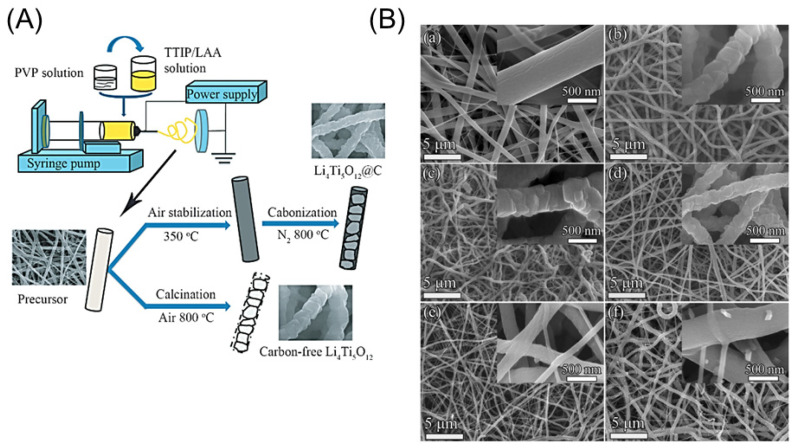
(**A**) Electrospinning synthesis route of pristine LTO and LTO/C fibers. (**B**) SEM images of (**a**) as-spun fibrous precursor, (**b**) LTO fibers, (**c**) 0.85 wt.%C/LTO, (**d**) 0.95 wt.% C/LTO, (**e**) 6.02 wt.% C/LTO, and (**f**) 9.90 wt.% C/LTO fibers. The insets show the corresponding high-magnification images. Reproduced with permission from [140]. Copyright 2014 Wiley.

Electrospinning is a synthesis method used to generate micro- or nanofibers from a polymer solution using a high-voltage power supply. In this technique, a precursor solution is loaded into a syringe and is subjected to a high voltage (1–20 kV), which lets the solution be extruded from a nozzle forming a jet. Fibers are formed and deposited on the collector by the jet during drying. Electrospinning creates a high surface area to volume ratios needed for most catalyst systems depending on operation parameters (voltage, flow rate, distance between tip and collector, concentration, and viscosity of the solution). The scheme of the electrospinning synthesis route is shown in Figure 1a. Electrospinning has been successfully employed to prepare 3D net architectures of spinel LTO nanofibers [95,462,463]. In a typical experiment, the electrospun solution consists of a mixture of TIP, lithium acetylacetonate (LiAAc), and polyvinylpyrrolidone (PVP) in isopropyl alcohol. The electrospinning process is conducted in air. A dc voltage of 1.2 kV is applied between a gauge needle and a stainless-steel collector electrode. Finally, PVP is removed from nanofibers by treating them in air at 500 °C for 1 h. Guo and co-workers [464] fabricated two types of LTO/C composites through the electrospinning method (high voltage of 10 kV) of two different solutions: (i) the first composite consists of LTO nanoparticles and aggregates coated by carbon and connected by carbon nanofibers using a solution prepared by adding 0.8 g polyacrylonitrile (PAN, *M*_w_ =150,000) and 2 g nano-LTO (primary particle size of 20–60 nm) into 17.2 g dimethylformamide (DMF) solvent, and the second composite is constructed solely by LTO/C fibers using a mixture of 1.52 g tetrabutyl titanate, 0.23 g lithium acetate and 1 g PVP into 18 g isopropyl alcohol solvent. Electrochemical tests show specific capacities of 115 and 120 mAh g^−1^ at a 2C rate for LTO/C particles/fibers and LTO/C fibers, respectively. LTO@C nanofibers containing tiny nanoparticles were obtained by electrospinning based on an adjustable solution strategy [132]. Typically, 4 mmol of Li(CH_3_COO)_2_·H_2_O, 5 mmol of titanium(IV) oxyacetylacetonate, and 1.4 g poly(vinylpyrrolidone) (PVP, *M*_w_ = 1,300,000) were dissolved into 10 mL ethanol. After vigorous stirring for 12 h, the homogenous precursor solution was poured into a syringe connected to a plastic needle, while a copper wire attached to a high-voltage generator was placed in the solution. A tension of 20 kV was applied between the needle and the Al foil target used for collection. Acetate and acetylacetonate were completely decomposed during the high-temperature process, while PVP gel was carbonized into conductive carbon nanofibers. The whole synthetic route is a simple, low-cost, and high-yield process. Finally, the as-collected electrospun fibers were calcined at 400 °C for 2 h and 800 °C for 5 h under an Ar atmosphere to obtain LTO@C hierarchical nanofibers. Wang et al. [465] investigated the growth of LTO nanofibers (average diameter of 230 nm) via thermal treating electrospun precursor fibers at 700 °C. Ji et al. [466] reported the electrospinning preparation of one-dimensional Ce^3+^-doped Li_4_Ti_5_O_12_ sub-micron belts with a width of approximately 500 nm and a thickness of about 200 nm. Li et al. [467] used a precursor solution of lithium acetate, tetraisopropyl titanate, acetic acid, and different doses of PVP. The solution was loaded into a plastic syringe and subjected to electrospinning. The flow rate was 1.5 mL h^−1^ and the distance between the tip of the needle and collector was 10 cm, the collector drum speed was 800~1000 rpm, and the experiment voltage was 12 kV. It was demonstrated that the viscosity of LTO precursor solution (from 11 to 117 cP) dramatically affects the morphology of LTO submicron fiber (from 0.17 to 0.58 µm diameter). Jo et al. [137] reported the synthesis of LTO nanofibers with uniform diameter <300 nm, a specific surface area of 9.7 m^2^ g^−1^, and an average pore size of ~2 nm through electrospinning. Xu et al. [140] prepared electrospun conformal Li_4_Ti_5_O_12_/C fibers using an as-spun fibrous precursor containing PVP, TIP, and LiAAc stabilized at 350 °C in air and then thermally treated at 800 °C in an N_2_ atmosphere. Long and continuous fibers with smooth surfaces and an average diameter of approximately 500 nm were observed (Figure 18).

The LTO electrospun nanofibers, with an average diameter of ~250 nm, were prepared by Park et al. [468] from the solution precursor of 1.42 g titanium butoxide mixed with a solution containing 2 mL ethanol and 1.25 mL acetic acid, 0.354 g lithium acetylacetonate and a polymeric solution consisting of 3 mL ethanol and 0.5 g PVP. The feeding rate was set at 0.2 mL h^−1^ and a high voltage of ~20 kV was exerted to collect nanofibers. Chen and co-workers [469] prepared electrospun LTO fibers using acetic acid, ethanol, butyl titanate, lithium acetate, and PVP (K90) as the raw materials according to the ratio 4.28:14.19:4.16:1:2.45. The electrospinning process was carried out ay 19–21 °C under 20 kV dc high voltage. Zou et al. [470] reported the preparation of electrospun Li_3.9_Cr_0.3_Ti_4.8_O_12_ nanofibers as anode material for high-rate and low-temperature LIBs. The precursor solution was prepared by dissolving 6.808 g tetrabutyl titanate, 0.507 g chromium nitrate, 1.742 g lithium acetate, and 2.500 g polyvinyl pyrrolidone (PVP, *M*_w_ = 1,300,000) in a mixed solution with 30 mL ethanol and 10 mL acetic acid. The dc voltage of 20 kV was loaded on the needles, and the distance between the needle and a collector was 15 cm. The spinning speed was controlled at 2.0 mL h^−1^ using an auto-syringe pump. The as-spun nanofibers (heat-treated at 700 °C for 3 h in air) exhibited excellent lithium storage performance: 140 mAh g^−1^ at 10C and 91 mAh g^−1^ at 50C rates at room temperature, 100 mAh g^−1^ at 1 C rate at −20 °C. Electrospun bare LTO and Ag-LTO nanofibers were synthesized using 1.48 mL of titanium(Ⅳ) isopropoxide and 0.413 g of lithium acetate dissolved over 1 h in 10 mL of ethanol and 7 mL of acetic acid and added with 0.8 g PVP [471]. The randomly aligned nanofibers have average diameters of 50–200 nm and lengths extending to several tens of micrometers. At the highest rate of 30 C, the specific capacity of the Ag–LTO nanofibers is 107 mAh·g^−1^, which is significantly higher than that of the bare LTO nanofibers (82.2 mAh·g^−1^). LTO/Ag composite nanobelts with an average width of ca. 1.12 µm were synthesized by Li et al. [144]. These composites showed good rate capability (specific capacity of 132 mAh g^−1^ at 15C rate) and cycling stability (172 mAh g^−1^ after 100 cycles at 0.2C rate). Castano et al. [96] synthesized LTO fibers via a two-step method. First, a precursor solution containing a spinnable polymer, and titanium and lithium salts, which were dissolved in a mixture of solvents, was subjected to a process of electrospinning. Isopropyl alcohol was used as a solvent for PVP as well as for lithium acetate and titanium butoxide precursor salts. Then, the obtained fibers were calcined at temperatures between 650 and 850 °C for 7–10 h in air or argon. Zhang et al. [472] synthesized a self-standing nonwoven flexible Li_4_Ti_5_O_12_/carbon nanofiber composite (denoted LTO/CNF) by using a facile method involving the electrospinning fabrication of CNFs and chemical deposition of LTO over the CNF surface. LTO/CNF film was composed of 50 ± 20 nm diameter LTO polycrystalline mesoporous particles distributed over 300 ± 50 nm diameter CNF nanofibers. As an anode, it delivered a specific capacity of 109 mAh g^−1^ at a rate of 50C.

Li_4_Ti_5_O_12_/rutile TiO_2_ (LTO/RT) heterostructured nanorods with tunable oxide phases in situ fabricated by annealing the electrospun nanofiber precursor delivered a high capacity of 160.3 mAh·g^−1^ at 1C after 200 cycles, 125.5 mAh·g^−1^ at 10C after 500 cycles and superior capacity retention of 90.3% after 1000 cycles at 30C [473].

**Figure 19:**
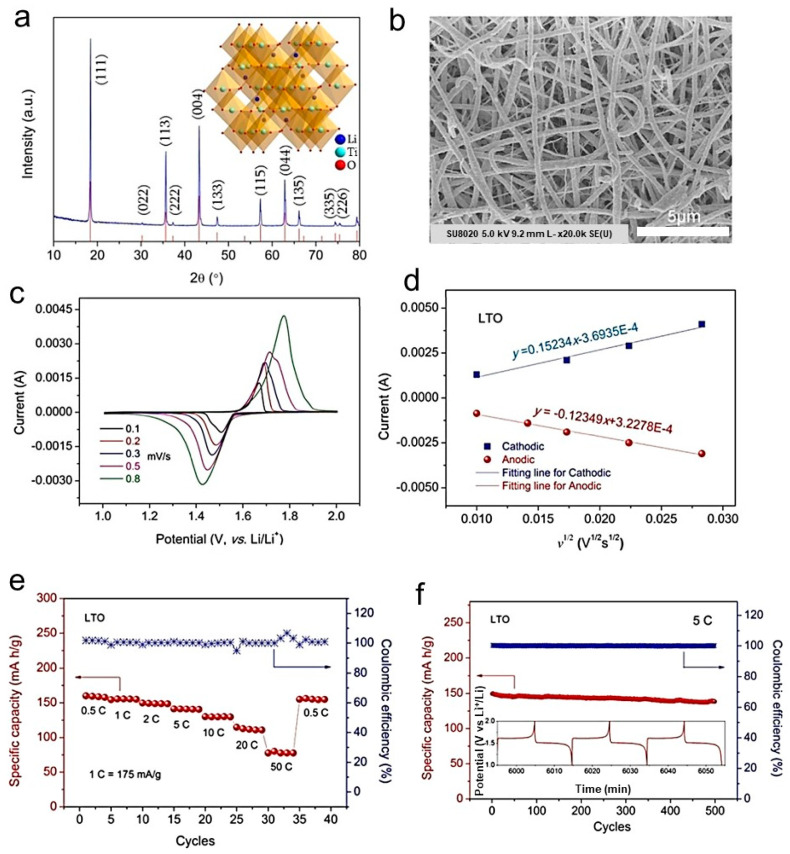
Electrospun LTO nanowires. (**a**) XRD pattern, (**b**) SEM image. (**c**) Cyclic voltammograms at various scanning rates. (**d**) Relationship between peak currents and square root of the scanning rate; Li-ion diffusion coefficient values are 6.33 × 10^−10^ and 4.25 × 10^−10^ cm^2^ s^−1^ for anodic and cathodic reactions, respectively. (**e**) Rate capability. (**f**) Cycling performance of LTO nanowires in Li-ion half cells. Reproduced with permission from [475]. Copyright 2016 Elsevier.

Wang et al. [474] utilized an amphiphilic triblock copolymer (Pluronic F127) as a surfactant owing to the hydrophilic group for the synthesis of electrospun mesoporous LTO/C nanofibers. The initial concentration of F127 in the inorganic precursor solution was lower than its critical micelle concentration (cmc). During electrospinning, the progressive evaporation of ethanol concentrated the surfactant to a concentration that is higher than its cmc. Meanwhile, TIP and LiOAc underwent hydrolysis and condensation, leading to the formation of hydroxyl Li–Ti–O precursors. The self-assembly of hydroxyl Li–Ti–O precursor to form mesopores was driven at a higher concentration of surfactant. For the synthesis of LTO nanowires (500 nm diameter, 10 µm length), Liu et al. [475] used a solution of ethanol (5.0 g), acetylacetone (0.5 g), and acetic acid (1.6 g), where 1.4218 g of tetrabutyl titanate and 0.2298 g of lithium acetate were added. After stirring for 2 h, 0.55 g of PVP (*M*_w_ = 1,300,000) was dissolved into the solution. A transparent yellow precursor was formed after stirring for 24 h at room temperature. The electrospinning process was performed using a 10 mL plastic needle tube tipped with a 24-gauge stainless-steel needle at a rate of 0.8 mL h^−1^. The applied high voltage between the injector nozzle and the collector was 15 kV, and the distance was about 7 cm. The nanowires were annealed in air atmosphere at 700 °C for 6 h with a heating rate of 5 °C min^−1^. Characterizations and electrochemical properties are displayed in Figure 19. In the potential range 1–2 V, the LTO nanowires delivered a specific capacity of 155 and 80 mAh g^−1^ at 1C and 50C rates, respectively. Table 16 lists the details of the electrospinning synthesis and electrochemical performance of LTO nanofibers.

### 2.20. Ion Exchange

Ion exchange is a chemical process in which ions of a certain charge contained in a solution (for example cations) are removed from this solution by adsorption on a solid material (the ion exchanger), to be replaced by an equivalent quantity of other ions of the same charge emitted by the solid. Oppositely charged ions are not affected. An ion exchanger is a salt, an acid, or a base, solid and insoluble in water, but hydrated, that is to say, swollen with water like a sponge. The water content of an apparently dry material can be more than 50% of its total mass and the exchange reactions take place in this water, called swelling or hydration water, inside the exchanger. Ion exchange is another method used to prepare nanoscale LTO particles. This method is feasible for mass production compared with hydro- or solvothermal treatments.

A novel process for the fabrication of LTO was proposed by Lu et al. [121]. LTO was prepared by a two-step ion-exchange technique. They first prepared α-Li_2_TiO_3_ via the hydrothermal treatment of anatase TiO_2_ with a uniform particle size of 4.5 nm in a 2 mol L^−1^ LiOH aqueous solution, which was then reacted with a proper amount of HCl to allow an ion exchange between the α-Li_2_TiO_3_ and the solution. This led to the formation of (Li_0.4_H_0.6_)_2_TiO_3_ nanocrystals. Well-dispersed uniform LTO single crystals (size of ~40 nm) were obtained after the further calcination of the precursor at 400 °C for 2 h. Ma and Cheng [376] used metatitanic acid and lithium hydroxide as raw materials, for the preparation of LTO via one-step hydrothermal ion exchange synthesis method. Yagi et al. [141] prepared Na^+^-intercalated hydrogen titanate nanotubes as LTO precursors from a colloidal solution by dispersing 0.5 g of anatase TiO_2_ powder in 10 mL of a 10 mol L^−1^ NaOH aqueous solution. Li_4_Ti_5_O_12_ with nanotube/nanowire morphology and high surface area was prepared using low-temperature hydrothermal ion exchange processing from hydrogen titanate (H-TiO_2_) precursor [130]. H-TiO_2_ nanotubes were prepared from industrial TiO_2_ powders via a sonochemical-hydrothermal reaction in concentrated 10–15 mol L^−1^ NaOH at 130–170 °C for 24–72 h. Further hydrothermal treatment of the H-TiO_2_ nanotube/nanowires in the aqueous LiOH solution allowed an ion exchange between H-TiO_2_ and the solution to form an LTO precursor. Na^+^ ions in the interlayer of the synthesized H-TiO_2_ were exchanged with Li^+^ ions by a reflux treatment using 100 mL of a 2.5 mol L^−1^ LiOH aqueous solution, in which 1 g of the Na^+^-intercalated H-TiO_2_ powder was dispersed.

LTO nanowires were synthesized via a two-step ionic exchange process from Na_2_Ti_3_O_7_ nanowires obtained by hydrothermal reaction of P-25 TiO_2_ dispersed in 10 mol L^−1^ NaOH solution [132]. Na_2_Ti_3_O_7_ nanowires were subjected to ionic exchange in an HCl aqueous solution to produce H_2_Ti_3_O_7_ nanowires. Next, H_2_Ti_3_O_7_ nanowires were treated in a LiOH aqueous solution via hydrothermal ion exchange at 100 °C for 24 h. Kim et al. [477] elaborated the H_2_Ti_2_O_5_·H_2_O precursor by hydrothermal ion exchange treatment of NaOH on TiO_2_ with HCl solution. LTO nanofibers were subsequently prepared by a second hydrothermal ion exchange process of H_2_Ti_2_O_5_·H_2_O and LiOH·H_2_O and a final calcination at 350–400 °C. Kataoka et al. [478] prepared the lithiated H_2_Ti_12_O_25_ sample by the H^+^/Li^+^ ion exchange synthetic technique in the molten LiNO_3_ at 270 °C using H_2_Ti_12_O_25_ as a starting compound. The obtained sample, H_1.05_Li_0.35_Ti_12_O_25−δ_ having δ = 0.3, exhibits a high discharge capacity of 192 mAh g^−1^ at 1 A g^−1^ current density. The completely Li^+^/H^+^ ion exchanged sample using LiOH or LiI in high-temperature solutions was unsuccessful, resulting in the formation of Li_4_Ti_5_O_12_ and Li_2_TiO_3_ as main products. Nanoparticle-stacked Li_4_Ti_5_O_12_-TiO_2_ nanowire arrays (SLTO) were synthesized via a two-step ion exchange [375]. First, Ti foils were hydrothermally treated in 1 mol L^−1^ NaOH and 3 mol L^−1^ NaCl at 200 °C for 12 h and subsequently immersed in a diluted HCl solution for several hours to thoroughly exchange the Na^+^ with H^+^. Second, the obtained H_2_Ti_2_O_5_·H_2_O nanowire arrays were placed in the solution of 2 mol L^−1^ LiOH·H_2_O at 60 °C for 10 h to thoroughly exchange the H^+^ with Li^+^. Electrochemical performances of nanostructured LTO materials synthesized by using the two-step ion exchange technique are listed in Table 17.

### 2.21. Thin-Film Techniques

Li_4_Ti_5_O_12_ thin films were fabricated using various deposition techniques such as sol–gel coating [257], solid-liquid phase epitaxy [481], radio-frequency (RF) magnetron sputtering method [482,483], direct-current (DC) magnetron sputtering epitaxy [484], and laser-pulse deposition (PLD) technique [13,485,486,487,488,489,490,491]. The sputtering deposition method is the most popular technique to grow metal-oxide films since it allows faster deposition rates. Its main advantage comes from the production of good surface uniformity of as-deposited films. Advantages of the PLD technique include easy control of the film composition by tuning the deposition parameters and a good reproducible stoichiometry of the target material in the films. In the PLD technique, a pulsed laser beam (10 ns duration) is focused by a lens to ablate the Li-rich LTO target. The energy of the beam is in the range of 100–500 mJ per pulse (laser fluence of 10 J cm^−2^) with a laser pulse repetition of 10 Hz. Target and substrates are placed inside the deposition chamber and evacuated to ~1 mPa. To avoid depletion of the deposit at any given spot, the target rotates at 10 rpm. For reactive synthesis, pure oxygen gas is introduced into the chamber with a typical partial pressure (pO_2_) in the range of 0.1–10 Pa to obtain single-phased and stoichiometric LTO films. The typical deposition rate is in the range of 2–5 Å s^−1^. Epitaxial thin-films fabricated by PLD could provide an ideal surface with a roughness of less than 1 nm. However, a target ceramic containing excess Li species is required to fabricate stoichiometric Li_4_Ti_5_O_12_ thin films [492].

Rho et al. [256,257,493,494] fabricated LTO films using a sol–gel coating with Ti and Li isopropoxide added by propanol (*i*-C_3_H_7_OH), acetic acid (CH_3_COOH)and poly(vinylpyrrolidone) (PVP, 55,000 m.w.) as an additive for sol, which is very effective for preparing crack-free thin films. The molar composition of the starting solution was 5:4:100:100:5. The sols were coated on the Au substrate or the quartz glass substrate with a spin coater at 3000 rpm. The prepared sol films were converted to gel films in the course of the spin-coating process. Then, the gel films were heated at 800 °C for 1 h. LTO thin film 0.4 µm thick showed a high electrochemical response until 50 mV s^−1^. Mosa et al. [174] reported on the sol–gel process and coating preparation of spinel Li_4_Ti_5_O_12_ thin films by dipping using lithium ethoxide in situ that reacts with titanium alkoxide as the starting reagents to produce precursor solution without precipitation. The alkoxide solution method shows promising potential for control over particle size, high purity, good chemical homogeneity, low thermal treatment temperatures and times, and micro-structural uniformity. In situ production of a lithium alkoxide and its reaction with a titanium alkoxide reduces the thermal treatment to obtain a pure phase at only 700 °C and 15 min. Wang et al. [482] reported the fabrication and characterization of LTO films grown by RF magnetron sputtering onto Au(100 nm)/Ti(10 nm)/SiO_2_/Si substrate maintained at various temperatures in the range of 500–700 °C. The spinel phase of LTO appears at deposition temperatures above 500 °C. The films were deposited at the pressure of 30 mTorr with the mixed Ar/O_2_ (3:2) gas, and the power density was estimated to be ~4 W cm^−2^. Wude et al. [495] reported LTO thin films (250 nm thick) deposited by dc-ion beam sputtering at different O_2_ partial pressures and different substrate temperatures. Thin films prepared at a substrate temperature of 600 °C and an oxygen partial pressure of 3 × 10^−4^ mbar show the most characteristic LTO features. Kumatani et al. [477] fabricated Li_4_Ti_5_O_12_ (111) epitaxial thin films on α-Al_2_O_3_(0001) substrates by RF magnetron sputtering. Thin films of amorphous Li_4_Ti_5_O_12_ were deposited at room temperature, and then the films were annealed at high temperatures for the formation of epitaxial thin films. The RF power applied to the target was maintained at 100 W, and the films were deposited in a mixed Ar/O_2_ atmosphere at different oxygen partial pressures (*p*O_2_), controlled by the flow ratio of Ar to O_2_. The total pressure was kept at 1.0 Pa, and thin film thicknesses were approximately 100 nm (deposition rate was approximately 1.5 nm/min). After the deposition, the films were annealed at different temperatures (800–1100 °C) in a vacuum (~1.0 × 10^−3^ Pa).

**Figure 20:**
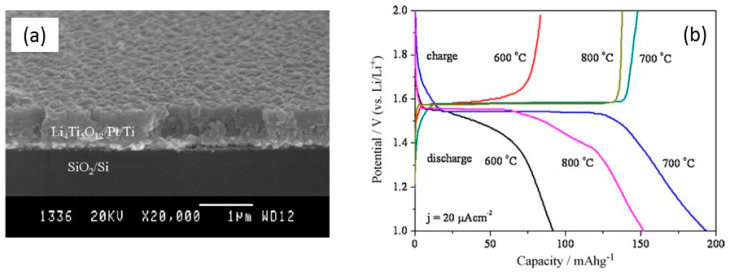
(**a**) SEM cross-section image of LTO film (410 nm thick) heat treated at 800 °C. (**b**) Charge-discharge profiles recorded at 20 µA cm^−2^ (i.e., ~1.15C) current density in the voltage range 1–2 V vs. Li^+^/Li of PLD films heated at various temperatures. Reproduced from [485] with permission. Copyright 2009 Elsevier.

The first PLD growth of LTO thin films deposited onto Pt/Ti/SiO_2_/Si substrates using a KrF excimer laser beam (248 nm, 250 mJ) was reported by Deng et al. [485]. Films annealed at 800 °C (410 nm thick) exhibited a cubic structure with a lattice constant 8.375 Å larger than that of the LTO crystal (8.359 Å). The SEM cross-section image (Figure 20a) revealed the porous morphology induced by the high-temperature treatment. The discharge-specific capacity was the largest for films annealed at 700 °C due to the optimized adhesion strength between film and substrate (Figure 20b). The anode films discharged at a current density of 10 µA cm^−2^ (0.58C rate) showed excellent cycleability; the discharge capacity remained 149 mAh g^−1^ after 50 cycles. Yu et al. [488] reported the growth of LTO films (545 nm thick) deposited at 600 °C for 90 min on conducting fluorine-doped tin oxide (FTO) by PLD using a KrF excimer laser at an energy density of 5 J cm^−2^ with a repetition rate of 9 Hz. Using high-purity oxygen kept at 30 P, the films are grown with a grain size of ~250 nm and roughness of 50 nm in the perpendicular direction toward the thin film.

Hirayama et al. [496,497] fabricated epitaxial Li_4_Ti_5_O_12_ thin-films deposited on SrTiO_3_ single-crystal substrates with (111), (110), and (100) lattice plane orientations using a PLD apparatus equipped with a KrF excimer laser with a wavelength of 248 nm under O_2_ atmosphere. LTO films have the same orientation as the SrTiO_3_ substrates: Li_4_Ti_5_O_12_ (111) on SrTiO_3_ (111), Li_4_Ti_5_O_12_ (110) on SrTiO_3_ (110), and Li_4_Ti_5_O_12_ (100) on SrTiO_3_ (100). These epitaxial films contained island structures, and the morphologies of the (111), (110), and (100) films exhibit angular, needle-like, and circular shapes, respectively. Impurity-free epitaxial LTO thin-film was obtained from a Li-rich target Li_5.2_Ti_5_O_12_ when deposited at *T*_s_ = 700 °C. The electrochemical features of LTO film anodes (20 nm thick) exhibited discharge capacities of ~200 and ~250 mAh g^−1^ for (100)- and (111)-orientation, respectively. Epitaxial LTO(110) films were also deposited on Nb:SrTiO_3_(110) substrate. These films (~28 nm thick) tested by cyclic voltammetry at a scan rate of 1 mV s^−1^ exhibited redox peaks at 1.53 and 1.60 V corresponding to the insertion and extraction of Li^+^ ions. As-deposited films at a substrate temperature of 700 °C in 6.6 Pa oxygen partial pressure exhibited high initial capacity (~200 mAh g^−1^) but poor stability [497]. Kumatani et al. [486] investigated the PLD growth process of epitaxial LTO films deposited on the MgAl_2_O_4_ (111) substrate. With *T*_s_ = 800 °C and *P*_O2_ = 1 × 10^−3^ Torr LTO films had excellent crystallinity and low resistivity of 3.3 × 10^−4^ Ω cm. at 25 °C. At lower *P*_O2_, the PLD LiTi_2_O_4_ film was formed, while at higher *P*_O2_ Ti was segregated as TiO_2_ rutile and Li_0.74_Ti_3_O_6_.

Studies of the electrochemical performance and kinetic behavior of PLD LTO films deposited on Pt/Ti/SiO_2_/Si substrates were reported by Deng et al. [489]. Using a Li-rich target (i.e., an excess of 5 wt.% Li_2_O), the films annealed at 700 °C for 2 h in air were well-crystallized items with densely packed grains. The galvanic charge-discharge plateau was observed around 1.56 V and an initial specific capacity of 159 mAh g^−1^ was delivered with a retention of 93.7% after 20 cycles. The diffusion coefficient of Li^+^ ions in such an LTO framework was in the range of 10^−15^–10^−12^ cm^2^ s^−1^. The energy barrier of the diffusion of lithium ions was estimated to be *E*_a_ = 0.11 eV in LTO (111)-oriented PLD thin films (190 nm thick) grown on spinel MgAl_2_O_4_ (111) substrate [13]. Zhao et al. reported the optical properties of epitaxially grown LTO films on (001)-oriented MgAl_2_O_4_ substrate. The optical bandgap of 3.14 eV was measured for 86 nm thick films (surface roughness of 4.61 nm) [490]. Schichtel et al. fabricated an all-solid-state microbattery with LTO as a positive electrode. PLD films were obtained on various substrates at *T*_s_ = 650 °C under a 0.3 Pa pure oxygen atmosphere using a commercially available LTO powder. As-prepared films (650 nm thick) revealed columnar growth that allowed a Coulombic efficiency >97% after the second cycle and a discharge capacity of 33 µAh cm^−2^ at 3.5 µA cm^−2^ current density [491]. Pfenninger et al. demonstrated that LTO thin films deposited by PLD on MgO substrate kept at 500 °C using dense Li_7.1_Ti_5_O_12_ target sintered at 1000 °C for 12 h are compatible with Li_6.25_Al_0.25_La_3_Zr_2_O_12_ electrolyte pellet. Such films display stable structure and cycleability almost close to 175 mAh g^−1^. The typical voltage plateau at 1.57 V (oxidation) and 1.53 V (reduction) was observed at the current density of 2.5 mA g^−1^ [498]. Among the Li_1+x_Ti_1−x_O_4_ ternary system, LiTi_2_O_4_ thin films were grown by the PLD route in the temperature range of 400–800 °C using a target with a higher Li/Ti ratio of 0.8 [499]. Chopdekar et al. grew epitaxial PLD LiTi_2_O_4_ thin films on various crystalline-oriented substrates such as single crystalline substrates of MgAl_2_O_4_, MgO, and SrTiO_3_ [500]. The authors stated the PLD conditions with *T*_s_ held at 450–600 °C in a vacuum better than 5 × 10^−6^ Torr without mention of oxygen partial pressure, while Kumatani determined that stoichiometric LiTi_2_O_4_ thin films were obtained at a *P*_O2_ of 5 × 10^−6^ Torr with *T*_s_ = 800 °C [486]. Pagani et al. [501] reported the growth of epitaxial, single-crystal, strain-free LTO thin films (150 nm thick) deposited by PLD on MgO (111) single-crystal substrate at 500 °C in an oxygen atmosphere at a pressure of 1.7 Pa. A KrF excimer laser (λ = 248 nm) was employed at a fluence of 2.6 J cm^−2^ and a frequency set at 10 Hz. For comparison, polycrystalline LTO film, representing a nonideal system including grain boundaries, was deposited on a sputtered polycrystalline MgO film on Si(100). In a second publication, the target for PLD was prepared by mixing and grinding Li_2_CO_3_ and TiO_2_ rutile to obtain an overlithiated target with a composition of Li_5.35_Ti_5_O_12_. Recently, the same group [484] used Li_4_Ti_5_O_12_ and Li_2_O targets simultaneously in confocal configuration to sputter LTO epitaxial films at a power density of 4.93 and 1.48 W cm^−2^, respectively, and at a substrate temperature of 700 °C in an argon atmosphere at a pressure of 1.5 mTorr and an argon flow of 30 sccm resulting in a deposition rate of 0.14 Å s^−1^. Lithium losses were mitigated by Li_2_O co-sputtering at a rate of 0.02 Å s^−1^. Yu et al. [487] prepared textured Li_4_Ti_5_O_12_ thin films (thickness from 35 to 210 nm) via PLD. Films were deposited on various substrates maintained at 450 °C under a flowing oxygen atmosphere at 5 Pa. An XeCl excimer laser with a wavelength of 308 nm and laser pulse frequency of 8 Hz was used for thin-film growth. Both the target and substrate holder were rotated at a constant speed to ensure uniform film thickness in each sample. The distance between the target and the substrate was kept at 4.7 cm. Prior to film deposition, the vacuum chamber was evacuated to a background pressure of 1 × 10^−4^ Pa. The thin films were further annealed at 600 °C in an atmospheric environment for 30 min to increase their crystallinity. The growth rate is estimated to be 2.33 nm min^−1^. It is shown that the pseudocapacitive charging is remarkably activated by the nanocrystalline microstructure full of defect-rich surface, which can simultaneously promote Na-ion and electron accessibility to the surface/subsurface. A highly reversible charge capacity of 225 mAh g^−1^ at 1C is achievable for the Li//LTO supercapacitor with 1 mol L^−1^ LiClO_4_ in a nonaqueous solution of ethylene carbonate and polycarbonate (50:50, wt.%). Cunha et al. [502] fabricated PLD LTO films deposited on Nd-doped single crystalline SrTiO_3_ (100), (110) and (111) substrates at *T*_s_ = 700 °C under *P*_O2_ = 20 Pa from a sintered Li_4.8_Ti_5_O_12_ (20 wt.% excess Li_2_O) target. It was shown that increasing the relative amount of ⟨111⟩ facets would significantly increase the storage capacity, although the reversibility of this capacity could be limited by irreversible surface reactions at these high compositions.

### 2.22. 3D Ink Printing

The fabrication of wearable batteries using 3D printing approaches is highly desired because of their capability of printing arbitrary shapes and sizes and configuring multiple materials at different positions as needed. These techniques include lithography-based 3D printing, inkjet printing, direct ink writing, and fused deposition modeling. The composition and rheology of each ink must be optimized to ensure reliable high aspect ratio electrode architectures [503]. Li_4_Ti_5_O_12_ is the most commonly used anode material in 3D-printed batteries [504,505,506,507,508,509,510,511,512,513,514,515]. The first 3D-interdigitated microbattery architectures (3D-IMA) with LTO/LFP materials were developed in 2013 by the Lewis group [504]. LTO ink was well designed by adding deionized water, 20–30 wt.% ethylene glycol, 27 wt.% glycerol, and 9 wt.% cellulose-based viscosifiers. Prior to printing, interdigitated Au current collector patterns were prepared by a combination of lithographic patterning and e-beam deposition. The LTO ink was then deposited onto a pattern to form multilayer electrodes. After the printing was finished and the electrodes were dried, the LTO interdigital structure was heated to 600 °C in an inert gas to remove the organic additives to advance the nanoparticle sintering. The packaged micro-battery showed a capacity of 1.2 mAh cm^−2^ at a rate of 0.5C and exhibited a high areal energy density of 9.7 J cm^−2^ at a power density of 2.7 mW cm^−2^.

Zhao et al. fabricated LTO thin films by using the ink-jet printing technique [505]. The average thickness of 10-layer LTO film was about 1.7~1.8 μm and the active material in the thin film was nano-sized about 50–300 nm. It was also found that the as-prepared thin film exhibited a high discharge capacity of about 174 mAh g^−1^ and the discharge capacity in the 300th cycle retained 88% of the largest discharge capacity at a current density of 10.4 μA cm^−2^ in the potential range of 1.0–2.0 V [505]. Chen et al. [506] fabricated a 3D microbattery using an ultraviolet-curable poly(ethylene glycol) (PEG)-based gel polymer as a resin for micro-stereolithography. Active materials, LiFePO_4_ (cathode) and Li_4_Ti_5_O_12_ (anode) were mixed with carbon black and the GPE resin, which was then flown into the 3D structure. This LFP/PEG-gel polymer/LTO microbattery delivered a specific capacity of 1.4 µAh cm^−2^ at a 2C rate. Inkjet printing of ionogel was elaborated to construct a lithium microbattery delivering 300 µAh cm^−2^ over 100 cycles (i.e., 60 mAh g^−1^ at 0.1C rate) [507]. Homogenous porous composite electrodes, obtained by tape casting an aqueous slurry, contained an active material powder (LTO and LFP), a polymeric binder (carboxyl methyl cellulose (CMC), M_w_ = 250,000 g mol^−1^), and an electronic conductor powder (carbon Super P, SSA = 60 m^2^ g^−1^). Zhou et al. [508] used the 3D printing technology to optimize the electrode geometry for the sake of enhanced electrochemical performance. 3D direct writing ink composed of LTO, carboxymethylcelluclose sodium (CMC), carbon nanotube, and water achieved LTO electrical conductivity of 2.08 S cm^−1^ and an ultrahigh areal capacity of 5.05 mAh cm^−2^. The full cells assembled with the 3D-printed LFP and LTO microlattices showed a stable capacity and still delivered a capacity of 102 mAh g^−1^ after 35 cycles. In a recent work, Wei et al. prepared LTO functional ink using 30 vol.% LTO with 1.35 vol.% Ketjenblack in 1 mol L^−1^ LiTFSI/anhydrous propylene carbonate with 1 wt.% PVP (*M*_w_ = 40,000 g mol^−1^). The packaged LIBs composed of thick LFP/LTO electrodes, customized separator, and glassy carbon current collectors delivered an areal capacity of 4.45 mAh cm^−2^ (second cycle) at a current density of 0.14 mA cm^−2^ [509]. Wang et al. [510] incorporated LTO nanofibers into a PVDF (*M*_w_ = 600,000 g mol^−1^) dissolved in n-methyl-2-pyrrolidone (NMP) solution with CNT conductive additive to make the anode ink. The all-fiber quasi-solid-state LIB device assembled by twisting LFP and LTO printed electrodes together with gel polymer electrolyte, exhibited a high discharge specific capacity of ≈110 mAh g^−1^ at 50 mA g^−1^. To achieve a balance between the electrochemical, mechanical, and rheological properties of ink-printed LTO anode, Kohlmeyer et al. [511] developed a system in which the ink components (i.e., active material, carbon nanofibers (CNFs), and polymer) could be independently varied to tune the ink rheology and the final electrode properties. Each batch consisted of 100 mg of active material and 5 mL solvent (i.e., 1-methyl-2-pyrrolidone (NMP), diethyl carbonate, and isopropanol). Direct ink write printing was performed by extrusion at a pressure ranging from 0.5 to 5 psi. The Li_4_Ti_5_O_12_/CNF/PVDF electrode with composition 40/40/20 displayed a conductivity of ~10 S cm^−1^ and delivered a specific capacity of 89 mAh g^−1^ at 5C rate. Ragones et al. [512] utilized a fused-filament fabrication (FFF) method for 3D printable microbatteries (3DPMs). The fabrication of electrochemically active LTO was realized using LTO/carbon/polyester polylactic acid mixture. The reversible capacity of the Li/0.3 mol L^−1^ LiTFSI–PYR14TFSI/LTO half-cells was 80 mAh g^−1^ at 30 µA cm^−2^ after 30 cycles. Recently, Viviani et al. [515] proposed a simple ink formulation that is aqueous-based, non-toxic, and safe to handle. In a typical procedure, 1.5 g of LTO powders, 0.21 g of polyvynilpirrolidone (PVP), and 0.187 g of a carbon-based conductive agent, either carbon black or MWCNTs, were ball milled at 420 rpm for 8 h with 2.5 mL of ethylene glycol and 2.5 mL 2-propanol (IPA). A second ball-milling step was carried out for 1 h at 150 rpm after adding 40 mL aqueous solution of 1 mmol L^−1^ lithium dodecyl sulfate (LDS) and 0.4 g of lithium polyacrylate (Li-PAA). Inkjet-printed LTO thin-film electrodes manufactured with carbon black and MWCNTs deliver a specific capacity of 150 mAh g^−1^ at 0.2 C, showing negligible capacity loss for over 100 cycles.

### 2.23. Miscellaneous Treatments

**Figure 21:**
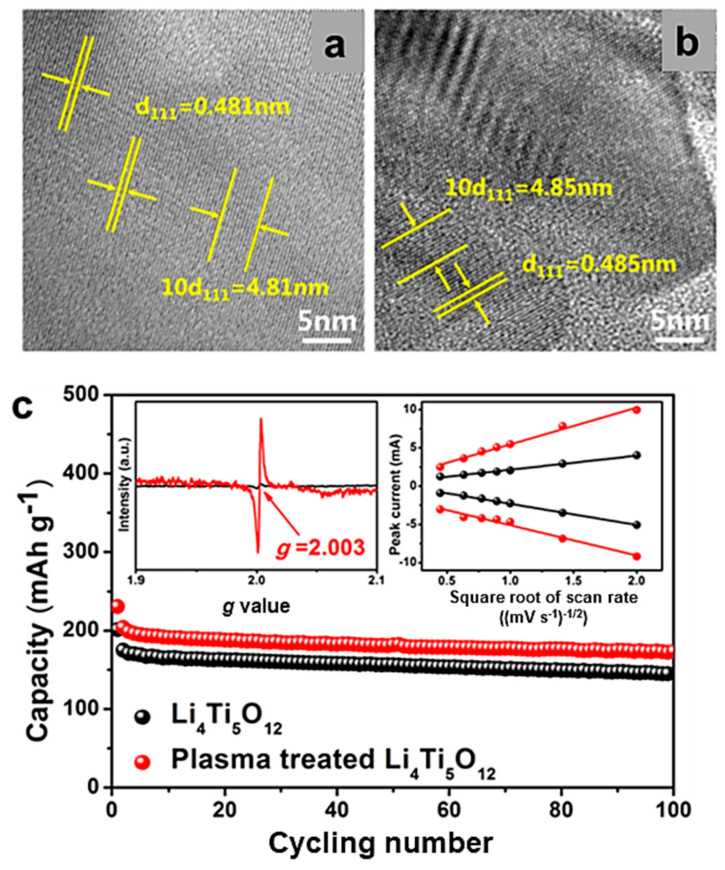
Characterization of pristine LTO and oxygen defective LTO. HRTEM images of (**a**) as-prepared LTO and (**b**) plasma-treated LTO. (**c**) Cycling performance at 1C rate of pristine LTO and plasma-treated LTO. Insets show the electron paramagnetic resonance (EPR) spectra and the linear relationship between the peak current of cyclic voltammograms and the square root of scan rate. The high EPR signal at *g* = 2.003 in plasma-treated LTO originates from the unpaired electrons trapped in O vacancies. Reproduced with permission from [517]. Copyright 2019 The American Chemical Society.

LTO confined in activated carbon nanopores (LTO/AC) prepared by a vacuum impregnation technique yielded nanocomposite electrodes for asymmetric supercapacitors [516]. Typically, 340 mg of titanium (IV) butoxide and 57 mg of lithium acetate were dissolved in 2 mL of methanol by ultrasonication for 5 min to obtain a transparent solution with a bright yellow color as the precursor. After adding the solution dropwise to 200 mg spherical nanoporous activated carbon, a vacuum was applied during the interval of every second addition. The precursor AC composites were then preheated in air at 300 °C for 1 h, followed by calcination at 800 °C in an argon atmosphere for 10 h. LTO-AC with a large content of 30−50 nm mesopores retains up to 50% of its capacity at the 200C rate. For boosting the Li-ion transport in the spinel framework of LTO, Zhu et al. incorporated oxygen defects by eco-friendly and cost-effective plasma treatment in an H_2_/N_2_ atmosphere at a pressure of 5 Pa at 150 °C for 2 h [517]. Oxygen vacancies were evidenced by XRD, XPS, UV-Vis, and ESR measurements (Figure 21). A clear shift of the (111) and (400) X-ray reflections is observed towards lower 2θ angles due to the lattice expansion of ~1% due to the partial reduction of Ti^4+^ to larger Ti^3+^ ions to maintain charge neutrality. The oxygen-deficient LTO delivered a capacity of 133 mAh g^−1^ at a 20C rate after 500 cycles with a Coulombic efficiency of 100% (Figure 21).

Sodium titanium oxide with a spinel-type structure (Na_3_LiTi_5_O_12_, NTO) phase was demonstrated to be a stable structure as an anode material for sodium-ion batteries (SIBs) [518]. NTO was fabricated by the reaction of commercially available LTO powder with a sodium–organic reduction reagent (SOR). SOR was prepared using an excess of sodium-metal flakes dissolved into the biphenyl 1,2-dimethoxyethane solution. The color of the LTO powder soaked into SOR solution for 1 day in Ar atmosphere turned to black from white, confirming that enough sodium insertion reaction has occurred.

To overcome the drawbacks of pure LTO materials, i.e., low electronic conductivity at room temperature and moderate Li^+^ diffusion coefficient, surface modification to change the oxygen stoichiometry has been utilized. Thermal nitridation of metal oxides has been known to change the oxygen stoichiometry, to reduce the metal oxidation state, and to introduce nitride thin films [519,520,521]. In 1994, Richard et al. demonstrated the modification of the surface structure of spinel oxides, LiMn_2_O_4_ and Li_4_Ti_5_O_12_ via annealing in ammonia [520]. Nitridation should change Li insertion/extraction behaviors because Li_4_Ti_5_O_12−δ_ has an average Mn oxidation state of less than 4 (i.e., formation of a mixed-valent intermediate phase) and substantial Li can be extracted from it (i.e., resulting in an increase discharge capacity). From Rietveld refinements, it was found that LTO treated at 150 and 200 °C under a flow of anhydrous NH_3_ for 1.5 h exhibits oxygen occupation in 32*e* Wyckoff sites of 3.958 and 3.873, respectively. In addition, NH_3_ treatment should also make bonding between surface Ti and N simultaneously. As a functional material easily prepared, TiN has high thermal stability, high chemical stability, and, also, high electronic conductivity, and it introduces a significant enhancement in the battery performance. For example, a simple synthesis of nitrogen- and TiN-modified LTO by solid-state reaction of Li_2_CO_3_ and TiO_2_ anatase in an ammonia-containing atmosphere was reported [522]. A uniformly coated conducting TiN/TiO_x_N_y_ layer was synthesized using electrospinning and subsequent nitridation process on the surface of LTO nanofiber [468]. Wang et al. [193] used a different procedure for the LTO nitridation by heating the powders from room temperature to 700 °C at the rate of 5 C min^−1^ under Ar atmosphere; then, the gas was changed into NH_3_ and the temperature was kept at 700 °C for ~2 h, followed by natural cooling to room temperature under flowing Ar gas preventing oxidation. The TiN layer formed on the surface of Li_4_Ti_5_O_11.7_Br_0.3_ powder after heating in NH_3_ provided excellent electrochemical performance, i.e., a reversible capacity of 138 and 104 mAh g^−1^ after 100 cycles at 10C and 20C, respectively. Li et al. have successfully prepared TiN-coated LTO submicrospheres by solvothermal method and subsequent nitridation process in the presence of ammonia [186]. With a TiN coating layer 5 nm thick, the anode material delivered a discharge capacity of 101 mAh g^−1^ at a 20C rate.

Doping is one of the best and most cost-effective ways to improve the performance of LTO as an anode material. An extensive review of the doping strategy of LTO has been published by Ezhyeh et al., concluding that among the metal dopants Al, Ca, Co, Fe, Nb, Ru, V, W, and Zn present the highest discharge capacity in the range of 190–340 mAh g^−1^, while in non-metal candidates N with discharge capacity of 226 mAh g^−1^ shows the best result and finally Al/Mn as a good performance of co-doping displays discharge capacity about 230 mAh g^−1^ [37]. We can also mention Cu^2+^ and F^−^ co-doping. The best results were obtained with Li_4_Ti_4.75_Cu_0.25_O_11.7_F_0.3_, which delivered a capacity of 134.7 mAh g^−1^ at 1C with a capacity retention of 97.7 % after 100 cycles [523]. Nevertheless, even better results were obtained recently by Ali et al. who succeeded in doping LTO with Cd^2+^ at the Li(8*a*) site to obtain Li_4–*x*_Cd*_x_*Ti_5_O_12_ prepared by a solid-state route up to the limit of x = 0.20. The Cd dopant is at the Li(8*a*) site in the LTO lattice. At rate 2C, the half-cell obtained with x = 0.20 delivered an initial capacity of 106.8 mAh g^−1^, with 98.62% retention after 300 cycles [524]. Noreochim et al. also used the solid-state route to prepare lithium iodide-doped LTO by adding 0.2 mol of LiI to the precursors of LTO based on 1 mol LTO. This I-doped LTO delivered a capacity of 123 mAh g^−1^ at 15C. At 1C, the cell delivered a discharge capacity of 171 mAh g^−1^ with a capacity retention of 99.15% after 100 cycles [525]. Synergetic effects can be obtained by the synthesis of composites made of doped LTO and a conductive material.

Qasim and Mousa used a solid-state method to produce pure LTO and Mg, Mn, and V-doped LTO nanoparticles, as well as a chemical polymerization method to synthesize polyaniline (PANI) and composites of pure and doped LTO@PANI nanoparticles. The V-LTO@PANI demonstrated the highest performance with pseudo-capacitive behavior. It demonstrated a specific capacitance of 202 F g^−1^, an energy density of 72.8 Wh kg^−1^, a power density of 2430 W kg^−1^, and 82.6% capacitance retained over 3000 cycles at 1 A g^−1^ [526]. Self-doping is also a promising strategy to improve the LTO properties. In. particular, Yeo et al. synthesized porous zeolitic LTO (Z-LTO) microspheres using TiO_2_ and LiOH∙H_2_O via a hydrothermal process combined with Ar/H_2_ thermal treatment. The increased concentration of Ti^3+^ self-doping-derived oxygen vacancies improved significantly the electronic conductivity and structural stability, as the anode delivered a capacity of 181 mAh *g*^−1^ at a 5C rate after 2000 cycles, corresponding to 90% capacity retention [527].

Tian et al. prepared in-situ growing Ti_3_C_2_T_x_ MXene/LTO (M-LTO) nanocomposite via controllable natural oxidation and hydrothermal lithiation method. The flake-like Ti_3_C_2_T_x_ MXene was used as both the titanium source and conductive skeletons to support LTO. In the process, the monodispersed LTO nanoparticles grew evenly on the surface of Ti_3_C_2_T_x_ MXene. The in situ growing M-LTO composites resulted in an oriented growth between LTO and Ti_3_C_2_T_x_ MXene, i.e., LTO [110]//Ti_3_C_2_T_x_ [001], which improved the electrical conductivity. As a result, the MXene/M-LTO) exhibited a much superior rate performance than the mixture of Ti_3_C_2_T_x_ MXene and LTO or pristine LTO. At 10C, a reversible capacity of 137 mAh g^−1^ was obtained, with a capacity retention of 87.5% after 1000 cycles [528]. Li et al. reported a different approach to utilizing Ti_3_C_2_ in batteries, in which Ti_3_C_2_ only serves as an intermediate for the synthesis of LTO. The Ti_3_C_2_ with extracted Ti-ion served as the raw material for a one-step hydrothermal process. The resultant LTO product (M-LTO) inherited the original morphology of Ti_3_C_2_ and contained the in-situ formed carbon quantum dots increasing the conductivity. At 5C, this anode delivered an initial capacity of 175.4 mAh, maintained at 160.7 mAh g^−1^ after 5000 cycles, corresponding to a capacity retention of 91.3%. It still delivered capacities of 168.8 and 168.4 mAh g^−1^ at 30 and 40C, respectively. At the low rate of 0.5C, the capacity is barely affected when the temperature is lowered down to −30 °C [529].

Wang et al. employed the industrial metatitanic acid (H_2_TiO_3_, HTO) as the Ti source to be wrapped with the carbon source first, and then mixed with the lithium source, followed by only one sintering process to prepare LTO. The low-cost glucose was introduced to pre-coat HTO, which could not only avoid the generation of Li_2_SO_4_ impurity but also suppress the particle growth of LTO. The amorphous crystal structure of HTO is more conducive for the Li^+^ insertion, making it possible to decrease the calcination temperature to 700 °C, which is hard to achieve for TiO_2_ as the Ti source in the traditional route. As a result, the LTO particles were uniformly coated with carbon. As an anode, the composite delivered the specific capacity of 132.9 mAh g^−1^ is retained even under the current rate of 20C. At 2C, the capacity was 157 mAh g^−1^ with a capacity retention of 84.8% after 1000 cycles [184]. This synthesis process reduces importantly the fabrication cost of the LTO anode.

Meng et al. used the flame melting method to prepare oxygen-vacancy-rich LTO hollow macrospheres of 40–80 μm in diameter, providing a tap density of 1.26 g cm^−3^. The synthesis proceeded in three steps: i) Li_2_Ti_3_O_7_: Li_4_Ti_5_O_12_ and Li_2_Ti_3_O_7_ were prepared by a conventional solid-state method. ii) Li_2_Ti_3_O_7_ hollow microspheres were prepared by the flame melting method. iii) The Li_2_Ti_3_O_7_ hollow macrospheres were heated under an Ar atmosphere at 600–900 °C for 5 h. This material delivered excellent low-temperature performance even at a high mass loading (5 g cm^−2^). At −20 °C, the specific capacities achieved at 1C and 5C are 136.5 and 107.6 mAh g^−1^, respectively, stable over the 500 cycles that have been tested [530]. This synthesis provides insight into the design of an LTO anode for high mass loading at low temperatures, which outperforms the conventional LTO prepared by solid-state reaction.

## 3. Concluding Remarks

In this article, we have presented a comprehensive overview of the procedures and different strategies used for the synthesis of Li_4_Ti_5_O_12_ anode materials. Spinel Li_4_Ti_5_O_12_ can be synthesized by many different techniques: solid-state reaction, sol–gel methods, microwave-assisted synthesis, spray pyrolysis, and in hydrothermal batch reactors. The sol–gel method can be employed to prepare particles with uniform composition distributions at low temperatures. Spray pyrolysis enables the direct synthesis of powder from precursor solutions, using low processing temperatures and resulting in high purity and homogeneity of the as-synthesized material. However, it is difficult to control the morphology and chemical composition of the products. Hydrothermal synthesis is known for allowing the preparation of nanoparticles at low temperatures, but the products usually have low crystallinity, which might cause a fast decrease in the capacity of nanoparticulate LTO. In addition, organic residues that appear when using organic solvents result in low Coulombic efficiency. For practical applications, the solid-state method is the most widely used method since it is easy to scale up, and the precursors are cheap and abundant.

We show that optimized LTO is a very good electrode material exhibiting excellent reversible lithium-ion intercalation and de-intercalation processes during charge–discharge cycling without structural change (zero-strain insertion material). Additionally, unlike graphite, LTO exhibits an attractive property for the LIB anode since it operates within the stable electrolyte voltage window with reduced solid electrolyte interphase formation. LTO anode has favorable characteristics for automotive applications being explored intensively. Moreover, recent prospects have shown that LTO is a promising candidate as anode material for SIBs [531]. At present, the strategies to improve the sodium storage performance of LTO include: (i) surface coating and ion doping to increase the ion diffusion rate and electron conductivity and to alleviate the lattice distortion during sodium encapsulation; (ii) designing nano-sized LTO materials to improve the properties of materials by shortening the ion diffusion distance and increasing the contact area with electrolyte.

It has been long recognized that the complete electrode microstructure is decisive for the charge transport and for this reason, research effort is aimed at controlling the particle morphology through the choice of an adequate strategy for the material synthesis. Additionally, the best technique must be relatively cost-effective for commercial applications. There is a general trend showing that nanosized LTO has a higher capacity and better rate performance; however, its production is expensive. Therefore, a trade-off must be found between the performance and cost of the final product. Various computer models have been proven to be invaluable in studying the effects of particle size on the electrochemical performance of LTO anode materials and have been used to define appropriate design parameters needed for high-power applications [532]. Newman and co-workers utilized a pseudo-2D (P2D) model to characterize the performance of the LTO electrode [533]. A full-cell mathematical model is used to compare the performance of graphite (Li_x_C_6_) and LTO, building Ragone plots as a function of the electrode porosity. Kashkooli et al. [534] showed that it was possible to optimize a particle size according to the desired operating rate. A model design with adjustable parameters based on monodispersed active electrode particles reflects real battery performance and provides the optimum nano-particle size, which eliminates the performance loss due to the limited mass transportation. Table 18 lists the advantages and drawbacks of LTO synthesis methods.

For industrial production, the solid-state method is the most widely used technique since it is easy to scale up and the precursors are cheap and abundant. As mentioned by several workers, this method often leads to particle agglomeration or sintering [220,230,535]. For most titanium salts, the starting precursors are easily hydrolyzed to form TiO_2_, which is necessary to heat with lithium salts at high temperatures for extended periods of time to obtain well-crystallized LTO powders. This process is energy-consuming. Furthermore, at high temperatures, the LTO particle sizes grow to several hundred nanometers or even several microns resulting in an increase of the lithium diffusion path within isolated particles and thereby decreases electrode performance.

Solvo/hydrothermal methods using cost-effective precursors are known for allowing the preparation of nanoparticles at low temperatures, but the products usually have low crystallinity. However, they provide nanosized LTO particles exhibiting highly improved rate performances at C-rates as high as 30C and 60C, when compared to LTO prepared by the solid-state reaction method. The sol–gel synthesis or Pechini process is currently employed to synthesize particles with uniform composition distributions at low temperatures. In such processes, Li and Ti cations are normally trapped homogeneously on the atomic scale throughout the polymer matrix and thus the reaction temperature and time are reduced. Using low processing temperatures, spray pyrolysis has the advantage of the direct synthesis of powder from precursor solutions, resulting in high purity and homogeneity of the as-prepared LTO material. However, controlling the morphology and chemical composition of the powder is difficult. The best electrochemical performances are influenced by the small particle size, good particle dispersion, and high specific surface area of the LTO anode.

Note that in this review, we did not discuss the thermodynamic stability region issues because, even though the solid-state and sol–gel reaction processes are suitable for large-scale fabrication, they cannot be used to synthesize LTO with particle sizes <300 nm. Smaller particles are needed to obtain good electrochemical properties. That is why modern high-energy mechanical milling is employed to facilitate pulverization to obtain an LTO powder with nanoscale particle size by mechanical force that produces materials with metastable and nano-crystalline phases. In such a case, the effort paid to nucleation and growth direction related to surface chemistry before ball milling has little effect after the milling. The referee mentions the phase stability of the precursors, but this has been analyzed for instance in Ref. [76], as reported in the paper, with the stabilization of water-sensitive metal alkoxides precursor, and the importance of the presence of carboxylic acids.

In the spray pyrolysis process, precursor salts are dissolved in a solution that allows uniform mixing of each component at the molecular level, leading to high chemical purity in the products. This technique is cost-effective and industrially scalable, providing nanostructured ceramics and composites with high electrochemical performances. Using appropriately chosen precursor salts, spray pyrolysis is expected to decrease heat treatment times, and thus save energy and cost in large-scale production.

Substantial efforts have been devoted to developing nanostructured Li_4_Ti_5_O_12_ and Li_4_Ti_5_O_12_/carbon nanocomposite to improve the rate performance for high-power Li-ion batteries, but there are still several challenges. First, it is often difficult to match the nano-dimension with the optimal crystallinity of the produced material through a low-temperature synthesis method. Second, the nanoparticles will result in low powder tap density, which consequently causes low volumetric energy density of the cell. Third, the interparticle contact resistance remains a main rate limiting factor, which is attributed to the aggregation of nanoparticles. Making Li_4_Ti_5_O_12_/carbon nanocomposites using carbon coating could improve the rate capability, but it also hinders Li-ion diffusion. Thus, the optimal carbon layer should be as thin as possible. Facile methods suitable for large-scale commercial production of this microscale/nanoscale hybrid materials with uniform carbon coating are still requested.

At this stage, it is possible to prepare LTO-based lithium-ion batteries competitively for the next generation of lithium batteries. For instance, Zaghib et al. [18] studied an 18650-type battery (capacity of 800 mAh) using carbon-coated LiFePO_4_ as a cathode and carbon-free Li_4_Ti_5_O_12_ derived from a solid-state reaction as an anode. This battery with a permanently stable capacity after 20,000 cycles at a charge rate of 10C and discharge rate of 5C was used in a real car with the charge time reduced to 5 min using a three-level charger in parallel (500 V, 125 A). More recently, batteries with LTO anodes and high-voltage cathodes have been proven to be safe with remarkable rate capability and cycle ability [19,20,23]. Already, some companies, such as Chongqi, Zhuhai, and Shenzhen of China, have used batteries comprised of Li_4_Ti_5_O_12_ as the anode in the EV power system which has driven more than 50,000 km and charged more than 2000 times. It gives evidence that it is now possible to prepare nanostructured LTO with high purity and small size dispersion, in synthesis processes that avoid aggregation.

Other issues, however, need to be improved. Most efforts have been focused on the performance of LTO. Doping is a low-cost strategy for this purpose, and so is the synthesis of composites of LTO with a conductive element. However, both strategies should be used simultaneously to search for a synergetic effect by the synthesis of a composite of doped LTO with a conductive material. More research on such synergetic effects is needed. For practical use, some other aspects should now be considered. The cost is an important parameter for the industrialization. In this respect, the liquid-state method and the sol–gel method needing many organic reagents in the process are more expensive than the solid-state process but allow for better control of the size of the nanoparticles. Other parameters are the tap density and the mass loading, considered only in a few works [416,530]. Further investigations should then focus on these parameters to further extend the industrialization application of Li_4_Ti_5_O_12_ in advanced energy storage devices.

## Figures and Tables

**Table 1:** Synthesis conditions of various types of nanosized LTO materials classified according to their dimensionality from 0D to 3D architectures.

Type	Particle	Method ^(a)^	Reagents ^(b)^	Particule Size (nm)	Ref.
0D	nanoparticle	CL	A-TiO_2_+LiOH	30	[119]
	nanosphere	HL	TiN+LiOH+PVP	dia. ~120	[39]
1D	nanorod	HT	A-TiO_2_+LiOH	dia. ~200	[122]
	nanowire	HT+IE	TiO_2_+NaOH+LiOH	dia. 50–100	[129]
	nanofiber	ES	LAA+TIP+PVP	dia. ~500	[135]
	nanotube	HR	TNBT+CTACl+LiOH	outer 11/inner 6	[54]
	nanoflake	HT	TBuT+EG+LiOH	200–400	[142]
	nanobelt	ES	TIP+LiNO_3_+DMF+PVP	dia. 100	[144]
2D	nanoplate	HT	TBuT+LiOH+BA	300 × 8	[152]
	nanosheet	EF+CL	H_0.68_Ti_1.83_O_4_+LiOH	400 × 2.5	[168]
	wave-like	HL	Li+DMEA+TBuT	400 × 10	[152]
3D	nanoflowers	HT	LiOH+H_2_O_2_+TNBT	-	[153]
	nanoporous	HT	A-TiO_2_+LiOH	pore dia. 2.6	[55]
	mesoporous	SG	TIP+Li+AC+PBI-b-PEO	pore = 20	[67]
	hierarchical	US+HT	TBuT+LiOH+CTAB	-	[158]
	nanotube arrays	TP	TiO_2_+LiOH	-	[159]
	nanobelt arrays	IE+HT	Ti foil+HCL+LIOH	-	[160]
	nanoflake arrays	TP+HT	TIP+LiOH	10–50	[161]
	nanosheet arrays	HT+TD	Ti foil+LiOH	~14	[169]
	nanowire arrays	HT+IE	Ti foil+LiOH	90	[163]
	nanowall arrays	HT	Ti foil+NaOH+LiOH	10–20	[164]

^(a)^ Synthesis methods: CL = chemical lithiation; HL = hydrolysis; HT = hydrothermal, ES = electrospinning; TP = templating; IE = ion exchange; EF = exfoliation; CL = chemical lithiation. ^(b)^ Reagent abbreviations: PVP = poly(vinyl pyrrolidone); LAA = lithium acetylacetonate; TIP = titanium isopropoxide; TNBT = titanium n-butoxide; CTACl = cetyltrimethyl ammonium chloride; TBuT = tetrabutyl titanate; EG = ethylene glycol; DMEA = N,N-dimethylethanolamine; BA = benzyl alcohol-NH_3_·H_2_O; PVDF = polyvinylidene fluoride; DMF = N,N-dimethylformamide; CTAB = hexadecyl trimethylammonium bromide; A-TiO_2_ = anatase titanium dioxide.

**Table 2:** Frequencies and assignments of Raman active modes of spinel Li_4_Ti_5_O_12_.

Raman Shift (cm^−1^)					Assignment	
Observed	From Ref. [174]	from Ref. [175]	From Ref. [176]	From Ref. [177]		
234	225	232	233	232	ν_b_(O−Li−O)	*F*_2g_^1^
265	272	271	-	271	ν_b_(O−Ti−O)	*F*_2g_^2^
347	340	347	348	355	ν_s_(Li^16d^O_6_)	*F*_2g_^3^
427	420	430	419	428	ν_s_(Li^8a^O_4_)	*E*_g_
517	514	520	-	6	overtone	-
674	675	671	670	672	ν_s_(TiO_6_)	*A*_1g_
754	-	749	748	730	ν_s_(TiO_6_)	*A*_1g_ shoulder

**Table 3:** Starting materials employed for the synthesis of Li_4_Ti_5_O_12_.

Synthesis	Ti Source ^(a)^	Li Source	Solution/Additive ^(b)^	Particle Size (nm)	Ref.
Solid-state reaction	TiO_2_	Li_2_CO_3_	acetone	>1000	[185]
Solid-state reaction	TiO_2_	Li_2_CO_3_	urea	400	[179]
Hydrolysis	TiN	LiOH∙H_2_O	H_2_O_2_+NH_3_	120	[39]
Solvothermal	TBT	LiOH∙H_2_O	water-ethanol (2:3)	800–1300	[186]
Spray pyrolysis	TIP	Li(CH_3_COO)	2-propanol (IPA)	120	[115]
Ball milling	A-TiO_2_	Li_2_CO_3_	ammonium salt	230	[93]
Spray drying	R-TiO_2_	Li_2_CO_3_	polyvinyl butyral	1000	[111]
Hydrothermal	TIP	LiOH∙H_2_O	ethanol-water	200–400	[110]
Wet chemistry	TiOCl_2_+NH_4_OH	Li(CH_3_COO)∙2H_2_O	water+oxalic acid	5000	[167]
Combustion	TiO_2_	LiOH∙H_2_O	methyl alcohol	30–40	[104]
Combustion	Ti(OH)_4_	LiNO_3_	HNO_3_	40–80	[103]
Combustion	R-TiO_2_	Li_2_CO_3_	cellulose/HNO_3_	-	[102]
Combustion	A-TiO_2_	LiNO_3_	cellulose/glycine	200–800	[101]
Ball milling	TIP	LiNO_3_	lactic acid/NH_4_NO_3_	200–300	[100]
Solid-state reaction	TiCl_4_	LiCl	70 wt.% oxalic acid	41 ^(c)^	[187]
Sol–gel	TIP	Li ethoxide	PBI-b-PEO	20–30	[67]
Soft-templating	TBT	Li(CH_3_COO)∙2H_2_O	2-methoxyethanol	18 ^(d)^	[188]
Microwave	TIP	dissolved Li metal	benzyl alcohol	150 ^(d)^	[189]
Precipitation	TBT	LiOH	DI water	10 ^(e)^	[190]
Sol–gel	TiCl_4_	LiOH∙H_2_O	ethanol+TEA	360	[191]
Hydrothermal	Ti foil	LiOH∙H_2_O	H_2_O_2_+DI water	~12	[192]
Solid-phase	H_2_TiO_3_	Li_2_CO_3_	-	800	[194]

^(a)^ TBT = tetrabutyl titanate (Ti(OC_4_H_9_)_4_); TIP = titanium(IV)isopropoxide (Ti(OC_3_H_7_)_4_); PBI-b-PEO = poly(isobutylene)-b-polyethylene oxide; R-TiO_2_ = rutile titanium dioxide; A-TiO_2_ = anatase titanium dioxide. ^(b)^ PBI-b-PEO = poly(isobutylene-b-polyethylene oxide); TEA = triethanolamine. ^(c)^ Crystallite size according to the Scherrer equation. ^(d)^ Average diameter of open pores. ^(e)^ Length of nanorods as primary particles.

**Table 4:** Preparation of LTO anode materials via solid-state reaction.

Ti Source	Li Source ^(a)^	Synthesis Procedure	Particle Size (µm)	Ref.
anatase TiO_2_	Li_2_CO_3_	mixed in acetone; 900 °C/10 h in air	~1.5	[43]
anatase TiO_2_	Li_2_CO_3_	ball milled; 800 °C/5 h in air, N_2_, H_2_/Ar	0.3–0.4	[205]
anatase TiO_2_	LiOAc	intermediate phase Li_2_TiO_3_	0.1	[231]
TiO(OH)_2_	Li_2_TiO_3_	Li:Ti = 0.85; 750–800 °C/6 h in air	1–5	[232]
TiO_2_+6% NaCl	LiOH	Li:Ti = 0.8; 800 °C/12 h in O_2_	1.8–2.3	[233]
Ti(C_4_H_9_O)_4_	LiOH·H_2_O	dissolved in ethyl alcohol 750 °C/5 h in air	-	[234]
rutile TiO_2_	Li_2_CO_3_	milling methanol; 800 °C/12 h in air	-	[45]
rutile TiO_2_	Li_2_CO_3_	ground with methanol; 800 °C/12 h in air	0.35	[235]
Ti foil	LiOH	30% (*w*/*w*) H_2_O_2_ aqueous solution; 130 °C/5 h	1/0.012	[192]
anatase TiO_2_	Li_2_CO_3_	ground with ethanol/3 h; 900 °C/5 h in air	0.4–0.5	[47]
anatase TiO_2_	Li_2_CO_3_	ball mill 400 rpm/4 h; 750 °C/12 h	≈0.7	[48]
TiO_2_	Li_2_CO_3_	ball mil/48 h with hexamethylene; 900 °C/24 h	-	[236]
TiO_2_	Li_2_CO_3_	phenolic resin+ethyl alcool slurry solution	0.2–0.5	[237]
H_2_TiO_3_	Li_2_CO_3_	direct mixing + sintering at 700 °C/6 h in Ar	0.8	[184]

^(a)^ LiOAc = lithium acetate dihydrate (CH_3_COOLi·2H_2_O).

**Table 5:** Comparison of the electrochemical performance of nanostructured LTO materials synthesized by solid-state reaction with different morphologies. Cycle number is given in parentheses.

Type ^(a)^	Reagents ^(b)^	Sintering (°C:h)	Particle Size (nm)	Specific Capacity (mAh g^−1^)	Ref.
NPs	A-TiO_2_+Li_2_CO_3_	800:2	40	101@0.1 A g^−1^ (500)	[119]
NPs	A-TiO_2_+LiOH+PAA	800:8	~300	133@10C (50)	[219]
MPs	A-TiO_2_+LiOH	800:12	~2000	114@10C (1065)	[233]
AGs	LiCl+TiCl_4_+OA	800:10	-	70@10C (200)	[187]
SpGs	TiO_2_+Li_2_CO_3_	800:5	~300	80@40C (5)	[205]
Sub-µ	R-TiO_2_+Li_2_CO_3_	800:36	700	35@30C (5)	[235]
Sub-µ	TiO_2_+Li_2_CO_3_	850:24	~600	93@5C (200)	[224]
SpGs	A-TiO_2_+Li_2_CO_3_	750:12	~700	60@10C (500)	[48]
NPs	A-TiO_2_+Li_2_CO_3_	800:3	162	139@10C (3)	[227]
NPs	TiCl_4_+H_2_C_2_O_4_+LiCl	800:10	200	70@10C (100)	[222]
NPs	TiO_2_+Li_2_CO_3_	800:20	200–500	170@1C (50)	[248]
AGs	A-TiO_2_+LiOAc	800:3	-	130@10C (10)	[249]
NPs	A-TiO_2_+Li_2_CO_3_	850:24	~500	212@1C (150)	[250]

^(a)^ NPs = nanoparticles; MPs = microparticles; AGs = aggregates; SpGs = spherical grains; Sub-µ = submicronic gains. ^(b)^ PAA = polyacrylate acid; OA = oxalic acid; LiOAc = lithium acetate dihydrate; A-TiO_2_ = anatase titanium dioxide; R-TiO_2_ rutile titanium dioxide.

**Table 6:** Examples of starting materials used in wet-chemical methods for the synthesis of LTO anodes ^(a)^.

Ti Source	Li Source	Complexing Agent	Solvent	Particle Size (nm)	Ref.
TBT	LiOAc	Triethanolamine	ethanol	223–264	[64]
TIP	LiOEt	PEG	ethanol	4–5	[285]
TBT	LiOAc	acetic acid	isopropyl alcohol	100	[62]
TIP	LiOH	*n*-octanol+HOP	acetonitrile	450	[89]
TET	Li_2_CO_3_	citric acid	TiH_4_O_4_+HNO_3_	300–650	[286]
TBT	LiOAc	lauric acid	alcohol	65	[287]
TBT	Li_2_CO_3_	oxalic acid	alcohol	200	[63]
TBT	LiOAc	glacial acetic acid	ethanol+water	400–800	[267]
TBT	LiOAc	propionic acid	alcohol	150 × 60	[283]
TIP	LiOAc	P123 copolymer	ethanol	90–110	[288]
TBT	LiOAc	PVP	isopropyl alcohol	20–75	[279]

^(a)^ TBT = tetrabutyl titanate; LiOEt = lithium ethoxide; PEG = polyethylene glycol; TIP = titanium isopropoxide; HOP = hydroxypropyl cellulose; TET = tetraethoxytitanium; P123 = non-ionic surfactant (PEO)_20_(PPO)_70_(PEO)_20_ triblock copolymer; PVP = polyvinylpyrrolidone; LiOAc = lithium acetate (CH_3_COOLi·2H_2_O).

**Table 7:** Comparison of the electrochemical performance of nanostructured LTO materials synthesized by sol–gel route with different morphologies. Cycle number is given in parentheses.

Type ^(a)^	Reagents ^(b)^	Sintering (°C:h)	Particle Size (nm)	Specific Capacity (mAh g^−1^)	Ref.
NCs	TBT+ LiOAc	800:10	65	163@1C (50)	[287]
NCs	TBT+LiOAc	700:12	136	157@10C (1)	[303]
TF	TBT+LiOAc	650	18 (pores)	148@64C (3000)	[188]
NCs	TIP+LiOAc	700:5	100	108@40C (250)	[288]
NPs	TIP+LiOH	800:10	450	160@0.1C (80)	[89]
NSs	TBT+LiOAc	800:1	150 × 60	56@20C (80)	[283]
NPs	TBT+Li_2_CO_3_	750:5	200–500	160@1C (1000)	[263]
NPs	TBT+LiOAc	800:12	300	60@10C (50)	[178]
NPs	TIP+LiOAc	800:12	200	160@0.2 (30)	[291]
NPs	TBT+Li_2_CO_3_	750:5	~100	145@5C (400)	[301]
NPs	TBT+Li_2_CO_3_	800:20	200	150@0.5 mA cm^−2^ (35)	[63]
NPs	TIP+LiOAc	800:2	50–200	170@0.2C (200)	[304]
NPs	TBT+LiOAc+HCl	900:2	240	302@1C (3)	[305]
NPs	TIP+LiOH+CA	800:2	-	70@0.1C (3)	[306]
NPs	TIP+LiOAc	800:12	5–14	214@1C (150)	[307]
NCs	TBT+LiOH+EtOH	600:2	50–200	90@40C (100)	[308]

^(a)^ NCs = nanocrystals; TF = thin film; NPs = nanoparticles; NSs = nanostructures; NCs = nanocables. ^(b)^ TBT = tetrabutyl titanate; LiOAc = lithium acetate dihydrate. TIP = titanate isopropoxide.

**Table 8:** Preparation of the LTO spinel phases by hydrolysis route.

Ti Source	Li Source	Solution	Process	Ref.
TBT	LiOH	DI water/ethanol	stirring for 24 h	[106]
TBT+ammonia	LiOH	glycerol/water	formation of clear solution	[314]
TBT	LiOAc	DI water	drying at 90 °C for 5 h	[313]
TBT	LiOH	DI water	slurry dried at 100 °C + grinding	[315]
TBT	Li metal	DMEA	H_2_ byproduct is used as fuel	[152]
TiN	LiOH	DI water/H_2_O_2_	stirring with ammonia	[39]

**Table 9:** Comparison of the electrochemical performance of nanostructured LTO materials synthesized by mechano-chemical method with different morphologies. Cycle number is given in parentheses.

Type ^(a)^	Reagents ^(b)^	Sintering (°C:h)	Particle Size (nm)	Specific Capacity (mAh g^−1^)	Ref.
NPs	A-TiO_2_+LiOAc+AG	800:10	50–400	153@1C (1000)	[316]
Nc1	A-TiO_2_+Li_2_CO_3_+EtOH	750:12	<1 µm	108@10C (500)	[48]
Nc1	A-TiO_2_+Li_2_CO_3_+EtOH	750:12	100–200	260@88 A g^−1^ (160)	[241]
NPs	TiO_2_+Li_2_CO_3_+EA	800:20	200–500	171@1C (50)	[325]
C-Ncs	TiO_2_+Li_2_CO_3_+EA	800:10	50–400	142@1C (1000)	[318]
NPs	R-TiO_2_+Li_2_CO_3_	800:3	~400	150@4C (3)	[328]
HEBM	A-TiO_2_+LiOH+GL	800:12	150	140@1C (1)	[92]
HEBM	A-TiO_2_+Li_2_CO_3_+PAA	800:3	146	120@20C (3)	[93]
NPs	TOS+LiOH	500:2.5	~1 µm	150@1.5 A g^−1^ (50)	[46]
NPs	TBT+Li_2_CO_3_+NH_3_	800:7	200–500	175@0.5C (300)	[312]
NPs	TiCl_4_+LiOAc+CA	800:10	500	141@30 mA g^−1^ (30)	[326]
NPs	A-TiO_2_+Li_2_CO_3_	800:8	620	135@10C (10)	[329]
MPs	a-TiO_2_+Li_2_CO_3_+Et	800:12	1 µm	160@0.1C (100)	[330]

^(a)^ NPs = nanoparticles; Nc1 = LTO-Li_2_TiO_3_ nanocomposite; Nc2 = LTO-ZnO nanocomposite; C-Ncs N-doped carbon LTO nanocomposites; HEBM = high-energy ball-milled particles; MPS = micron-sized particles. ^(b)^ A-TiO_2_ = anatase TiO_2_; R-TiO_2_ = rutile TiO_2_; a-TiO_2_ = amorphous TiO_2_; AG = acetyl glucosamine; EtOH = ethanol; EA = ethyl alcohol; GL = glycol; PAA = polycarboxylic acid ammonium salt; TOS = titanium(IV) oxide sulfate sulfuric acid hydrate; LiOAc = lithium acetate (CH_3_COOLi·2H_2_O).

**Table 10:** Electrochemical performances of nanostructured LTO materials synthesized by combustion method. Cycle number is given in parentheses.

Reagents ^(a)^	Chelate ^(b)^	Sintering (°C:h)	Particle Size (nm)	Specific Capacity (mAh g^−1^)	Ref.
TiO_2_+LiOAc	PVP	800:8	200–500	142@5C (20)	[280]
TiO_2_+LiNO_3_	glycine	750:2	40–80	175@1C (1)	[217]
TIP+LiNO_3_	lactic acid	300	200–300	132@1C (100)	[100]
TBT+LiNO_3_	glycine	850:17	50–100	187@1C (50)	[99]
R-TiO_2_+Li_2_CO_3_	cotton fiber	800:5	-	130@2C (50)	[102]
TBT+LiNO_3_	glycine	800:5	50–350	111@10C (150)	[341]
TiNt+LiNO_3_	glycine	800:½	50–100	159@1C (200)	[342]
TiNt+LiNO_3_	glycine	800	<150	170@0.5C (100)	[97]

^(a)^ TIP = titanium(IV) isopropoxide (Ti(OC_3_H_7_)_4_); TBT = tetrabutyl titanate (Ti(OC_4_H_9_)_4_); R-TiO_2_ = rutile TiO_2_; TiNt = titanyl nitrate (TiO(NO_3_)_2_); LiOAc = lithium acetate (CH_3_COOLi·2H_2_O). ^(b)^ PVP = polyvinylpyrrolidone.

**Table 11:** Electrochemical performances of nanostructured LTO materials synthesized by hydrothermal method. Cycle number is given in parentheses.

Reagents ^(a)^	HT Reaction (°C:h)	Sintering (°C:h)	Particle Size (nm)	Specific Capacity (mAh g^−1^)	Ref.
TIP+LiOH+EG	170:36	500:2	200	152@8C (100)	[146]
TIP+LiOH+H_2_O_2_	150:¼	500:2	~100	150@0.2C (30)	[351]
TBT+LiOH	180:12	600:3	200–300	155@0.1C (50)	[50]
TBT+LiOH	180:12	550:6	300–600	150@20C (5)	[345]
TBT+LiOH	120	500:6	6–11 ^(b)^	156@0.1C (25)	[54]
TBT/LiOH	180:24	500:10	nanosheets	115@5C (50)	[354]
Ti(SO_4_)_2_+LiOH	100:20	800:2	500	160@0.2C (70)	[53]
TBT+LiOH	180:36	66:6	nanoflakes	127@0.2C (100)	[355]
H_2_TiO_3_+LiOH	170:24	-	-	126@0.1C (30)	[376]
TBT+LiOEt	150:10	400–500	<100	140@2C (1)	[195]
TBT+LiOH	180:36	700:6	nanosheets	144@ 2C (600)	[149]
Na_2_Ti_3_O_7_+LiOH	180:24	450:1	500 × 40	0.55@3 mA cm^−2^ (1000) ^(c)^	[145]
H_2_Ti_3_O_7_+LiOH	150:12	500:4	50 × 10	95@5C (300)	[129]
TBT+LiOAc	170:72	1050:1	30–40	80@60C (5)	[362]
TBT+LiOAc+PVP	160:24	800:10	20–75	125@20C (50)	[279]
TiO_2_(P25)+LiOH	120:24	400:4	nanosheets	126@20C (3000)	[353]

^(a)^ TIP = titanium(IV) isopropoxide (Ti(OC_3_H_7_)_4_); TBT = tetrabutyl titanate (Ti(OC_4_H_9_)_4_); LiOEt = lithium ethoxide (CH_3_CH_2_OLi); LiOAc = lithium acetate (CH_3_COOLi·2H_2_O); PVP = polyvinylpyrrolidone; EG = ethylene glycol. ^(b)^ LTO nanotube diameter. ^(c)^ Specific discharge capacity expressed in mAh cm^−2^.

**Table 12:** Electrochemical performances of nanostructured LTO materials synthesized by solvothermal method. Cycle number is given in parentheses.

Reagents ^(a)^	HT Reaction (°C:h)	Sintering (°C:h)	Particle Size (nm)	Specific Capacity (mAh g^−1^)	Ref.
TBT+LTB+P123	170:20	400:10	35–55	140@100C (1000)	[387]
TIP+LiOH+EtOH	180:10	600:3	20	125@1C (100)	[383]
TBT+LIOH+EG	180:16	600:8	100–200	160@10C (1000)	[200]
TBT+LiOAc+EtOH	-	550:2	0.5–1 µm	92@20C (100)	[60]
TIP+Li+BA	250:48	750:1	1–2 µm	148@1C (200)	[203]
TBT+LiOH+EtOH	200:24	600:3	200	170@0.5C (100)	[384]
TiO_2_+LiOH+EtOH	180:16	700:4	~660	106@10C (100)	[180]
TBL+LiOH+EtOH	200:12	500:3	13	105@10C (10)	[386]

^(a)^ TBT = tetrabutyl titanate (Ti(OC_4_H_9_)_4_); LTB = lithium tert-butoxide (LiOC(CH_3_)_3_); TIP = titanium(IV) isopropoxide (Ti(OC_3_H_7_)_4_); EtOH = ethanol; EG = ethylene glycol (C_2_H_6_O_2_); BA = benzyl alcohol (C_7_H_8_O); TBL = titanium(IV) bis(ammonium lactato)dihydroxide ([CH_3_CH(O-)CO_2_NH_4_]_2_Ti(OH)_2_).

**Table 13:** Electrochemical performances of nanostructured LTO materials synthesized by templating method. Cycle number is given in parentheses.

Reagents ^(a)^	Template	Particle Size (nm)	Specific Capacity (mAh g^−1^)	Ref.
TIP+Li+EtOH+Tol	carbon black	~70	160@1C (100)	[397]
TIP+Li+EtOH+OA	PI-*b*-PEO	mesoporous	115@10C (500)	[155]
TiCl_4_+LiOAc	PMMA	microporous	155@0.5 mA cm^−2^	[167]
TiCl_4_+LiOH	ZnO nanorods	nanotube arrays	110@40C (200)	[399]
R-TiO_2_+Li_2_CO_3_	PFR	~800	60@20C (5)	[154]
TiO_2_+LiOH	ZnO nanorods	nanotubes ^(b)^	144@10C (500)	[159]
TBT+LiOAc	NPGF	fibers	146@1C (100)	[134]
TOT+LiOAc+EtOH	whatman paper	250–300	88@5C (1100)	[87]

^(a)^ TIP = titanium(IV) isopropoxide (Ti(OC_3_H_7_)_4_); TBT = tetrabutyl titanate (Ti(OC_4_H_9_)_4_); EtOH = ethanol; Tol = toluene; OA = oxalic acid; PI-*b*-PEO = polyisoprene-block-poly(ethylene oxide; PMMA = poly(methyl methacrylate); PFR = phenol–formaldehyde resin; NPGF = nitrogen-doped mesoporous graphene fibers; TOT = tetraethyl orthotitanate (Ti(OC_2_H_5_)_4_; R-TiO_2_ = rutile titanium dioxide. ^(b)^ nanotubes with diameter of 200–300 nm and shell thickness 20–30 nm.

**Table 14:** Electrochemical performances of nanostructured LTO materials synthesized by spray-drying method. Cycle number is given in parentheses.

Reagents ^(a)^	Sintering (°C:h)	Particle Size (µm)	Specific Capacity (mAh g^−1^)	Ref.
TIP+LiOH+H_2_O_2_	675:16	1–5	75@20C (100)	[432]
TiO_2_ (P25)+LiOH	750:10	~5	140@1C (200)	[437]
TiO_2_+LiOH+PEG	850:2	0.6	120@4C (10)	[430]
TBT+LiOH	650:16	1–1.5	100@20C (100)	[403]
TIP+Li_2_CO_3_+OA	800:12	0.2–0.4	120@2C (200)	[407]
A-TiO_2_+LiOH	850:12	0.3–0.5	40@20C (2)	[410]
A-TiO_2_+Li_2_CO_3_+APC	850:5	0.2	110@5C (100)	[404]
A-TiO_2_+Li_2_CO_3_	800:12	10–20	145@1C (250)	[405]
n-TiO_2_+Li_2_CO_3_	850:12	1.18	161@0.5C (1)	[406]
TIP+H_2_O_2_+LiOH+NH_3_	700:16	1–2	135@5C (100)	[408]
A-TiO_2_+Li_2_CO_3_	850:8	10	173@1C (400)	[416]
TBT+LiOH+AA	750:4	0.2	192@2C (20)	[424]
TIP+LiNO_3_+IsoP	800:2	3.5–5.5	50@10C (10)	[440]

^(a)^ TIP = titanium(IV) isopropoxide (Ti(OC_3_H_7_)_4_); PEG = polyethylene glycol;TBT = tetrabutyl titanate (Ti(OC_4_H_9_)_4_); IsoP = isopropanol; APC = ammonium polycarbonate; n-TiO_2_ = nano titatium dioxide (20 nm); AA = acetic acid.

**Table 15:** Electrochemical performances of nanostructured LTO materials synthesized by spray-pyrolysis technique. Cycle number is given in parentheses.

Reagents ^(a)^	Sintering (°C:h)	Particle Size (nm)	Specific Capacity (mAh g^−1^)	Ref.
TBT+LiOAc+EtOH	800:1	800	146@10C (500)	[450]
LNT-EtOH+TIP-EHA	-	~10	146@1C (450)	[77]
Li-acac+TIP+EHA+xylene	-	~14	100@25C (10)	[445]
LTN+TIP in water	800:12	700	170@0.1C (70)	[72]
Li-acac+TIP+EHA	none	~10	87@1C (200)	[77]
LiOH+TIP+LA	800	~1000	162@1C (100)	[443]
THE+LiOAc+MeOH	none	20	140@ 1C (75)	[451]
TBT+LNT+EG	none	600	165@0.1C (50)	[452]
TIP+LNT	800:3	700	175@1C (100)	[453]
TIP+LNT	700:3	300	157@0.1C (50)	[449]

^(a)^ TBT = tetrabutyl titanate (Ti(OC_4_H_9_)_4_); TIP = titanium(IV) isopropoxide (Ti(OC_3_H_7_)_4_); EtOH = ethanol; LNT = LiNO_3_; EHA = ethylhexanoic acid; Li-acac = lithium acetylacetonate; LA = lactic acid; THE = titanium (IV) 2-ethylhexanoate; MeOH = methanol; EG = ethylene glycol.

**Table 16:** Detail of the electrospinning synthesis and electrochemical performance of LTO nanofibers. Cycle number is given in parentheses.

Ti Source ^(a)^	Li Source ^(b)^	Solvent ^(c)^	Polymer ^(d)^	Performance ^(e)^ (mAh g^−1^)	Ref.
TIP	LiOAc	DMF+AA	PVAc	75@10C (1)	[476]
TIP	LiOAc	EtOH+AA	PVP+Pluronic	123@5C (100)	[474]
TIP	LiAAc	isoproxyl alcohol	F127	85@1.5C (30)	[95]
TiOAc	LiOAc	EtOH	PVP	165@1C (100)	[133]
nano-LTO	-	DMF	PVP	115@2C (1)	[464]
TBT	LiOAc	ethanol+AAc+AA	PAN	139@5C (500)	[474]
TIP	LiAAc	isoproxyl alcohol	PVP	120@10C (100)	[140]
TIP	LiOAc	EtOH+AA	PVP	120@1C (200)	[471]
TBT	LiAAc	EtOH+AA	PVP	60@25C (1)	[468]
TIP	LiOAc	EtOH+AA	PVP	140@10C (30)	[137]
TIP	LAAc	EtOH+AA	PVP	132@20C (300	[135]

^(a)^ TIP = Titanium(IV) isopropoxide, Ti[OCH(CH_3_)_2_]_4_; TBT = tetrabutyl titanate, Ti(C_4_H_9_O)_4_. ^(b)^ LiOAc = lithium acetate dihydrate, Li(CH_3_CO_2_)·2H_2_O; LiAAc = lithium acetylacetonate (LiC_5_H_7_O_2_). ^(c)^ DMF = N,N-dimethyl formamide; EtOH = ethanol; AA = acetic acid; AAc = acethylacetone C_5_H_8_O_2_. ^(d)^ PVAc = polyvinyl acetate; PVP = polyvinylpyrrolidone; PAN = polyacrylonitrile. ^(e)^ 1C = 175 mA g^−1^.

**Table 17:** Electrochemical performances of nanostructured LTO materials synthesized by using two-step ion exchange technique. Cycle number is given in parentheses.

First Step	Second Step	Particle ^(a)^	Specific Capacity (mAh g^−1^)	Ref.
TiO_2_/NaOH/HNO_3_	H-TiO_x_/LiOH	NTs 7 nm	110@1C (30)	[130]
TiO_2_/Na_2_CO_3_/HCl	H_2_Ti_3_O_7_/LiNO_3_	1–2 µm	190@5C (3)	[478]
TiO_2_/NaOH/HCl	H_2_Ti_2_O_5_/LiOH	NFs	118@5C (4)	[477]
Ti/NaOH+NaCl/HCl	H_2_Ti_2_O_5_/LiOH	NW arrays	125@30C (5000)	[375]
-	TiO_2_/NaOH/LiOH	10–50 nm	60@50C (1)	[141]
TiO_2_/NaOH/HCl	H_2_Ti_3_O_7_/LiOH	NWs	145@0.2C (50)	[132]
Ti/NaOH/HCl	H-TiO_x_/LiOH	arrays 7 µm	0.2@1 mA cm^−2^ (1000) ^(b)^	[165]
-	TiO_2_/NaOH/LiOH	20–30 nm	148@10C (200)	[479]
TiO_2_/NaOH/HCl	H_2_Ti_2_O_5_/LiOH	NWs 80 nm	125@10C (100)	[480]

^(a)^ NT = nanotube; NF = nanofiber; NW = nanowire; ^(b)^ Areal specific capacity in mAh cm^−2^.

**Table 18:** Advantages and drawbacks of LTO synthesis methods.

Synthesis Method	Advantages	Disadvantages
Solid state reaction	Simple method; use of starting materials at low cost; large-scale production	Need for high-temperature treatment; formation of unwanted byproducts; difficult to adjust particle size, particle size distribution, and lack of stoichiometric control
Hydrothermal/solvothermal	Production of nanomaterials; well controlled through liquid-phase reaction	Expensive method; time-consuming; not feasible for mass production; organic residues appear with organic solvents
Sol–gel	Low-cost; offer products with a homogeneous distribution of uniform, submicron-size particles with good stoichiometric control	Use of chelating agent for the control of nanostructure and morphology; difficult to use for mass production
Solution-combustion		Needs additional post-annealing treatment
Microwave	Fast synthesis route; low-temperature process; rapid volumetric heating, high reaction rate; small particle size	Purity and the crystallinity of the products are dependent upon the irradiation power and time; difficult to use for mass production
Spray pyrolysis	Direct synthesis of powder from precursor solutions; products with high purity and homogeneity; industrially scalable	Difficult to control the morphology and chemical composition
Electrospinning	Simple, versatile, and low-cost technology to produce well-defined fibers at the nanoscale	Two-step procedure; expensive
Templating	Formation of mesoporous structures; monodispersed products	Removal of the templates after synthesis may damage the desired configurations; not suitable for large-scale production
Molten salt	Low temperature and short time; suitable for small particle size	Not suitable for large-scale production

## Abbreviations

AA	acetic acid
AC	activated carbon
AHTO	amorphous hydrous titanium oxide
BET	Brunauer-Emmett-Teller
CA	citric acid
CNT	carbon nanotube
CTAB	cetyl trimethyl ammonium bromide
CTAC	cetyltrimethyl ammonium chloride
DI water	deionized water
DMEA	2-dimethylaminoethanol
DTA	differential thermal analysis
EDTA	ethylene diamine tetraacetic acid
EtOH	ethanol
FSP	flame spray pyrolysis
HEBM	high-energy ball milling
HOP	hydroxypropyl
HTS	hydrothermal synthesis
LiAAc	lithium acetylacetonate
LIB	lithium-ion battery
LiOAc	lithium acetate dihydrate
LTH	lithium titanate oxide hydrate
MA	malic acid
MeOH	methanol
MWCNT	multiwalled carbon nanotube
MWHT	microwave-assisted hydrothermal
PAA	polyacrylate acid
PAN	polyacrylonitrile
PBI-b-PEO	poly(isobutylene)-b-polyethylene oxide
PEG	polyethylene glycol
PF	phenol-formaldehyde
PVA	polyvinyl alcohol
PVP	polyvinylpyrrolidone
rGO	reduced graphene oxide
SD	Spray drying
SDBS	sodium dodecyl benzene sulfonate
SEI	solid electrolyte interphase
SEM	scanning electron microscopy
SG	space group
SIB	sodium-ion battery
SOR	sodium–organic reduction reagent
SSR	solid-state reaction
TEA	triethanolamine
TBT	tetrabutyl titanate
TBOT	titanium tetrabutoxide
TEA	triethanolamine
TET	tetraethoxytitanium
TG	thermogravimetry analysis
TIP	titanium(IV) isopropoxide
TNBT	titanium n-butoxide
XRD	X-ray diffraction

## References

[1] Mauger A., Julien C.M., Goodenough J.B., Zaghib K. (2020). Tribute to Michel Armand: From rocking chair—Li-ion to solid-state lithium batteries. J. Electrochem. Soc..

[2] Mauger A., Xie H., Julien C.M. (2016). Composite anodes for lithium-ion batteries: Status and trends. AIMS Mater. Sci..

[3] Jonker G.H. Compounds in the system Li_2_O–TiO_2_ and their stability. Proceedings of the Third International Symposium on the Reactivity of Solids.

[4] Deschanvres A., Raveau B., Sekkal Z. (1971). Mise en évidence et étude cristallographique d’une nouvelle solution solide de type spinelle Li_1+x_Ti_2−x_O_4_, 0 ≤ x ≤ 0.33. Mater. Res. Bull..

[5] Johnston D.C., Prakash H., Zachariasen W.H., Viswanathan R. (1973). High temperature superconductivity in the Li-Ti-O ternary system. Mater. Res. Bull..

[6] Murphy D.W., Cava R.J., Zahurak S.M., Santoro A. (1983). Ternary Li_x_TiO_2_ phases from insertion reaction. Solid State Ion..

[7] Colbow K.M., Dahn J.R., Haering R.R. (1989). Structure and electrochemistry of the spinel oxides LiTi_2_O_4_ and Li_4/3_Ti_5/3_O_4_. J. Power Sources.

[8] Ferg E., Gummow R.J., de Kock A. (1995). Spinel anodes for lithium-ion batteries. J. Electrochem. Soc..

[9] Amatucci G.G., Badway F., Du Pasquier A., Zheng T. (2001). An asymmetric hybrid nonaqueous energy storage cell. J. Electrochem. Soc..

[10] Ohzuku T., Ueda A., Yamamoto N. (1995). Zero-strain material of Li[Li_1/3_Ti_5/3_]O_4_ for rechargeable lithium cells. J. Electrochem. Soc..

[11] Sun X., Radovanovic P.V., Cui B. (2015). Advances in spinel Li_4_Ti_5_O_12_ anode materials for lithium-ion batteries. New J. Chem..

[12] Liu H., Zhu Z., Huang J., He X., Chen Y., Zhang R., Lin R., Li Y., Yu S., Xing X. (2019). Elucidating the limit of Li insertion into the spinel Li_4_Ti_5_O_12_. ACS Mater. Lett..

[13] Sugiyama J., Umegaki I., Uyama T., McFadden R.M.L., Shiraki S., Hitosugi T., Salman Z., Saadanoui H., Morris G.D., MacFarlane W.A. (2017). Lithium diffusion in spinel Li_4_Ti_5_O_12_ and LiTi_2_O_4_ films detected with ^8^Li β-NMR. Phys. Rev. B.

[14] Huang Q., Yang Z., Mao J. (2017). Mechanisms of the decrease in low-temperature electrochemical performance of Li_4_Ti_5_O_12_-based anode materials. Sci. Rep..

[15] Jung H.G., Jang M.W., Hassoun J., Sun Y.K., Scrosati B. (2011). A high-rate long-life Li_4_Ti_5_O_12_/Li[Ni_0.45_Co_0.1_Mn_1.45_]O_4_ lithium-ion battery. Nat. Commun..

[16] Wang W., Choi D., Yang Z. (2013). Li-ion battery with LiFePO_4_ cathode and Li_4_Ti_5_O_12_ anode for stationary energy storage. Metall. Mater. Trans. A.

[17] Yang C., Hu H., Lin S.J., Chien W. (2014). Electrochemical performance of V-doped spinel Li_4_Ti_5_O_12_/C composite anode in Li-half and Li_4_Ti_5_O_12_/LiFePO_4_–full cell. J. Power Sources.

[18] Zaghib K., Dontigny M., Guerfi A., Charest P., Rodrigues I., Mauger A., Julien C.M. (2011). Safe and fast-charging Li-ion battery with long shelf life for power applications. J. Power Sources.

[19] Behi H., Karimi D., Behi M., Jaguemont J., Ghanbarpour M., Behnia M., Berecibar M., Van Mierlo J. (2020). Thermal management analysis using heat pipe in the high current discharging of lithium-ion battery in electric vehicles. J. Energy Storage.

[20] Behi H., Karimi D., Gandoman F.H., Akbarzadeh M., Khaleghi S., Kalogiannis T., Hosen M.S., Jaguemont J., Van Mierlo J., Berecibar M. (2021). PCM assisted heat pipe cooling system for the thermal management of an LTO cell for high-current profiles. Case Stud. Therm. Eng..

[21] Behi H., Karimi D., Kalogiannis T., He J., Patil M.S., Muller J.-D., Haider A., Mierlo J.V., Berecibar M. (2022). Advanced hybrid thermal management system for LTO battery module under fast charging. Case Stud. Therm. Eng..

[22] Yang W., Zhang M., Ma S., Luo Y., Zhang B., Wang S., Yan L., Tong Z., Lu T., Zhou Y.-N. (2023). Li_4_Ti_5_O_12_-based battery energy storage system with dual-phase cathode. Energy Technol..

[23] Piao N., Wang P.-F., Chen L., Deng T., Fan X., Wang L., He X. (2023). Nonflammable all-fluorinated electrolytes enabling high-power and long-life LiNi_0.5_Mn_1.5_O_4_/Li_4_Ti_5_O_12_ lithium-ion batteries. Nano Energy.

[24] Wolfenstine J., Lee U., Allen J.L. (2006). Electrical conductivity and rate-capability of Li_4_Ti_5_O_12_ as a function of heat-treatment atmosphere. J. Power Sources.

[25] Wolfenstine J., Allen J.L. (2008). Electrical conductivity and charge compensation in Ta doped Li_4_Ti_5_O_12_. J. Power Sources.

[26] Thackeray M.M., Amine K. (2021). Li_4_Ti_5_O_12_ spinel anodes. Nat. Energy.

[27] Vikram Babu B., Vijaya Babu K., Tewodros Aregai G., Seeta Devi L., Madhavi Latha B., Sushma Reddi M., Samatha K., Veeraiah V. (2018). Structural and electrical properties of Li_4_Ti_5_O_12_ anode material for lithium-ion batteries. Results Phys..

[28] Wagemaker M., Simon D.R., Kelder E.M., Schoonman J., Ringpfeil C., Haake U., Lützenkirchen-Hecht D., Frahm R., Mulder F.M. (2006). A kinetic two-phase and equilibrium solid solution in spinel Li_4+x_Ti_5_O_12_. Adv. Mater..

[29] Borghols W.J.H., Wagemaker M., Lafont U., Kelder E.M., Mulder F.M. (2009). Size effects in the Li_4+x_Ti_5_O_12_ spinel. J. Am. Chem. Soc..

[30] Kavan L., Prochazka J., Spitler T.M., Kalbac M., Zukalova M.T., Drezen T., Gratzel M. (2003). Li insertion into Li_4_Ti_5_O_12_ (spinel): Charge capability vs. particle size in thin-film electrodes. J. Electrochem. Soc..

[31] Zhang H., Yang Y., Xu H., Wang L., Lu X., He X. (2022). Li_4_Ti_5_O_12_ spinel anode: Fundamentals and advances in rechargeable batteries. InfoMat.

[32] Yi T.-F., Yang S.-Y., Xie Y. (2015). Recent advances of Li_4_Ti_5_O_12_ as a promising next generation anode material for high power lithium-ion batteries. J. Mater. Chem. A.

[33] Zhao B., Ran R., Liu M., Shao Z. (2015). A comprehensive review of Li_4_Ti_5_O_12_-based electrodes for lithium-ion batteries: The latest advancements and future perspectives. Mater. Sci. Eng. R.

[34] Zhu G.N., Wang Y.G., Xia Y.Y. (2012). Ti-Based compounds as anode materials for Li-ion Batteries. Energy Environ. Sci..

[35] Yuan T., Tan Z., Ma C., Yang J., Ma Z.-F., Zheng S. (2017). Challenges of spinel Li_4_Ti_5_O_12_ for lithium-ion battery industrial applications. Adv. Energy Mater..

[36] Yan H., Zhang D., Lu Q., Duo X., Sheng X. (2021). A review of spinel lithium titanate (Li_4_Ti_5_O_12_) as electrode material for advanced energy storage devices. Ceram. Int..

[37] Ezhyeh Z., Khodaei M., Torabi F. (2023). Review on doping strategy in Li_4_Ti_5_O_12_ as an anode material for lithium-ion batteries. Ceram. Int..

[38] Shen Y., Eltzholtz J., Iversen B. (2013). Controlling size, crystallinity, and electrochemical performance of Li_4_Ti_5_O_12_ nanocrystals. Chem. Mater..

[39] Wang C., Wang S., Tang L., He Y.-B., Gan L., Li J., Du H., Li B., Lin Z., Kang F. (2016). A robust strategy for crafting monodisperse Li_4_Ti_5_O_12_ nanospheres as superior rate anode for lithium ion batteries. Nano Energy.

[40] Lin C. (2017). Improvements of Li_4_Ti_5_O_12_ anode material for lithium-ion batteries. Mater. Res. Found..

[41] Meng W., Xu Y., Yan B. (2018). In situ nano-sized spinel Li_4_Ti_5_O_12_ powder fabricated by a one-step roasting process in molten salts. J. Alloys Compd..

[42] Chang-Jian C.W., Cho E.C., Huang J.H., Huang J.H., Chou J.A., Ho B.C., Lee K.C., Hsiao Y.S. (2019). Spray-drying synthesis of Li_4_Ti_5_O_12_ microspheres in pilot scale using TiO_2_ nanosheets as starting materials and their application in high-rate lithium ion battery. J. Alloys Compd..

[43] Shenouda A.Y., Murali K.R. (2008). Electrochemical properties of doped lithium titanate compounds and their performance in lithium rechargeable batteries. J. Power Sources.

[44] Panero S., Reale P., Ronci F., Rossi Albertini V., Scrosati B. (2000). Structural and electrochemical study on Li(Li_1/3_Ti_5/3_)O_4_ anode material for lithium ion batteries. Ionics.

[45] Ge H., Li N., Li D., Dai C., Wang D. (2008). Electrochemical characteristics of spinel Li_4_Ti_5_O_12_ discharged to 0.01 V. Electrochem. Commun..

[46] Lai C., Wu Z., Zhu Y., Wu Q., Liang Li L., Wang C. (2013). Ball-milling assisted solid-state reaction synthesis of mesoporous Li_4_Ti_5_O_12_ for lithium-ion batteries anode. J. Power Sources.

[47] Chandrasekhar J., Dhananjaya M., Hussain O.M., Mauger A., Julien C.M. (2021). Enhanced electrochemical performance of Li_4_Ti_5_O_12_ by niobium doping for pseudocapacitive applications. Micro.

[48] Wang Y., Zhou A., Dai X., Feng L., Li J., Li J. (2014). Solid-state synthesis of submicron-sized Li_4_Ti_5_O_12_/Li_2_TiO_3_ composites with rich grain boundaries for lithium ion batteries. J. Power Sources.

[49] Sarantuya L., Sevjidsuren G., Zltantsog P., Tsogbadrakh N. (2018). Synthesis, structure and electronic properties of Li_4_Ti_5_O_12_ anode material for lithium ion batteries. Solid State Phenom..

[50] Zhang E., Zhang H. (2019). Hydrothermal synthesis of Li_4_Ti_5_O_12_-TiO_2_ composites and Li_4_Ti_5_O_12_ and their applications in lithium-ion batteries. Ceram. Int..

[51] Ge H., Chen L., Yuan W., Zhang Y., Fan Q., Osgood H., Matera D., Song X.-M., Wu G. (2015). Unique mesoporous spinel Li_4_Ti_5_O_12_ nanosheets as anode materials for lithium-ion batteries. J. Power Sources.

[52] Tang Y., Yang L., Fang S., Qiu Z. (2009). Li_4_Ti_5_O_12_ hollow microspheres assembled by nanosheets as an anode material for high-rate lithium ion batteries. Electrochim. Acta.

[53] Yan H., Zhu Z., Zhang D., Li W., Lu Q. (2012). A new hydrothermal synthesis of spherical Li_4_Ti_5_O_12_ anode material for lithium-ion secondary batteries. J. Power Sources.

[54] Lee S.C., Lee S.M., Lee J.W., Lee J.B., Han S.S., Lee H.C., Kim H.J. (2009). Spinel Li_4_Ti_5_O_12_ nanotubes for energy storage materials. J. Phys. Chem. C.

[55] Shen L., Yuan C., Luo H., Zhang X., Xu K., Xia Y. (2010). Facile synthesis of hierarchically porous microspheres for high rate lithium ion batteries. J. Mater. Chem..

[56] Shen L., Yuan C., Luo H., Zhang X., Yang S., Lu X. (2011). *In situ* synthesis of high-loading Li_4_Ti_5_O_12_–graphene hybrid nanostructures for high rate lithium ion batteries. Nanoscale.

[57] Wang Y., Rong H., Li B., Xing L., Li X., Li W. (2014). Microemulsion-assisted synthesis of ultrafine Li_4_Ti_5_O_12_/C nanocomposite with oleic acid as carbon precursor and particle size controller. J. Power Sources.

[58] Qian K., Tang L., Wagemaker M., He Y.-B., Liu D., Li H., Shi R., Li B., Kang F. (2017). A facile surface reconstruction mechanism toward better electrochemical performance of Li_4_Ti_5_O_12_ in lithium-ion battery. Adv. Sci..

[59] Li Y., Zhao H., Tian Z., Qiu W., Li X. (2008). Solvothermal synthesis and electrochemical characterization of amorphous lithium titanate materials. J. Alloys Compd..

[60] Shen L., Yuan C., Luo H., Zhang X., Chen L., Li H. (2011). Novel template-free solvothermal synthesis of mesoporous Li_4_Ti_5_O_12_-C microspheres for high power lithium ion batteries. J. Mater. Chem..

[61] Bach S., Pereira-Ramos J.P., Baffier N. (1999). Electrochemical properties of sol–gel Li_4/3_Ti_5/3_O_4_. J. Power Sources.

[62] Shen C.M., Zhang X.G., Zhou Y.K., Li H.L. (2002). Preparation and characterization of nanocrystalline Li_4_Ti_5_O_12_ by sol–gel method. Mater. Chem. Phys..

[63] Hao Y.-J., Lai Q.-Y., Lu J.-Z., Wang H.-L., Chen Y.-D., Ji X.-Y. (2005). Synthesis and characterization of spinel Li_4_Ti_5_O_12_ anode material by oxalic acid-assisted sol gel method. J. Power Sources.

[64] Hao Y.J., Lai Q.Y., Xu Z., Liu X., Ji X. (2005). Synthesis of TEA sol-gel method and electrochemical properties of Li_4_Ti_5_O_12_ anode material for lithium-ion battery. Solid State Ion..

[65] Hao Y.J., Lai Q.Y., Lu J.Z., Liu D.Q., Ji X.Y. (2007). Influence of various complex agents on electrochemical property of Li_4_Ti_5_O_12_ anode material. J. Alloys Compd..

[66] Miao X., He H., Shi L., Zhao X., Fang J. (2014). Sol-gel synthesis of nanocomposite/carbon nanotubes as anode materials for high-rate performance lithium-ion batteries. Adv. Mater. Res..

[67] Mosa J., Aparicio M. (2020). Sol-gel synthesis of nanocrystalline mesoporous Li_4_Ti_5_O_12_ thin-films as anodes for Li-ion microbatteries. Nanomaterials.

[68] Priyono S., Sofyan N., Subhan A., Prihandoko B., Yuwono A.H. (2023). Preparation of Al-doped Li_4_Ti_5_O_12_ anode material via sol-gel process with acidic catalyst for lithium-ion batteries. AIP Conf. Proc..

[69] Mani J., Katzke H., Habouti S., Moonoosawmy K.R., Dietze M., Es-Souni M. (2012). A template-free synthesis and structural characterization of hierarchically nano-structured lithium-titanium-oxide films. J. Mater. Chem..

[70] Jiang C.H., Ichihara M., Honma I., Zhou H.S. (2007). Effect of particle dispersion on high rate performance of nano-sized Li_4_Ti_5_O_12_ anode. Electrochim. Acta.

[71] Liu G., Zhang R., Bao K., Xie H., Zheng S., Guo J., Liu G. (2016). Synthesis of nano- anode material for lithium ion batteries by a biphasic interfacial reaction route. Ceram. Int..

[72] Ju S.H., Kang Y.C. (2009). Effects of preparation conditions on the electrochemical and morphological characteristics of Li_4_Ti_5_O_12_ powders prepared by spray pyrolysis. J. Power Sources.

[73] Ju S.H., Kang Y.C. (2009). Characteristics of spherical-shaped Li_4_Ti_5_O_12_ anode powders prepared by spray pyrolysis. J. Phys. Chem. Solids.

[74] Ju S.H., Kang Y.C. (2010). Effects of types of drying control chemical additives on the morphologies and electrochemical properties of Li_4_Ti_5_O_12_ anode powders prepared by spray pyrolysis. J. Alloys Compd..

[75] Ernst F.O., Kammler H.K., Roessler A., Pratsinis S.E., Stark W.J., Ufheil J., Novak P. (2007). Electrochemically active flame-made nanosized spinels: LiMn_2_O_4_, Li_4_Ti_5_O_12_ and LiFe_5_O_8_. Mater. Chem. Phys..

[76] Meierhofer F., Li H., Gockeln M., Kun R., Grieb T., Rosenauer A., Fritsching U., Kiefer J., Birkenstock J., Madler L. (2017). Screening precursor−solvent combinations for Li_4_Ti_5_O_12_ energy storage material using flame spray pyrolysis. ACS Appl. Mater. Interfaces.

[77] Gockeln M., Pokhrel S., Meierhofer F., Glenneberg J., Schowalter M., Rosenauer A., Fritsching U., Busse M., Mädler L., Kun R. (2018). Fabrication and performance of Li_4_Ti_5_O_12_/C Li-ion battery electrodes using combined double flame spray pyrolysis and pressure–based lamination technique. J. Power Sources.

[78] Terechshenko A., Sanbayeva A., Babaa M.R., Nurpeissova A., Bakenov Z. (2019). Spray-pyrolysis preparation of Li_4_Ti_5_O_12_/Si composites for lithium-ion batteries. Eurasian Chem. Technol. J..

[79] Xie Z., Song Q., Xie H., Yin H., Ning Z. (2021). Chemically driven synthesis of Ti3+ self-doped Li_4_Ti_5_O_12_ spinel in molten salt. J. Am. Ceram. Soc..

[80] Bai Y., Wang F., Wu F., Wu C., Bao L. (2008). Influence of composite LiCl–KCl molten salt on microstructure and electrochemical performance of spinel Li_4_Ti_5_O_12_. Electrochim. Acta.

[81] Sharmila S., Senthilkumar B., Kalai Selvan R. (2011). Molten-salt synthesis and characterization of Li_4_Ti_5_O_12_. AIP Conference Proceedings, Proceedings of the 55th DAE Solid State Physics.

[82] Guo Q., Li S., Wang H., Gao Y., Li B. (2014). Molten salt synthesis of nano-sized Li_4_Ti_5_O_12_ doped with Fe_2_O_3_ for use as anode material in the lithium-ion battery. RSC Adv..

[83] Xue X., Yan H., Fu Y. (2019). Preparation of pure and metal-doped Li_4_Ti_5_O_12_ composites and their lithium-storage performances for lithium-ion batteries. Solid State Ion..

[84] Kim H.K., Jegal J.-P., Kim J.-Y., Yoon S.-B., Roh K.C., Kim K.-B. (2013). In situ fabrication of lithium titanium oxide by microwave-assisted alkalization for high-rate lithium-ion batteries. J. Mater. Chem. A.

[85] He N., Wang B., Huang J. (2010). Preparation and electrochemical performance of monodisperse Li_4_Ti_5_O_12_ hollow spheres. J. Solid State Electrochem..

[86] Yu L., Wu H.B., Lou X.W. (2013). Mesoporous Li_4_Ti_5_O_12_ hollow spheres with enhanced lithium storage capability. Adv. Mater..

[87] Kawade U.V., Jayswal M.S., Ambalkar A.A., Kadam S.R., Panmand R.P., Ambekar J.D., Kulkarni M.V., Kale B.B. (2018). Surface modified Li_4_Ti_5_O_12_ by paper templated approach for enhanced interfacial Li^+^ charge transfer in Li-ion batteries. RSC Adv..

[88] Hermawan A., Wibowo A., Asri L.A.T.W., Shu Yin S., Purwasasmita B.S. (2019). Improved ionic conductivity of porous Li_4_Ti_5_O_12_ synthesized by sol-gel method using eggshell membrane as soft template. Mater. Res. Express.

[89] Kanamura K., Chiba T., Dokko K. (2006). Preparation of Li_4_Ti_5_O_12_ spherical particles for rechargeable lithium batteries. J. Eur. Ceram. Soc..

[90] Kim D.H., Ahn Y.S., Kim J. (2005). Polyol-mediated synthesis of Li_4_Ti_5_O_12_ nanoparticles and its electrochemical properties. J. Electrochem. Commun..

[91] Kataoka K., Takahashi Y., Kijima N., Akimoto J., Ohshima K. (2008). Single crystal growth and structure refinement of Li_4_Ti_5_O_12_. J. Phys. Chem. Solids.

[92] Wang G., Xu J., Wen M., Cai R., Ran R., Shao Z. (2008). Influence of high-energy ball milling of precursor on the morphology and electrochemical performance of Li_4_Ti_5_O_12_–Ball-milling time. Solid State Ion..

[93] Han S.-W., Jeong J., Yoon D.-H. (2014). Effects of high-energy milling on the solid-state synthesis of pure nano-sized Li_4_Ti_5_O_12_ for high power lithium battery applications. Appl. Phys. A.

[94] Li Y., Xie H., Li J., Wang J. (2012). Mechanochemical synthesis and electrochemical performances of Li_4_Ti_5_O_12_ anode materials for lithium-ion batteries. Adv. Mater. Res..

[95] Lu H.W., Zeng W., Li Y.S., Fu Z.W. (2007). Fabrication and electrochemical properties of three-dimensional net architectures of anatase TiO_2_ and spinel Li_4_Ti_5_O_12_ nanofibers. J. Power Sources.

[96] Castano N., Cortes M.A., Garcia E., Martinez H.V. (2018). Synthesis and morphological characterization of Li-Ti/PVP fibers as precursors for Li_4_Ti_5_O_12_ towards its future use as anode materials in Li-ion batteries by means of electrospinning. IOP Conf. Ser. Mater. Sci. Eng..

[97] Prakash A.S., Manikandan P., Ramesha K., Sathiya M., Tarascon J.M., Shukla A.K. (2010). Solution–combustion synthesized nanocrystalline Li_4_Ti_5_O_12_ as high-rate performance Li-ion battery anode. Chem. Mater..

[98] Cai R., Yu X., Liu X., Shao Z. (2010). Li_4_Ti_5_O_12_/Sn composite anodes for lithium-ion batteries: Synthesis and electrochemical performance. J. Power Sources.

[99] Yuan T., Cai R., Wang K., Ran R., Liu S., Shao Z. (2009). Combustion synthesis of high-performance Li_4_Ti_5_O_12_ for secondary Li-ion battery. Ceram. Int..

[100] De Sloovere D., Marchal W., Ulu F., Vranken T., Verheijen M., Van Bael M.K., Hardy A. (2017). Combustion synthesis as a low temperature route to Li_4_Ti_5_O_12_ based powders for lithium ion battery anodes. RSC Adv..

[101] Yuan T., Wang K., Cai R., Ran R., Shao Z. (2009). Cellulose-assisted combustion synthesis of Li_4_Ti_5_O_12_ adopting anatase TiO_2_ solid as raw material with high electrochemical performance. J. Alloys Compd..

[102] Yuan T., Cai R., Gu P., Shao Z. (2010). Synthesis of lithium insertion material Li_4_Ti_5_O_12_ from rutile TiO_2_ via surface activation. J. Power Sources.

[103] Raja M.W., Mahanty S., Kundu M., Basu R.N. (2009). Synthesis of nanocrystalline Li_4_Ti_5_O_12_ by a novel aqueous combustion technique. J. Alloys Compd..

[104] Lee S.S., Byun K.-T., Park J.P., Kim S.K., Kwak H.-Y., Shim I.-W. (2007). Preparation of nanoparticles by a simple sonochemical method. Dalton Trans..

[105] Ghosh S., Mitra S., Barpanda P. (2016). Sonochemical synthesis of nanostructured spinel Li_4_Ti_5_O_12_ negative insertion material for Li-ion and Na-ion batteries. Electrochim. Acta.

[106] Ni H., Song W.-L., Fan L.-Z. (2014). A strategy for scalable synthesis of Li_4_Ti_5_O_12_/reduced graphene oxide toward high rate lithium-ion batteries. Electrochem. Commun..

[107] Jin Y.-H., Min K.-M., Shim H.-W., Seo S.-D., Hwang I.-S., Park K.-S., Kim D.W. (2012). Facile synthesis of nano- for high-rate Li-ion battery anodes. Nanoscale Res. Lett..

[108] Mao S., Huang X., Chang J., Cui S., Zhou G., Chen J. (2015). One-step continuous synthesis of spherical Li_4_Ti_5_O_12_/graphene composite as an ultra-long cycle life lithium-ion battery anode. NPG Asia Mater..

[109] Tang B., Li A., Tong Y., Song H., Chen X., Zhou J., Ma Z. (2017). Carbon-coated Li_4_Ti_5_O_12_ tablets derived from metal-organic frameworks as anode material for lithium-ion batteries. J. Alloys Compd..

[110] Tang Y., Yang L., Qiu Z., Huang J. (2009). Template-free synthesis of mesoporous spinel lithium titanate microspheres and their application in high-rate lithium ion batteries. J. Mater. Chem..

[111] Wen Z., Gu Z., Huang S., Yang J., Lin Z., Yamamoto O. (2005). Research on spray-dried lithium titanate as electrode materials for lithium ion batteries. J. Power Sources.

[112] Wu X., Xinghua Liang X., Zhang X., Lan X., Li L., Suo Gai Q. (2021). Structural evolution of plasma sprayed amorphous Li_4_Ti_5_O_12_ electrode and ceramic/polymer composite electrolyte during electrochemical cycle of quasi-solid-state lithium battery. J. Adv. Ceram..

[113] Yin S.Y., Song L., Wang X.Y., Zhang M.F., Zhang K.L., Zhang Y.X. (2009). Synthesis of spinel Li_4_Ti_5_O_12_ anode material by a modified rheological phase reaction. Electrochim. Acta.

[114] Nugroho A., Yoon D., Joo O.-S., Chung K.Y., Kim J. (2014). Continuous synthesis of Li_4_Ti_5_O_12_ nanoparticles in supercritical fluids and their electrochemical performance for anode in Li-ion batteries. Chem. Eng. J..

[115] Doi T., Iriyama Y., Abe T., Ogumi Z. (2005). Electrochemical insertion and extraction of lithium ion at uniform nanosized Li_4/3_Ti_5/3_O_4_ particles prepared by a spray pyrolysis method. Chem. Mater..

[116] Naoi K., Ishimoto S., Isobe Y., Aoyagi S. (2010). High-rate nano-crystalline Li_4_Ti_5_O_12_ attached on carbon nano-fibers for hybrid supercapacitors. J. Power Sources.

[117] Bresser D., Paillard E., Copley M., Bishop P., Winter M., Passerini S. (2012). The importance of “going nano” for high power battery materials. J. Power Sources.

[118] Naoi K. (2010). Nanohybrid capacitor: The next generation electrochemical capacitors. Fuel Cells.

[119] Liu Y., Liu J.Y., Hou M.Y., Fan L., Wang Y., Xia Y. (2017). Carbon-coated Li_4_Ti_5_O_12_ nanoparticles with high electrochemical performance as anode material in sodium-ion batteries. J. Mater. Chem. A.

[120] Jiang C.H., Hosono E., Ichihara M., Honma I., Zhou H.S. (2008). Synthesis of nanocrystalline Li_4_Ti_5_O_12_ by chemical lithiation of anatase nanocrystals and postannealing. J. Electrochem. Soc..

[121] Lu J., Nan C., Peng Q., Li Y. (2012). Single crystalline lithium titanate nanostructure with enhanced rate performance for lithium ion battery. J. Power Sources.

[122] Song K., Seo D.-H., Jo M.R., Kim Y.-I., Kang K., Kang Y.-M. (2014). Tailored oxygen framework of Li_4_Ti_5_O_12_ nanorods for high-power Li ion battery. J. Phys. Chem. Lett..

[123] Li Y., Pan G.L., Liu J.W., Gao X.P. (2009). Preparation of Li_4_Ti_5_O_12_ nanorods as anode materials for lithium-ion batteries. J. Electrochem. Soc..

[124] Zhou Q., Liu L., Tan J.L., Tan Z., Huang Z., Wang X. (2015). Synthesis of lithium titanate nanorods as anode materials for lithium and sodium ion batteries with superior electrochemical performance. J. Power Sources.

[125] Priyono B., Herwono M.F., Syahrial A.Z., Nugraha M.R., Faizah, Subhan A. (2020). Enhancing lithium titanite (Li_4_Ti_5_O_12_) nanorods performance with graphite and nano tin as anode for lithium-ion batteries. AIP Conference Proceedings.

[126] Luo H., Shen L., Rui K., Li H., Zhang X. (2013). Carbon coated Li_4_Ti_5_O_12_ nanorods as superior anode material for high rate lithium ion batteries. J. Alloys Compd..

[127] Wang W., Guo Y., Liu L., Wang S., Yang X., Guo H. (2014). Gold coating for a high performance nanorod aggregates anode in lithium-ion batteries. J. Power Sources.

[128] Li Y., Song J., Tian Q. (2023). Li_4_Ti_5_O_12_ nanowires intertwined with carbon nanotubes for ultra-long life and conductive additive-free anodes of lithium-ion batteries. Mater. Lett..

[129] Hu G., Wu J., Du K., Peng Z., Jia M., Yang H., Cao Y. (2019). Surface-fluorinated Li_4_Ti_5_O_12_ nanowires/reduced graphene oxide composite as a high-rate anode material for lithium ion batteries. Appl. Surf. Sci..

[130] Li J.R., Tang Z.L., Zhang Z.T. (2005). Controllable formation and electrochemical properties of one-dimensional nanostructured spinel Li_4_Ti_5_O_12_. Electrochem. Commun..

[131] Bachtiar A.R., Syahrial A.Z., Nugraha M.R., Faizah, Subhan A., Priyono B. (2020). Enhancing performance of Li_4_Ti_5_O_12_ nanowire with addition of graphite and ZnO nanoparticle as anode for lithium-ion batteries. AIP Conference Proceedings.

[132] Kim T.-T., Yu C.-Y., Yoon C.S., Kim S.-J., Sun Y.-K., Myung S.-T. (2015). Carbon-coated Li_4_Ti_5_O_12_ nanowires showing high rate capability as an anode material for rechargeable sodium batteries. Nano Energy.

[133] Liu H., Tank K., Song K., van Aken P.A., Yu Y., Maier J. (2013). Tiny Li_4_Ti_5_O_12_ nanoparticles embedded in carbon nanofibers as high-capacity and long-life anode materials for both Li-ion and Na-ion batteries. Phys. Chem. Chem. Phys..

[134] Jia X., Lu Y., Wei F. (2016). Confined growth of Li_4_Ti_5_O_12_ nanoparticles in nitrogen-doped mesoporous graphene fibers for high-performance lithium-ion battery anodes. Nano Res..

[135] Xu H., Hu X., Sun Y., Luo W., Chen C., Liu Y., Huang Y. (2014). Highly porous Li_4_Ti_5_O_12_/C nanofibers for ultrafast electrochemical energy storage. Nano Energy.

[136] Bian M., Yang Y., Tian L. (2018). Carbon-free Li_4_Ti_5_O_12_ porous nanofibers as high-rate and ultralong-life anode materials for lithium-ion batteries. J. Phys. Chem. Solids.

[137] Jo M.R., Jung Y.S., Kang Y.-M. (2012). Tailored Li_4_Ti_5_O_12_ nanofibers with outstanding kinetics for lithium rechargeable batteries. Nanoscale.

[138] Zhao Y., Li J., Li Z., Yang K., Gao F. (2017). Pr-modified Li_4_Ti_5_O_12_ nanofibers as an anode material for lithium-ion batteries with outstanding cycling performance and rate performance. Ionics.

[139] Ji X., Lu Q., Guo E., Li D., Yao L., Liu H., Li X. (2018). Bamboo-shaped Zn^2+^-doped Li_4_Ti_5_O_12_ nanofibers: One-step controllable synthesis and high-performance lithium-ion batteries. J. Electrochem. Soc..

[140] Xu H., Hu X., Luo W., Sun Y., Yang Z., Hu C., Huang Y. (2014). Electrospun conformal Li_4_Ti_5_O_12_/C fibers for high-rate lithium-ion batteries. ChemElectroChem.

[141] Yagi S., Morinaga T., Togo M., Tsuda H., Shio S., Nakahira A. (2016). Ion-exchange synthesis of Li_4_Ti_5_O_12_ nanotubes and nanoparticles for high-rate Li-ion batteries. Mater. Trans..

[142] Jiang Y.-M., Wang K.-X., Wu X.-Y., Zhang H.-J., Bartlett B.M., Chen J.-S. (2014). Li_4_Ti_5_O_12_/TiO_2_ hollow spheres composed nanoflakes with preferentially exposed Li_4_Ti_5_O_12_ (011) facets for high-rate lithium ion batteries. ACS Appl. Mater. Interfaces.

[143] Zhang X., Xu W., Li X., Zhong X., Liu W., Lin Y., Xia R. (2018). Li_4_Ti_5_O_12_/Ti4O7/carbon nanotubes composite anode material for lithium-ion batteries. Micro Nano Lett..

[144] Wang H., Li S., Yang Y., Yu W., Ma Q., Dong X., Wang J., Liu G. (2019). Electrochemical characteristics of Li_4_Ti_5_O_12_/Ag composite nanobelts prepared via electrospinning. Russ. J. Phys. Chem. A.

[145] Qin W., Chen Y., An J., Zhang J., Wen X. (2022). High-loaded nanobelt-array/nanobelt-microsphere multilayer Li_4_Ti_5_O_12_ self-supported on Ti foils for high-performance lithium ion batteries. Electrochim. Acta.

[146] Tang Y.F., Yang L., Qiu Z., Huang J.S. (2008). Preparation and electrochemical lithium storage of flower-like spinel Li_4_Ti_5_O_12_ consisting of nanosheets. Electrochem. Commun..

[147] Hong Z.S., Lan T.B., Xiao F.Y., Zhang H.X., Wei M.D. (2011). Ultrafine Li_4_Ti_5_O_12_ nanosheets as a high performance anode for Li-ion battery. Funct. Mater. Lett..

[148] Wu L., Leng X., Liu Y., Wei S., Li C., Wang G., Lian J., Jiang Q., Nie A., Zhang T.-Y. (2017). A strategy for synthesis of nanosheets consisting of alternating spinel Li_4_Ti_5_O_12_ and rutile TiO_2_ lamellas for high-rate anodes of lithium-ion batteries. ACS Appl. Mater. Interfaces.

[149] Xu G.B., Tian Y., Wei X.L. (2017). Free-standing electrodes composed of carbon-coated Li_4_Ti_5_O_12_ nanosheets and reduced graphene oxide for advanced sodium ion batteries. J. Power Sources.

[150] Lai C., Dou Y.Y., Li X., Gao X.P. (2010). Improvement of the high rate capability of hierarchical structured Li_4_Ti_5_O_12_ induced by the pseudocapacitive effect. J. Power Sources.

[151] Sha Y., Zhao B., Ran R., Cai R., Shao Z. (2013). Synthesis of well-crystalized Li_4_Ti_5_O_12_ nanoplates for lithium-ion batteries with outstanding rate capability and cycling stability. J. Mater. Chem. A.

[152] Liu J., Wei X., Liu X.-W. (2015). Two-dimensional wavelike spinel lithium titanate for fast lithium storage. Sci. Rep..

[153] Salvatore K.L., Vila M.N., Renderos G., Li W., Housel L.M., Tong X., McGuire S.C., Gan J., Paltis A., Lee K. (2022). Probing the physicochemical behavior of variously doped Li_4_Ti_5_O_12_ nanoflowers. ACS Phys. Chem. Au.

[154] Lv Y., Zhang H., Cao G.P., Wang B.Y., Wang X.D. (2011). Phenol-formaldehyde resin-assisted synthesis of pure porous Li_4_Ti_5_O_12_ for rate capability improvement. Mater. Res. Bull..

[155] Kang E., Jung Y.S., Kim G.-H., Chun J., Wiesner U., Dillon A.C., Kim J.K., Lee J. (2011). Highly improved rate capability for a lithium-ion battery nano-Li_4_Ti_5_O_12_ negative electrode via carbon-coated mesoporous uniform pores with a simple self-assembly method. Adv. Funct. Mater..

[156] Saxena S., Sil A. (2016). Nanoporous Li_4_Ti_5_O_12_ material for the electrode of lithium ion battery. IETE Techn. Rev..

[157] Shao D., He J., Luo Y., Liu W., Yu X., Fang Y. (2012). Synthesis and electrochemical performance of nanoporous Li_4_Ti_5_O_12_ anode material for the lithium-ion batteries. J. Solid State Electrochem..

[158] Wang H., Wang L., Lin J., Yang J., Feng Wu F., Li L., Chen R. (2021). Structural and electrochemical characteristics of hierarchical Li_4_Ti_5_O_12_ as high-rate anode material for lithium-ion batteries. Electrochim. Acta.

[159] Liu J., Song K., van Aken P.A., Maier J., Yu Y. (2014). Self-supported Li_4_Ti_5_O_12_-C nanotube arrays as high-rate and long-life anode materials for flexible Li-ion batteries. Nano Lett..

[160] Qin W., Chen Y., An J., Wen X. (2022). Self-supported Li_4_Ti_5_O_12_ nanobelt array anode with long-life and improved low-temperature performance for flexible lithium-ion batteries. Ceram. Int..

[161] Liu J., Wei A., Pan G., Shen S., Xiao Z., Zhao Y., Xia X. (2021). Self-supported hierarchical porous Li_4_Ti_5_O_12_/carbon arrays for boosted lithium ion storage. J. Energy Chem..

[162] Pawlitzek F., Althues H., Schumm B., Kaskel S. (2017). Nanostructued networks for energy stotage: Vertically aligned carbon nanotubes (VACNT) as current collectors for high-power Li_4_Ti_5_O_12_(LTO)//LiMn_2_O_4_(LMO) lithium ion batteries. Batteries.

[163] Shen L., Uchaker E., Zhang X., Cao G. (2012). Hydrogenated Li_4_Ti_5_O_12_ nanowire arrays for high rate lithium ion batteries. Adv. Mater..

[164] Xia Q., Jabeen N., Savilov S.V., Aldoshind S.M., Xia H. (2016). Black mesoporous Li_4_Ti_5_O_12–δ_ nanowall arrays with improved rate performance as advanced 3D anodes for microbatteries. J. Mater. Chem. A.

[165] Jiang C., Ding W., Wu H., Yu Z., Ma L., Zou Z. (2018). Hierarchical Li_4_Ti_5_O_12_ nanosheet arrays anchoring on carbon fiber cloth as ultra-stable free-standing anode of Li-ion battery. Ceram. Int..

[166] Kim S.D., Rana K., Ahn J.-H. (2016). Additive-free synthesis of Li_4_Ti_5_O_12_ nanowire arrays on freestanding ultrathin graphite as a hybrid anode for flexible lithium ion batteries. J. Mater. Chem. A.

[167] Sorensen E.M., Barry S.J., Jung H.K., Rondinelli J.R., Vaughey J.T., Poeppelmeier K.R. (2006). Three-dimensionally ordered macroporous Li_4_Ti_5_O_12_: Effect of wall structure on electrochemical properties. Chem. Mater..

[168] Li N., Zhou G., Li F., Wen L., Cheng H.-M. (2013). A self-standing and flexible electrode of Li_4_Ti_5_O_12_ nanosheets with a N-doped carbon coating for high rate lithium ion batteries. Adv. Funct. Mater..

[169] Chen S., Xin Y., Zhou Y., Ma Y., Zhou H., Qi L. (2014). Self-supported Li_4_Ti_5_O_12_ nanosheet arrays for lithium ion batteries with excellent rate capability and ultralong cycle life. Energy Environ. Sci..

[170] Julien C.M., Massot M., Zaghib K. (2004). Structural studies of Li_4/3_Me_5/3_O_4_ (Me = Ti, Mn) electrode materials: Local structure and electrochemical aspects. J. Power Sources.

[171] Leonidov I.A., Leonidova O.N., Perelyaeva A.L., Samigullina R.F., Kovyazina S.A., Patrakeev M.V. (2003). Structure, ionic conduction, and phase transformations in lithium titanate Li_4_Ti_5_O_12_. Phys. Solid State.

[172] Julien C.M., Massot M. (2003). Lattice vibrations of materials for lithium rechargeable batteries. I. Lithium manganese oxide spinel. Mater. Sci. Eng. B.

[173] Pelegov D.V., Slautin B.N., Gorshkov V.S., Zelenovskiy P.S., Kiselev E.A., Kholkin A.L., Shur V.Y. (2017). Raman spectroscopy “big data” and local heterogeneity of solid state synthesized lithium titanate. J. Power Sources.

[174] Mosa J., Aparicio M., Tadanaga K., Hayashi A., Tatsumisago M. (2014). Li_4_Ti_5_O_12_ thin-film electrodes by in-situ synthesis of lithium alkoxide for Li-ion microbatteries. Electrochim. Acta.

[175] Tran M.V., Huynh N.L.T., Nguyen T.T., Ha D.T.C., Le P.M.L. (2016). Facile solution route to synthesize nanostructure Li_4_Ti_5_O_12_ for high rate Li-ion battery. J. Nanomater..

[176] Zhang Q., Lu H., Zhong H., Yan X., Ouyang C., Zhang L. (2015). W^6+^ and Br^−^ codoped Li_4_Ti_5_O_12_ anode with super rate performance for Li-ion batteries. J. Mater. Chem. A.

[177] Pelegov D.V., Nasara R.N., Tu C., Li S. (2019). Defects in Li_4_Ti_5_O_12_ induced by carbon deposition: An analysis of unidentified bands in Raman spectra. Phys. Chem. Chem. Phys..

[178] Kang C.-Y., Krajewski M., Lin J.-Y. (2021). Impact of titanium precursors on formation and electrochemical properties of Li_4_Ti_5_O_12_ anode materials for lithium-ion batteries. J. Solid State Electrochem..

[179] Li X., Hu H., Huang S., Yu G., Gao L., Liu H., Yu Y. (2013). Nano-sized Li_4_Ti_5_O_12_ anode material with excellent performance prepared by solid state reaction: The effect of precursor size and morphology. Electrochim. Acta.

[180] Lin C., Fan X., Xin Y., Cheng F., Lai M.O., Zhou H., Lu L. (2014). Monodispersed mesoporous Li_4_Ti_5_O_12_ submicrospheres as anode materials for lithium-ion batteries: Morphology and electrochemical performances. Nanoscale.

[181] Lin Y.-S., Duh J.-G., Tsai M.-C., Lee C.-Y. (2012). Self-assembled synthesis of monodispersed mesoporous Li_4_Ti_5_O_12_ beads and their applications in secondary lithium-ion batteries. Electrochim. Acta.

[182] Pawlitzek F., Pampel J., Schmuck M., Althues H., Schumm B., Kaskel S. (2016). High-power lithium ion batteries based on preorganized necklace type Li_4_Ti_5_O_12_/VACNT nano-composites. J. Power Sources.

[183] Tang Y., Tan X., Hou G., Zheng G. (2014). Nanocrystalline Li_4_Ti_5_O_12_-coated TiO_2_ nanotube arrays as three-dimensional anode for lithium-ion batteries. Electrochim. Acta.

[184] Wang G., Wang H., Ma G., Du X., Du L., Jing P., Wang Y., Wu K., Wu H., Wang Q. (2022). Investigation on process mechanism of a novel energy-saving synthesis for Li_4_Ti_5_O_12_ anode material. J. Energy Chem..

[185] Zhang B., Yu Y., Liu Y., Huang Z.-D., He Y., Kim J.-K. (2013). Percolation threshold of graphene nanosheets as conductive additives in Li_4_Ti_5_O_12_ anodes of Li-ion batteries. Nanoscale.

[186] Li Z., Ding F., Zhao Y., Wang Y., Li J., Yang K. (2016). synthesis and electrochemical performance of Li_4_Ti_5_O_12_ submirospheres coated with TiN as anode materials for lithium-ion batteries. Ceram. Int..

[187] Lin C.-Y., Duh J.-G. (2011). Porous Li_4_Ti_5_O_12_ anode material synthesized by one-step solid-state method for electrochemical properties enhancement. J. Alloys Compd..

[188] Haetge J., Hartman P., Brezenski K., Janek J., Brezenski T. (2011). Ordered large-pore mesoporous Li_4_Ti_5_O_12_ spinel thin film electrodes with nanocrystalline framework for high rate rechargeable lithium batteries: Relationships among charge storage, electrical conductivity, and nanoscale structure. Chem. Mater..

[189] Nowack L.V., Waser O., Yarema O., Wood V. (2013). Rapid, microwave-assisted synthesis of battery-grade lithium titanate (LTO). RSC Adv..

[190] Nguyen Huynh L.T., Duy Ha C.T., Nguyen V.D., Nguyen D.Q., Phung Le M.L., Tran V.M. (2019). Structure and electrochemical properties of Li_4_Ti_5_O_12_ prepared via low-temperature precipitation. J. Chem..

[191] Mahmoud A., Saadoune I., Lippens P., Chamas M. (2017). The design and study of new Li-ion full cells of LiCo_2/3_Ni_1/6_Mn_1/6_O_2_ positive electrode paired with MnSn_2_ and Li_4_Ti_5_O_12_ negative electrodes. Solid State Ion..

[192] Wang L., Zhang Y.M., Scofield M.E., Yue S.Y., McBean C., Marschilok A.C., Takeuchi K.J., Takeuchi E.S., Wong S.S. (2015). Enhanced performance of “flower-like” Li_4_Ti_5_O_12_ motifs as anode materials for high-rate lithium-ion batteries. ChemSusChem.

[193] Wang J., Yang Z., Li W., Zhong X., Gu L., Yu Y. (2014). Nitridation Br-doped Li_4_Ti_5_O_12_ anode for high rate lithium ion batteries. J. Power Sources.

[194] Hong H.-J., Ban G., Lee S.-M., Park I.-S., Lee Y.-J. (2020). Synthesis of 3D-structured Li_4_Ti_5_O_12_ from titanium(IV) oxysulfate (TiOSO_4_) solution as a highly sustainable anode material for lithium-ion batteries. J. Alloys Compd..

[195] Spitler T., Prochazka J., Kavan L., Graetzel M., Sugnaux F. (2009). High Performance Lithium Titanium Spinel Li4Ti5O12 for Electrode Material. U.S. Patent.

[196] Han S.Y., Kim I.Y., Jo K.Y., Hwang S.J. (2012). Solvothermal-assisted hybridization between reduced graphene oxide and lithium metal oxides: A facile route to graphene-based composite materials. J. Phys. Chem. C.

[197] Jung H.G., Myung S.T., Yoon C.S., Son S.B., Oh K.H., Amine K., Scrosati B., Sun Y.K. (2011). Microscale spherical carbon-coated Li_4_Ti_5_O_12_ as ultra high power anode material for lithium batteries. Energy Environ. Sci..

[198] Wu F., Wang Z., Li X., Wu L., Wang X., Zhang X., Wang Z., Xiong X., Guo H. (2011). Preparation and characterization of spinel Li_4_Ti_5_O_12_ anode material from industrial titanyl sulfate solution. J. Alloys Compd..

[199] Tsai M.C., Tsai T.L., Lin C.T., Chung R.J., Sheu H.S., Chiu H.T., Lee C.Y. (2008). Tailor made Mie scattering color filters made by size-tunable titanium dioxide particles. J. Phys. Chem. C.

[200] Zhang Q., Zhang S., Ning F., Lu X., Liu Y., Nie L., Ouyang C., Zhang L. (2017). Calcium doping of lithium titanium oxide nanospheres: A combined first-principles and experimental study. Energy Technol..

[201] Zhang F., Yi F., Gao A., Shu D., Sun Z., Mao J., Zhou X., Zhu Z., Sun Y. (2020). Interfacial electrostatic self-assembly in water-in-oil microemulsion assisted synthesis of Li_4_Ti_5_O_12_/graphene for lithium-ion-batteries. J. Alloys Compd..

[202] Liao J.-Y., Xiao X., Higgins D., Lee D., Hassan F., Chen Z. (2013). Hierarchical Li_4_Ti_5_O_12_-TiO_2_ composite microsphere consisting of nanocrystals for high power Li-ion batteries. Electrochim. Acta.

[203] Yu S.-H., Pucci A., Herntrich T., Willinger M.-G., Baek S.-H., Sungac Y.-E., Pinna N. (2011). Surfactant-free nonaqueous synthesis of lithium titanium oxide (LTO) nanostructures for lithium ion battery applications. J. Mater. Chem..

[204] Wu L., Kan S.R., Lu S.G., Zhang X.J., Jin W.H. (2007). Effect of particle size and agglomeration of TiO_2_ on synthesis and electrochemical properties of Li_4_Ti_5_O_12_. Trans. Nonferrous Met. Soc. China.

[205] Yuan T., Cai R., Shao Z. (2011). Different effect of the atmospheres on the phase formation and performance of Li_4_Ti_5_O_12_ prepared from ball-milling-assisted solid-phase reaction with pristine and carbon precoated TiO_2_ as starting materials. J. Phys. Chem. C.

[206] Xu R., Li J., Tang Z., Zhang Z. (2011). Li_4_Ti_5_O_12_ heat treated under nitrogen ambient with outstanding rate capabilities. J. Nanomater..

[207] Mahmoud A., Amarilla J.M., Lasri K., Saadoune I. (2013). Influence of the synthesis method on the electrochemical properties of the Li_4_Ti_5_O_12_ spinel in Li-half and Li-ion full-cells. A systematic comparison. Electrochim. Acta.

[208] Chen X., Guan X., Li L., Li G. (2012). Defective mesoporous Li_4_Ti_5_O_12−y_: An advanced anode with anomalous capacity and cycling stability at a high rate of 20C. J. Power Sources.

[209] Wang Y.Q., Gu L., Guo Y.G., Li H., He X.Q., Tsukimoto S., Ikuhara Y., Wan L.J. (2012). Rutile-TiO_2_ nanocoating for a high-rate Li_4_Ti_5_O_12_ anode of a lithium-ion battery. J. Am. Chem. Soc..

[210] Chiu H., Demopoulos G.P. (2013). A novel green approach to synthesis of nanostructured Li_4_Ti_5_O_12_ anode material. ECS Trans..

[211] Demopoulos G., Chiu H., Zaghib K., Guerfi A. (2014). Layered and Spinel Lithium Titanates and Processes for Preparing the Same. World Patent.

[212] Feng X., Zou H., Xiang H., Guo X., Zhou T., Wu Y., Xu W., Yan P., Wang C., Zhang J.-G. (2016). Ultrathin Li_4_Ti_5_O_12_ nanosheets as anode materials for lithium and sodium storage. ACS Appl. Mater. Interfaces.

[213] Suzuki S., Kozawa T., Murakami T., Naito M. (2017). Mechanochemical-hydrothermal synthesis of layered lithium titanate hydrate nanotubes at room temperature and their conversion to Li_4_Ti_5_O_12_. Mater. Res. Bull..

[214] Liu J., Li X., Geng D., Li Y., Wang D., Li R., Sun X., Cai M., Verbrugge M.W. (2012). Microwave-assisted hydrothermal synthesis of nanostructured spinel Li_4_Ti_5_O_12_ as anode materials for lithium ion batteries. Electrochim. Acta.

[215] Xu G.B., Li W., Yang L.W., Wei X.L., Ding J.W., Zhong J.X., Chu P.K. (2015). Highly-crystalline ultrathin Li_4_Ti_5_O_12_ nanosheets decorated with silver nanocrystals as a high-performance anode material for lithium ion batteries. J. Power Sources.

[216] Feng X.-Y., Li X., Tang M., Gan A., Hu Y.-Y. (2017). Enhanced rate performance of Li_4_Ti_5_O_12_ anodes with bridged grain boundaries. J. Power Sources.

[217] Yuan T., Cai R., Ran R., Zhou Y., Shao Z. (2010). A mechanism study of synthesis of Li_4_Ti_5_O_12_ from TiO_2_ anatase. J. Alloys Compd..

[218] Lin C., Lai M.O., Li L., Zhou H., Xin Y. (2013). Structure and high performance of Ni^2+^ doped Li_4_Ti_5_O_12_ for lithium ion battery. J. Power Sources.

[219] Hu X., Li Z., Yang K., Huai Y., Deng Z. (2011). Effects of carbon source and carbon content on electrochemical performances of Li_4_Ti_5_O_12_/C prepared by one-step solid-state reaction. Electrochim. Acta.

[220] Shen Y., Søndergaard M., Christensen M., Birgisson S., Iversen B.B. (2014). Solid state formation mechanism of Li_4_Ti_5_O_12_ from an anatase TiO_2_ source. Chem. Mater..

[221] Harrison M.R., Edwards P.P., Goodenough J.B. (1985). The superconductor-semiconductor transition in the Li_1+x_Ti_2–x_O_4_ spinel system. Philos. Mag. B.

[222] Jhan Y.R., Duh J.G. (2012). Synthesis of entanglement structure in nanosized Li_4_Ti_5_O_12_/multi-walled carbon nanotubes composite anode material for Li-ion batteries by ball-milling-assisted solid-state reaction. J. Power Sources.

[223] Wang Y., Zou W., Dai X., Feng L., Zhang H., Zhou A., Li J. (2014). Solid-state synthesis of graphite carbon-coated Li_4_Ti_5_O_12_ anode for lithium ion batteries. Ionics.

[224] Yi T.F., Yang S.Y., Li X.Y., Yao J.H., Zhu Y.R., Zhu R.S. (2014). Sub-micrometric Li_4−x_Na_x_Ti_5_O_12_ (0 ≤ x ≤ 0.2) spinel as anode material exhibiting high-rate capability. J. Power Sources.

[225] Yang L.X., Gao L.J. (2009). Li_4_Ti_5_O_12_/C composite electrode material synthesized involving conductive carbon precursor for Li-ion battery. J. Alloys Compd..

[226] Kim J., Cho J. (2007). Spinel Li_4_Ti_5_O_12_ nanowires for high-rate Li-ion intercalation electrode. Electrochem. Solid-State Lett..

[227] Han S.-W., Ryu J.H., Jeong J., Yoon D.-H. (2013). Solid-state synthesis of Li_4_Ti_5_O_12_ for high power lithium-ion battery applications. J. Alloys Compd..

[228] Yao W., Zhuang W., Ji X., Chen J., Lu X., Wang C. (2016). Solid-state synthesis of Li_4_Ti_5_O_12_ whiskers from TiO_2_-B. Mater. Res. Bull..

[229] Cheng L., Yan J., Zhu G.-N., Luo J.-Y., Wang C.-X., Xia Y.-Y. (2010). General synthesis of carbon-coated nanostructure Li_4_Ti_5_O_12_ as a high rate electrode material for Li-ion intercalation. J. Mater. Chem..

[230] Wang D., Wu X., Zhang Y., Wang J., Yan P., Zhang C., He D. (2014). The influence of the TiO_2_ particle size on the properties of Li_4_Ti_5_O_12_ anode material for lithium-ion battery. Ceram. Int..

[231] Ohtake T., Iijima K. (2015). Li_4_Ti_5_O_12_ synthesis with high specific surface area and single phase. J. Mater. Sci. Chem. Eng..

[232] Ma G., Cheng M. (2019). Preparation and properties of Li_4_Ti_5_O_12_/C composites. Integr. Ferroelectr..

[233] Purwanto A., Muzayanha S.U., Yudha C.S., Widiyandari H., Jumari A., Dyartanti E.R., Nizam M., Putra M.I. (2020). High performance of salt-modified–LTO anode in LiFePO_4_ battery. Appl. Sci..

[234] Bai X., Li T., Bai Y.-J. (2020). Capacity degradation of Li_4_Ti_5_O_12_ during long-term cycling in terms of composition and structure. Dalton Trans..

[235] Allen J.L., Jow T.R., Wolfenstine J. (2006). Low temperature performance of nanophase Li_4_Ti_5_O_12_. J. Power Sources.

[236] Yao X.L., Xie S., Nian H.Q., Chen C.H. (2008). Spinel Li_4_Ti_5_O_12_ as a reversible anode material down to 0 V. J. Alloys Compd..

[237] Wang Z., Wang Z., Peng W., Guo H., Li X. (2014). An improved solid-state reaction to synthesize Zr-doped Li_4_Ti_5_O_12_ anode material and its application in LiMn_2_O_4_/Li_4_Ti_5_O_12_ full-cell. Ceram. Int..

[238] Zaghib K., Simoneau M., Armand M., Gauthier M. (1999). Electrochemical study of Li_4_Ti_5_O_12_ as negative electrode for Li-ion polymer rechargeable batteries. J. Power Sources.

[239] Berbenni V., Milanese C., Bruni G., Marini A. (2010). Mechano-thermally activated solid-state synthesis of Li_4_Ti_5_O_12_ spinel from Li_2_CO_3_-TiO_2_ mixtures. Z. Naturforsch. B Chem. Sci..

[240] Liu W., Zhang J., Wang Q., Xie X., Lou Y., Xia B. (2014). The effects of Li_2_CO_3_ particle size on the properties of lithium titanate as anode material for lithium-ion batteries. Ionics.

[241] Wang Y., Ren Y., Dai X., Yan X., Huang B., Li J. (2018). Electrochemical performance of ZnO-coated Li_4_Ti_5_O_12_ composite electrodes for lithium-ion batteries with the voltage ranging from 3 to 0.01 V. R. Soc. Open Sci..

[242] Huang S., Wen Z.Y., Zhu X.J., Gu Z.H. (2004). Preparation and electrochemical performance of Ag doped Li_4_Ti_5_O_12_. Electrochem. Commun..

[243] Zhou T.P., Feng X.Y., Guo X., Wu W.W., Cheng S., Xiang H.F. (2015). Solid-state synthesis and electrochemical performance of Ce-doped Li_4_Ti_5_O_12_ anode materials for lithium-ion batteries. Electrochim. Acta.

[244] Becker D., Haberkorn R., Kickelbick G. (2018). Mechanochemical induced structure transformations in lithium titanates: A detailed PXRD and ^6^Li MAS NMR study. Inorganics.

[245] Huang Z., Wang D., Lin Y., Wu X., Yan P., Zhang C., He D. (2014). Enhancing the high-rate performance of Li_4_Ti_5_O_12_ anode material for lithium-ion battery by a wet ball milling assisted solid-state reaction and ultra-high speed nano-pulverization. J. Power Sources.

[246] Natalia V., Gustami A.P., Rahmawati F., Lestari W.W., Purwanto A. (2018). Lithium titanate (LTO) synthesis through solid state reaction and its performance for LiFePO_4_/LTO battery. J. Math. Fund. Sci..

[247] Xiao H., Huang X., Ren Y., Ding X., Zhou S. (2019). Fabrication of Li_4_Ti_5_O_12_@CN composite with enhanced rate properties. Front. Chem..

[248] Krajewski M., Michalska M., Hamankiewicz B., Ziolkowska D., Korona K.P., Jasinski J.B., Maria Kaminska M., Lipinska L., Czerwinski A. (2014). Li_4_Ti_5_O_12_ modified with Ag nanoparticles as an advanced anode material in lithium-ion batteries. J. Power Sources.

[249] Kim S., Alauzun J.G., Louvain N., Brun N., Stievano L., Boury B., Monconduit L., Mutin P.H. (2018). Alginic acid aquagel as a template and carbon source in the synthesis of Li_4_Ti_5_O_12_/C nanocomposites for application as anodes in Li-ion batteries. RSC Adv..

[250] Yi T.-F., Xie Y., Zhu Y.-R., Zhu R.-S., Shen H. (2013). Structural and thermodynamical stability of Li_4_Ti_5_O_12_ anode material for lithium-ion battery. J. Power Sources.

[251] Qiao Y., Hu X., Liu Y., Huang Y. (2012). Li_4_Ti_5_O_12_ nanocrystallites for high-rate lithium-ion batteries synthesized by a rapid microwave-assisted solid-state process. Electrochim. Acta.

[252] Shi L., Hu X., Huang Y. (2014). Fast microwave-assisted synthesis of Nb-doped Li_4_Ti_5_O_12_for high-rate lithium-ion batteries. J. Nanopart. Res..

[253] Ohtake T. (2015). Single phase Li_4_Ti_5_O_12_ synthesis for nanoparticles by two steps sintering. J. Mater. Sci. Chem. Eng..

[254] Bach S., Pereira-Ramos J.P., Baffier N. (1998). Electrochemical behaviour of a lithium titanium spinel compound synthesized via a sol-gel process. J. Mater. Chem..

[255] Venkateswarlu M., Chen C.H., Do J.S., Lin C.W., Chou T.C., Hwang B.J. (2005). Electrochemical properties of nano-sized Li_4_Ti_5_O_12_ powders synthesized by a sol–gel process and characterized by X-ray absorption spectroscopy. J. Power Sources.

[256] Rho Y.H., Kanamura K. (2004). Li^+^ ion diffusion in Li_4_Ti_5_O_12_ thin film electrode prepared by PVP sol-gel method. J. Solis State Chem..

[257] Rho Y.H., Kanamura K. (2004). Preparation of Li_4/3_Ti_5/3_O_4_ thin film electrodes by a PVP sol-gel coating method and their electrochemical properties. J. Electrochem. Soc..

[258] Gao J., Jiang C., Wan C. (2010). Synthesis and characterization of spherical La-doped nanocrystalline Li_4/3_Ti_5/3_O_4_/C compound for Lithium-ion batteries. J. Electrochem. Soc..

[259] Wang G.J., Gao J., Fu L.J., Zhao N.H., Wu Y.P., Takamura T. (2007). Preparation and characteristic of carbon-coated Li_4/3_Ti_5/3_O_4_ anode material. J. Power Sources.

[260] Liu D.Q., Lai Q.Y., Hao Y.J. (2004). Study on synthesis and mechanism of Li_4/3_Ti_5/3_O_4_ by sol-gel method. Chin. J. Inorg. Chem..

[261] Xiang H.F., Tian B.B., Lian P.C., Li Z., Wang H. (2011). Sol-gel synthesis and electrochemical performance of Li_4_Ti_5_O_12_/graphene composite anode for lithium-ion batteries. J. Alloys Compd..

[262] Fawwaz T.A., Retna D.P., Riesma T., Ade U.H., Sri R., Damish, Hanif Y., Oka P.A., Nendar H., Yelvia D. (2022). Synthesis of lithium titanium oxide (Li_4_Ti_5_O_12_) through sol-gel- method and the effect of graphene addition in lithium-ion battery anodes. Defect Diff. Forum.

[263] Zhang C., Zhang Y., Wang J., Wang D., He D., Xia Y. (2013). Li_4_Ti_5_O_12_ prepared by a modified citric acid sol-gel method for lithium-ion battery. J. Power Sources.

[264] Supriadi C.P., Syahrial A.Z., Subhan A. (2020). The effect of sol-gel derived TiO_2_ crystallite size to Li_4_Ti_5_O_12_ anode performance in lithium-ion battery. Ionics.

[265] Livage J. (1991). Vanadium pentoxide gels. Chem. Mater..

[266] Julien C., El-Farh L., Rangan S., Massot M. (1999). Studies of LiNi_1–y_Co_y_O_2_ cathode materials prepared by a citric acid-assisted sol-gel method for lithium batteries. J. Sol-Gel Sci. Technol..

[267] Luo G., He J., Song X., Huang X., Yu X., Fang Y., Chen D. (2015). Bamboo carbon assisted sol-gel synthesis of Li_4_Ti_5_O_12_ anode material with enhanced electrochemical activity for lithium ion battery. J. Alloys Compd..

[268] Hao Y.-J., Lai Q.-Y., Liu D.-Q., Xu Z.-U., Ji X.-Y. (2005). Synthesis by citric acid sol-gel method and electrochemical properties of Li_4_Ti_5_O_12_ anode material for lithium-ion battery. Mater. Chem. Phys..

[269] Yang G., Su Z., Fang H., Yao Y., Li Y., Yang B., Ma W. (2013). Synthesis and performance of Li_4_Ti_5_O_12_/C with little inert carbon. Electrochim. Acta.

[270] Kuo Y.-C., Lin J.-Y. (2014). One-pot sol-gel synthesis of Li_4_Ti_5_O_12_/C anode materials for high-performance Li-ion batteries. Electrochim. Acta.

[271] Kurajica S. (2019). A brief review on the use of chelation agents in sol-gel Synthesis with emphasis on β-diketones and β-ketoesters. Chem. Biochem. Eng. Q..

[272] Kirillov S.A., Romanova I.V., Lisnycha T.V., Potapenko A.V. (2018). High-rate electrochemical performance of Li_4_Ti_5_O_12_ obtained from TiCl4 by means of a citric acid aided route. Electrochim. Acta.

[273] Wang J., Liu X.-M., Yang H., Shen X. (2011). Characterization and electrochemical properties of carbon-coated Li_4_Ti_5_O_12_ prepared by a citric acid sol–gel method. J. Alloys Compd..

[274] Purwamargapratala Y., Sujatno A., Sabayu Y.L., Kartini E. (2019). Synthesis of Li_4_Ti_5_O_12_ (LTO) by sol-gel method for lithium ion battery anode. IOP Conf. Ser. Mater. Sci. Eng..

[275] Stenina I.A., Il’in A.B., Yaroslavtsev A.B. (2015). Synthesis and ionic conductivity of Li_4_Ti_5_O_12_. Inorg. Mater..

[276] Mahmoud A., Amarilla J.M., Saadoune I. (2015). Effect of thermal treatment used in the sol-gel synthesis method of Li_4_Ti_5_O_12_ spinel on its electrochemical properties as anode for lithium ion batteries. Electrochim. Acta.

[277] Zhou W., Shao Z., Jin W. (2006). Synthesis of nanocrystalline conducting composite oxides based on a non-ion selective combined complexing process for functional applications. J. Alloys Compd..

[278] Rho Y.H., Kanamura K. (2003). Preparation of Li_4_Ti_5_O_12_ thin film electrode with PVP sol-gel for a rechargeable lithium microbattery. J. Surf. Sci. Soc. Jpn..

[279] Fang W., Cheng X., Zuo P., Ma Y., Liao L., Yin G. (2013). Hydrothermal-assisted sol-gel synthesis of Li_4_Ti_5_O_12_/C nano-composite for high-energy lithium-ion batteries. Solid State Ion..

[280] Chang L.-J., Luo D.-H., Zhang H.-L., Qi X.-W., Wang Z.-Y., Liu Y.-G., Zhai Y.-C. (2014). Synthesis and performance of Li_4_Ti_5_O_12_ anode materials using the PVP-assisted combustion method. Chin. Chem. Lett..

[281] Lee J.-M., Jun Y.-D., Kim D.-W., Lee Y.-H., Oh S.-G. (2009). Effects of PVP on the formation of silver–polystyrene heterogeneous nanocomposite particles in novel preparation route involving polyol process: Molecular weight and concentration of PVP. Mater. Chem. Phys..

[282] Ma G., Cheng M. (2020). Preparation and performance of Li_4_Ti_5_O_12_ by sol-gel method. Integr. Ferroelectr..

[283] Xia Y., Sun B., Wei Y., Tao B., Zhao Y. (2017). Simple sol-gel method synthesis of 3-dimension Li_4_Ti_5_O_12_-TiO_2_ nanostructures using butterfly wings as biotemplates for high-rate performance lithium-ion batteries. J. Alloys Compd..

[284] Qiu C., Yuan Z., Liu L., Ye N., Liu J. (2013). Sol-gel preparation and electrochemical properties of La-doped anode material for lithium-ion batteries. J. Solid State Electrochem..

[285] Kavan L., Grätzel M. (2002). Facile synthesis of nanocrystalline Li_4_Ti_5_O_12_ (spinel) exhibiting fast Li insertion. Electrochem. Solid State Lett..

[286] Pershina S.V., Antonov B.D., Farlenkov A.S. (2021). Optimization of technology for synthesis of Li_4_Ti_5_O_12_ anode materials for lithium-ion batteries. Russ. J. Appl. Chem..

[287] Wang J., Liu X., Yang H. (2012). Synthesis and electrochemical properties of highly dispersed Li_4_Ti_5_O_12_ nanocrystalline for lithium secondary batteries. Trans. Nonferrous Met. Soc. China.

[288] Zhang N., Liu Z., Yang T., Liao C., Wang Z., Sun K. (2011). Facile preparation of nanocrystalline Li_4_Ti_5_O_12_ and its high electrochemical performance as anode material for lithium-ion batteries. Electrochem. Commun..

[289] Morsy S.M.I. (2014). Role of surfactants in nanotechnology and their applications. Int. J. Curr. Microbiol. Appl. Sci..

[290] Dokan F.K., Sahan H., Ozdemir B., Ozdemir N., Patat S. (2014). Synthesis and characterization of spinel Li_4_Ti_5_O_12_ anode material by CTAB assisted sol-gel method. Acta Phys. Pol. A.

[291] Khomane R.B., Prakash A.S., Ramesha K., Sathiya M. (2011). CTAB-assisted sol-gel synthesis of Li_4_Ti_5_O_12_ and its performance as anode material for Li- ion batteries. Mater. Res. Bull..

[292] Chen J., Yang S., Fang S., Hirani S., Tachibana K. (2012). Synthesis of hierarchical mesoporous nest-like Li_4_Ti_5_O_12_ for high-rate lithium ion batteries. J. Power Sources.

[293] Li D., Zhao W., Cao L., Gao Y., Liu Y., Wang W., Qi T. (2019). Mixed-surfactant-assisted synthesis of dual-phase Li_4_Ti_5_O_12_-TiO_2_ hierarchical microspheres as high-performance anode materials for Li-ion batteries. ChemSusChem.

[294] Akintola T., Shellikeri A., Akintola T., Zheng J.P. (2021). The influence of Li_4_Ti_5_O_12_ preparation method on lithium-ion capacitor performance. Batteries.

[295] Meyer F., Hempelmann R., Mathur S., Veith M. (1999). Microemulsion mediated sol-gel synthesis of nano-scaled MAl2O4 (M=Co, Ni, Cu) spinels from single-source heterobimetallic alkoxide precursors. J. Mater. Chem..

[296] Hasegawa G., Kanamori K., Kiyomura T., Kurata H., Nakanishi K., Abe T. (2015). Hierarchically porous Li_4_Ti_5_O_12_ anode materials for Li- and Na-ion batteries: Effects of nanoarchitectural design and temperature dependence of the rate capability. Adv. Energy Mater..

[297] Wang D., Liu H., Li M., Wang X., Bai S., Shi Y., Tian J., Shan Z., Meng M., Liu P. (2019). Nanosheet-assembled hierarchical Li_4_Ti_5_O_12_ microspheres for high-volumetric-density and high-rate Li-ion battery anode. Energy Storage Mater..

[298] Erdas A., Ozcan S., Guler M.O., Akbulut H. (2015). Sol-gel synthesis of nanocomposite Cu–Li_4_Ti_5_O_12_ structures for ultrahigh rate Li-ion batteries. Acta Phys. Pol. A.

[299] Liu Z., Huang Y., Wang X., Zhang Y., Ding J., Guo Y. (2021). Synthesis of Li_4_Ti_5_O_12_/V_2_O_5_ nanocomposites for lithium-ion batteries by one-pot co-precipitation method. J. Mater. Sci. Mater. Electron..

[300] Zhang Y., Zhang C., Lin Y., Xiong D.-B., Wang D., Wu X., He D. (2014). Influence of Sc^3+^ doping in B-site on electrochemical performance of Li_4_Ti_5_O_12_ anode materials for lithium-ion battery. J. Power Sources.

[301] Wei G., Rambo C.R., Guo Y., Ning Z., Guo S., Zhao M., Huang Z., Zhang C., He D. (2017). Graphene coated La^3+^/Sc^3+^ co-doped Li_4_Ti_5_O_12_ anodes for enhanced Li-ion battery performance. Mater. Lett..

[302] Alias N., Kufian M., Teo L., Majid S., Arof A. (2009). Synthesis and characterization of Li_4_Ti_5_O_12_. J. Alloys Compd..

[303] Llaín-Jiménez H.A., Buchberger D.A., Winkowska-Struzik M., Ratyński M., Krajewski M., Boczar M., Hamankiewicz B., Czerwiński A. (2022). Correlation between lithium titanium oxide powder morphology and high rate performance in lithium-ion batteries. Batteries.

[304] Rajoba S.J., Shirsat A.N., Tyagi D., Jadhav L.D., Kalubarme R.S., Wani B.N., Varma S. (2021). Sol-gel assisted spinel Li_4_Ti_5_O_12_ and its performance and stability as anode for long life Li-ion battery. Mater. Lett..

[305] Najihah A.I., Priyono S., Imam Supardi Z.A., Subhan A., Prihandoko B. (2020). Synthesized of Li_4_Ti_5_O_12_ materials via sol-gel method to lithium ion battery anodes. IOP Conf. Ser. J. Phys..

[306] Priyono S., Nuroniah I., Subhan A., Sanjaya E., Prihandoko B. (2019). Synthesis and characterization of Li_4_Ti_5_O_12_ with sol gel method as a lithium-ion battery anode material. J. Sains Mater. Indones..

[307] Chang C.-M., Chen Y.-C., Ma W.-L., Wang P.-H., Lee C.-F., Chen H.-S., Chen-Yang Y.W. (2015). Sol-gel synthesis of low carbon content and low surface area Li_4_Ti_5_O_12_/carbon black composite as high-rate anode materials for lithium ion battery. RSC Adv..

[308] Shen L., Yuan C., Luo H., Zhang X., Xu K., Zhang F. (2011). In situ growth of Li_4_Ti_5_O_12_ on multi-walled carbon nanotubes: Novel coaxial nanocables for high rate lithium ion batteries. J. Mater. Chem..

[309] Zhong Z. (2007). Synthesis of Mo^4+^ substituted spinel Li_4_Ti_5–x_Mo_x_O_12_. Electrochem. Solid-State Lett..

[310] Zhang A., Zheng Z.-M., Cheng F.-Y., Liang Z., Chen J. (2011). Preparation of Li_4_Ti_5_O_12_ submicrospheres and their application as anode materials of rechargeable lithium-ion batteries. Sci. China Chem..

[311] Wang X.-Y., Li Y.-J., Xu C., Kong L., Li L. (2014). Synthesis and characterization of Li_4_Ti_5_O_12_ via a hydrolysis process from TiCl_4_ aqueous solution. Rare Met..

[312] Li D., Shen G., Zhao W., Gao Y., Hui Z., Liu Y., Yi L., Wang W., Cao L., Liu Y. (2019). Synthesis of Li_4_Ti_5_O_12_ with theoretical capacity in Li_2_CO_3_-ammonia ball milling system. Mater. Res. Bull..

[313] Wang R., Cao X., Zhao D., Zhu L., Xie L., Liu J., Liu Y. (2020). Wet-chemistry synthesis of Li_4_Ti_5_O_12_ as anode materials rendering high-rate Li-ion storage. Int. J. Energy Res..

[314] Xu C., Xue L., Zhang W., Fan X., Yan Y., Li Q., Huang Y., Zhang W. (2014). Hydrothermal synthesis of Li_4_Ti_5_O_12_/TiO_2_ nanocomposite as high performance anode material for Li-ion batteries. Electrochim. Acta.

[315] Xie L.-L., Xu Y.-D., Zhang J.J., Cao X.-Y., Wang B., Yan X.-Y., Qu L.-B. (2013). Facile Synthesis and characterization of Li_4_Ti_5_O_12_ as anode material for lithium ion batteries. Int. J. Electrochem. Sci..

[316] Shin J.W., Hong C.H., Yoon D.H. (2012). Effects of TiO_2_ starting materials on the solid-state formation of Li_4_Ti_5_O_12_. J. Am. Ceram. Soc..

[317] Hong C.H., Noviyanto A., Ryu J.H., Kim J., Yoon D.H. (2012). Effects of the starting materials and mechanochemical activation on the properties of solid-state reacted Li_4_Ti_5_O_12_ for lithium ion batteries. Ceram. Int..

[318] Li H., Shen L., Zhang X., Wang J., Nie P., Che Q., Ding B. (2013). Nitrogen-doped carbon coated Li_4_Ti_5_O_12_ nanocomposite: Superior anode materials for rechargeable lithium ion batteries. J. Power Sources.

[319] Veljković I., Poleti D., Karanović L., Zdujić M., Branković G. (2011). Solid state synthesis of extra phase-pure Li_4_Ti_5_O_12_ spinel. Sci. Sinter..

[320] Guerfi A., Yuichi A., Charest P., Mamoru S., Zaghib K. (2006). Li_4_Ti_5_O_12_ material prepared by mechanochemical activation: Structure and electrochemical performance. ECS Meet. Abstr..

[321] Priyono B., Winowatan P.W., Syahrial A.Z., Faizah, Subhan A. (2018). Optimizing the performance of Li_4_Ti_5_O_12_/LTO by addition of silicon nanoparticles in half cell lithium_ion battery anode. IOP Conf. Ser. Earth Environ. Sci..

[322] Liu W., Zhang J., Wang Q., Xie X., Lou Y., Han X., Xia B. (2013). Microsized TiO_2_ activated by high-energy ball milling as starting material for the preparation of Li_4_Ti_5_O_12_ anode material. Powder Technol..

[323] Jia P., Shao Z., Liu K. (2014). Synthesis and electrochemical performance of Li_4_Ti_5_O_12_ by high temperature ball milling method. Mater. Lett..

[324] Jia P., Shao Z., Liu K. (2014). Pretreatments-assisted high temperature ball milling route to Li_4_Ti_5_O_12_ and its electrochemical performance. Mater. Lett..

[325] Michalska M., Krajewski M., Ziolkowska D., Hamankiewicz B., Andrzejczuk M., Lipinska L., Korona K.P., Czerwinski A. (2014). Influence of milling time in solid-state synthesis on structure, morphology and electrochemical properties of Li_4_Ti_5_O_12_ of spinel structure. Powder Technol..

[326] Yan G., Fang H., Zhao H., Li G., Yang Y., Li L. (2009). Ball milling-assisted sol–gel route to Li_4_Ti_5_O_12_ and its electrochemical properties. J. Alloys Compd..

[327] HKim H.J., Oh M.H., Son W.K., Kim T.I., Park S.G. Novel synthesis method and electrochemical characteristics of lithium titanium oxide as anode material for high power device. Proceedings of the 2006 IEEE 8th International Conference on Properties & Applications of Dielectric Materials.

[328] Wang D., Zhang C., Zhang Y., Wang J., He D. (2013). Synthesis and electrochemical properties of La-doped Li_4_Ti_5_O_12_ as anode material for Li-ion battery. Ceram. Int..

[329] Jang I.-S., Kang S.-H., Kang Y.C., Roh K.C., Chun J. (2022). Facile synthesis of surface fluorinated-Li_4_Ti_5_O_12_/carbon nanotube nanocomposites for a high-rate capability anode of lithium-ion batteries. Appl. Surf. Sci..

[330] Ye Z., Zhong F., Chen Y., Zou Z., Jiang C. (2022). Unique CNTs-chained Li_4_Ti_5_O_12_ nanoparticles as excellent high rate anode materials for Li-ion capacitors. Ceram. Int..

[331] Chauque S., Oliva F.Y., Visintin A., Barraco D., Leiva E.P.M., Cámara O.R. (2017). Lithium titanate as anode material for lithium ion batteries: Synthesis, post-treatment and its electrochemical response. J. Electroanal. Chem..

[332] Zhang C., Shao D., Yu J., Zhang L., Huang X., Xu D., Yu X. (2016). Synthesis and electrochemical performance of cubic Co-doped Li_4_Ti_5_O_12_ anode material for high-performance lithium-ion batteries. J. Electroanal. Chem..

[333] Cheng L., Liu H.J., Zhang J.J., Xiong H.M., Xia Y.Y. (2006). Nanosized Li_4_Ti_5_O_12_ prepared by molten salt method as an electrode material for hybrid electrochemical supercapacitors. J. Electrochem. Soc..

[334] Nithya V.D., Sharmila S., Vediappan K., Lee C.W., Vasylechko L., Kalai Selvan R. (2014). Electrical and electrochemical properties of molten-saltsynthesized 0.05 mol Zr- and Si-doped Li_4_Ti_5_O_12_ microcrystals. J. Appl. Electrochem..

[335] Nithya V.D., Kalai Selvan R., Kumaran V., Sharmila S., Lee C.W. (2012). Molten salt synthesis and characterization of Li_4_Ti_5−x_Mn_x_O_12_ (x = 0.0, 0.05 and 0.1) as anodes for Li-ion batteries. Appl. Surf. Sci..

[336] Guo Q., Wang Q., Chen G., Shen M., Li B. (2017). Molten salt synthesis of different ionic radii metallic compounds doped lithium titanate used in Li-ion battery anodes. Mater. Trans..

[337] Rahman M., Wang J.Z., Hassan M.F., Wexler D., Liu H.K. (2011). Amorphous carbon coated high grain boundary density dual phase Li_4_Ti_5_O_12_-TiO_2_: A nanocomposite anode material for Li-ion batteries. Adv. Energy. Mater..

[338] Sharmila S., Senthilkumar B., Nithya V.D., Vediappan K., Lee C.W., Kalai Selvan R. (2013). Electrical and electrochemical properties of molten salt-synthesized Li_4_Ti_5–x_O_12_Sn_x_O_12_ (x = 0.0, 0.05 and 0.1) as anodes for Li-ion batteries. J. Phys. Chem. Solids.

[339] Rahman M.M., Wang J.-Z., Hassan M.F., Chou S., Wexler D., Liu H.-K. (2010). Basic molten salt process—A new route for synthesis of nanocrystalline Li_4_Ti_5_O_12_–TiO_2_ anode material for Li-ion batteries using eutectic mixture of LiNO_3_–LiOH–Li_2_O_2_. J. Power Sources.

[340] Julien C., Camacho-Lopez M.A., Lemal M., Mohan T., Chitra S., Kalyani P., Gopukumar S. (2000). Combustion synthesis and properties of substituted lithium cobalt oxides in lithium batteries. Solid State Ion..

[341] Sha Y., Yuan T., Zhao B., Cai R., Wang H., Shao Z. (2013). Solid lithium electrolyte-Li_4_Ti_5_O_12_ composites as anodes of lithium-ion batteries showing high-rate performance. J. Power Sources.

[342] Li X., Lin H., Cui W., Xiao Q., Zhao J. (2014). Fast solution-combustion synthesis of nitrogen-modified Li_4_Ti_5_O_12_ nanomaterials with Improved electrochemical performance. ACS Appl. Mater. Interfaces.

[343] Shi W.D., Song S.Y., Zhang H.J. (2013). Hydrothermal synthesis strategies of inorganic semiconducting nanostructures. Chem. Soc. Rev..

[344] Li B., Han C., He Y.-B., Yang C., Du H., Yang Q.-H., Kang F. (2012). Facile synthesis of Li_4_Ti_5_O_12_/C composite with super rate performance. Energy Environ. Sci..

[345] Wu H.-Y., Hon M.-H., Kuan C.-Y., Leu I.-C. (2015). Hydrothermal synthesis of nanosheets as anode materials for lithium ion batteries. RSC Adv..

[346] Li N., Mei T., Zhu Y.C., Wang L., Liang J., Zhang X., Qian Y., Tang K. (2012). Hydrothermal synthesis of layered Li_1.81_H_0.19_Ti_2_O_5_·xH_2_O nanosheets and their transformation to single-crystalline Li_4_Ti_5_O_12_ nanosheets as the anode materials for Li-ion batteries. CrystEngComm.

[347] Fattakhova D., Krtil P. (2002). Electrochemical activity of hydrothermally synthesized Li-Ti-O cubic oxides toward Li insertion. J. Electrochem. Soc..

[348] Wu S.C., Guo Y.X., Zhou J.H., Zhao J., Ding X., Hu J. (2011). Effect of heat-treatment temperature on the structure and properties of Li4Ti5O12 nanorods prepared by the hydrothermal ion exchange method. J. Inorg. Mater..

[349] Zhang Z., Cao L., Huang J., Zhou S., Huang Y., Cai Y. (2013). Hydrothermal synthesis of Zn-doped Li_4_Ti_5_O_12_ with improved high rate properties for lithium ion batteries. Ceram. Int..

[350] Kim K.-T., Kim S.-J., Myung S.-T. (2013). Fast Li^+^ transfer achieved by carbon coating on Li_4_Ti_5_O_12_ nanobelts. ECS Meet. Abstr..

[351] Shi Y., Gao J., Abruna H.D., Liu H.-K., Li H. (2014). Rapid synthesis of Li_4_Ti_5_O_12_/graphene composite with superior rate capability by a microwave-assisted hydrothermal method. Nano Energy.

[352] Ding M., Liu H., Zhu J., Zhao X., Pang L., Qin Y., Deng L. (2018). Constructing of hierarchical yolk-shell structure Li_4_Ti_5_O_12_-SnO_2_ composites for high rate lithium ion batteries. Appl. Surf. Sci..

[353] He Y., Muhetaer A., Li J., Wang F., Liu C., Li Q., Xu D. (2017). Ultrathin Li_4_Ti_5_O_12_ nanosheet based hierarchical microspheres for high-rate and long-life Li-ion batteries. Adv. Energy Mater..

[354] Zhang Z., Cao L., Huang J., Wang D., Wu J., Cai Y. (2013). Hydrothermal synthesis of Li_4_Ti_5_O_12_ microsphere with high capacity as anode material for lithium ion batteries. Ceram. Int..

[355] Zhang Y., Zhang F., Li J., Yang Q. (2021). Hydrothermal synthesis and electrochemical performances of Ag-coated nanoflake-like Li_4_Ti_5_O_12_ as an anode material for lithium-ion batteries. Int. J. Electrochem. Sci..

[356] Prasad G.V., Reddy T.M., Narayana A.L., Hussain O.M., Gopal T.V., Shaikshavali P. (2023). Construction of the embedded Li_4_Ti_5_O_12_-MWCNTs nanocomposite electrode for diverse applications in electrochemical sensing and rechargeable battery. J. Inorg. Organomet. Polymers Mater..

[357] Wang M., He Y., Hong W., Zhang S.Y., Yang C.X., Yan C. (2023). Three-dimensional network microstructure design of the Li_4_Ti_5_O_12_/rGO nanocomposite as an anode material for high-performance lithium-ion batteries. J. Phys. Chem. C.

[358] Fang W., Dong E., Zhang Y., Yang L., Zhang L., Zhang H., Wang Y., Che G., Yin G. (2022). Self-assembled Li_4_Ti_5_O_12_/rGO nanocomposite anode for high power lithium-ion batteries. Inorg. Chem. Commun..

[359] Sha Y., Xu X., Li L., Cai R., Shao Z. (2016). Hierarchical carbon-coated acanthosphere-like Li_4_Ti_5_O_12_ microspheres for high-power lithium-ion batteries. J. Power Sources.

[360] Wang M., Fang P.G., Chen Y., Leng X.Y., Yan Y., Yang S.B., Xu P., Yan C. (2023). Synthesis of highly stable LTO/rGO/SnO_2_ nanocomposite via in situ electrostatic self-assembly for high-performance lithium-ion batteries. Adv. Func. Mater..

[361] Zhu M., Deng X., Li W., He M., Xiong D., Feng Z. (2022). Porous layered SnO_2_-LTO@C as a high-performance anode material for lithium-ion batteries. Ionics.

[362] Fang W., Ma Y., Zuo P., Cheng X., Yin G. (2013). Nano-Li_4_Ti_5_O_12_ pore microspheres: A high power electrode material for lithium ion batteries. Int. J. Electrochem. Sci..

[363] Kim S., Fang S.H., Zhang Z.X., Chen J.Z., Yang L., Penner-Hahn J.E., Deb A. (2014). The electrochemical and local structural analysis of the mesoporous Li_4_Ti_5_O_12_ anode. J. Power Sources.

[364] Wu F., Li X., Wang Z., Guo H. (2013). Petal-like Li_4_Ti_5_O_12_–TiO_2_ nanosheets as high-performance anode materials for Li-ion batteries. Nanoscale.

[365] Chen C., Huang Y., Zhang H., Wang X., Li G., Wang Y., Jiao L., Yuan H. (2015). Small amount of reduced graphene oxide modified Li_4_Ti_5_O_12_ nanoparticles for ultrafast high-power lithium ion battery. J. Power Sources.

[366] Yi T.-F., Yang S.-Y., Zhu Y.-R., Ye M.-F., Xie Y., Zhu R.-S. (2014). Enhanced rate performance of Li_4_Ti_5_O_12_ anode material by ethanol-assisted hydrothermal synthesis for lithium-ion battery. Ceram. Int..

[367] Zhang W., Li J., Guan Y., Jin Y., Zhu W., Guo X., Qiu X. (2013). Nano-Li_4_Ti_5_O_12_ with high rate performance synthesized by a glycerol assisted hydrothermal method. J. Power Sources.

[368] Li X.-P., Mao J. (2014). Sol-hydrothermal synthesis of Li_4_Ti_5_O_12_/rutile-TiO_2_ composite as high rate anode material for lithium ion batteries. Ceram. Int..

[369] Zhang Y., Zhang Y., Huang L., Zhou Z., Wang J., Liu H., Wu H. (2016). Hierarchical carambola-like Li_4_Ti_5_O_12_-TiO_2_ composites as advanced anode materials for lithium-ion batteries. Electrochim. Acta.

[370] Chou S.-L., Wang J.-Z., Liu H.-K., Dou S.-X. (2011). Rapid synthesis of Li_4_Ti_5_O_12_ microspheres as anode materials and its binder effect for lithium-ion battery. J. Phys. Chem. C.

[371] Hui Y., Cao L., Xu Z., Huang J., Ouyang H., Li J. (2016). Mesoporous Li_4_Ti_5_O_12_ nanoparticles synthesized by a microwave-assisted hydrothermal method for high rate lithium-ion batteries. J. Electroanal. Chem..

[372] Cheng J., Che R., Liang C., Liu J., Wang M., Xu J. (2014). Hierarchical hollow Li_4_Ti_5_O_12_ urchin-like microspheres with ultra-high specific surface area for high rate lithium ion batteries. Nano Res..

[373] Li J., Huang S., Xu S., Lan L., Lu L., Li S. (2017). Synthesis of spherical silver-coated Li_4_Ti_5_O_12_ anode material by a sol-gel-assisted hydrothermal method. Nanoscale Res. Lett..

[374] Pryono B., Murti P.B., Syahrial A.Z., Subhan A. (2017). Optimizing the performance of Li_4_Ti_5_O_12_ anode synthesized from TiO_2_ xerogel and LiOH with hydrothermal-ball mill method by using acetylene black. AIP Conference Proceedings.

[375] Gao L., Wang L., Dai S., Cao M., Zhong Z., Shen Y., Wang M. (2017). Li_4_Ti_5_O_12_-TiO_2_ nanowire arrays constructed with stacked nanocrystals for high-rate lithium and sodium ion batteries. J. Power Sources.

[376] Ma G., Cheng M. (2018). Hydrothermal method preparing lithium ion battery anode material Li_4_Ti_5_O_12_ using metatitanic acid. Ferroelectrics.

[377] Nugroho A., Kim S.J., Chung K.Y., Cho B.-W., Lee Y.-W., Kim J. (2011). Facile synthesis of nanosized Li_4_Ti_5_O_12_ in supercritical water. Electrochem. Commun..

[378] Nugroho A., Kim S.J., Chung K.Y., Kim J. (2012). Synthesis of Li_4_Ti_5_O_12_ in supercritical water for Li-ion batteries: Reaction mechanism and high-rate performance. Electrochim. Acta.

[379] Hayashi H., Nakamura T., Ebina T. (2014). Hydrothermal synthesis of Li_4_Ti_5_O_12_ nanoparticles using a supercritical flow reaction system. J. Ceram. Soc. Jpn.

[380] Nugroho A., Kim S.J., Chang W., Chung K.Y. (2013). Facile synthesis of hierarchical mesoporous Li_4_Ti_5_O_12_ microspheres in supercritical methanol. J. Power Sources.

[381] Laumann A., Bremholm M., Hald P., Holzapfel M., Fehr K.T., Iversen B.B. (2012). Rapid green continuous flow supercritical synthesis of high performance Li_4_Ti_5_O_12_ nanocrystals for Li ion battery applications. J. Electrochem. Soc..

[382] Mathew V., Lim J., Gim J., Kim D., Moon J., Kang J., Kim J. (2011). Optimized Li_4_Ti_5_O_12_ nanoparticles by solvothermal route for Li-ion batteries. J. Nanosci. Nanotechnol..

[383] Li N., Liang J., Wei D., Zhu Y., Qian Y. (2014). Solvothermal synthesis of micro-/nanoscale Cu/Li_4_Ti_5_O_12_ composites for high-rate Li-ion batteries. Electrochim. Acta.

[384] Zhang Y.-Q., Zeng M., Wu X., Bai Y., Li J. (2020). Solvothermal synthesis for dual-phase Li_4_Ti_5_O_12_/TiO_2_ composites for high-stability lithium storage. Mater. Res. Express.

[385] Chen J.Z., Yang L., Fang S.H., Tang Y.F. (2010). Synthesis of sawtooth-like Li_4_Ti_5_O_12_ nanosheets as anode materials for Li-ion batteries. Electrochim. Acta.

[386] Kageyama H., Oaki Y., Imai H. (2014). Basicity-controlled synthesis of Li_4_Ti_5_O_12_ nanocrystals by a solvothermal method. RSC Adv..

[387] Feckl J.M., Fominykh K., Döblinger M., Fattakhova-Rohlfing D., Bein T. (2012). Nanoscale porous framework of lithium titanate for ultrafast lithium insertion. Angew. Chem. Int. Ed..

[388] Kim H.-K., Roh K.C., Kang K., Kim K.-B. (2013). Synthesis of nano-Li_4_Ti_5_O_12_ decorated on non-oxidized carbon nanotubes with enhanced rate capability for lithium-ion batteries. RSC Adv..

[389] Gangaja B., Nair S., Santhanagopalan D. (2020). Surface-engineered Li_4_Ti_5_O_12_ nanostructures for high-power Li-ion batteries. Nano-Micro Lett..

[390] Santhoshkumar P., Shaji N., Sim G.S., Nanthagopal M., Lee C.W. (2022). Towards environment-friendly and versatile energy storage devices: Design and preparation of mesoporous Li_4_Ti_5_O_12_-TiO_2_ nano-hybrid electrode materials. Appl. Surf. Sci..

[391] Ho C.K., Li C.Y.V., Chan K.Y., Yung H., Tay Y.Y. (2019). Interfacing TiO2(B) nanofibers with Li4Ti5O12 towards highly reversible and durable TiO2-based anode for Li−ion batteries. Energy Technol..

[392] Lu S., Shang Y., Zheng W., Huang Y., Wang R., Zeng W., Zhan H., Yang Y., Mei J. (2022). TiO_2_(B) nanosheets modified Li_4_Ti_5_O_12_ microsphere anode for high-rate lithium-ion batteries. Nanotechnology.

[393] Singhai A., Skandan G. (2004). Nanostructured Li4Ti5O12 Powders and Method of Making the Same. US Patent.

[394] Meng T., Zeng R., Sun Z., Yi F., Shu D., Li K., Li S., Zhang F., Cheng H., He C. (2018). Chitosan-confined synthesis of N-doped and carbon-coated Li_4_Ti_5_O_12_ nanoparticles with enhanced lithium storage for lithium-ion batteries. J. Electrochem. Soc..

[395] Yin Y., Luo X., Xu B. (2022). In-situ self-assembly synthesis of low-cost, long-life, shape-controllable spherical Li_4_Ti_5_O_12_ anode material for Li-ion batteries. J. Alloys Compd..

[396] Dong H., Yin Y., Zhang Z., Yang S. (2012). Synthesis and properties of Li_4_Ti_5_O_12_/C composite by a microwave-assisted method using PAM as both the template and the carbon source. Phys. Scr..

[397] Hao X., Bartlett B.M. (2013). Li_4_Ti_5_O_12_ nanocrystals synthesized by carbon templating from solution precursors yield high performance thin film Li-ion battery electrodes. Adv. Energy Mater..

[398] Lee S.J., Jung K.H., Park B.G., Kim H.G., Park Y.J. (2010). Synthesis of Li_4_Ti_5_O_12_ thin film with inverse hemispheric structure. Bull. Korean Chem. Soc..

[399] Liu J., Wei A.X., Chen M., Xia X. (2018). Rational synthesis of Li_4_Ti_5_O_12_/N-C nanotube arrays as advanced high-rate electrodes for lithium-ion batteries. J. Mater. Chem. A.

[400] Singh D.P., Mulder F.M., Wagemaker M. (2013). Templated spinel Li_4_Ti_5_O_12_ Li-ion battery electrodes combining high rates with high energy density. Electrochem. Commun..

[401] Vertruyen B., Eshraghi N., Piffet C., Bodart J., Mahmound A., Boschini F. (2018). Spray-drying of electrode materials for lithium- and sodium-ion batteries. Materials.

[402] Nowack L.V., Bunjaku T., Wegner K., Pratsinis S.E., Luisier M., Wood V. (2015). Design and fabrication of microspheres with hierarchical internal structure for tuning performance. Adv. Sci..

[403] Wu F., Wang Z., Li X., Guo H., Yue P., Xiong X., He Z., Zhang Q. (2012). Characterization of spherical-shaped Li_4_Ti_5_O_12_ prepared by spray-drying. Electrochim. Acta.

[404] Alaboina P.K., Ge Y., Uddin M.-J., Liu Y., Lee D., Park S., Zhang X., Cho S.-J. (2016). Nanoscale porous lithium titanate anode for superior high temperature performance. ACS Appl. Mater. Interfaces.

[405] Dai C., Ye J., Zhao S., He P., Zhou H. (2016). Fabrication of high-energy Li-ion cells with Li_4_Ti_5_O_12_ microspheres as anode and 0.5 Li_2_MnO_3_·0.5 LiNi_0.4_Co_0.2_Mn_0.4_O_2_ microspheres as cathode. Chem. Asian J..

[406] Deng L., Yang W.-H., Zhou S.-X., Chen J.-T. (2015). Effect of carbon nanotubes addition on electrochemical performance and thermal stability of Li_4_Ti_5_O_12_ anode in commercial LiMn_2_O_4_/Li_4_Ti_5_O_12_ full-cell. Chin. Chem. Lett..

[407] Gao J., Jiang C., Wan C. (2010). Influence of carbon additive on the properties of spherical Li_4_Ti_5_O_12_ and LiFePO_4_ materials for lithium-ion batteries. Ionics.

[408] He Z., Wang Z., Wu F., Guo H., Li X., Xiong X. (2012). Spherical Li_4_Ti_5_O_12_ synthesized by spray-drying from a different kind of solution. J. Alloys Compd..

[409] Hsiao K.-C., Liao S.-C., Chen J.-M. (2008). Microstructure effect on the electrochemical property of Li_4_Ti_5_O_12_ as an anode material for lithium-ion batteries. Electrochim. Acta.

[410] Hsieh C.-T., Chen I.-L., Jiang Y.-R., Lin J.-Y. (2011). Synthesis of spinel lithium titanate anodes incorporated with rutile titania nanocrystallites by spray-drying followed by calcination. Solid State Ion..

[411] Jung H.-G., Kim J., Scrosati B., Sun Y.-K. (2011). Micron-sized, carbon-coated Li_4_Ti_5_O_12_ as high power anode material for advanced lithium batteries. J. Power Sources.

[412] Kadoma Y., Chiba Y., Yoshikawa D., Mitobe Y., Kumagai N., Ui K. (2012). Influence of the carbon source on the surface and electrochemical characteristics of lithium excess Li_4.3_Ti_5_O_12_ carbon composite. Electrochemistry.

[413] Lee B., Yoon J.R. (2013). Synthesis of high-performance Li_4_Ti_5_O_12_ and its application to the asymmetric hybrid capacitor. Electron. Mater. Lett..

[414] Li C., Li G., Wen S., Ren R. (2017). Spray-drying synthesis and characterization of Li_4_Ti_5_O_12_ anode material for lithium ion batteries. J. Adv. Oxid. Technol..

[415] Lu X., Gu L., Hu Y.-S., Chiu H.-C., Li H., Demopoulos G.P., Chen L. (2015). New insight into the atomic-scale bulk and surface structure evolution of Li_4_Ti_5_O_12_ anode. J. Am. Chem. Soc..

[416] Ren J., Ming H., Jia Z., Zhang Y., Ming J., Zhou Q., Zheng J. (2017). High tap density Li_4_Ti_5_O_12_ microspheres: Synthetic conditions and advanced electrochemical performance. Energy Technol..

[417] Ruan D., Kim M.-S., Yang B., Qin J., Kim K.-B., Lee S.-H., Liu Q., Tan L., Qiao Z. (2017). 700 F hybrid capacitors cells composed of activated carbon and Li_4_Ti_5_O_12_ microspheres with ultra-long cycle life. J. Power Sources.

[418] Wu F., Li X., Wang Z., Guo H., He Z., Zhang Q., Xiong X., Yue P. (2012). Low-temperature synthesis of nano-micron Li_4_Ti_5_O_12_ by an aqueous mixing technique and its excellent electrochemical performance. J. Power Sources.

[419] Yoshikawa D., Suzuki N., Kadoma Y., Ui K., Kumagai N. (2012). Li excess Li_4+x_Ti_5–x_O_12–δ_/C composite using spray-drying method and its electrode properties. Funct. Mater. Lett..

[420] Zhang Q., Peng W., Wang Z., Li X., Xiong X., Guo H., Wang Z., Wu F. (2013). Li_4_Ti_5_O_12_/reduced graphene oxide composite as a high rate capability material for lithium ion batteries. Solid State Ion..

[421] Zheng X., Dong L., Dong C. (2014). Easy synthesis of Li_4_Ti_5_O_12_/C microspheres containing nanoparticles as anode material for high-rate lithium batteries. Surf. Rev. Lett..

[422] Zhu G.-N., Liu H.-J., Zhuang J.-H., Wang C.-X., Wang Y.-G., Xia Y.-Y. (2011). Carbon-coated nano-sized Li_4_Ti_5_O_12_ nanoporous micro-sphere as anode material for high-rate lithium-ion batteries. Energy Environ. Sci..

[423] Zhu W., Zhuang Z., Yang Y., Zhang R., Lin Z., Lin Y., Huang Z. (2016). Synthesis and electrochemical performance of hole-rich Li_4_Ti_5_O_12_ anode material for lithium-ion secondary batteries. J. Phys. Chem. Solids.

[424] Wen S., Li G., Ren R., Li C. (2015). Preparation of spherical Li_4_Ti_5_O_12_ anode materials by spray drying. Mater. Lett..

[425] Xu G., Quan X., Gao H., Li J., Cai Y., Cheng X., Guo L. (2017). Facile spray drying route for large scale nitrogen-doped carbon-coated Li_4_Ti_5_O_12_ anode material in lithium-ion batteries. Solid State Ion..

[426] Yoshikawa D., Kadoma Y., Kima J.-M., Ui K., Kumagai N., Kitamura N., Idemoto Y. (2010). Spray-drying synthesized lithium-excess Li_4+x_Ti_5–x_O_1–δ_ and its electrochemical property as negative electrode material for Li-ion batteries. Electrochim. Acta.

[427] Lee B.-G., Lee S.-H. (2017). Application of hybrid supercapacitor using granule Li_4_Ti_5_O_12_/activated carbon with variation of current density. J. Power Sources.

[428] Kumagai N., Yoshikawa D., Kadoma Y., Ui K. (2010). Spray-drying synthesized lithium-excess Li_4+x_Ti_4.95−x_Nb_0.05_O_12−δ_ and its electrochemical property as negative electrode material for Li-ion batteries. Electrochemistry.

[429] Liu W., Wang Q., Cao C., Han X., Zhang J., Xie X., Xia B. (2015). Spray drying of spherical Li_4_Ti_5_O_12_/C powders using polyvinyl pyrrolidone as binder and carbon source. J. Alloys Compd..

[430] Piffet C., Vertruyen B., Caes S., Thomassin J.-M., Broze G., Malherbe C., Boschini F., Cloots R., Mahmoud A. (2020). Aqueous processing of flexible, free-standing Li_4_Ti_5_O_12_ electrodes for Li-ion batteries. Chem. Eng. J..

[431] Yuan T., Li W.-T., Zhang W., He Y.-S., Zhang C., Liao X.-Z., Ma Z.-F. (2014). One-pot spray dried graphene sheets encapsulated nano-Li_4_Ti_5_O_12_ microspheres for a hybrid BatCap system. Ind. Eng. Chem. Res..

[432] Wu F., Li X., Wang Z., Guo H. (2014). Synthesis of chromium-doped lithium titanate microspheres as high-performance anode material for lithium ion batteries. Ceram. Int..

[433] Jia X.L., Kan Y.F., Zhu X., Ning G.Q., Lu Y.F., Wei F. (2014). Building flexible Li_4_Ti_5_O_12_/CNT lithium-ion battery anodes with superior rate performance and ultralong cycling stability. Nano Energy.

[434] Wang Q., Geng J., Yuan C., Kuai L., Geng B. (2016). Mesoporous spherical Li_4_Ti_5_O_12_/TiO_2_ composites as an excellent anode material for lithium ion batteries. Electrochim. Acta.

[435] Park J.-H., Kang S.-W., Kwon T.-S., Park H.S. (2018). Spray-drying assisted synthesis of a Li_4_Ti_5_O_12_/C composite for high rate performance lithium ion batteries. Ceram. Int..

[436] Lee G.-W., Kim M.-S., Jeong J.H., Roh H.-K., Roh K.C., Kim K.-B. (2018). Comparative study of Li_4_Ti_5_O_12_ composites prepared with pristine, oxidized, and surfactant-treated multiwalled carbon nanotubes for high-power hybrid supercapacitors. ChemElectroChem.

[437] Chien W.-C., Wu Z.-H., Hsieh Y.-C., Wu Y.-S., Wu S.-H., Yang C.-C. (2020). Electrochemical performance of Li_4_Ti_5_O_12_ anode materials synthesized using a spray-drying method. Ceram. Int..

[438] Hsieh C.-T., Lin J.-Y. (2010). Influence of Li addition on charge-discharge behavior of spinel lithium titanate. J. Alloys Compd..

[439] Jamin C., Jungers T., Piffet C., Mahmoud A., Cloots R., Vertruyen B., Boschini F. (2018). Li_4_Ti_5_O_12_ powders by spray-drying: Influence of the solution concentration and particle size on the electrochemical properties. IOP Conf. Ser. J. Phys..

[440] Lenggoro I.W., Hata T., Iskandar F., Lunden M.M., Okuyama K. (2000). An experimental and modeling investigation of particle production by spray pyrolysis using a laminar flow aerosol reactor. J. Mater. Res..

[441] Zhu Y., Choi S.H., Fan X., Shin J., Ma Z., Zachariah M.R., Choi J.W., Wang C. (2017). Recent progress on spray pyrolysis for gigh performance electrode materials in lithium and sodium rechargeable batteries. Adv. Energy Mater..

[442] Laine R.M., Baranwal R., Hinklin T., Treadwell D., Sutorik A., Bickmore C., Waldner K., Neo S.S. (1999). Making nanosized oxide powders from precursors by flame spray pyrolysis. Key Eng. Mater..

[443] Ogihara T., Yamada M., Fujita A., Akao S., Myoujin K. (2011). Effect of organic acid on the electrochemical properties of Li_4_Ti_5_O_12_/C composite powders synthesized by spray pyrolysis. Mater. Res. Bull..

[444] Karhunen T., Lähde A., Leskinen J., Büchel R., Waser O., Tapper U., Jokiniemi J. (2011). Transition metal-doped lithium titanium oxide nanoparticles made using flame spray pyrolysis. ISRN Nanotechnol..

[445] Waser O., Brenner O., Groehn A.J., Pratsinis S.E. (2017). Process design for size-controlled flame spray synthesis of Li_4_Ti_5_O_12_ and electrochemical performance. Chem. Proc. Eng..

[446] Ju S.H., Kang Y.C. (2010). Effects of drying control chemical additive on properties of Li_4_Ti_5_O_12_ negative powders prepared by spray pyrolysis. J. Power Sources.

[447] Takahashi M., Tani J., Kido H., Hayashi A., Tadanaga K., Tatsumisago M. (2011). Thin film electrode materials Li_4_Ti_5_O_12_ and LiCoO_2_ prepared by spray pyrolysis method. IOP Conf. Ser. Mater. Sci. Eng..

[448] Yamada M., Kodera T., Ogihara T. (2010). Synthesis and electrochemical properties of C/Li_4_Ti_5_O_12_ powders by spray pyrolysis using aqueous solution of organic acid. Electrochemistry.

[449] Kim M.H., Kang Y.C. (2013). Electrochemical properties of nanosized Li_4_Ti_5_O_12_ powders prepared by flame spray pyrolysis. Int. J. Electrochem. Sci..

[450] Du G., Winton B.R., Hashim I.M., Sharma N., Konstantinov K., Reddy M.V., Guo Z. (2014). Mass production of Li_4_Ti_5_O_12_ with a conductive network via in-situ spray pyrolysis as a long cycle life, high rate anode material for lithium ion batteries. RSC Adv..

[451] Birrozzi A., Copley M., von Zamory J., Pasqualini M., Calcaterra S., Nobili F., Di Cicco A., Rajantie H., Briceno M., Bilbe E. (2015). Scaling up “nano” Li_4_Ti_5_O_12_ for high-power lithium-ion anodes using large scale flame spray pyrolysis. J. Electrochem. Soc..

[452] Zhang X., Xu H., Zhao Y., Zhu G., Yu A. (2014). A facile one-step spray pyrolysis method to synthesize spherical Li_4_Ti_5_O_12_ for lithium-ion battery. Mater. Lett..

[453] Yang K.M., Ko Y.N., Yun J.-Y., Kang Y.C. (2014). Preparation of Li_4_Ti_5_O_12_ yolk–shell powders by spray pyrolysis and their electrochemical properties. Chem. Asian J..

[454] Kim J.H., Yoon J.R. (2013). Preparation and characterization of Li_4_Ti_5_O_12_ synthesized using hydrogen titanate nanowire for hybrid super capacitor. J. Adv. Ceram..

[455] Bhat M.H., Miura A., Vinatier P., Levasseur A., Rao K.J. (2003). Microwave synthesis of lithium lanthanum titanate. Solid State Commun..

[456] Li J., Jin Y.-L., Zhang X.-G., Yang H. (2007). Microwave solid-state synthesis of spinel Li_4_Ti_5_O_12_ nanocrystallites as anode material for lithium-ion batteries. Solid State Ion..

[457] Balaji S., Mutharasu D., Sankara Subramanian N., Ramanathan K. (2009). A review on microwave synthesis of electrode materials for lithium-ion batteries. Ionics.

[458] Yang L.H., Dong C., Guo J. (2008). Hybrid microwave synthesis and characterization of the compounds in the Li-Ti-O system. J. Power Sources.

[459] Liu H.W., Feng C.Q., Tang H., Song L., Zhang K.L. (2004). Synthesis and electrochemical properties of indium doped spinel LiMn_2_O_4_. J. Mater. Sci..

[460] Liu H., Feng Y., Wang K., Xie J. (2008). Synthesis and electrochemical properties of Li_4_Ti_5_O_12_/C composite by the PVB rheological phase method. J. Phys. Chem. Solids.

[461] Wang Z., Xie G., Gao L. (2012). Electrochemical characterization of Li_4_Ti_5_O_12_/C anode material prepared by starch-sol-assisted rheological phase method for Li-ion battery. J. Nanomater..

[462] Abureden S., Hassan F.M., Lui G., Ahn W., Sy S., Yu A.P., Chen Z.W. (2016). Multigrain electrospun nickel doped lithium titanate nanofibers with high power lithium ion storage. J. Mater. Chem. A.

[463] Zhu N., Liu W., Xue M., Xie Z., Zhao D., Zhang M., Chen J., Cao T. (2010). Graphene as a conductive additive to enhance the high-rate capabilities of electrospun Li_4_Ti_5_O_12_ for lithium-ion batteries. Electrochim. Acta.

[464] Guo B., Li Y., Yao Y., Lin Z., Ji L., Xu G., Liang Y., Shi Q., Zhang X. (2011). Electrospun Li_4_Ti_5_O_12_/C composites for lithium-ion batteries with high rate performance. Solid State Ion..

[465] Wang L., Xiao Q., Li Z., Lei G., Zhang P., Wu L. (2012). Synthesis of Li_4_Ti_5_O_12_ fibers as a high-rate electrode material for lithium-ion batteries. J. Solid State Electrochem..

[466] Ji X., Li D., Lu Q., Guo E., Yao L. (2017). Electrospinning preparation of one-dimensional Ce^3+^-doped Li_4_Ti_5_O_12_ sub-microbelts for high-performance lithium-ion batteries. J. Nanopart. Res..

[467] Li X., Yang K., Gao F., Hu C., Liu H., Li D., Wang L. (2015). Electrospinning synthesis of spinel Li_4_Ti_5_O_12_ and its characterization. IOP Conf. Ser. Mater. Sci. Eng..

[468] Park H., Song T., Han H., Paik U. (2013). Electrospun Li_4_Ti_5_O_12_ nanofibers sheathed with conductive TiN/TiOxNy layer as an anode material for high power Li-ion batteries. J. Power Sources.

[469] Chen C.H., Feng J.J., Shao W.Q., Chen S.O., He L.Z., Li Y.Q., Ban Y.Q., Hei F., Guo X.J. (2016). Electrochemical performance of Li_4_Ti_5_O_12_/TiO_2_/Li_0.4_TiO_2_/C composite prepared by the electrospinning method using as lithium battery anode material. Mater. Sci. Forum.

[470] Zou H.L., Xiang H.F., Liang X., Feng X.Y., Cheng S., Jin Y., Chen C.H. (2017). Electrospun Li_3.9_Cr_0.3_Ti_4.8_O_12_ nanofibers as anode material for high-rate and low-temperature lithium-ion batteries. J. Alloys Compd..

[471] Kim J.-G., Shi D., Park M.-S., Jeong G., Heo Y.-U., Seo M., Kim Y.-J., Kim J.H., Dou S.X. (2013). Controlled Ag-driven superior rate-capability of Li_4_Ti_5_O_12_ anodes for lithium rechargeable batteries. Nano Res..

[472] Zhang Z., Deng X., Sunarso J., Cai R., Chu S., Miao J., Zhou W., Shao Z. (2017). Two-step fabrication of Li_4_Ti_5_O_12_-coated carbon nanofibers as a flexible film electrode for high-power lithium-ion batteries. ChemElectroChem.

[473] Zhou Y., Xiao S., Jiang J., Wu R., Niu X., Chen J.S. (2023). In-situ construction of Li_4_Ti_5_O_12_/rutile TiO_2_ heterostructured nanorods for robust and high-power lithium storage. Nano Res..

[474] Wang J., Shen L., Li H., Ding B., Nie P., Dou H., Zhang X. (2014). Mesoporous Li_4_Ti_5_O_12_/carbon nanofibers for high-rate lithium-ion batteries. J. Alloys Compd..

[475] Liu C., Wang S., Zhang C., Fu H., Nan X., Yang Y. (2016). High power high safety battery with electrospun Li_3_V_2_(PO_4_)_3_ cathode and Li_4_Ti_5_O_12_ anode with 95% energy efficiency. Energy Storage Mater..

[476] Wu Y., Reddy M.V., Chowdari B.V.R., Ramakrishna S. (2012). Electrochemical studies on electrospun Li(Li_1/3_Ti_5/3_)O_4_ grains as an anode for Li-ion batteries. Electrochim. Acta.

[477] Kim E.-K., Choi B.-H., Jee M.-J., Kwon Y.-J., Seo H., Kim Y.-J., Kim K.-B. (2010). Synthesis and electrochemical characteristics of Li_4_Ti_5_O_12_ nanofibers by hydrothermal method. J. Korean Ceram. Soc..

[478] Kataoka K., Nagai H., Sotokawa T., Kumashiro Y., Ishibai Y., Akimoto J. (2016). Ion-exchange synthesis and improved Li insertion property of lithiated H_2_Ti_12_O_25_ as a negative electrode material for lithium-ion batteries. J. Asian Ceram. Soc..

[479] Zhang J., Zhang F., Li J., Cai W., Zhang J., Yu L., Jin Z., Zhang Z. (2013). Preparation of Li_4_Ti_5_O_12_ by solution ion-exchange of sodium titanate nanotube and evaluation of electrochemical performance. J. Nanopart. Res..

[480] Liao J.-Y., Chabot V., Gu M., Wang C., Xiao X., Chen Z. (2014). Dual phase Li_4_Ti_5_O_12_-TiO_2_ nanowire arrays as integrated anodes for high-rate lithium-ion batteries. Nano Energy.

[481] Li N., Katase T., Zhu Y., Matsumoto T., Umemura T., Ikuhara Y., Ohta H. (2016). Solid-liquid phase epitaxial growth of Li_4_Ti_5_O_12_ thin film. Appl. Phys. Express.

[482] Wang C.-L., Liao Y.C., Hsu F.C., Tai N.H., Wu M.K. (2005). Preparation and characterization of thin film Li_4_Ti_5_O_12_ electrodes by magnetron sputtering. J. Electrochem. Soc..

[483] Kumatani A., Shiraki S., Takagi Y., Suzuki T., Ohsawa T., Gao X., Ikuhara Y., Hitosugi T. (2014). Epitaxial growth of Li_4_Ti_5_O_12_ thin films using RF magnetron sputtering. Jpn. J. Appl. Phys..

[484] Pagani F., Döbeli M., Battaglia C. (2021). Lithium-ion transport in Li_4_Ti_5_O_12_ epitaxial thin films vs state of charge. Batter. Supercaps.

[485] Deng J.Q., Lu Z.G., Belharouak I., Amine K., Chung C.Y. (2009). Preparation and electrochemical properties of Li_4_Ti_5_O_12_ thin film electrodes by pulsed laser deposition. J. Power Sources.

[486] Kumatani A., Ohsawa T., Shimizu R., Takagi Y., Shiraki S., Hitosugi T. (2012). Growth processes of lithium titanate thin films deposited by using pulsed laser deposition. Appl. Phys. Lett..

[487] Yu P., Li C., Guo X. (2014). Sodium storage and pseudocapacitance charge in textured Li_4_Ti_5_O_12_ thin films. J. Phys. Chem. C.

[488] Yu X., Wang R., He Y., Hu Y., Li H., Huang X. (2010). Electrochromic behavior of transparent Li_4_Ti_5_O_12_/FTO electrode. Electrochem. Solid-State Lett..

[489] Deng J., Lu Z., Chung C.Y., Hanc X., Wang Z., Zhou H. (2014). Electrochemical performance and kinetic behavior of lithium ion in Li_4_Ti_5_O_12_ thin film electrodes. Appl. Surf. Sci..

[490] Zhao M., Lian J., Jia Y., Jin K., Xu L., Hu Z., Yang X., Kang S. (2016). Investigation of the optical properties of LiTi_2_O_4_ and Li_4_Ti_5_O_12_ spinel films by spectroscopic ellipsometry. Opt. Mater. Express.

[491] Schichtel P., Geiß M., Leichtweiß T., Sann J., Weber D.A., Janek J. (2017). On the impedance and phase transition of thin film all-solid-state batteries based on the Li_4_Ti_5_O_12_ system. J. Power Sources.

[492] Julien C.M., Mauger A. (2018). Pulsed laser deposited films for energy storage and conversion: A review. Coatings.

[493] Rho Y.H., Kanamura K., Umegaki T. (2001). Preparation of Li_4/3_Ti_5/3_O_4_ thin film anode with high electrochemical response for rechargeable lithium batteries by sol–gel method. Chem. Lett..

[494] Rho Y.H., Kanamura K., Fujisaki M., Hamagami J., Suda S., Umegaki T. (2002). Preparation of Li_4_Ti_5_O_12_ and LiCoO_2_ thin film electrodes from precursors obtained by sol-gel method. Solid State Ion..

[495] Wude F., Berkemeier F., Schmitz G. (2012). Lithium diffusion in sputter-deposited Li_4_Ti_5_O_12_ thin films. J. Power Sources.

[496] Hirayama M., Kim K., Toujigamori T., Cho W., Kanno R. (2011). Epitaxial growth and electrochemical properties of Li_4_Ti_5_O_12_ thin-film lithium battery anodes. Dalton Trans..

[497] Kim K., Toujigamori T., Suzuki K., Taminato S., Tamura K., Mizuki J., Hirayama M., Kanno R. (2012). Characterization of nano-sized epitaxial Li_4_Ti_5_O_12_ (110) film electrode for lithium batteries. Electrochemistry.

[498] Pfenninger R., Afyon S., Garbayo I., Struzik M., Rupp J.L.M. (2018). Lithium titanate anode thin films for Li-ion solid state battery based on garnets. Adv. Funct. Mater..

[499] Mesoraca S., Kleibeuker J.E., Prasad B., MacManus-Driscoll J.L., Blamire M.G. (2016). Lithium outdiffusion in LiTi_2_O_4_ thin films grown by pulsed laser deposition. J. Cryst. Growth.

[500] Chopdekar R.V., Wong F.J., Takamura Y., Arenholz E., Suzuki Y. (2009). Growth and characterization of superconducting spinel oxide LiTi_2_O_4_ thin films. Phys. C Supercond..

[501] Pagani F., Stilp E., Pfenninger R., Reyes E.R., Remhof A., Balogh-Michels Z., Neels A., Sastre-Pellicer J., Stiefel M., Döbeli M. (2018). Epitaxial thin films as a model system for Li-ion conductivity in Li_4_Ti_5_O_12_. ACS Appl. Mater. Interfaces.

[502] Cunha D.M., Hendriks T.A., Vasileiadis A., Vos C.M., Verhallen T., Singh D.P., Wagemaker M., Huijben M. (2019). Doubling reversible capacities in epitaxial Li_4_Ti_5_O_12_ thin film anodes for microbatteries. ACS Appl. Energy Mater..

[503] Daminabo S., Goel S., Grammatikos S., Yazdani Nezhad H., Thakur V.K. (2020). Fused deposition modeling-based additive manufacturing (3D printing): Techniques for polymer material systems. Mater. Today Chem..

[504] Sun K., Wei T.S., Ahn B.Y., Seo J.Y., Dillon S.J., Lewis J.A. (2013). 3D Printing of interdigitated Li-ion microbattery architectures. Adv. Mater..

[505] Zhao Y., Liu G., Liu L., Jiang Z. (2009). High-performance thin-film Li_4_Ti_5_O_12_ electrodes fabricated by using ink-jet printing technique and their electrochemical properties. J. Solid State Electrochem..

[506] Chen Q., Xu R., He Z., Zhao K., Pan L. (2017). Printing 3D gel polymer electrolyte in lithium-ion microbattery using stereolithography. J. Electrochem. Soc..

[507] Delannoy P.E., Riou B., Lestriez B., Guyomard D., Brousse T., Le Bideau J. (2015). Toward fast and cost-effective ink-jet printing of solid electrolyte for lithium microbatteries. J. Power Sources.

[508] Zhou L., Ning W., Wu C., Zhang D., Wei W., Ma J., Li C., Chen L. (2018). 3D-printed microelectrodes with a developed conductive network and hierarchical pores toward high areal capacity for microbatteries. Adv. Mater. Technol..

[509] Wei T.S., Ahn B.Y., Grotto J., Lewis J.A. (2018). 3D printing of customized Li-ion batteries with thick electrodes. Adv. Mater..

[510] Wang Y., Chen C., Xie H., Gao T., Yao Y., Pastel G., Han X., Li Y., Zhao J., Fu K. (2017). 3D-printed all-fiber Li-ion battery toward wearable energy storage. Adv. Funct. Mater..

[511] Kohlmeyer R.R., Blake A.J., Hardin J.O., Carmona E.A., Carpena-Núñez J., Maruyama B., Daniel Berrigan J., Huang H., Durstock M.F. (2016). Composite batteries: A simple yet universal approach to 3D printable lithium-ion battery electrodes. J. Mater. Chem. A.

[512] Ragones H., Menkin S., Kamir Y., Gladkikh A., Mukra T., Kosa G., Golodnitsky D. (2018). Towards smart free form-factor 3D printable batteries. Sustain. Energy Fuels.

[513] Kim H., Choi J.-M., Kwon M.-S., Song M.S., Park Y., Doo S.-G. (2009). Inkjet printed electrode for rechargeable lithium thin film battery. ECS Meet. Abstr..

[514] Lawes S., Sun Q., Lushington A., Xiao B., Liu Y., Sun X. (2017). Inkjet-printed silicon as high performance anodes for Li-ion batteries. Nano Energy.

[515] Viviani P., Gibertini E., Iervolino F., Levi M., Magagnin L. (2021). Carbon additive effect on the electrochemical performances of inkjet printed thin-film Li_4_Ti_5_O_12_ electrodes. J. Manufact. Proc..

[516] Zhao E., Qin C., Jung H.-R., Berdichevsky G., Nese A., Marder S., Yushin G. (2016). Lithium titanate confined in carbon nanopores for asymmetric supercapacitors. ACS Nano.

[517] Zhu J., Chen J., Xu H., Sun S., Xu Y., Zhou M., Gao X., Sun Z. (2019). Plasma-introduced oxygen defects confined in Li_4_Ti_5_O_12_ nanosheets for boosting lithium-ion diffusion. ACS Appl. Mater. Interfaces.

[518] Kitta M., Kojima T., Kataoka R., Yazawa K., Tada K. (2020). Realizing the single-phase spinel-type sodium titanium oxide with the Li_4_Ti_5_O_12_-like structure for building stable sodium-ion batteries. ACS Appl. Mater. Interfaces.

[519] Ritala M., Leskela M., Rauhala E., Haussalo P. (1995). Atomic layer epitaxy growth of TiN thin films. J. Electrochem. Soc..

[520] Richard M.N., Fuller E.W., Dahn J.R. (1994). the effect of ammonia reduction on the spinel electrode materials, LiMn_2_O_4_ and Li(Li_1/3_Mn_5/3_)O_4_. Solid State Ion..

[521] Santucci S., Lozzi L., Passacantando M., Picozzi P., Alfonsetti R., Diamanti R., Moccia G. (1996). Study by X-ray photoelectron spectroscopy and X-ray diffraction of the growth of TiN thin films obtained by nitridation of Ti layers. Thin Solid Films.

[522] Wan Z.N., Cai R., Jiang S.M., Shao Z.P. (2012). Nitrogen- and TiN-modified Li_4_Ti_5_O_12_: One-step synthesis and electrochemical performance optimization. J. Mater. Chem..

[523] Kahrisi M., Kashani H., Ghaffarinejad A. (2023). Improving the cyclability and rate capability of Li_4_Ti_5_O_12_ anode material by Cu^2+^ and F^−^ co-doping. ChemistrySelect.

[524] Ali B., Muhammad R., Islam M., Anang D.A., Han D.-S., Moeez I., Chung K.Y., Cho M., Kim J.Y., Kim M.G. (2023). Cd-doped Li_4–*x*_Cd*_x_*Ti_5_O_12_ (*x* = 0.20) as a high rate capable and stable anode material for lithium-ion batteries. ACS Appl. Energy Mater..

[525] Noreochim L., Prabowo R.S., Widyastuti W., Susanti D., Subhan A., Idris N.H. (2023). Enhanced high-rate capability of iodide-doped Li_4_Ti_5_O_12_ as an anode for lithium-ion batteries. Batteries.

[526] Qasim K.F., Mousa M.A. (2023). Physicochemical properties of oriented crystalline assembled polyaniline/metal doped Li_4_Ti_5_O_12_ composites for Li-ion storage. J. Inorg. Organomet. Polym..

[527] Yeo S., Raj M.R., Lee G. (2023). Oxygen vacancy-modulated zeolitic Li_4_Ti_5_O_12_ microsphere anode for superior lithium-ion battery. Electrochim. Acta.

[528] Tian K., Hui X., Wang H., Zhang Z., Zhang L., Wang C., Yin L. (2022). *In situ* growth of Li_4_Ti_5_O_12_ nanoparticles on Ti_3_C_2_T_x_ MXene for efficient electron transfer as high-rate anode of Li ion battery. Electrochim. Acta.

[529] Li Y., Zhang W., Lai C., Yang T., Chang X., Zhang M., Sheng L., Yang Z., Ye D., Huang K. (2023). Ti_3_C_2_ MXene-derived Li_4_Ti_5_O_12_ nanoplates with in-situ formed carbon quantum dots for metal-ion battery anodes. J. Colloid Interface Sci..

[530] Meng Q., Hao Q., Chen F., Wang L., Li N., Sun X. (2023). Li_4_Ti_5_O_12_ hollow macrospheres combine high tap density and excellent low-temperature performance as anode materials for Li-ion batteries. Mater. Character..

[531] Liang K., Ren Y. (2020). Research progress on lithium titanate as anode material for sodium-ion batteries. Mater. Rep..

[532] Doyle M., Newman J. (1995). The use of mathematical modeling in the design of lithium/polymer battery systems. Electrochim. Acta.

[533] Christensen J., Srinivasan V., Newman J. (2006). Optimization of lithium titanate electrodes for high-power cells. J. Electrochem. Soc..

[534] Kashkooli A.G., Lui G., Farhad S., Lee D.U., Feng K., Yu A., Chen Z. (2016). Nano-particle size effect on the performance of Li_4_Ti_5_O_12_ spinel. Electrochim. Acta.

[535] Lim J., Choi E., Mathew V., Kim D., Ahn D., Gim J., Kang S.-H., Kim J. (2011). Enhanced high-rate performance of Li_4_Ti_5_O_12_ nanoparticles for rechargeable Li-ion batteries. J. Electrochem. Soc..

